# The potential of phenothiazinium dyes as cytotoxicity markers in cisplatin-treated cells

**DOI:** 10.1038/s41598-023-36721-0

**Published:** 2023-06-23

**Authors:** Luiz Miguel Pereira, Gisele Bulhões Portapilla, Guilherme Thomaz Pereira Brancini, Bruna Possato, Cássia Mariana Bronzon da Costa, Péricles Gama Abreu-Filho, Mark Wainwright, Ana Patrícia Yatsuda, Gilberto Úbida Leite Braga

**Affiliations:** 1 Faculdade de Ciências Farmacêuticas de Ribeirão Preto, Universidade de São Paulo, Av do Café, sn/n, Ribeirão Preto, SP 14040-903 Brazil; 2 Department of Biology, Edge Hill University, Ormskirk, L39 4QP UK; 3 Departamento de Análises Clínicas, Bromatológicas e Toxicológicas, Faculdade de Ciências Farmacêuticas de Ribeirão Preto, Universidade de São Paulo, Ribeirão Preto, 14040-903 Brazil

**Keywords:** Biological techniques, Cell biology, Drug discovery

## Abstract

Assessing the in vitro toxicity of compounds on cell cultures is an important step during the screening of candidate molecules for diverse applications. Among the strategies employed to determine cytotoxicity, MTT, neutral red, and resazurin are commonly used. Methylene blue (MB), a phenothiazinium salt, has several uses, such as dye, redox indicator, and even as treatment for human disease and health conditions, such as malaria and methemoglobinemia. However, MB has only been sparsely used as a cellular toxicity indicator. As a viability indicator, MB is mostly applied to fixed cultures at high concentrations, especially when compared to MTT or neutral red. Here we show that MB and its related compounds new methylene blue (NMB), toluidine blue O (TBO), and dimethylmethylene blue (DMMB) can be used as cytotoxicity indicators in live (non-fixed) cells treated for 72 h with DMSO and cisplatin. We compared dye uptake between phenothiazinium dyes and neutral red by analyzing supernatant and cell content via visible spectra scanning and microscopy. All dyes showed a similar ability to assess cell toxicity compared to either MTT or neutral red. Our method represents a cost-effective alternative to in vitro cytotoxicity assays using cisplatin or DMSO, indicating the potential of phenothiazinium dyes for the screening of candidate drugs and other applications.

## Introduction

The approval of new molecules for use in humans and animals depends on the evaluation of many properties, one of the most critical of which is toxicity. Assessment of toxicity for candidate compounds is initially performed via incubation with mammalian cells^1^. Then, several parameters pertaining to cell integrity, cell viability, and/or metabolism are evaluated^2–4^. Vital dyes, as the name implies, accumulate in live cells and allow the measuring of cell viability. For instance, trypan blue, methylene blue (MB), erythrosine B, nigrosine, eosin, safranin, propidium iodide, and 7-aminoactinomycin D have been applied for the determination of cell viability for diverse purposes^5^. Additionally, there are indicators that measure the activity of metabolic pathways related to cell viability; these are exemplified by the tetrazolium salts MTT, XTT, WST-1, and MDS, which have also been widely used for cell viability assays. For example, MTT is reduced by mitochondrial dehydrogenases in viable cells to MTT formazans, which are spectrophotometrically quantified at 570 nm^6,7^. Similarly, resazurin (Alamar blue) is also used for cell viability assays, in which the molecule (a blue dye) is reduced to the highly fluorescent resorufin by intracellular diaphorase enzymes^8,9^.

Molecules incorporated by live cells are also used as indicators of toxicity in drug screening strategies. For example, neutral red (NR) is a dye that is protonated (ionized) at low pH and thus accumulates in lysosomes due to the low pH of these organelles. Therefore, drugs that interfere with cell membranes and/or lysosomes decrease NR uptake, allowing the dye to serve as a cell viability indicator^10,11^. In this sense, MB is also applied as an indicator of cytotoxicity in mammalian cell culture because it accumulates in non-damaged cells. However, MB is mostly used as a cell viability indicator after formalin fixation and, for this application, is also used at higher concentrations (1% w/v) compared to NR (50 µg/ml; 0.005% w/v)^12,13^.

In the present study, we evaluated a method based on the accumulation of MB and its related compounds new methylene blue (NMB), toluidine blue O (TBO), and dimethylmethylene blue (DMMB) in live cells after treatment with DMSO and cisplatin. Cisplatin or *cis*-diamminedichloridoplatinum(II) is one of the most potent and widely used drugs for the treatment of solid tumors^14^. The most accepted mechanism of cisplatin is based on its interaction with purine bases, which results in DNA lesions leading to cell apoptosis^15^. However, side effects and drug resistance are constant challenges of cisplatin treatment^16,17^. This underscores the importance of novel strategies such as drug modifications or combinations to minimize side effects^18,19^. In this sense, the development of new cisplatin therapies depends on in vitro cytotoxicity assays, especially those with different mechanisms of detection that are capable of complementing classic methods (i.e. MTT, NR, and resazurin). Here we evaluated the potential of phenothiazinium dyes as indicators of cytotoxicity in cisplatin-sensitive and -resistant cell lineages. After 72 h of cisplatin treatment, phenothiazinium dyes showed a similar pattern of cell viability compared to MTT and NR. The use of phenothiazinium dyes for the evaluation of cisplatin cytotoxicity indicates an alternative, efficient, and cost-effective method for in vitro cell viability assays.

## Materials and methods

### Drugs and dyes

All compounds were purchased from Sigma-Aldrich. Four phenothiazinium dyes, namely MB (3,7-bis[dimethylamino]phenothiazin-5-ium chloride, catalogue number: 1428008), NMB (3,7-bis(ethylamino)-2,8-dimethylphenothiazin-5-ium chloride, catalogue number: R313718), TBO (3-amino-7-(dimethylamino)-2-methylphenothiazin-5-ium chloride, catalogue number: T3260), and DMMB (3,7-bis(dimethylamino)-1,9-dimethylphenothiazin-5-ium chloride, catalogue number: 341088) were used for toxicity evaluation and were compared to MTT (3-(4,5-Dimethyl-2-thiazolyl)-2,5-diphenyl-2H-tetrazolium bromide, catalogue number: M5655) and NR (3-amino-7-dimethylamino-2-methylphenazine hydrochloride, catalogue number: N4638). The dyes were stocked in water at 5 mg/ml and stored according to manufacturer’s instructions. For cytotoxicity assays, DMSO (dimethyl sulfoxide, catalogue number: 276855) and cisplatin (cis-diammineplatinum (II) dichloride, catalogue number: C2210000) were used and diluted in phenol red-free RPMI (Sigma-Aldrich, catalogue number: R8755) before the procedure (stocks of 40% and 0.5 mg/ml, respectively).

### Cell lines

Vero (African green monkey kidney epithelial cells), LLCMK (rhesus monkey kidney epithelial cells), human primary fibroblasts, MCF 10A (human mammary epithelial cells) and MDA-MB-231 (human breast adenocarcinoma) were used in this study. Vero and fibroblast cells were kindly gifted by Professor Solange Maria Gennari (FMVZ, Universidade de São Paulo, Brazil) and Professor Fabíola Attié de Castro (FCFRP, Universidade de São Paulo, Brazil), respectively. LLCMK, MCF 10A and MDA-MB-231 cells were kindly gifted by Professor Sergio Albuquerque (FCFRP, Universidade de São Paulo, Brazil). Cells were maintained in 75-cm^2^ flasks (Kasvi) in RPMI supplemented with 10% fetal bovine serum (FBS, Gibco) and propagated after trypsin treatment (Gibco) (Gibco)^20^.

### Detection of the phenothiazinium dyes accumulation in cells

The accumulation of phenothiazine dyes in cells was detected by light and confocal microscopies. Firstly, Vero cells were incubated with MB, NMB, TBO, and DMMB (100, 50, 25 and 12.5 µM) for 3 h, 37 °C, 5% CO_2_ and washed with PBS. The cells were observed in a light microscope and representative pictures of each incubation regimen acquired. For confocal detection of phenothiazinium dyes, Vero cell cultures were incubated with 10 μM of MB, NMB, TBO or DMMB for 30 min, 37 °C, with 5% CO_2_. For all procedures, non-treated controls were incubated with RPMI under the same conditions. The cultures were washed with PBS and treated with trypsin for 10 min, 37 °C and 5% CO_2_. The cells were transferred to slides and analyzed by confocal microscopy with excitation/emission wavelengths of 543/600 nm, respectively. The slides were observed in a TCS-SP8 AOBS (Leica Microsystems), using a 63× objective and processed by ImageJ software (version 1.53j, National Institute of Health, USA).

### Calculation of the logarithm of the octanol–water partition coefficient (log P) and the negative logarithmic of the acid dissociation constant (pK_a_)

To predict lipophilicity and localization of the phenothiazinium dyes in cells, we used log P and pK_a_ values as described in the literature^21,22^. Thus, log P was estimated using the method proposed by Ref.^23^, as defined by Ref.^24^ as being the “sum of the log P of the parent solute plus a π term” (Eqs. 1, 2). Reference^25^ defined that the rate of penetration of a given species to an active site is a parabolic function, that could be simplified by using π, a substituent constant derived from the partition coefficients, defined numerically as:

1 $$\pi \left(X\right)={\mathrm{log}P}_{(R{-}X) }-{\mathrm{log}P}_{\left(R{-}H\right)}.$$

Therefore, by adding π values to a log P, we have:

2 $${\mathrm{log}P}_{Y{-}R{-}X}={\mathrm{log}P}_{H{-}R{-}H}+{\pi }_{Y}+{\pi }_{X}.$$

Besides the calculations applying Eq. (2) and experimentally obtained π values, presented too by Ref.^24^, we also resorted to in silico calculations of log P, pK_a_ and log D of MB, NMB, TBO and DMMB using ChemAxon’s MarvinSketch (version 23.5) and its protonation and lipophilicity calculators. The molecular structures adopted were those for which the optimization is available at PubChem (https://pubchem.ncbi.nlm.nih.gov/). ChemDraw (version 21.0.0) was also used to draw the molecules shown in the text. The reference for pH values at physiologic media, lysosomes and mitochondria was obtained from Ref.^26^.

### Visible absorption spectra of phenothiazinium dyes and their cell uptake

Initially, to evaluate the absorption of dyes by cells, MB, NMB, TBO, and DMMB were diluted in phenol red-free RPMI (100 µM) and incubated with Vero cell monolayers (in 24-well plates) for 4 h at 37 °C and 5% CO_2_. An aliquot (200 µl in duplicate) of the supernatants were collected at intervals of 1 h (0, 1, 2, 3, and 4 h) and divided in two equal parts: the first part was homogenized with an alcohol-acid solution (2% acetic acid in absolute ethanol) and the second part was used as control. After incubation with phenothiazinium dyes, cell monolayers were washed with 100 µl of fixing solution (1% CaCl_2_ and 0.5% formaldehyde), followed by dye extraction with 250 µl of extraction solution (1% acetic acid in 50% ethanol). All samples (supernatants and cell extracts) were analyzed via visible light spectrophotometry in an ELISA reader (Epoch Biotek) and the area under the spectral curve (cell extracts) was calculated (using the absorbance values from 380 to 800 nm). The values from the incubated samples (1, 2, 3, and 4 h) were used to calculate the percentage of dye compound uptake relative to the initial absorbance (0 h). Three independent experiments were performed.

### Sensitivity assay for the detection of phenothiazinium dyes in Vero cells

Vero cells were distributed in 96-well plates and cultivated until confluence was achieved. Then, cells were incubated with 10 serial dilutions of MB, NMB, TBO, and DMMB (starting at 250 µM) for 3 h at 37 °C and 5% CO_2_. After incubation, cells were fixed with 100 µl of fixing solution and incorporated dye was extracted with 100 µl extraction solution. The detection of phenothiazinium dyes were performed in an ELISA reader at 660 nm, 630 nm, 630 nm and 650 nm for MB, NMB, TBO, and DMMB, respectively. The difference between the absorbance values of the dilutions and the control (composed of free dye RPMI) was applied as a sensitivity indicator of dyes in cells. Two independent experiments were performed.

### Linearity of phenothiazinium dyes incorporation in Vero cells

To evaluate the linearity of phenothiazinium dye incorporation, Vero cell monolayers were treated with trypsin for 15 min at 37 °C and 5% CO_2_. The cells were counted in a hemocytometer and suspended in RPMI (without FBS). The suspensions were serially diluted in 96-well plates (1 × 10^5^, 5 × 10^4^, 2.5 × 10^4^, 1.2 × 10^4^, 6.2 × 10^3^, 3.1 × 10^3^ and 1.5 × 10^3^ cells/well) and cultivated for 24 h at 37 °C and 5% CO_2_. After the cultivation, the medium was carefully removed and the cells incubated with phenothiazinium dyes (100 µM), NR (50 µg/ml) or MTT (0.5 mg/ml) for 3 h at 37 °C and 5% CO_2_. The supernatant was discarded, and the samples (stained with MB, NMB, TBO, DMMB or NR) were fixed with 100 µl fixing solution and the dyes were extracted with 100 µl of extraction solution. For MTT, the formazan crystals were diluted with 100 µl DMSO. Wells containing no cells were used as control. The plates were read at 660 nm (MB), 630 nm (NMB and TBO), 650 nm (DMMB), 540 nm (NR) or 570 nm (MTT) in an ELISA reader (Epoch Biotek). The difference between the absorbance values of the samples and the blank was applied for plotting absorbance × cell density graphs. The curves were analyzed by linear regression using the GraphPad 8.0 software (San Diego, California USA) and r^2^ values were calculated. Two independent experiments were performed.

### Cytotoxicity by phenothiazinium dyes, NR, and MTT

Vero cells, LLC-MK2, human primary fibroblasts, MCF 10A, and MDA-MB-231 cell lines were cultivated in 96-well plates at 37 °C and 5% CO_2_ until confluence (> 90%) was achieved. Cells were treated with either DMSO (Vero cells and primary fibroblasts) or cisplatin (LLC-MK2, MCF 10A, and MDA-MB-231). The cultures were incubated with seven serial dilutions of DMSO or cisplatin (starting at 20% and 200 µM, respectively) for 72 h at 37 °C and 5% CO_2_ in phenol red-free RPMI. The supernatant was discarded, cells were washed with phosphate-buffered saline (PBS), and the cultures were incubated with 100 µM of the phenothiazinium dyes (MB, NMB, TBO, and DMMB), MTT (0.5 mg/ml) or NR (50 µg/ml; 173 µM) for 3 h at 37 °C and 5% CO_2_ in duplicate. After treatment with DMSO and cisplatin, labeling with MTT or NR was performed as described previously^27,28^ with modifications^29,30^. Cells incubated with phenothiazinium dyes were processed by the same protocol used for NR. Briefly, samples labelled with MB, NMB, TBO, DMMB, or NR were quickly washed with a fixing solution (1% CaCl_2_ and 0.5% formaldehyde) and diluted with 100 µl of NR extraction solution. The cells incubated with MTT were diluted with 100 µl of DMSO. The plates were read at 540 nm (NR) or 570 nm (MTT) in an ELISA reader (Epoch Biotek). Three (LLC-MK2 and MDA-MB-231) and two (Vero cells, human primary fibroblasts and MCF 10A) independent experiments were performed.

### Statistical analyses

Values for area under the curve were calculated with GraphPad Prism 8.0 software (San Diego, California USA). For cells incubated with DMSO or cisplatin, the percentage of inhibition was calculated from the absorbance of treated samples relative to their non-treated controls. These values were used to calculate the half-maximal inhibitory concentration (IC_50_) of cisplatin and DMSO, which was done with Compusyn software (version 1.0)^31^.

## Results

### Accumulation of phenothiazinium dyes in Vero cells

**Figure 1:**
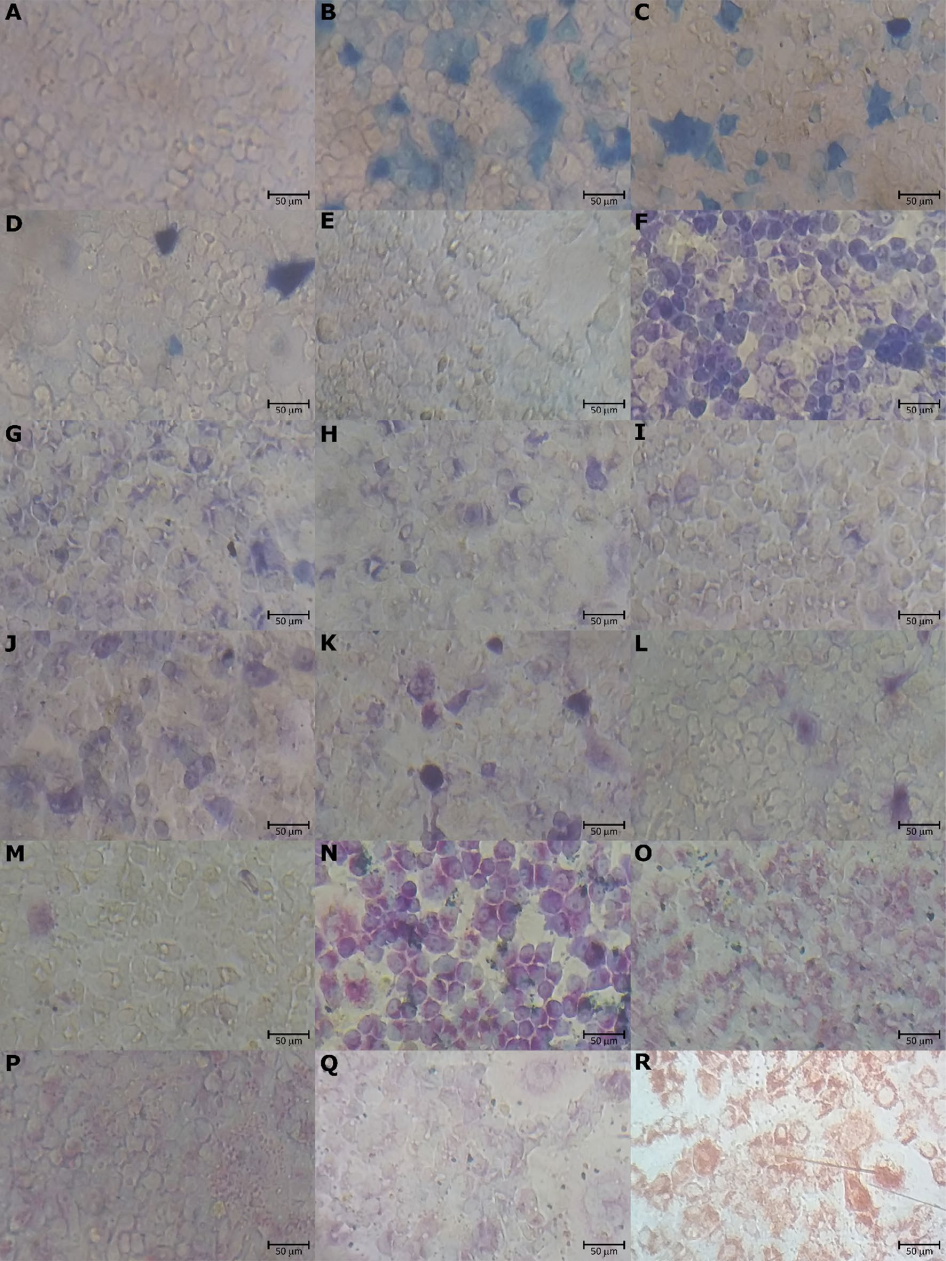
Uptake of phenothiazinium dyes by Vero cells. Cells were incubated with MB, NMB, TBO, and DMMB (100, 50, 25, and 12.5 µM, respectively) for 3 h and the cultures were observed under a light microscope. Cells incubated with RPMI (dye-free medium) or NR (50 µg/ml; 173 µM) were used as negative and positive controls, respectively. (**A**) No compound control. (**B–E**) Cells incubated with MB dilutions. (**F–I**) Cells incubated with NMB dilutions. (**J–M**) Cells incubated with TBO dilutions. (**N–Q**) Cells incubated with DMMB dilutions. (**R**) Cells incubated with NR. Bars = 50 µm.

Cells incubated with phenothiazines at 100 µM displayed no sign of toxicity and were morphologically similar to the control group (Fig. 1A). We observed that uptake of phenothiazinium dyes was diverse for each dye tested. On the one hand, MB and TBO had a heterogeneous accumulation pattern: stained cells presented spots with high dye content and the number of unstained cells was higher (Fig. 1B–E,J–M). On the other hand, cells incubated with NMB and DMMB were stained uniformly and fewer unstained cells were observed (Fig. 1F–I,N–Q). Dilution of all dyes decreased the number of labeled cells (Fig. 1B–Q). The dye NR, which was applied as a positive control for dye uptake, accumulated mostly in the cell cytoplasm (Fig. 1R). Likewise, NMB and DMMB were also found in the cell cytoplasm, even in samples incubated at concentrations below 50 µM (Fig. 1G–I,O–Q). Moreover, TBO has an affinity to large nucleoli in several cells (Fig. 1J–L). Crystals of NR formed during incubation with cells, whereas no dye precipitation was observed for phenothiazinium dyes (Fig. 1B–R).

**Figure 2:**
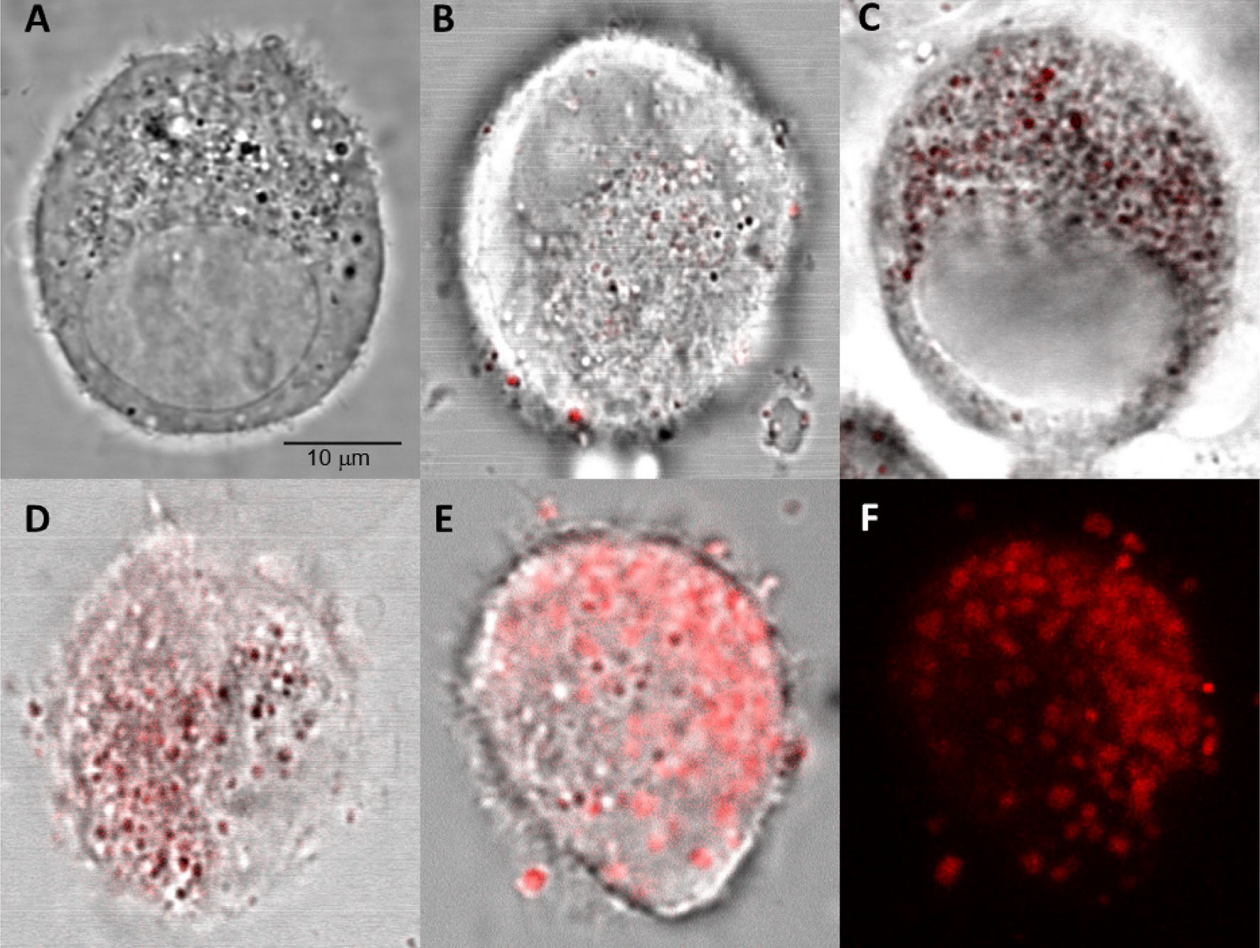
Detection of phenothiazine dyes accumulation in Vero cells by fluorescence. Vero cells were incubated with phenothiazine dyes (10 μM) and analyzed by confocal microscopy. Images were acquired using a 65× objective. The dyes were detected at 543 and 600 nm of excitation and emission wavelengths, respectively. (**A**) Non-treated control. (**B–E**) Cells treated with MB, NMB, TBO and DMMB, respectively. (**F**) Representation of 15 superimposed images of a cell treated with DMMB. The bar indicates 10 μm.

Detection of phenothiazinium dyes by fluorescence showed their accumulation in the cell cytoplasm. The dyes were detected by fluorescence in vesicle-like structures, which were not observed in untreated control (Fig. 2A). Among the dyes, MB showed less accumulation relative to NMB, TBO, and DMMB (Fig. 2B–F).

### Lipophilicity prediction and phenothiazinium dyes localization within cells

The values for log P were calculated either by applying Eq. (2)^24^ or by in silico ChemAxon calculators. Both methods resulted in the same pattern of dye lipophilicity: MB and TBO displayed a more hydrophilic behavior, with negative log P values, whereas NMB and DMMB had positive values, indicating a more lipophilic profile (Tables 1, 2). The use of Eq. (2) resulted in log P values for MB and TBO of − 0.62 and − 1.47, respectively (Table 1). Although in silico calculations presented slightly different numbers, the same overall pattern remained, with log P values of − 0.51 (MB) and − 1.09 (TBO) (Table 2). For DMMB, log P values obtained using Eq. (2) and the ChemAxon calculator were 0.5 and 0.23, respectively (Tables 1, 2). According to in silico estimations, among the phenothiazinium dyes, NMB is the only one that can have a free base form within the physiological pH range, in addition to the protonated molecule (Table 1). Adopting Eq. (2), the free base form of NMB has a log P of 4.84, while the software calculated a log P of 3.55. This happened because the last also considers the contribution of protonated forms to estimate log P. If one takes a closer look at π values of protonated amines^24^, it is easier to understand why they lower log P: the radical ^+^N(CH_3_)_3_ contributes with a π value of − 5.96, for example. When Eq. (2) was applied, the obtained log P for the protonated form of NMB decreased to 0.4 (Table 1). The predicted pK_a_ values of MB (~ 3.14), TBO (~ 3.17), and DMMB (~ 3.86) indicate a higher prevalence of protonated forms in physiological, lysosomal, or mitochondrial pHs (Supplementary Fig. 2). The pK_a_ of NMB (7.03) indicates a coexistence between the protonated and free base forms in acid and alkaline solutions, respectively (Supplementary Fig. 2). The lipophilicity tendencies were maintained for all dyes in physiologic (pH 7.4), lysosomal (pH 4.7) and mitochondrial (pH 8.0) milieus. In all analyzed conditions, the log D (the calculated lipophilicity when standard conditions are not met, in this case, in a specific pH) values were negative for MB and TBO and positive for NMB and DMMB (Table 2).

**Table 1:** Names, chemical structures and estimated log P of phenothiazinium dyes calculated using Eq. (2).

Compound	Structure	Estimated log P
Methylene Blue, MB		− 0.62
Toluidine Blue, TBO		− 1.47
Dimethylmethylene Blue, DMMB		0.5
New Methylene Blue, NMB		0.4
		4.84

**Table 2:** pK_a_, log P, and log D of phenothiazinium dyes calculated in silico with ChemAxon software. The pH of organelles was obtained from Ref.^26^.

	pK_a_	log P	log D (pH 7.4)	log D (lysosomes; pH 4.7)	log D (mitochondria; pH 8.0)
MB	3.14	− 0.51	− 0.62	− 0.63	− 0.62
NMB	7.03	3.55	3.72	1.52	3.84
TBO	3.17	− 1.09	− 1.04	− 1.05	− 1.04
DMMB	3.86	0.23	0.29	0.23	0.29

### Detection of phenothiazinium dyes in the culture supernatant

**Figure 3:**
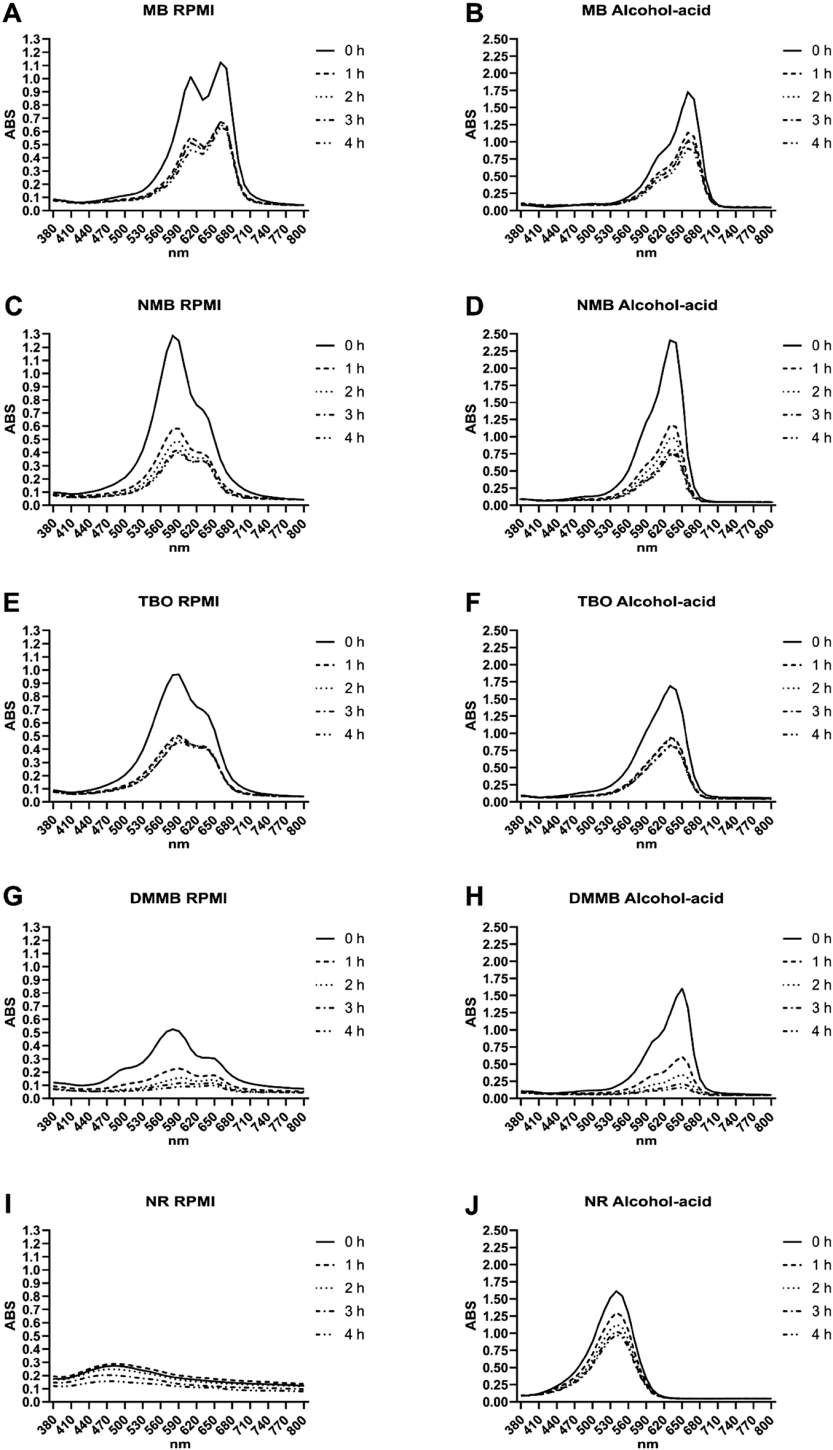
Absorption spectra for the supernatants obtained from Vero cell cultures incubated with phenothiazinium dyes and NR. The dyes MB, NMB, TBO, DMMB (each at 100 µM), and NR (173 µM) were diluted in RPMI and incubated with Vero cells for 4 h. An aliquot of the supernatants (in RPMI or diluted in an alcohol-acid solution) were collected after 0, 1, 2, 3 and 4 h of incubation and visible absorption spectra were obtained. (**A,C,E,G,I**) represent, respectively, the spectra of MB, NMB, TBO, DMMB, and NR in RPMI. (**B,D,F,H,J**) represent the spectra of supernatants diluted in alcohol-acid solution. Figures are representative of three independent experiments.

All phenothiazinium dyes diluted at 100 µM were promptly detected in the visible spectrum (Fig. 3). MB in RPMI presented two maximum absorption peaks (610 and 660 nm), which became a single peak at 660 nm after the addition of an alcohol-acid solution (Fig. 3A,B). Conversely, both NMB and TBO (in RPMI) produced a single peak at 580 nm, which shifted to 630 nm in an alcohol-acid solution (Fig. 3C–E). DMMB also presented a peak shift, but in this case from 570 to 650 nm (Fig. 3G,H). All phenothiazinium dyes were readily absorbed/internalized by the cells, leading to a decreased spectral absorption in the supernatant over time (Fig. 3A–H). Also, the dyes were incorporated by cells within the first hour of incubation, with a lower rate of absorption observed during the second, third, and fourth hours of culture (Fig. 3A–H). The two spectral peaks, characteristic of MB in RPMI, decreased from 1.0–1.1 to 0.5–0.6 after 1 h of incubation. Similarly, when the same supernatant was diluted in an acid-alcohol solution, the peak at 660 nm reduced from 1.7 to 1.2 (Fig. 3A,B). The peaks of detection of NMB, TBO, and DMMB in RPMI decreased from 1.30, 0.95, and 0.50 to 0.55, 0.50, and 0.18, respectively, after 1 h of incubation (Fig. 3C,E,G). A similar pattern was observed in supernatants diluted in an alcohol-acid solution. The peaks of detection of NMB, TBO, and DMMB reduced from 2.45, 1.70, and 1.60 to 1.20, 0.90, and 0.50, respectively (Fig. 3B,D,F,H). In general, after the second, third, and fourth hours of incubation, the absorbance rates for the compounds were lower for MB and TBO compared to NMB and DMMB. The maximum absorption peak for NR in RPMI varied by fewer than 0.2 units (Fig. 3I) whereas the sample diluted in alcohol-acid decreased from 1.6 to 0.9 (Fig. 3J).

### Detection of intracellular phenothiazinium dyes in cell monolayers

**Figure 4:**
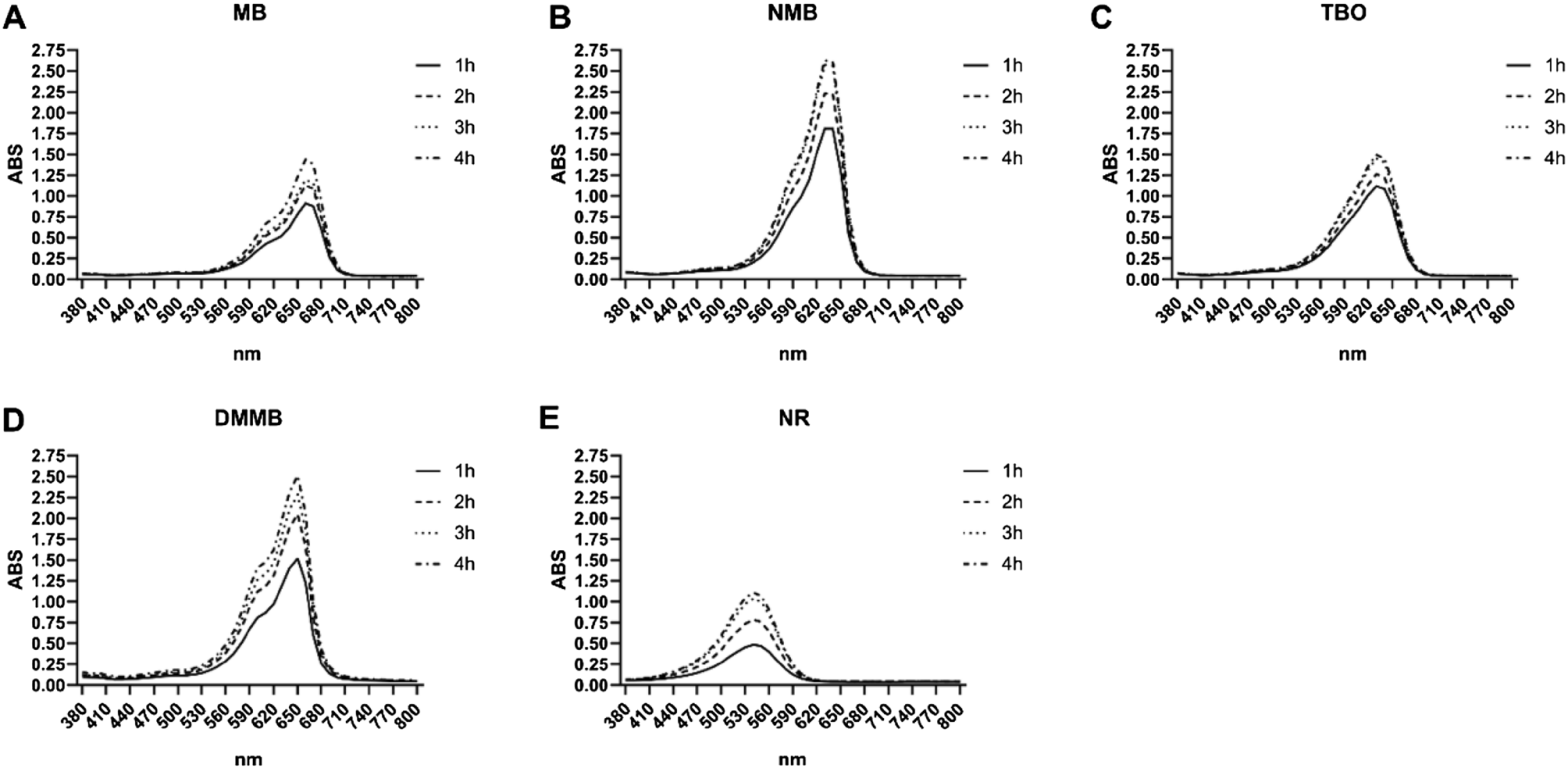
Absorption spectra for intracellular phenothiazinium dyes and NR of Vero cell cultures. Vero cells were incubated with (**A**) MB, (**B**) NMB, (**C**) TBO, and (**D**) DMMB in RPMI (100 µM) for 4 h. As an uptake control, (**E**) NR (173 µM) was incubated under the same conditions. After 1, 2, 3, and 4 h of incubation, cells were fixed and their content was extracted with a NR extraction solution. The visible spectra of the extracted cell contents were generated in an ELISA reader. (**A–E**) Represent the spectra of MB, NMB, TBO, DMMB and NR, respectively. Figures are representative of three independent experiments.

Phenothiazinium dyes that accumulated in cell monolayers were extracted with NR extraction solution after fixation. During incubation, intracellular dye concentrations increased, mainly after the first and second hours of incubation (Fig. 4).

**Figure 5:**
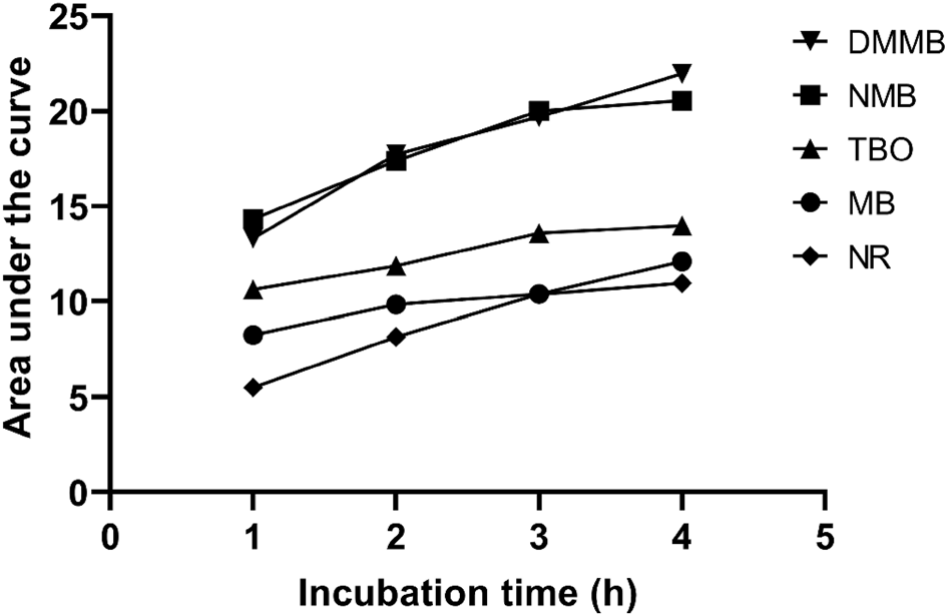
Area under the curve of dye spectra obtained for Vero cells. Vero cells were incubated with 100 µM of MB, NMB, TBO, and DMMB for up to 4 h. After 1, 2, 3, and 4 h of incubation, cell contents were extracted with an NR extraction solution. The visible spectra of cell contents were generated in an ELISA reader and area under the curve was calculated. NR (50 µg/ml; 173 µM) was used as a control for dye uptake and submitted to the same procedure.

Similar to the observed for the supernatant analyses, the phenothiazinium dyes presented higher levels of internalization compared to NR. Uptake of NMB and DMMB was higher compared to MB or TBO. To quantify this phenomenon, we calculated the area under the absorbance curve for each dye used. The areas under the curve for NMB were 14.3, 17.3, 19.9 and 20.5 after 1, 2, 3 and 4 h of incubation, whereas those for MB and TBO were 8.25/10.63, 9.84/11.87, 10.38/13.58 and 12.10/13.97, respectively (Fig. 5). For DMMB, the area under the curve was 13.3 (1 h), 17.7 (2 h), 19.6 (3 h) and 21.9 (4 h). Areas under the curve for NR were between 5.5 and 10.96 (after 1 and 4 h of incubation, respectively), with these values being lower than those observed for all phenothiazines (Fig. 5).

### Sensitivity of phenothiazinium dyes detection in cells

**Figure 6:**
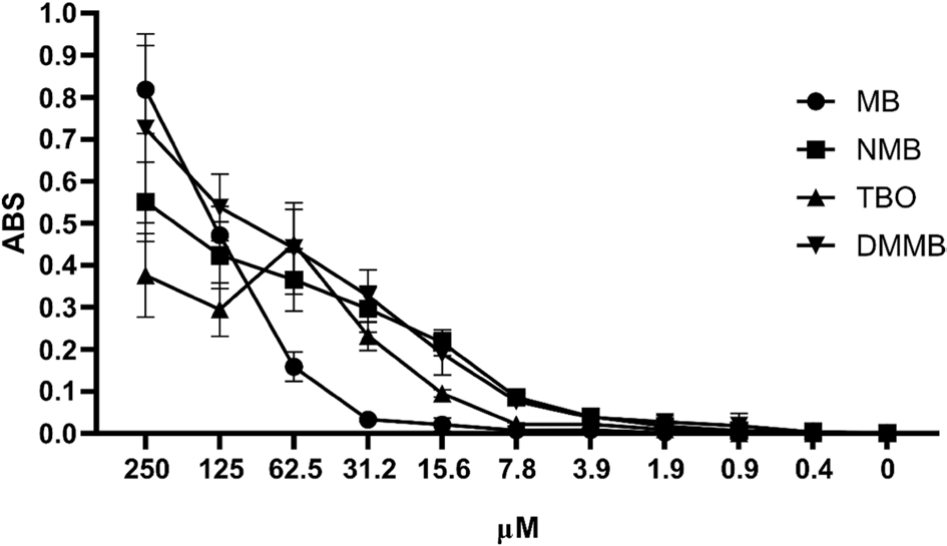
Limits of phenothiazinium dyes detection in Vero cells. Serial dilutions of MB, NMB, TBO, and DMMB (starting at 250 µM) were incubated with a Vero cell monolayer (in 96-well plates) for 3 h at 37 °C and 5% CO_2_. After the incubation, the cells were fixed, and the dyes extracted. The accumulation of dyes was measured in an ELISA reader at 660 nm (MB), 630 nm (NMB and TBO) or 650 nm (DMMB).

The minimum limit for detection of phenothiazinium dyes varied according to the dye: MB, NMB, TBO, and DMMB were detected in concentrations above 31.2, 7.8, 15.6, and 7.8 µM, respectively (Fig. 6). Except for TBO, correlation between absorbance and dye concentration was good for all phenothiazines (Fig. 6). For TBO, the absorbance/concentration correlation was observed only until 62 µM (Fig. 6). A low difference in detection among phenothiazines was observed at 125 µM, a concentration for which absorbance varied from 0.29 (TBO) to 0.53 (DMMB). At 100 µM (calculated using the linear regression equation), the difference in absorbance among dyes was lower compared to 125 µM, corresponding to 0.34, 0.39, 0.35, and 0.50 for MB, NMB, TBO, and DMMB, respectively (Fig. 6). Therefore, a concentration of 100 µM was selected for an adequate comparison among the dyes in the experiments that followed (i.e., linearity and cytotoxicity assays).

### Linearity of phenothiazinium dyes in Vero cells

**Figure 7:**
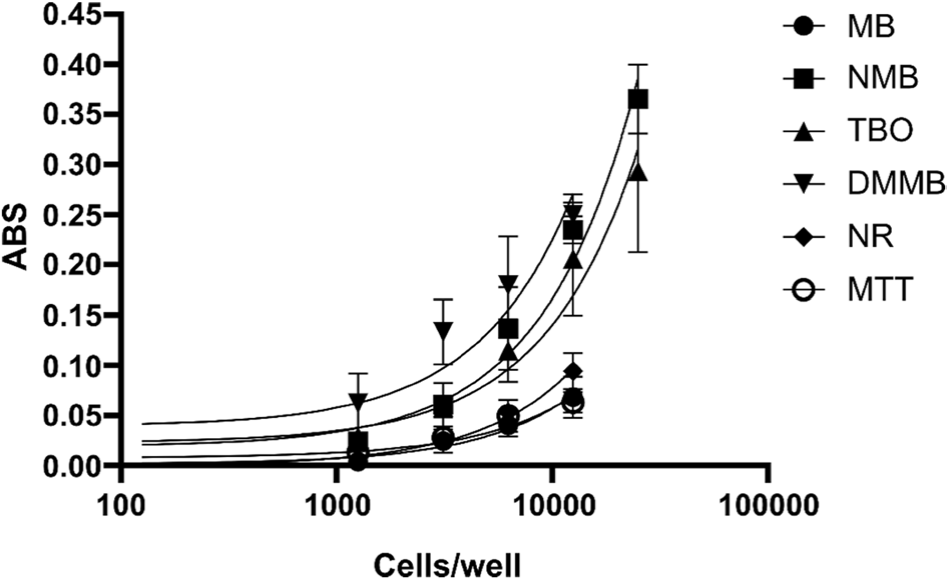
Linearity of phenothiazinium dyes in different cell densities. Vero cells were treated with trypsin, counted, diluted (2.5 × 10^4^, 1.2 × 10^4^, 6.2 × 10^3^, 3.1 × 10^3^, and 1.5 × 10^3^ cells/well) and distributed in 96-well plates. After 24 h of cultivation, the cells were incubated with 100 µM of MB, NMB, TBO, and DMMB, 50 µg/ml of NR or 0.5 mg/ml of MTT, for 3 h at 37 °C and 5% CO_2_ and the markers extracted. The plates were read in an ELISA reader for detection of MB (660 nm), NMB (630 nm), TBO (630 nm), DMMB (650 nm), NR (540 nm) or MTT (570 nm) and the absorbance values were plotted against cell density. The curves were analyzed by linear regression and the correlation coefficient (r^2^) was calculated.

The accumulation of phenothiazinium dyes in Vero cells was proportional to cell numbers within each well. All dyes were detected at cell densities above 10^3^ cells/well, similar to what was observed for MTT and NR (Fig. 7). All compounds had consistent correlation between cell number and absorbance, reaching saturation at densities above 1 × 10^4^ cells/well. Among the compounds, NMB and TBO showed dye saturation at 2.5 × 10^4^ cells/well whereas MB, DMMB, MTT, and NR reached a peak of detection at 1.25 × 10^4^ cells/well (Fig. 7). The linear regression analyses of curves generated from absorbance × number of cells resulted in r^2^ values of 0.83 (MB), 0.94 (NMB), 0.84 (TBO), 0.81 (DMMB), 0.94 (NR), and 0.80 (MTT).

### Phenothiazinium dyes as indicators of cytotoxicity in cells

**Figure 8:**
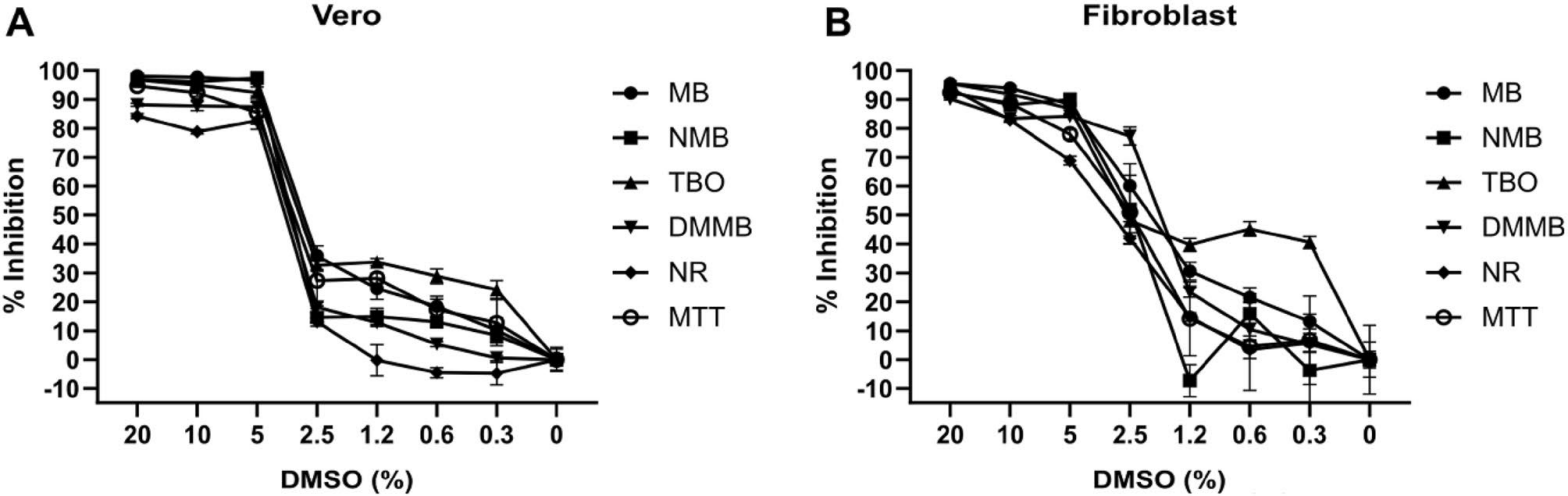
Inhibition curves of Vero and fibroblasts incubated with DMSO and labeled with phenothiazinium dyes. Vero (**A**) and fibroblasts (**B**) were treated with the indicated series of DMSO dilutions (starting with 20% v/v) for 72 h. After the treatment, cell cultures were incubated with phenothiazinium dyes (100 µM), NR (50 µg/ml; 173 µM) or MTT (0.5 mg/ml; 1.2 µM) for 3 h. Phenothiazinium dyes and NR were extracted using the NR extraction solution and formazan (MTT) was diluted with DMSO. The absorbances at 540, and 570 nm were measured in an ELISA reader, according to the cytotoxicity indicator (MB, NMB, TBO, DMMB, NR, and MTT, respectively). Absorbance values were used to calculate percent inhibition. Error bars are standard deviation from three independent experiments.

Phenothiazinium dyes were tested as cytotoxicity markers and their performance was measured against that of NR and MTT. Initially, Vero cells and fibroblasts were incubated with different concentrations of DMSO and cell viability was assessed. Vero cells and fibroblasts were inhibited (> 70%) by DMSO in concentrations above 5%, with decreased inhibition for more diluted samples (< 5%). Nonetheless, DMSO maintained a basal cell inhibition at concentrations of 2.5% and lower, especially as measured by TBO (Fig. 8).

**Figure 9:**
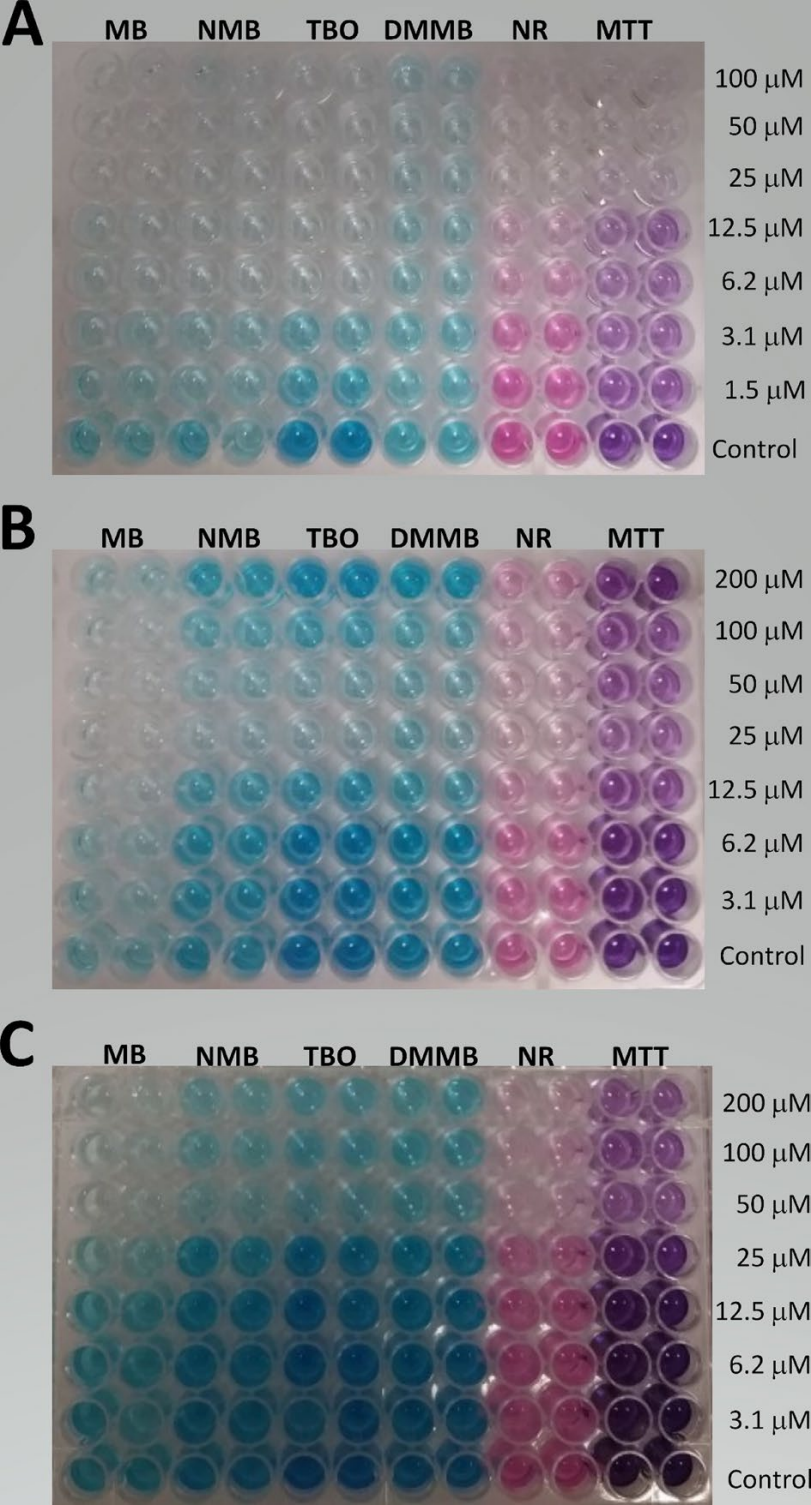
Cell cultures treated with cisplatin and stained with phenothiazinium dyes, NR, and MTT. The MDA-MB-231 (**A**), MCF 10 A (**B**), and LLC-MK2 (**C**) cell lines were incubated in 96-well plates and treated with cisplatin dilutions or drug-free controls for 72 h. After the treatment, cells were washed and incubated with MB, NMB, TBO, DMMB (100 µM), NR (50 µg/ml; 173 µM) or MTT (0.5 mg/ml; 1.2 µM) for 3 h. The phenothiazinium dyes and NR in cultures were solubilized with an acid-alcohol solution after fixation, whereas MTT was extracted with DMSO. Images are representative of three independent experiments.

Furthermore, phenothiazinium dyes allowed the evaluation of cisplatin cytotoxicity in both resistant and susceptible cell lines. After incubation with cisplatin, staining intensity of the toxicity markers showed a dose–response profile. Similar intensity patterns were observed between phenothiazinium (especially NMB, TBO, and DMMB) and NR. Monolayers of MDA-MB-231 cells (Fig. 9A) showed less intense staining than MCF 10A (Fig. 9B) and LLC-MK2 (Fig. 9C) cells.

**Figure 10:**
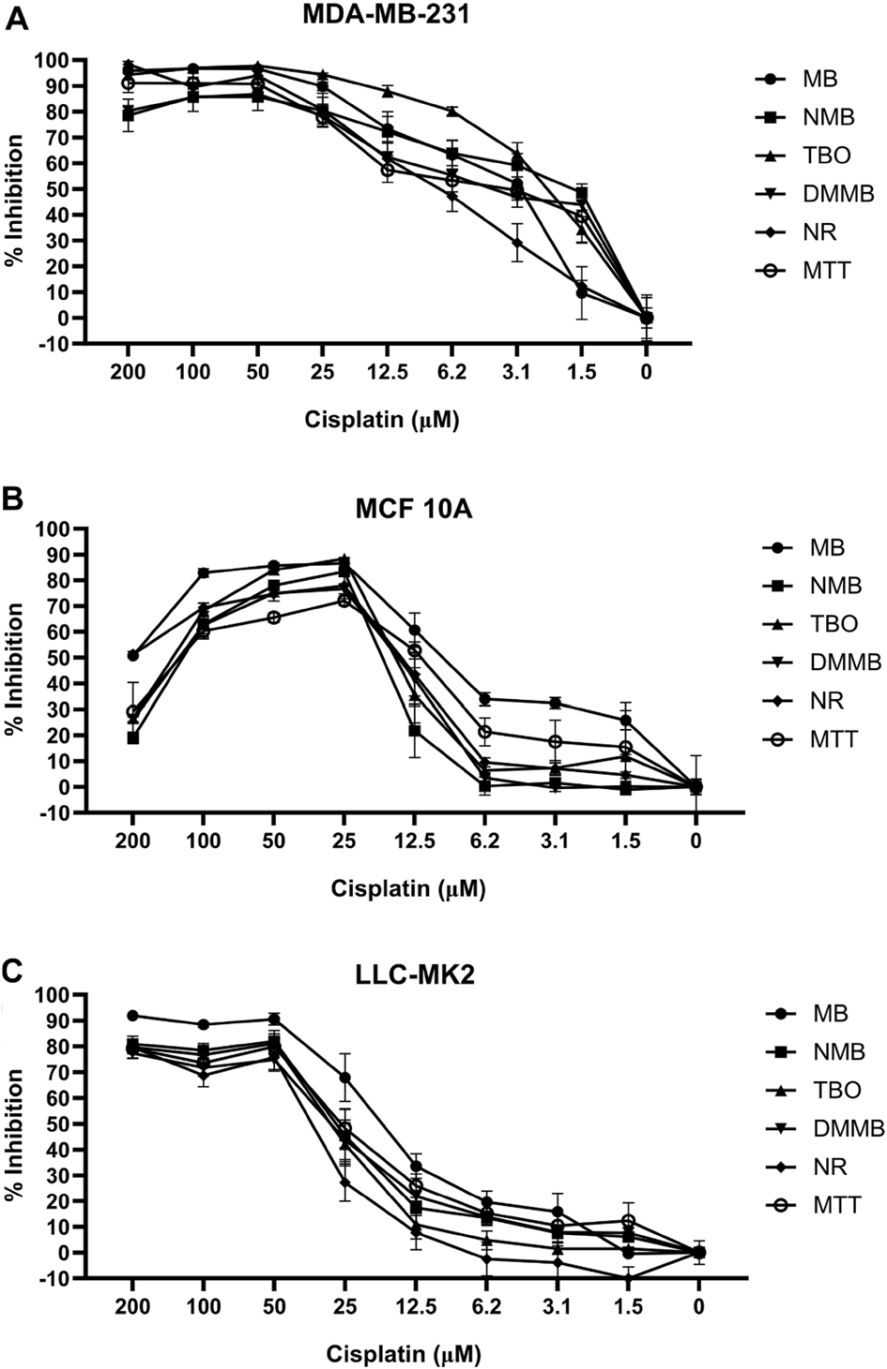
Dose-inhibition curves for cells treated with cisplatin and incubated with phenothiazinium dyes, NR, and MTT. MDA-MB-231 (**A**), MCF 10A (**B**), and LLC-MK2 (**C**) cells were treated with the indicated concentrations of cisplatin for 72 h, followed by labeling with phenothiazinium dyes. MB, NMB, TBO, and DMMB (100 µM) were incubated with the cells for 3 h and the dyes were extracted with an acid-alcohol solution. In parallel, NR and MTT were applied as cell viability controls. Absorbances were measured in an ELISA reader at wavelengths 540, and 570 nm for MB, NMB, TBO, DMMB, NR, and MTT, respectively. Percent inhibition was calculated using the absorbance values of treated samples relative to values for non-treated controls. Error bars are standard deviation from three independent experiments.

Quantification of the apparent staining changes resulted in the same pattern (Fig. 10). At concentrations above 12.5 µM, MDA-MB-231 cells were inhibited by more than 50% (Fig. 10A). On the other hand, MCF 10A and LLC-MK2 cells were resistant to cisplatin at concentrations below 25 µM and 50 µM, respectively (Fig. 10B,C). As observed on 96-well plates (Fig. 9B), the MCF 10A cell line had higher tolerance to cisplatin at 200 µM compared to 100 µM (Fig. 10B). For 100 µM and above, the curve fit of MCF 10A cells followed a dose–response pattern, similar to the observed for LLC-MK2 cultures (Fig. 10B,C). All inhibition curves from cell lines incubated with dyes allowed the calculation of IC_50_ values.

**Figure 11:**
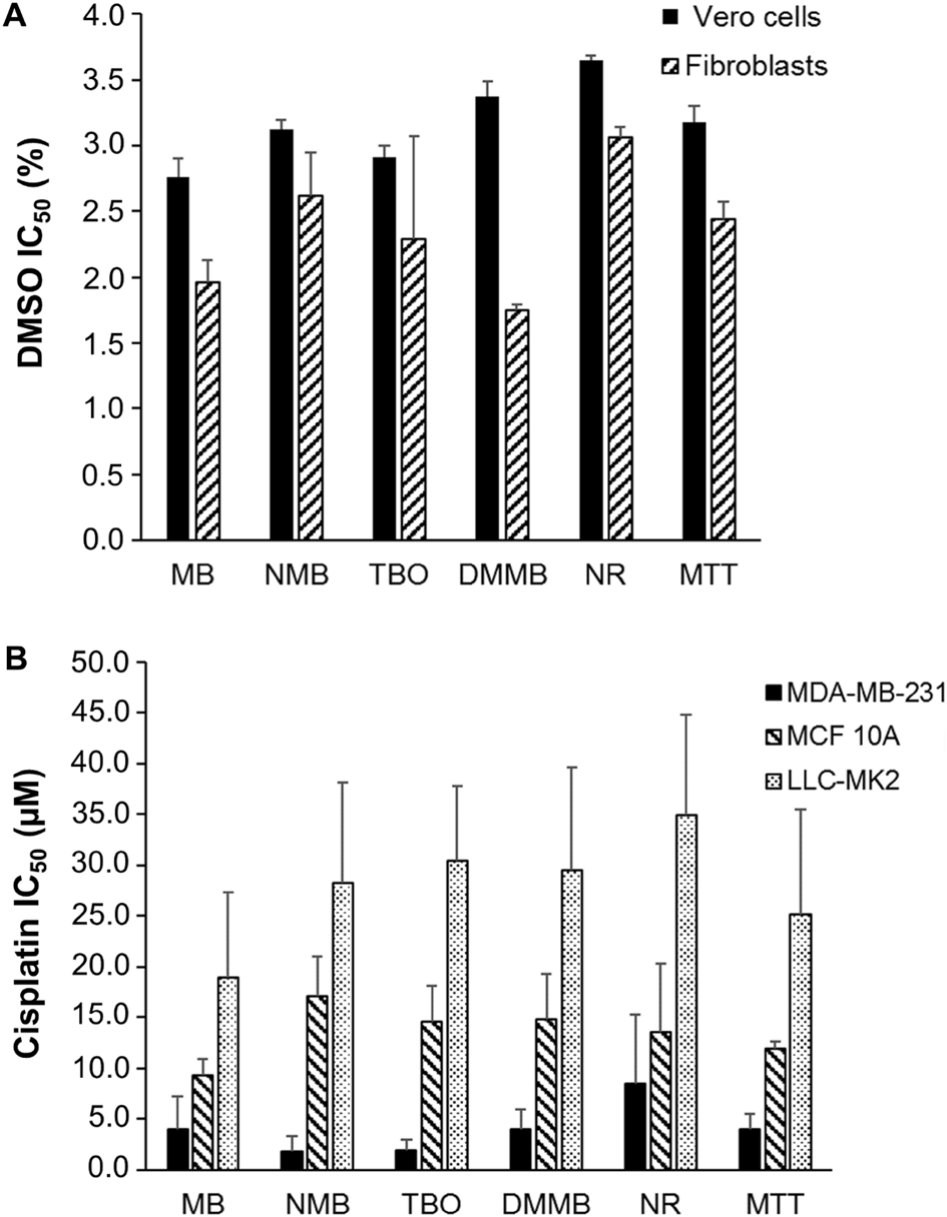
Half maximum inhibitory concentration (IC_50_) for cell lines treated with DMSO or cisplatin and incubated with phenothiazinium dyes, NR, and MTT. Vero cells and fibroblasts were treated with (**A**) DMSO (diluted from 20%) or (**B**) cisplatin for 72 h. After the treatment, cultures were incubated (for 3 h) with phenothiazinium dyes (100 µM), NR (50 µg/ml; 173 µM) or MTT (0.5 mg/ml; 1.2 µM) to allow dye uptake by viable cells. The plates were read in an ELISA reader and absorbance values were applied to calculate percent inhibition relative to non-treated controls. The IC_50_ values were calculated using the Compusyn software. Error bars are standard deviation from three independent experiments.

The IC_50_ values followed similar patterns for the phenothiazinium dyes, NR, and MTT. Vero cells and fibroblasts had equal susceptibility to DMSO, with IC_50_ values between 3.65 (for NR) and 2.76 µM (for MB) (Fig. 11A). For cisplatin, the treated samples presented IC_50_ values in accordance with susceptibility of the cell line. MDA-MB-231 showed the highest susceptibility to cisplatin, with IC_50_ between 1.83 (NMB) and 8.44 µM (NR). For MCF 10A cells, which showed an intermediate resistance to cisplatin, the IC_50_ values were 9.26, 11.88, 13.47, 14.57, 14.77, and 17.14 µM for MB, MTT, NR, TBO, DMMB, and NMB, respectively. Our results indicate a higher resistance of LLC-MK2 cells to cisplatin compared to MDA-MB-231 and MCF 10A. The IC_50_ values for LLC-MK2 were above 18.98 µM, which was observed for samples incubated with MB. For NMB, TBO, DMMB, NR, and MTT, the IC_50_ values were 28.23, 30.41, 29.46, 34.94, and 25.19 µM, respectively (Fig. 11B). There was no statistically significance difference between the performance of phenothiazines and NR or MTT for the quantification of cytotoxicity in the presence of DMSO and cisplatin.

## Discussion

The uptake of phenothiazinium dyes by viable cells revealed their potential as markers of cytotoxicity. Moreover, these dyes presented high stability, low toxicity, low cost, and simple and safe handling. Such factors, together with good assay reproducibility, are crucial factors for the development of methods to assess the in vitro cytotoxicity of compounds^32^. Each phenothiazinium dye showed a different absorption pattern by cells, especially when MB and TBO were compared to NMB and DMMB. However, all dyes had tropism for the cell cytoplasm, especially when diluted. Indeed, MB has affinity for lysosomes as previously reported for both cancerous and normal breast tissue cell lines^33^. In general, phenothiazinium dyes incubated with cells are absorbed within the first hour of incubation, similarly to what is observed for NR. This fast absorption rate is an important factor for cell labeling because NR is toxic to cells^34^. Phenothiazinium dyes are also toxic to Vero cells in longer regimens of incubation. After 72 h of incubation, MB had low cytotoxicity at 100 µM (IC_50_ > 62.5 µM), whereas NMB and TBO were toxic at lower concentrations (~ 20–25 µM). For DMMB, the toxic effects in Vero cells were observed at concentrations below 10 µM^30^. Thus, we tested the phenothiazinium dyes as cytotoxicity markers in short incubation periods (< 4 h), similar to the ones applied to NR or MTT assays.

The phenothiazinium dyes presented several advantages over NR, such as higher solubility and increased stability in culture media. NR usually precipitates in culture, requiring filtration and/or overnight incubation in a refrigerator before use^34,35^. Other strategies applied to decrease NR precipitation are filtration of the medium at the time of use or pre-filtering of the dye stock, both of which usually reduce the discrepancy between assays^35–37^. Therefore, the use of NR as a cytotoxicity marker requires additional preparation steps compared to phenothiazinium dyes. Moreover, the precipitation of NR is increased in the presence of salts and/or fetal bovine serum: stock solutions of NR revealed a higher number of crystals in PBS compared to distilled water^38^. Conversely, no precipitation or crystallization were observed for phenothiazinium dyes at 100 µM. Additionally, the stock solutions of phenothiazinium dyes are stable in water for long periods (> 6 months) at 5 mg/ml.

The main difference between currently used cytotoxicity assays employing NR and MB is in the cell model of stain. NR is applied to living cells, which accumulate the dye in lysosomes^11^. In contrast, MB is used on formalin-fixed cells^39,40^. The method presented here is novel in that it uses viable cells, similar to NR. Indeed, the phenothiazinium dyes accumulated in vesicle-like structures in Vero cells. The MB accumulation is also observed for MB and TBO in HeLa cells^41^. Moreover, MB is reported to accumulate in the lysosomes of mammary cells^33^.

Furthermore, our assay employs smaller amounts of phenothiazinium dyes compared to currently used methods applied to fixed cells. After fixation, MB labeling usually uses 5 to 10 g of dye per liter of staining solution^12,40^. Conversely, our study uses 52 mg/l of dye, similar to the concentration employed for NR (50 mg/l)^29,42^. Therefore, our results indicate a novel application of phenothiazinium dyes as viability markers.

In addition, we observed differences in absorption among the dyes: uptake by cells was more intense for NMB and DMMB, both of which equally stained the cultures. Conversely, MB and TBO presented a slower rate of dye incorporation compared to NMB and DMMB, producing an irregular pattern of dye accumulation. These differences among phenothiazinium dyes are a direct consequence of their chemical structures. Some parameters related to the molecular structure, such as log P and pK_a_ can indicate the pattern of uptake and accumulation in cell compartments. We used two strategies to predict the log P values (manually calculated through Eq. (2) and in silico using ChemAxon calculators) with similar results. In general, MB and TBO showed a more hydrophilic profile (log P < 1), whereas NMB and DMMB had a more lipophilic nature (log P > 1)^43^. Thus, the positive log P values corroborate the faster uptake rate of NMB and DMMB when compared to MB and TBO. Moreover, MB and TBO are protonated at pH 7.4, resulting in a slower cell uptake relative to NR and NMB. In this sense, the MB and TBO accumulation in lysosomes is probably dependent on the extent of cell endocytosis. Indeed, the use of membrane carriers (i.e. liposomes or nanoparticles) has improved MB uptake by cells^44,45^ through endocytosis pathways. For example, MB combined with citrate-coated maghemite nanoparticles (MAGCIT–MB) was internalized in breast and ovarian cell lines by the clathrin endocytosis pathway^46^. Once into the cells, hydrophilic dyes with ionizable amines (TBO) probably accumulate in lysosomes by ion trapping, similarly to the observed for NR. In this case, the cationic form of NR (pK_a_ of 6.9) is membrane impermeant with a log P of − 1. However, under physiologic conditions, half of the NR molecules are in a free base state (log P = 1.9), which passively enter the cells. In lysosomes (pH ~ 4.5), the cationic form of NR is predominant, resulting in accumulation within these organelles via ion-trapping^21^. Our results indicated that the free base form of NMB is predominant in alkaline media (pH > 7.0). In mitochondria (pH ~ 8.0), approximately 89.7% of NMB corresponds to the free base form, resulting in higher lipophilicity values (log P 3.84) compared to MB and TBO. Indeed, the log P values of cationic probes between 0 and 5 indicate an affinity to mitochondria, as reported by Refs.^22,47^. Besides the cell uptake rates, NMB and DMMB also have different patterns of cell accumulation compared to MB and TBO. Despite current knowledge and advances, further assays may contribute to the comprehension of phenothiazinium dye uptake and distribution into cells. For example, co-localization studies with well-established organelle markers (LysoTracker™ and MitoTracker™) may better indicate the localization of MB, NMB, TBO and DMMB in cells.

These differences among phenothiazinium dyes are also observed in other models. For example, DMMB and NMB exhibited higher toxicity in LLC-MK2 and Vero cells, in addition to parasiticidal activity against *Neospora caninum* and *Trypanosoma cruzi*, compared to MB and TBO^30,48^. Thus, further development of phenothiazinium analogues by manipulation of dye structure will potentially result in cytotoxicity markers with higher labeling capacity.

The differences among phenothiazinium dyes were also observed in sensitivity and linearity assays. In general, dyes incubated with Vero cells showed a good correlation between absorbance and dye concentration or cell density. NMB and TBO showed linearity with a higher number of cells (2.5 × 10^4^ cells/well) compared to MB, DMMB, NR and MTT (1.2 × 10^4^ cells/well). Probably, NMB and TBO have a lower saturation capacity compared to the other markers. However, new linearity assays using different cell lineages, dye concentrations, counters (i.e., flow cytometer) and markers (i.e. resazurin, LDH assay) may elucidate the differences in saturation among dyes. NMB and DMMB were detected at lower concentrations (> 7.8 µM) compared to MB and TBO (> 15.6 µM and > 31.2 µM, respectively). Although NMB and DMMB are the most sensitive, MB produced the highest absorbance value at 250 µM. Indeed, MB had a lower rate of accumulation, reaching saturation only at higher concentrations. However, in concentrations above 100 µM, all dyes have displayed toxic effects in cells (cell detachment, data not shown), indicating that the use of dyes at concentrations higher than 100 µM should be assessed in further assays. As such, we selected the concentration of 100 µM for cytotoxic assays. At this concentration, all dyes had low and non-significant affinity to the plastic material of plates (data not shown).

Furthermore, the dyes displayed linearity between absorbance and cell density, similar to the observed for MTT or NR. The values of r^2^ for all phenothiazinium dyes tested were above 0.8, which are in accordance with previous studies using MTT or NR^49–51^. A linear correlation between cell density and phenothiazinium dyes, MTT or NR absorbance values was achieved for cell densities ranging from 1000 to 25,000 cells/well. At 12,500 cells/well (NR and MTT) or 25,000 cells/well (NMB and TBO) the curves were out of the linear range, which was also reported in studies using different cell lineages^52–54^. In general, our results indicate a correlation between dye accumulation and cell density, similar to the observed for assays based on formalin fixed assays^39,40^. However, the mechanism of dye accumulation is different in live and fixed cells. In formalin-fixed cells, MB has an affinity to negatively charged structures of the cell (i.e., DNA)^55^. In our model, using non-fixed (live) cultures, the dyes (mainly NMB and DMMB) have an affinity to vesicles like-structures similar to the observed for NR. Despite the correlation between dye accumulation and cell density, new assays concerning MB incorporation and cell viability are necessary. For example, the comparison between phenothiazinium dyes and vital dyes (propidium iodine or trypan blue) may indicate a future application of MB and analogues as cell viability markers.

To evaluate to which extent phenothiazines could be compared to NR and MTT as cytotoxicity markers, we used DMSO and cisplatin, which have been extensively used in cytotoxicity assays. DMSO is widely applied with low toxicity for solubilization of non-polar compounds for in vitro assays^56,57^. DMSO usually presents similar toxicity for diverse cell lines, mainly when applied at concentrations above 2% (v/v)^58,59^. Likewise, in our study, DMSO inhibited Vero cells and fibroblasts between 1.75 and 3.65%. The assays employing DMSO indicate the applicability of phenothiazines as indicators of cytotoxicity, showing similar patterns compared to MTT or NR. The similarity among phenothiazinium dyes, MTT, and NR was confirmed via assays with cisplatin applied to both susceptible and resistant cell lines. Cisplatin is an antitumoral drug used for the treatment of bladder, head and neck, lung, ovarian, and testicular neoplasms^60^. Several studies have reported the use of cisplatin for in vitro assays in several cell lines^61,62^. Our results indicated three patterns of susceptibility to cisplatin, according to cell line: MCF 10A is a line applied as resistance control (for mammary cancer) to cisplatin, with IC_50_ concentrations between 5 and 26 µM^63,64^. In this study, MCF 10A was applied as non-cancerous control for the MDA-MB-231 cells, which is a breast adenocarcinoma lineage. Cisplatin was shown to inhibit 50% of the growth of MDA-MB-231 at 6 µM^63^, which is a similar value to the ones we observed for MTT, NR or the phenothiazinium dyes. To reinforce the reproducibility of our results, we tested the LLC-MK2 cell lineage, which is widely used in cytotoxicity assays^65,66^, including phenothiazinium dyes^48^. LLC-MK2 is a non-cancer cell lineage of renal tissue origin, with high robustness for cultivation^67–69^. The resistance and robustness of LLC-MK2 were confirmed by our results, which indicated the highest resistance to cisplatin compared to MCF 10A and MDA-MB-231 lines. Therefore, our study indicates a common pattern of susceptibility to cisplatin in different cell lineages, which validates the use of phenothiazinium dyes as cytotoxicity markers.

We have tested the cytotoxicity of cisplatin or DMSO in 72-h incubation regimens, which are applied in several drug screening strategies such as viral, parasitic and tumor models^70–72^. Cisplatin and DMSO are very different agents with clearly distinct modes-of-action. Nonetheless, our method was capable of evaluating their intrinsic toxicity to same extent as the classical assays. In this sense, our results open the perspective of developing novel assays for toxicity monitoring with varied molecules, from antimicrobials to antitumoral agents. In conclusion, this work presents the potential of phenothiazinium dyes as markers for cytotoxicity assays in living cells via a simple, easy-to-use, and cost-effective method that can be applied to the cytotoxicity assessment of candidate drugs in other models.

## Supplementary Information

Supplementary Information 1.

Supplementary Information 2.

Supplementary Figure 1.

Supplementary Figure 2.

## Data Availability

All data generated during this study are included in this published article and its Supplementary Information files.
