# Supplementary material for: The potential of phenothiazinium dyes as cytotoxicity markers in cisplatin-treated cells
Source: Sci Rep. 2023 Jun 23;13:10203. doi: 10.1038/s41598-023-36721-0 (PMC10290130; doi:10.1038/s41598-023-36721-0)
Supplement: Supplementary file 1 — Supplementary Information 1. [file 41598_2023_36721_MOESM1_ESM.docx]

Protocol

Use of phenothiazinium dyes as cytotoxicity markers in *in vitro* cultures

CELLS AND CHEMICALS:

Cells and Culture media:

- Cells: Use adherent cell lineages, cultured in 96-well plates until confluence (> 90%).

- Culture media: Use routine media (RPMI, DMEM) supplemented with 5-10% fetal bovine serum (FBS).

Chemicals:

- Phenothiazinium dyes: Methylene blue (MB, Sigma-Aldrich catalogue number: 1428008), New Methylene Blue (NMB, Sigma catalogue number: R313718), Toluidine blue O (TBO, Sigma-Aldrich catalogue number: T3260) and 1,9 dimethyl-ethylene blue (DMMB, Sigma-Aldrich catalogue number: 341088).

- Phenothiazinium dye stock solution: dilute 5 mg of each phenothiazinium dye in 1 mL of deionized water. The stock solution is stable at – 20°C for one year.

- Phosphate-buffered saline buffer: Dilute 8 g of sodium chloride (NaCl), 0.2 g of potassium chloride (KCl), 1.44 g of sodium phosphate dibasic (Na_2_HPO_4_) and 0.245 g of potassium phosphate monobasic (KH_2_PO_4_) in 800 mL of deionized water. Adjust the pH to ~ 7.4. Add deionized water to a volume of 1 L.

- Fixing solution: Dilute 0.5 g of CaCl_2_ (final concentration 1% w/v) and 0.7 mL of 36% formaldehyde (final concentration 0.5%) in 45 mL of deionized water. Adjust the volume to 50 mL with deionized water.

- NR extraction solution: Dilute 1 mL of acid acetic (glacial) in 45 mL deionized water. Adjust the volume to 50 mL with deionized water. Add 50 mL of absolute ethanol. Final concentration: 1% v/v acetic acid in 50% v/v ethanol.

PROCEDURE:

1 – Use adherent and confluent (> 90% of confluence) cell lineages, cultured in 96-well plates;

2 – Incubate the cells with test compound dilutions (in routine cell culture medium) for 72 h at 37 °C and 5% CO_2_;

3 – After treatment, discard the medium (by dumping) and carefully rinse the cells in 200 μl pre-warmed PBS.

4 – Remove the rinsing solution by dumping and add 200 μl of 100 μM phenothiazinium dyes (MB, NMB, TBO or DMMB) diluted in routine culture medium (pre warmed to 37 °C). Incubate for 3 h at 37 °C and 5% CO_2_.

5 – Discard the medium and quickly wash the cells with 100 μl of fixing solution.

6 – After discarding the fixing solution, add 100 μl of NR extraction solution. Extract the dyes in a plate shaker (or orbital mixer) for 15-30 minutes until complete dilution of the dye.

7 – Measure absorbance in an ELISA reader at 660 nm, 630 nm, 630 nm and 650 nm for MB, NMB, TBO and DMMB, respectively.
