# Supplementary material for: The potential of phenothiazinium dyes as cytotoxicity markers in cisplatin-treated cells
Source: Sci Rep. 2023 Jun 23;13:10203. doi: 10.1038/s41598-023-36721-0 (PMC10290130; doi:10.1038/s41598-023-36721-0)
Supplement: Supplementary file 2 — Supplementary Information 2. [file 41598_2023_36721_MOESM2_ESM.zip › Raw data/Linearity/Supplementary material.docx]

**Supplementary material**


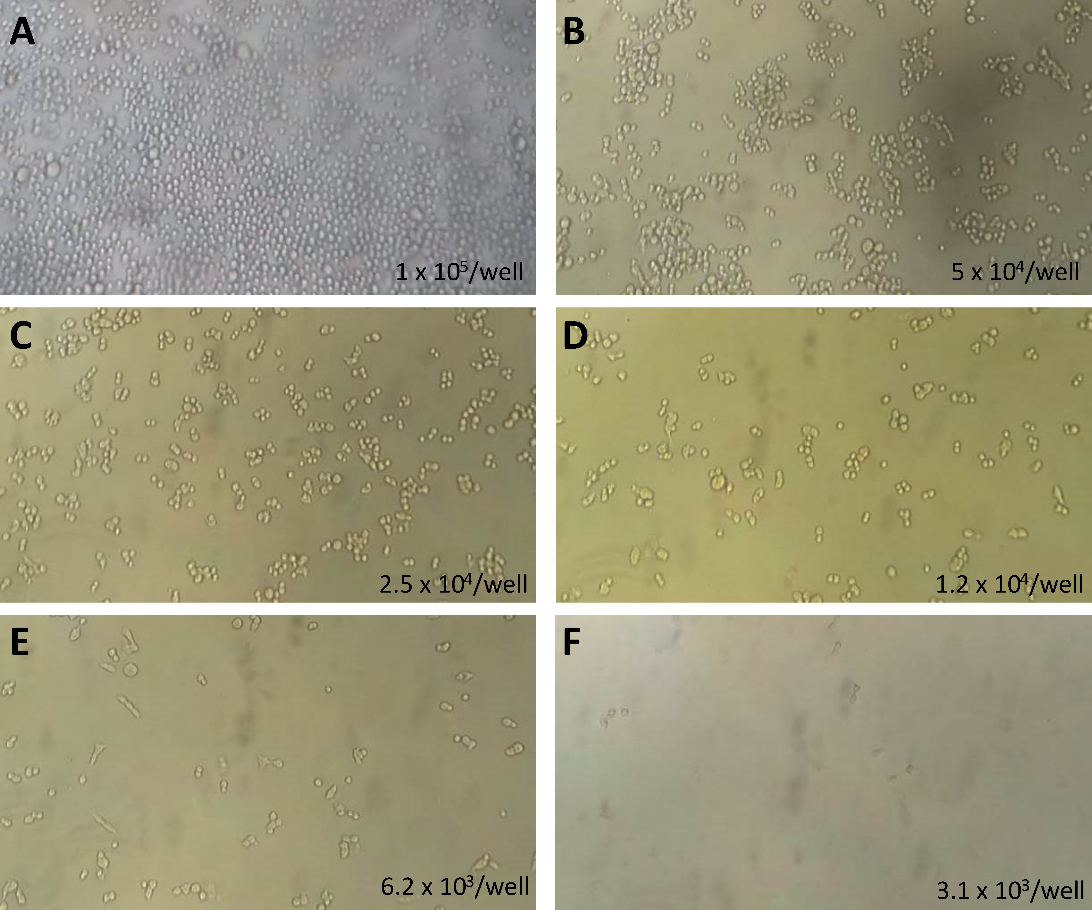


**Supplementary figure 1 – Dilutions of Vero cells suspensions.** Vero cells were treated with trypsin and distributed in 96 well-plates. The cells were diluted starting from 1 × 10^5^/well (100 μl) and incubated for 24 h, 37°C, 5% CO_2_. After incubation, the cells were incubated with MB (100 μM), NMB (100 μM), TBO (100 μM), DMMB (100 μM), NR (50 μg/ml) or MTT (0.5 mg/ml) for 3 h, 37°C, 5% CO_2_ and applied for the evaluation of the dye-cell linearity. A, B, C, D, E, F represents the cell suspensions diluted to 1 × 10^5^, 5 × 10^4^, 2.5 × 10^4^, 1.2 × 10^4^, 6.2 × 10^3^ and 3.1 × 10^3^ cells/well, respectively.
