# Supplementary figures and images for: The potential of phenothiazinium dyes as cytotoxicity markers in cisplatin-treated cells
Source: Sci Rep. 2023 Jun 23;13:10203. doi: 10.1038/s41598-023-36721-0 (PMC10290130; doi:10.1038/s41598-023-36721-0)

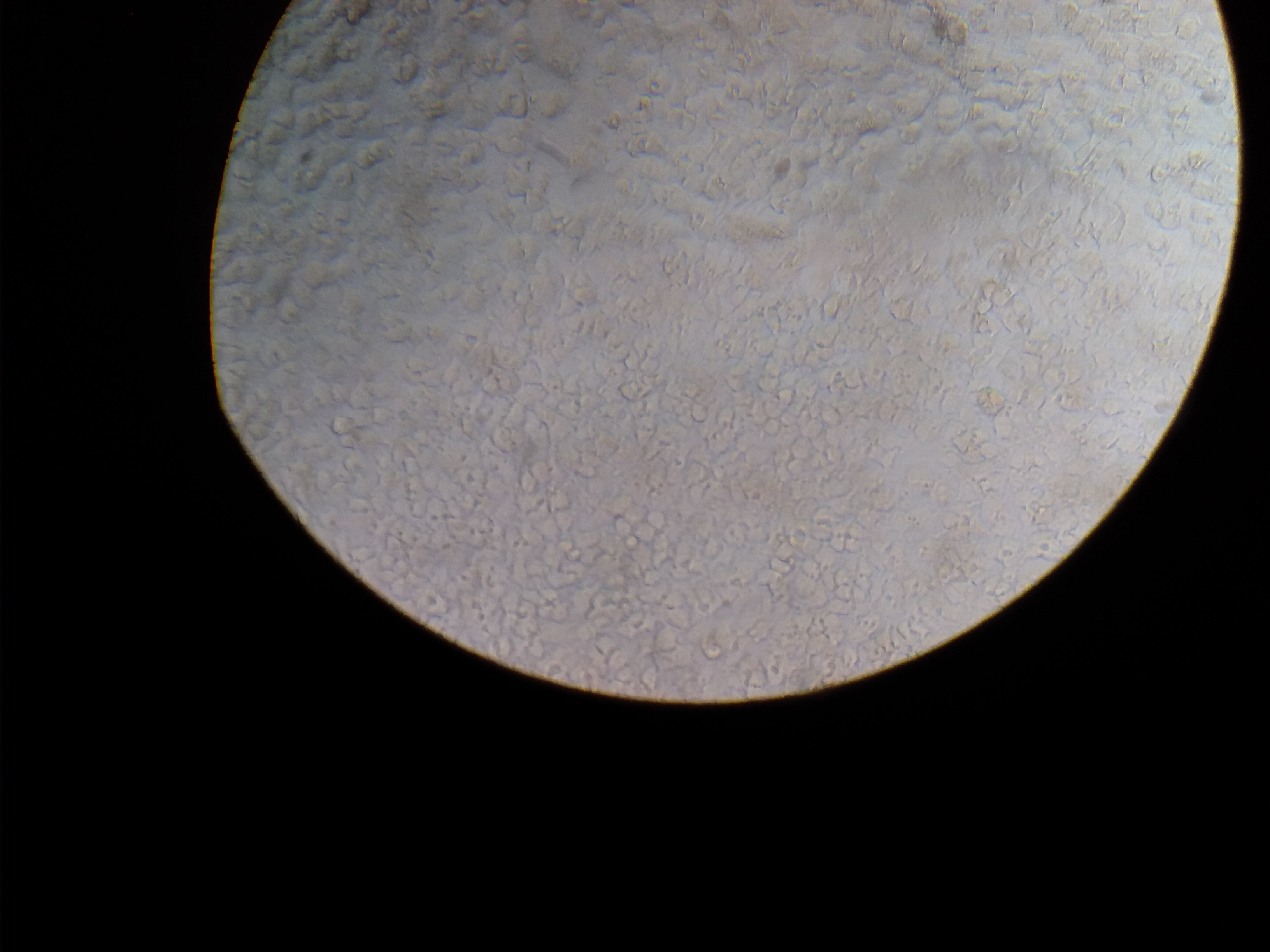

Supplement: Supplementary file 2 — Supplementary Information 2. [file 41598_2023_36721_MOESM2_ESM.zip › Raw data/Culture photos/20210609_175311.jpg]

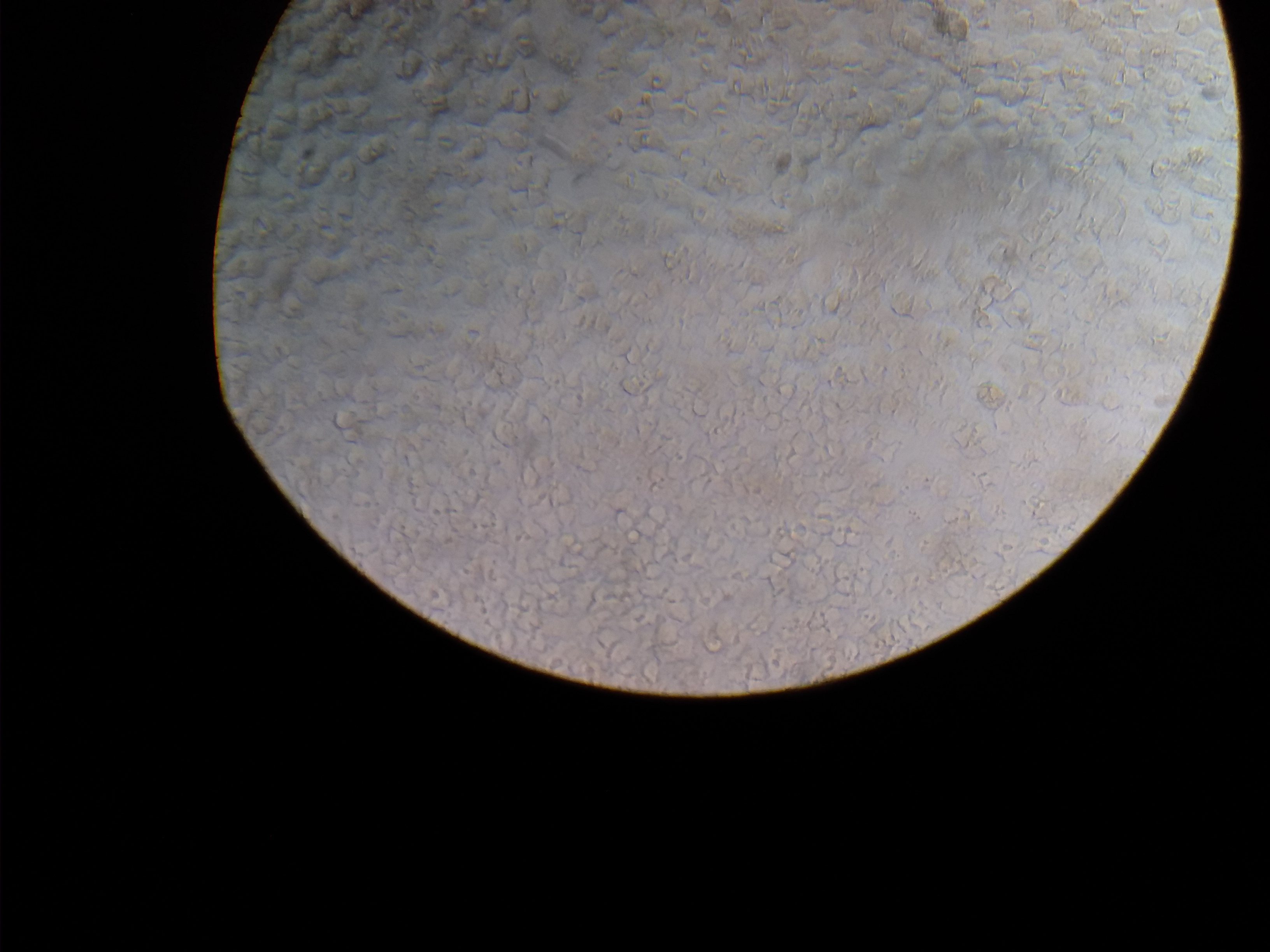

Supplement: Supplementary file 2 — Supplementary Information 2. [file 41598_2023_36721_MOESM2_ESM.zip › Raw data/Culture photos/20210609_175313.jpg]

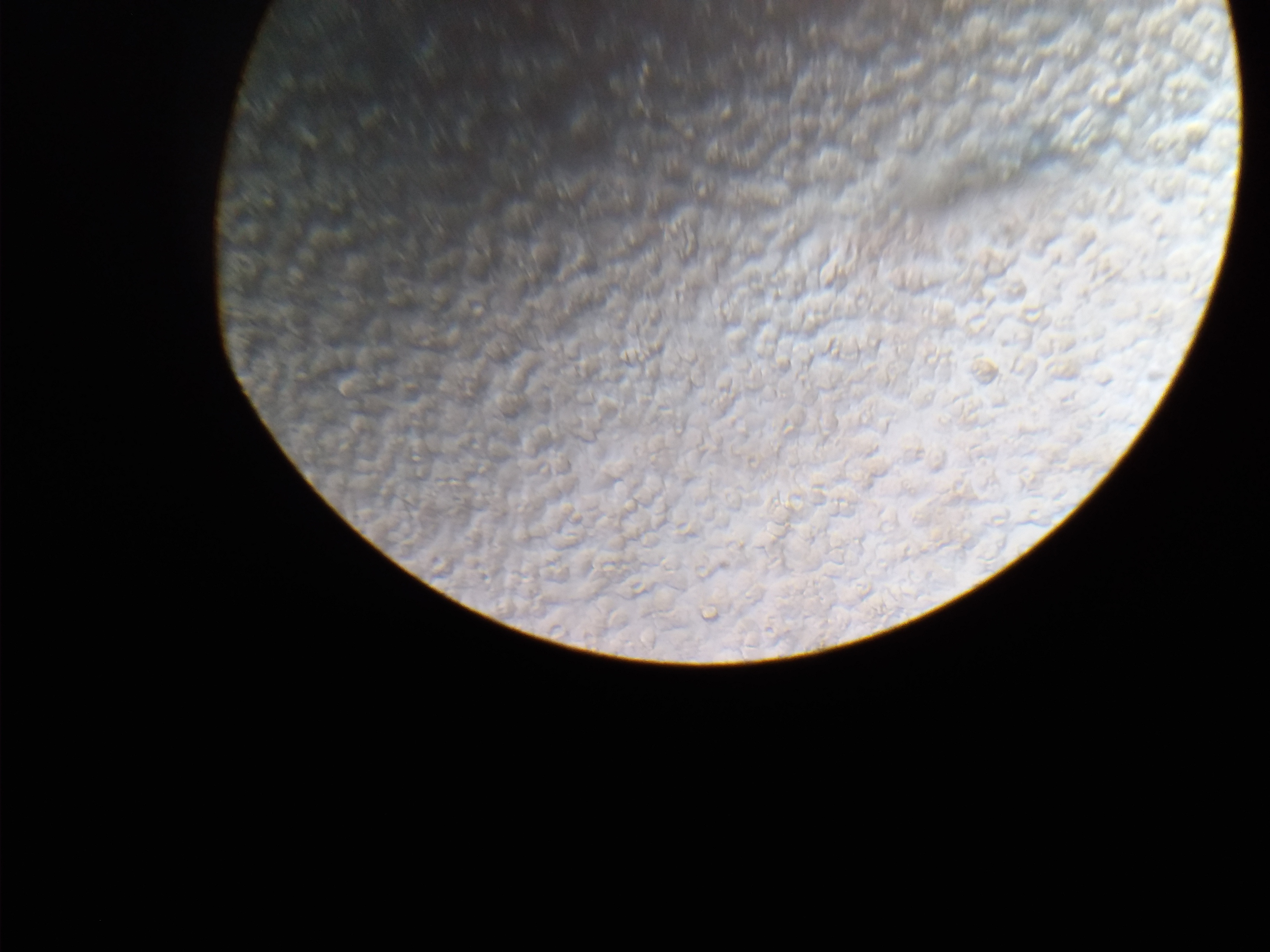

Supplement: Supplementary file 2 — Supplementary Information 2. [file 41598_2023_36721_MOESM2_ESM.zip › Raw data/Culture photos/20210609_175321.jpg]

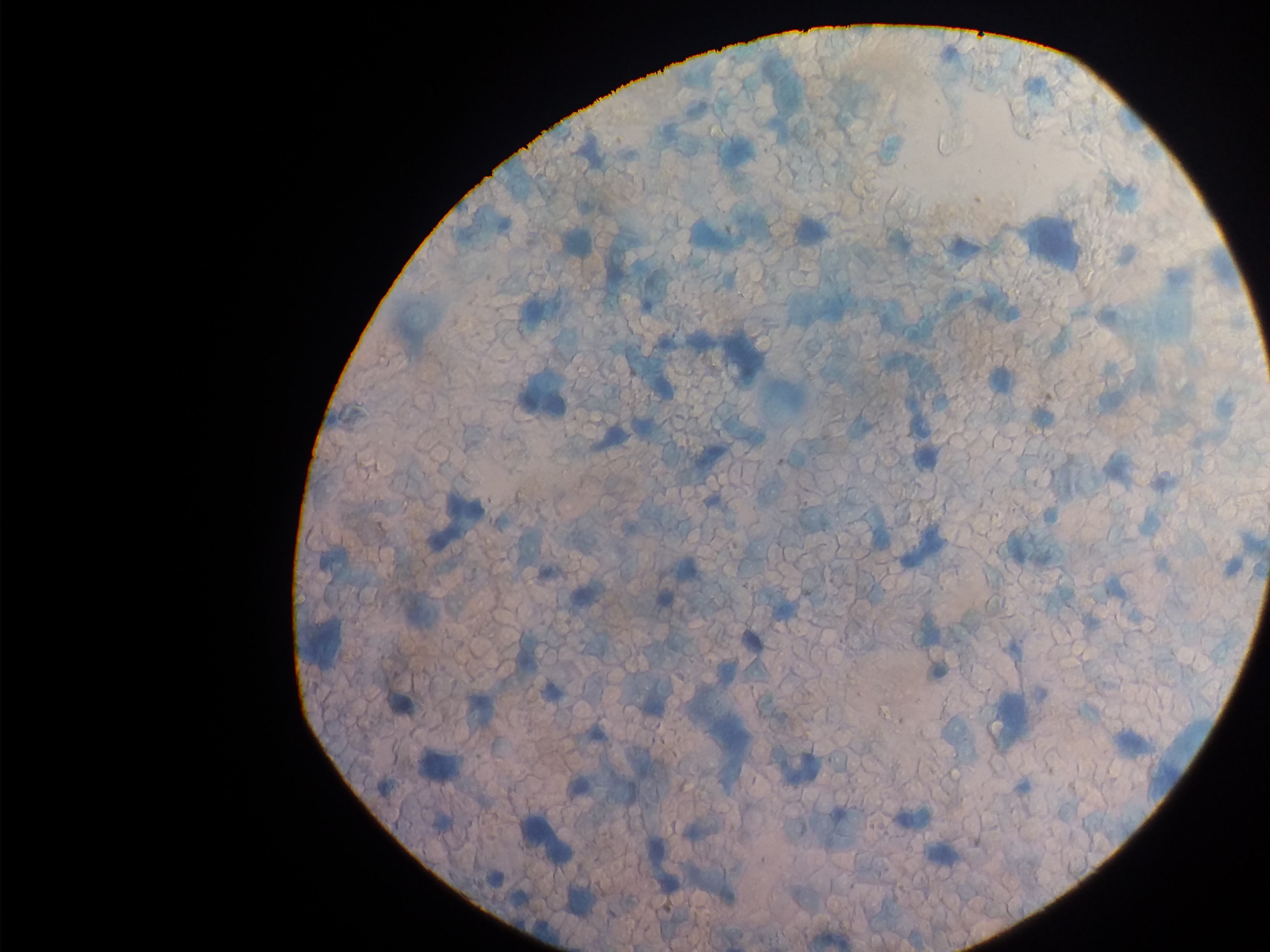

Supplement: Supplementary file 2 — Supplementary Information 2. [file 41598_2023_36721_MOESM2_ESM.zip › Raw data/Culture photos/20210609_175457.jpg]

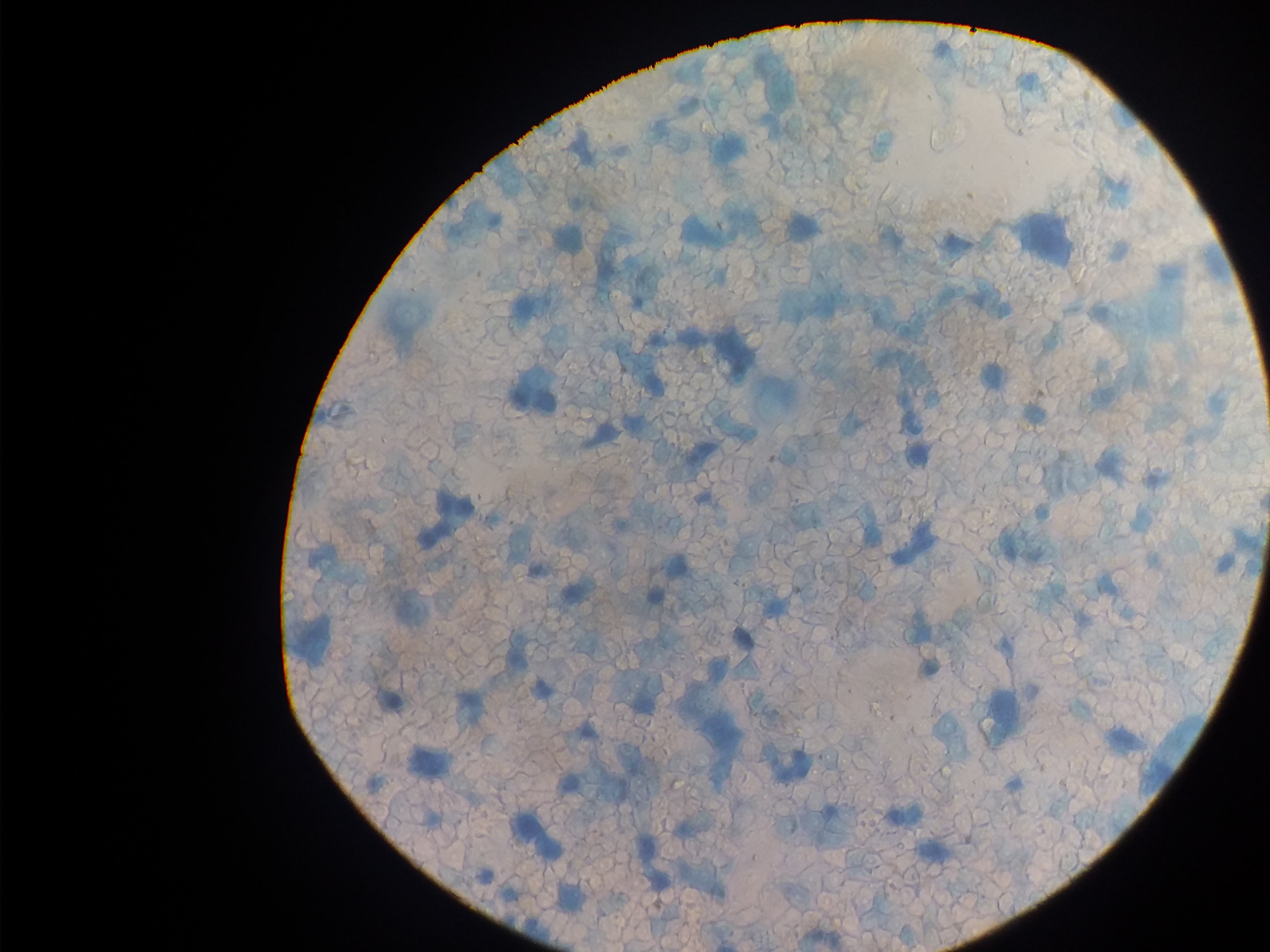

Supplement: Supplementary file 2 — Supplementary Information 2. [file 41598_2023_36721_MOESM2_ESM.zip › Raw data/Culture photos/20210609_175458.jpg]

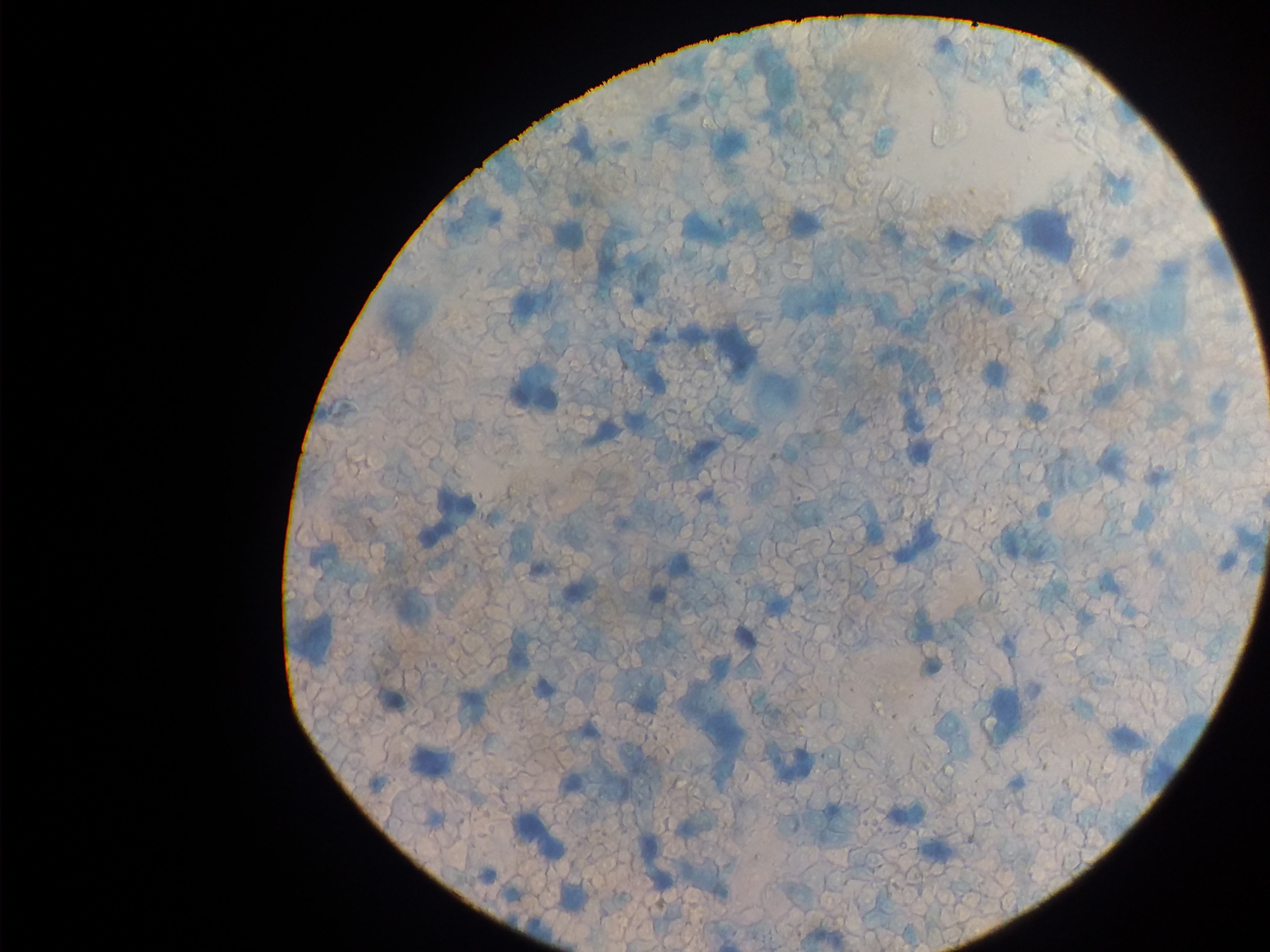

Supplement: Supplementary file 2 — Supplementary Information 2. [file 41598_2023_36721_MOESM2_ESM.zip › Raw data/Culture photos/20210609_175459.jpg]

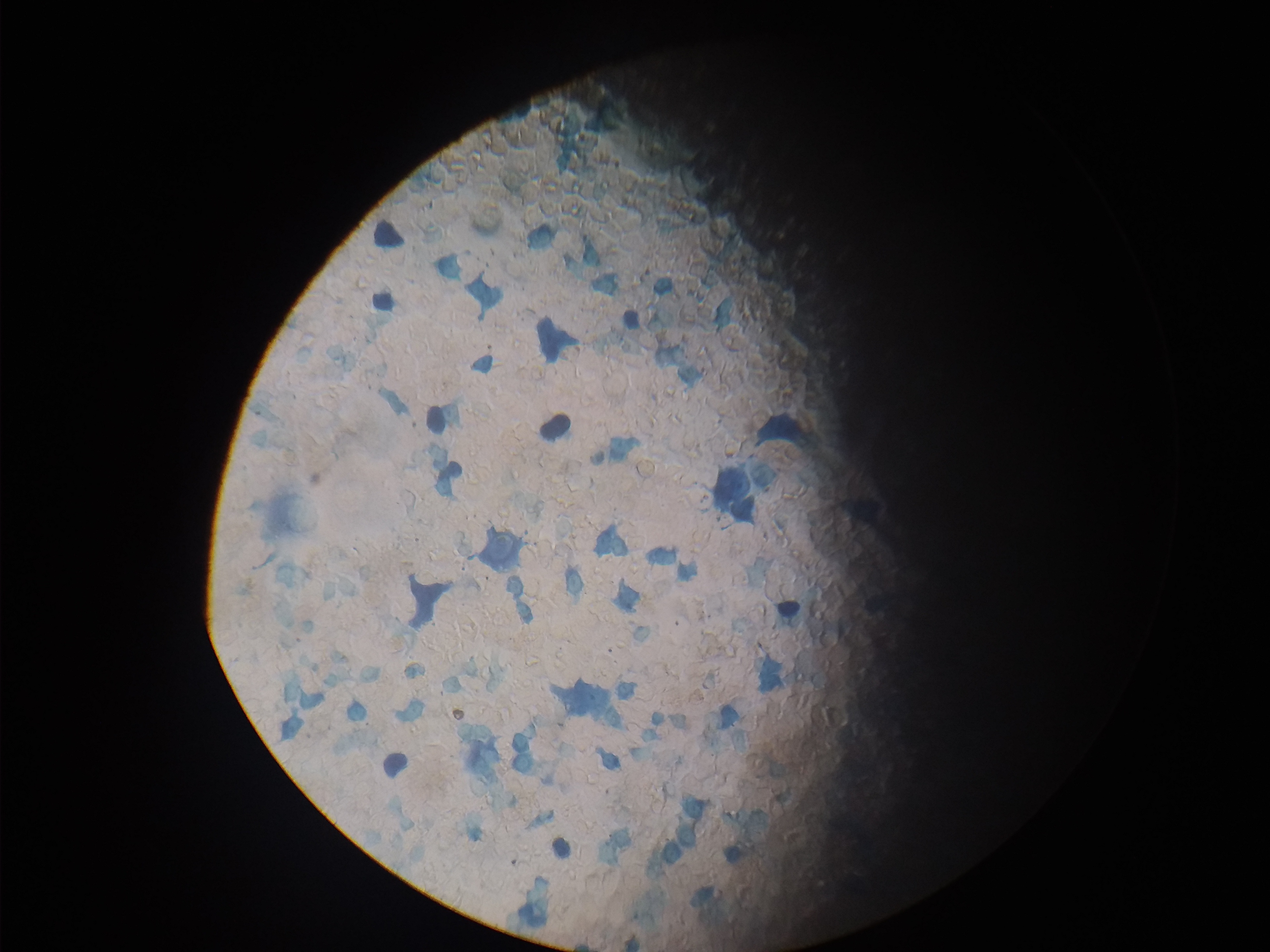

Supplement: Supplementary file 2 — Supplementary Information 2. [file 41598_2023_36721_MOESM2_ESM.zip › Raw data/Culture photos/20210609_175539.jpg]

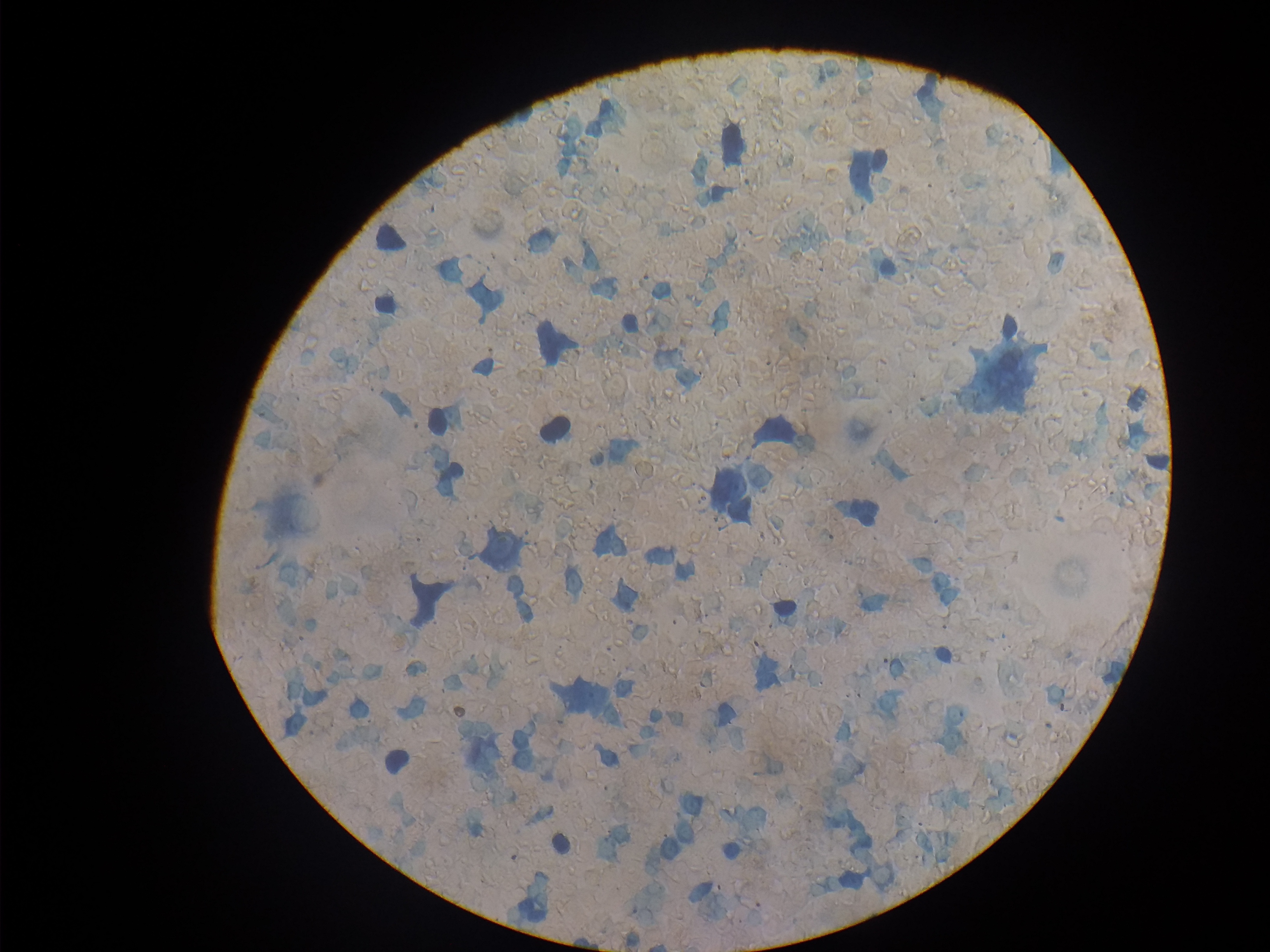

Supplement: Supplementary file 2 — Supplementary Information 2. [file 41598_2023_36721_MOESM2_ESM.zip › Raw data/Culture photos/20210609_175540.jpg]

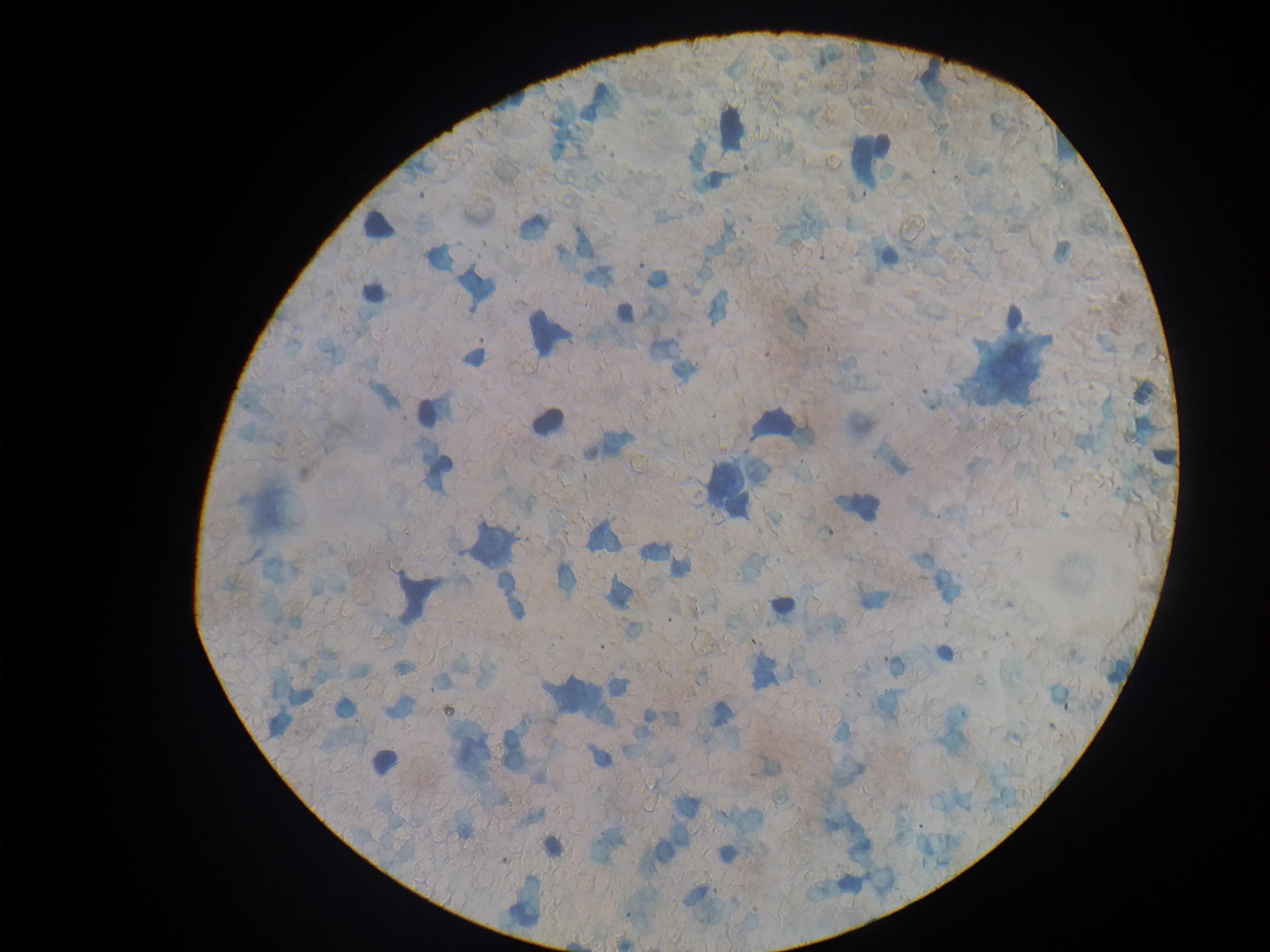

Supplement: Supplementary file 2 — Supplementary Information 2. [file 41598_2023_36721_MOESM2_ESM.zip › Raw data/Culture photos/20210609_175542.jpg]

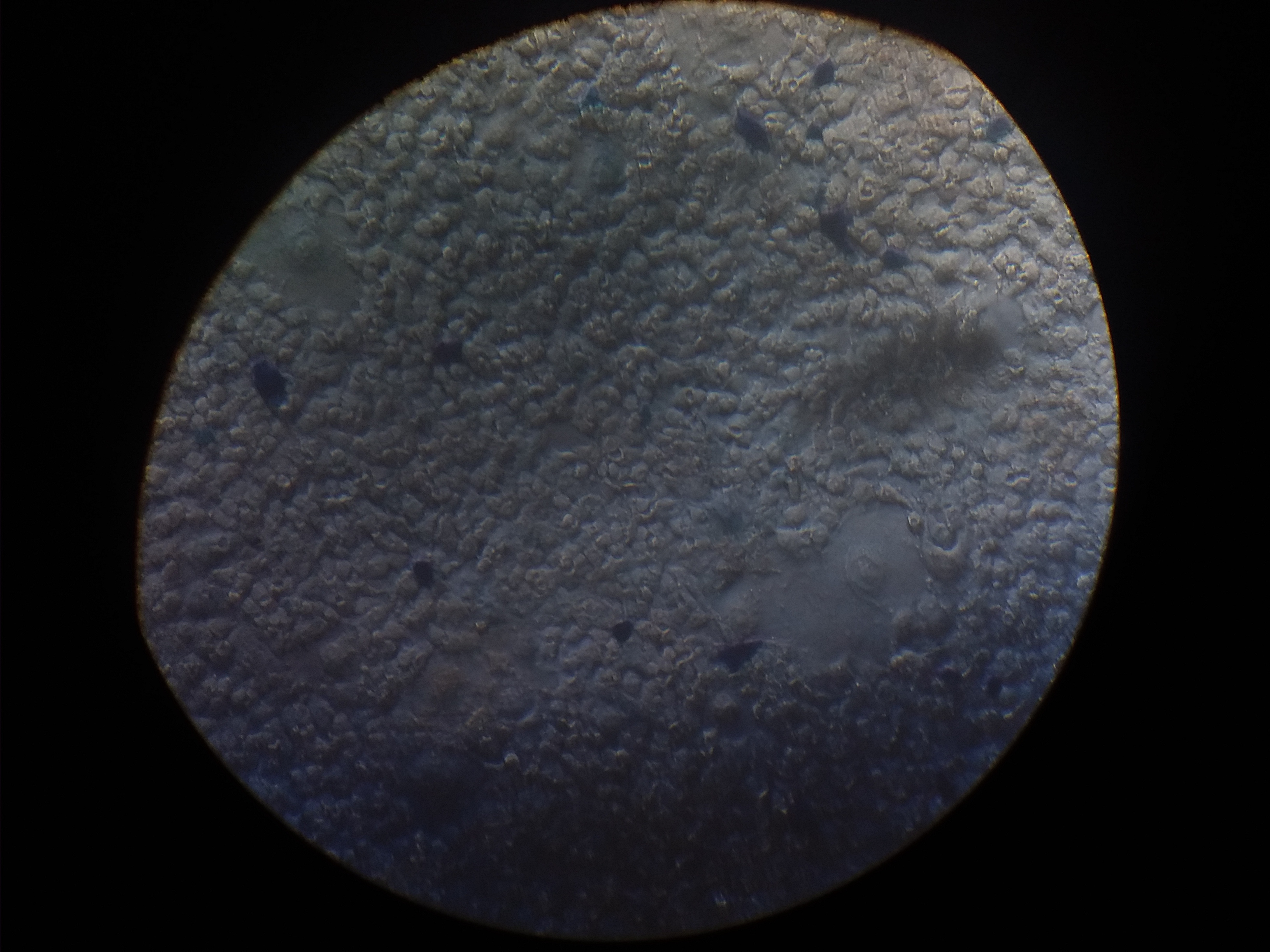

Supplement: Supplementary file 2 — Supplementary Information 2. [file 41598_2023_36721_MOESM2_ESM.zip › Raw data/Culture photos/20210609_175612.jpg]

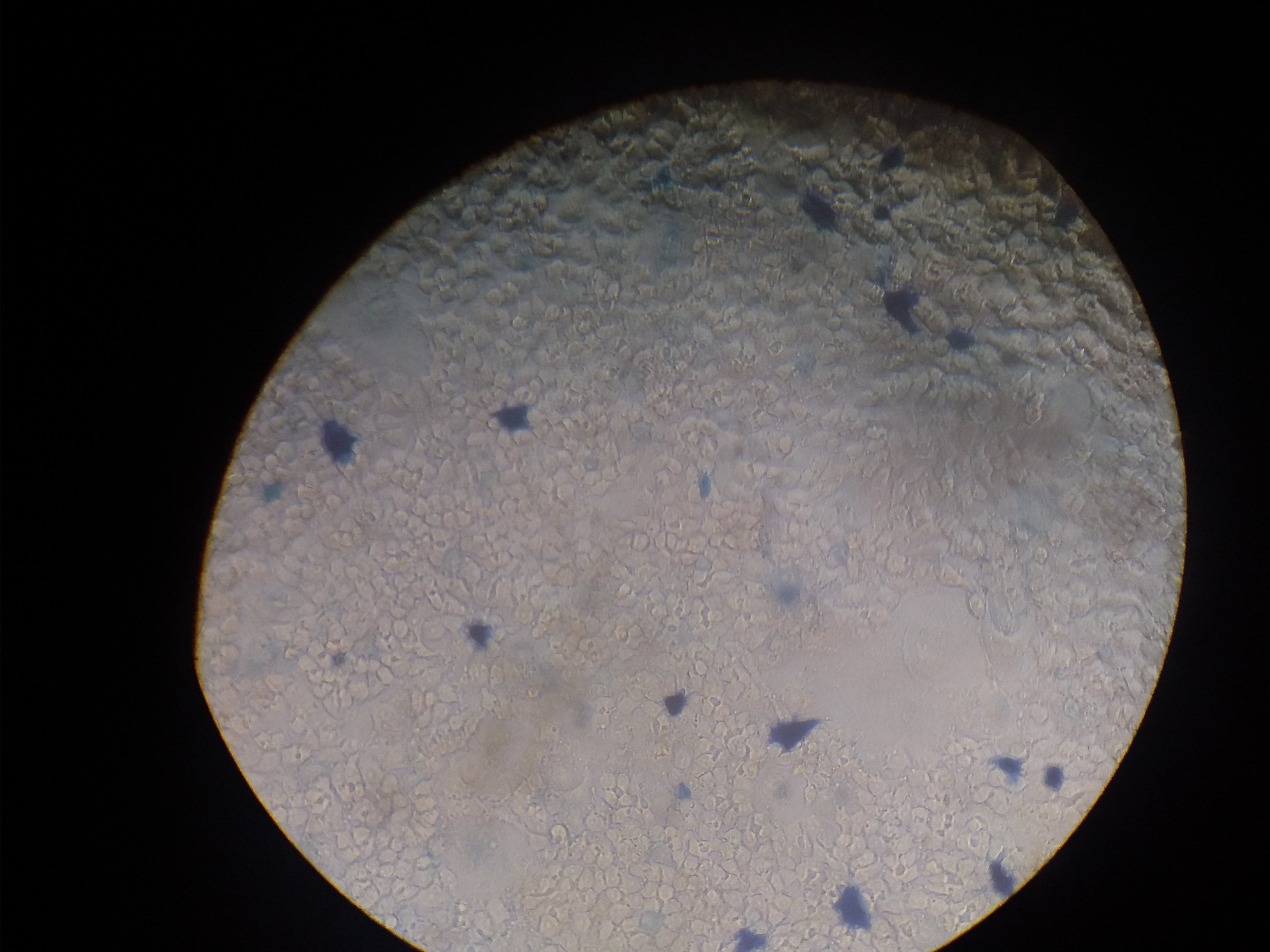

Supplement: Supplementary file 2 — Supplementary Information 2. [file 41598_2023_36721_MOESM2_ESM.zip › Raw data/Culture photos/20210609_175647.jpg]

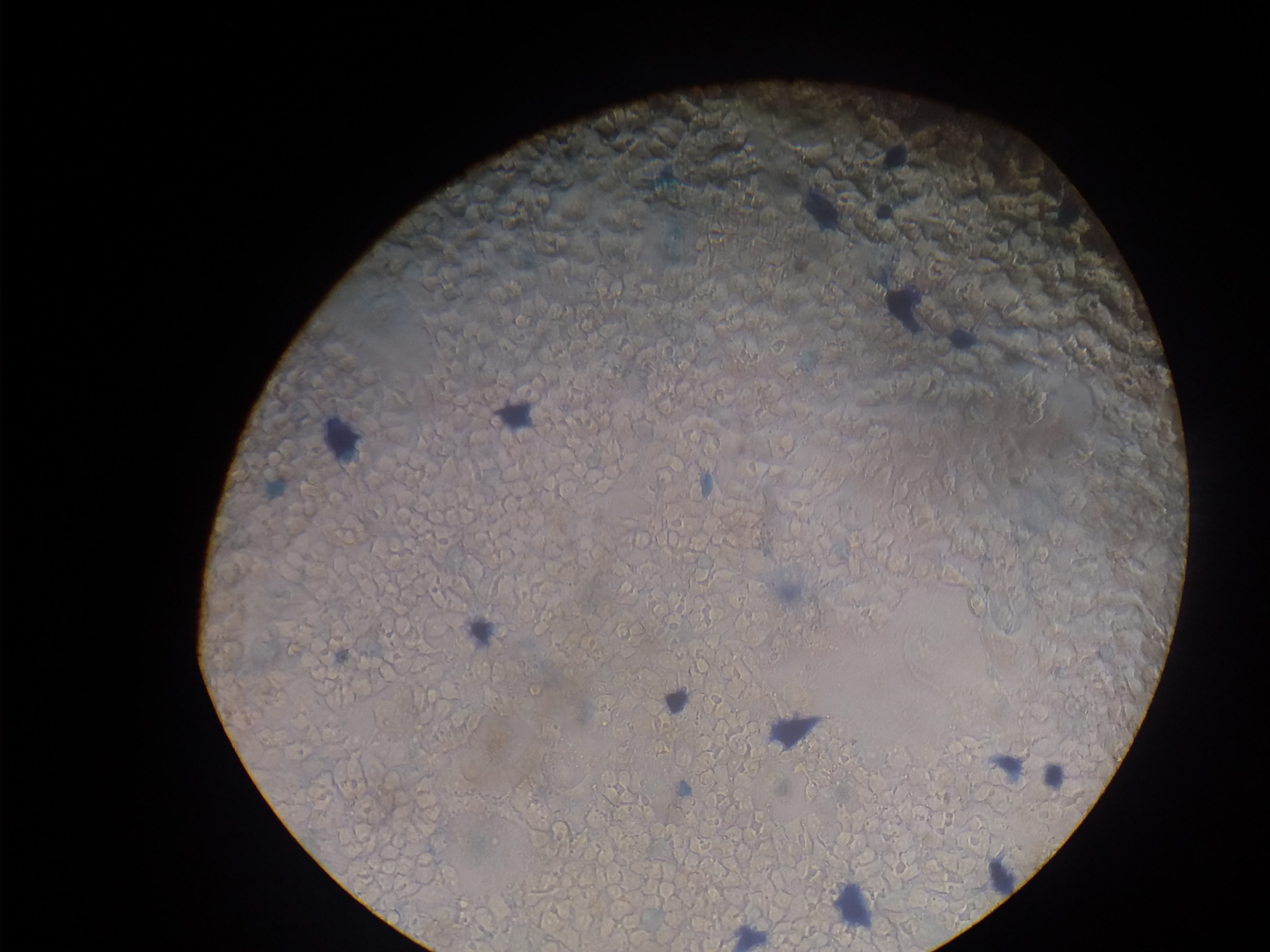

Supplement: Supplementary file 2 — Supplementary Information 2. [file 41598_2023_36721_MOESM2_ESM.zip › Raw data/Culture photos/20210609_175649.jpg]

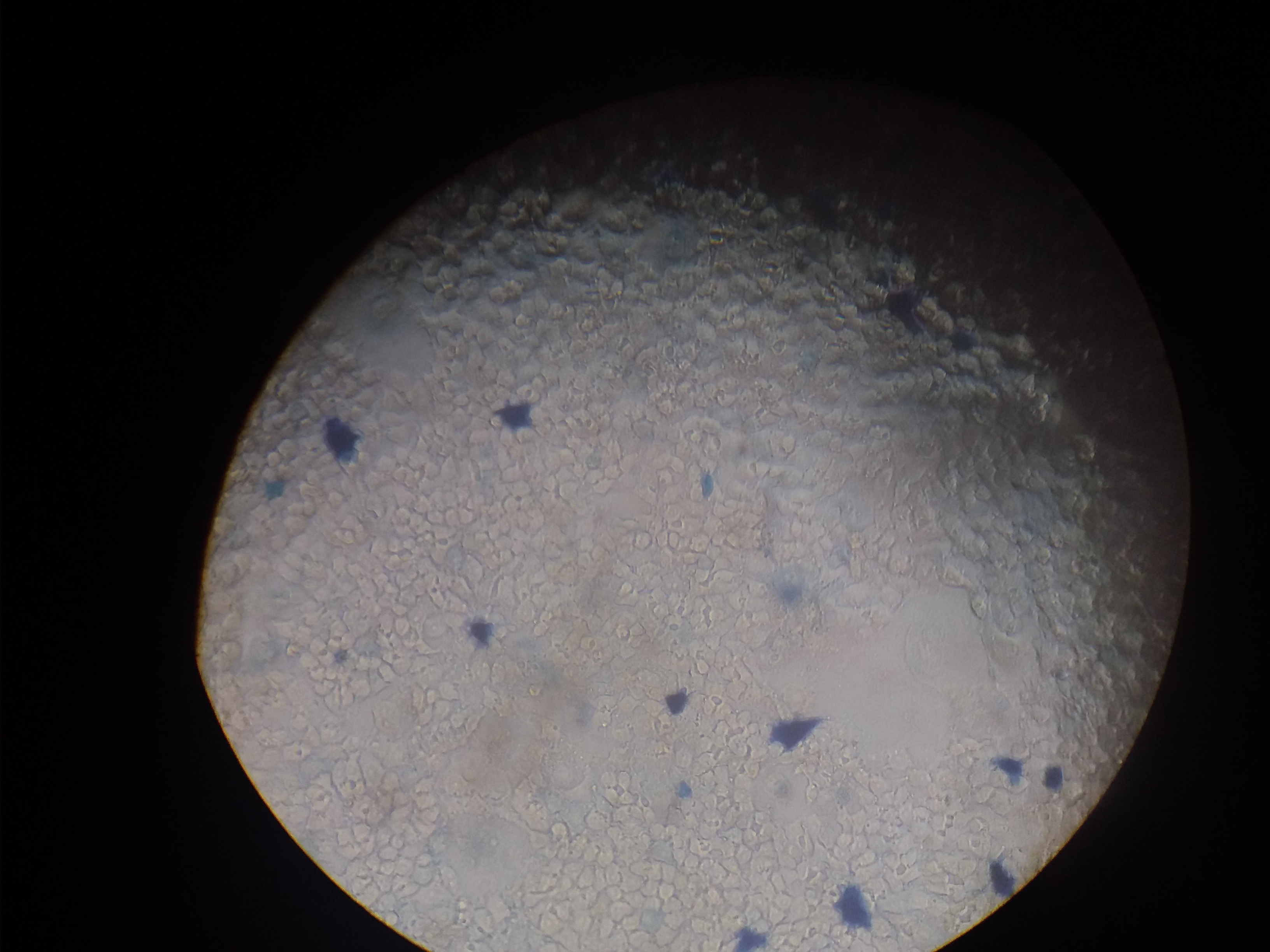

Supplement: Supplementary file 2 — Supplementary Information 2. [file 41598_2023_36721_MOESM2_ESM.zip › Raw data/Culture photos/20210609_175651.jpg]

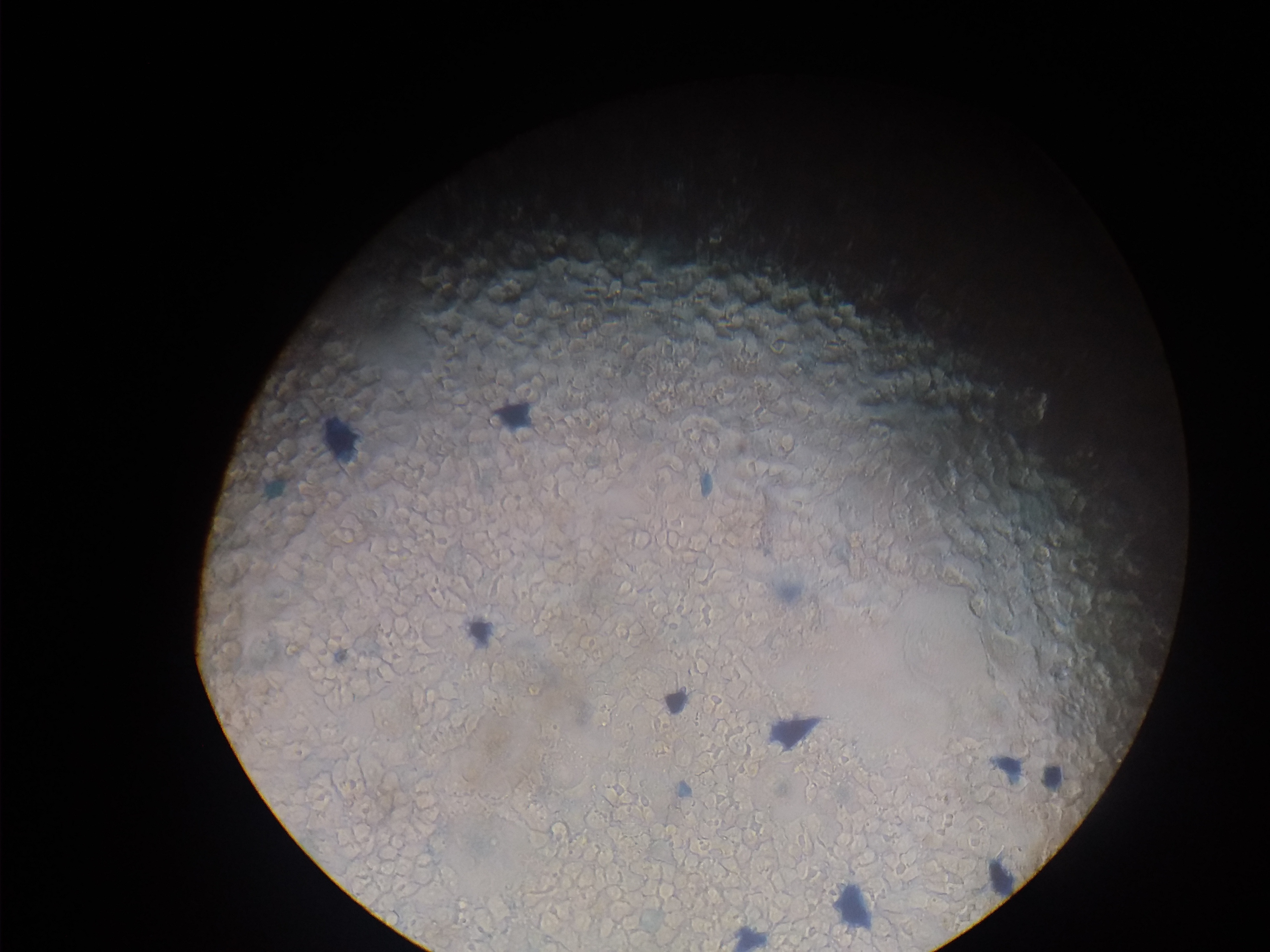

Supplement: Supplementary file 2 — Supplementary Information 2. [file 41598_2023_36721_MOESM2_ESM.zip › Raw data/Culture photos/20210609_175652.jpg]

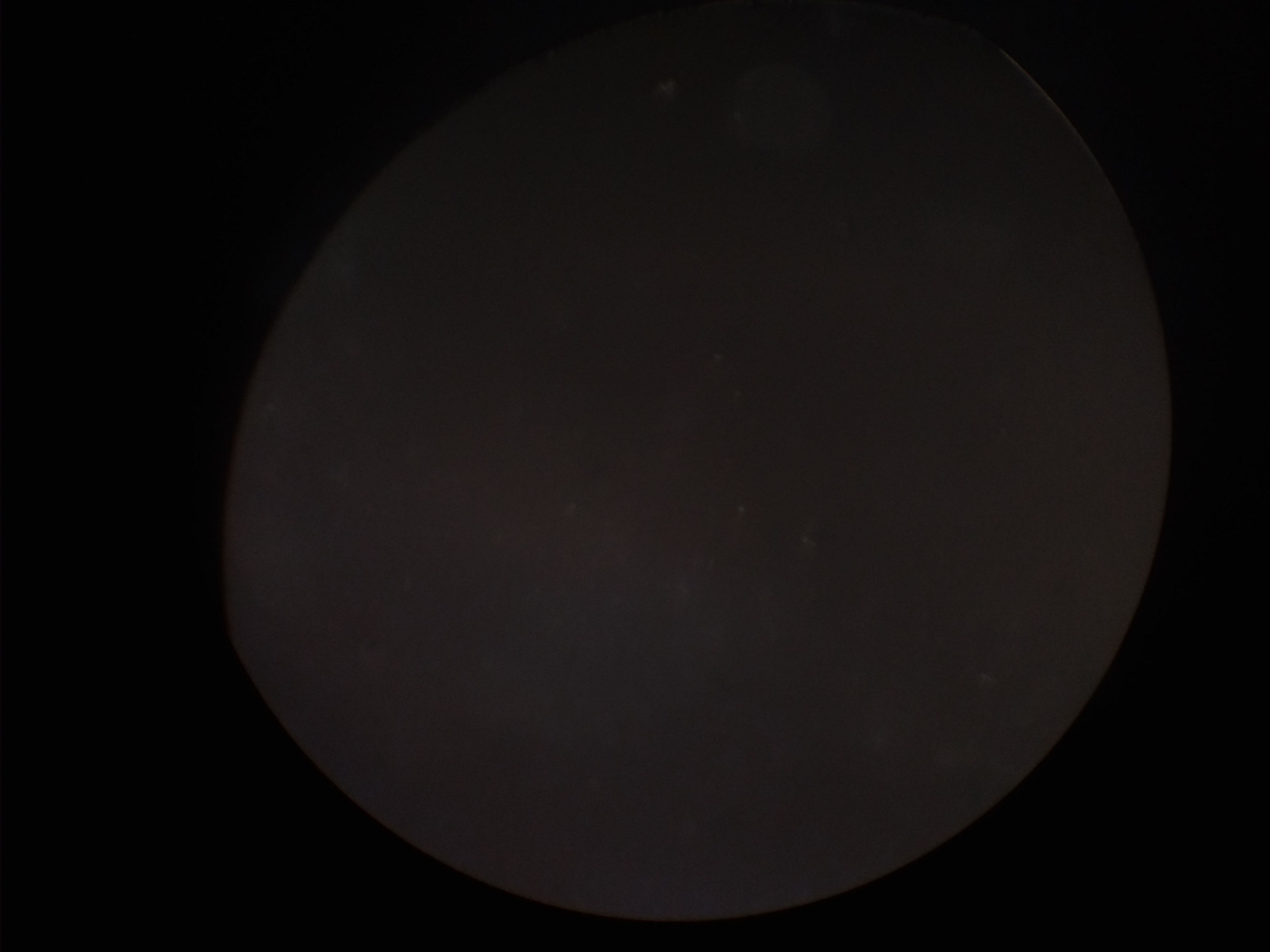

Supplement: Supplementary file 2 — Supplementary Information 2. [file 41598_2023_36721_MOESM2_ESM.zip › Raw data/Culture photos/20210609_175718.jpg]

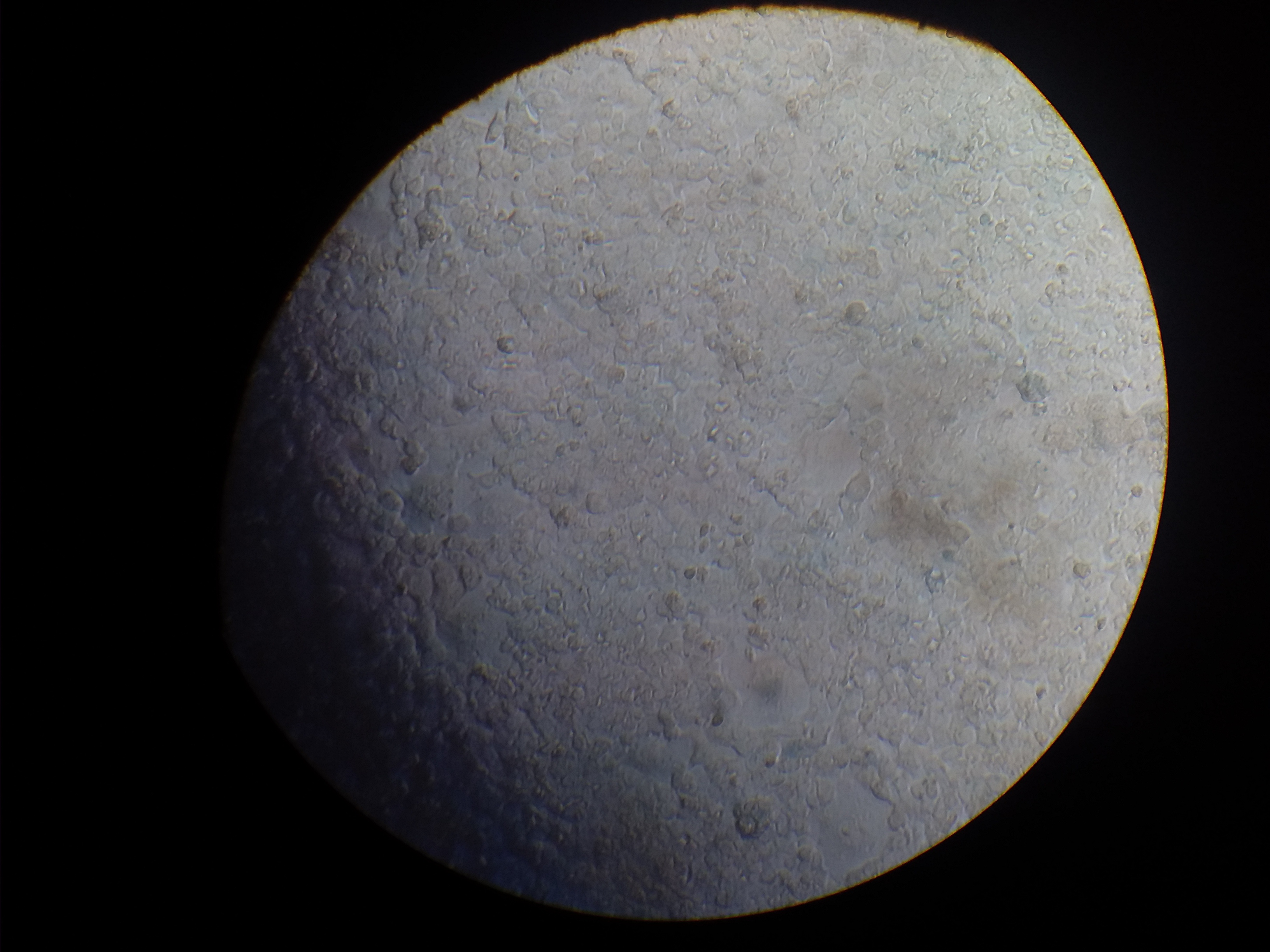

Supplement: Supplementary file 2 — Supplementary Information 2. [file 41598_2023_36721_MOESM2_ESM.zip › Raw data/Culture photos/20210609_175724.jpg]

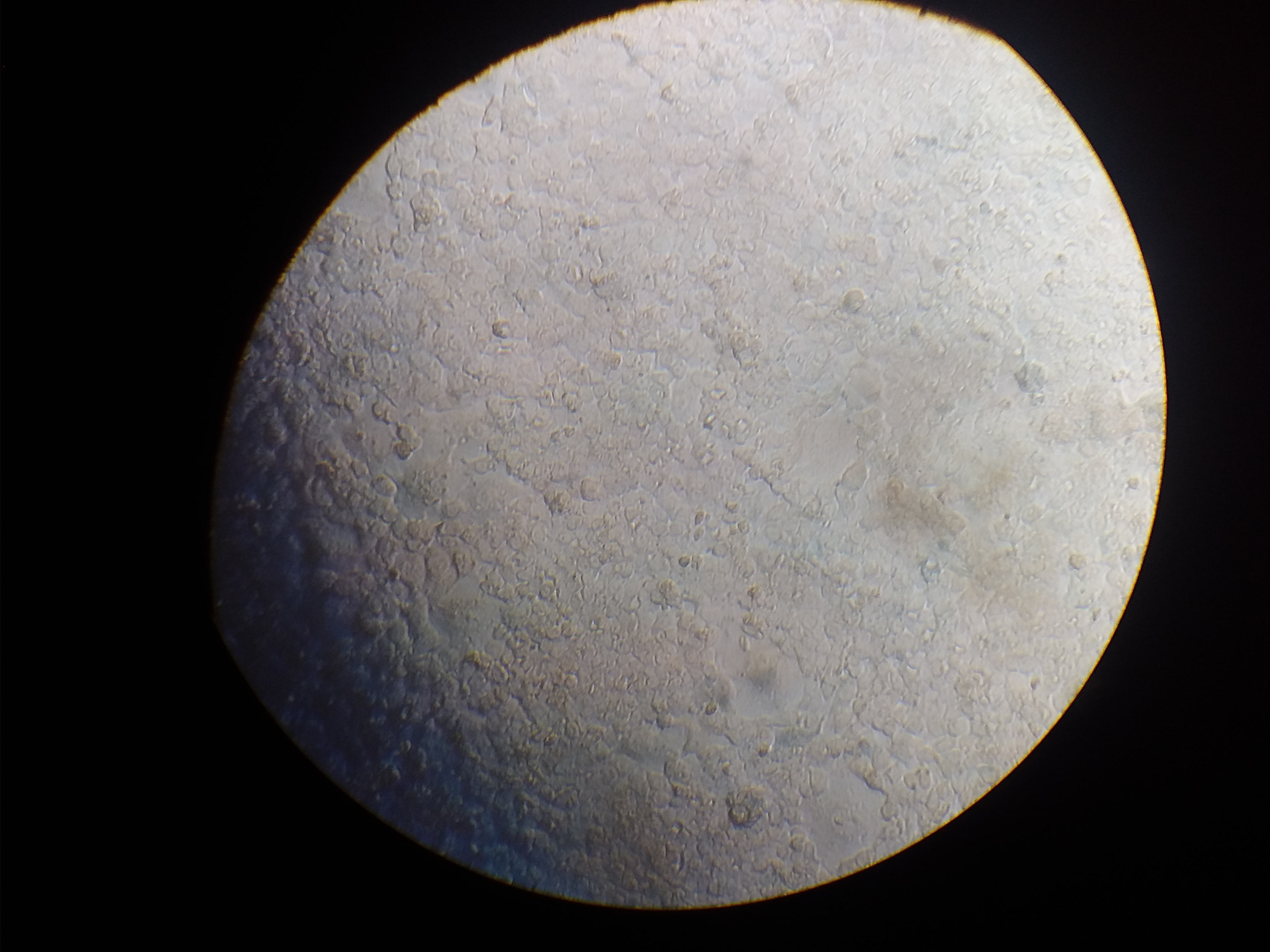

Supplement: Supplementary file 2 — Supplementary Information 2. [file 41598_2023_36721_MOESM2_ESM.zip › Raw data/Culture photos/20210609_175730.jpg]

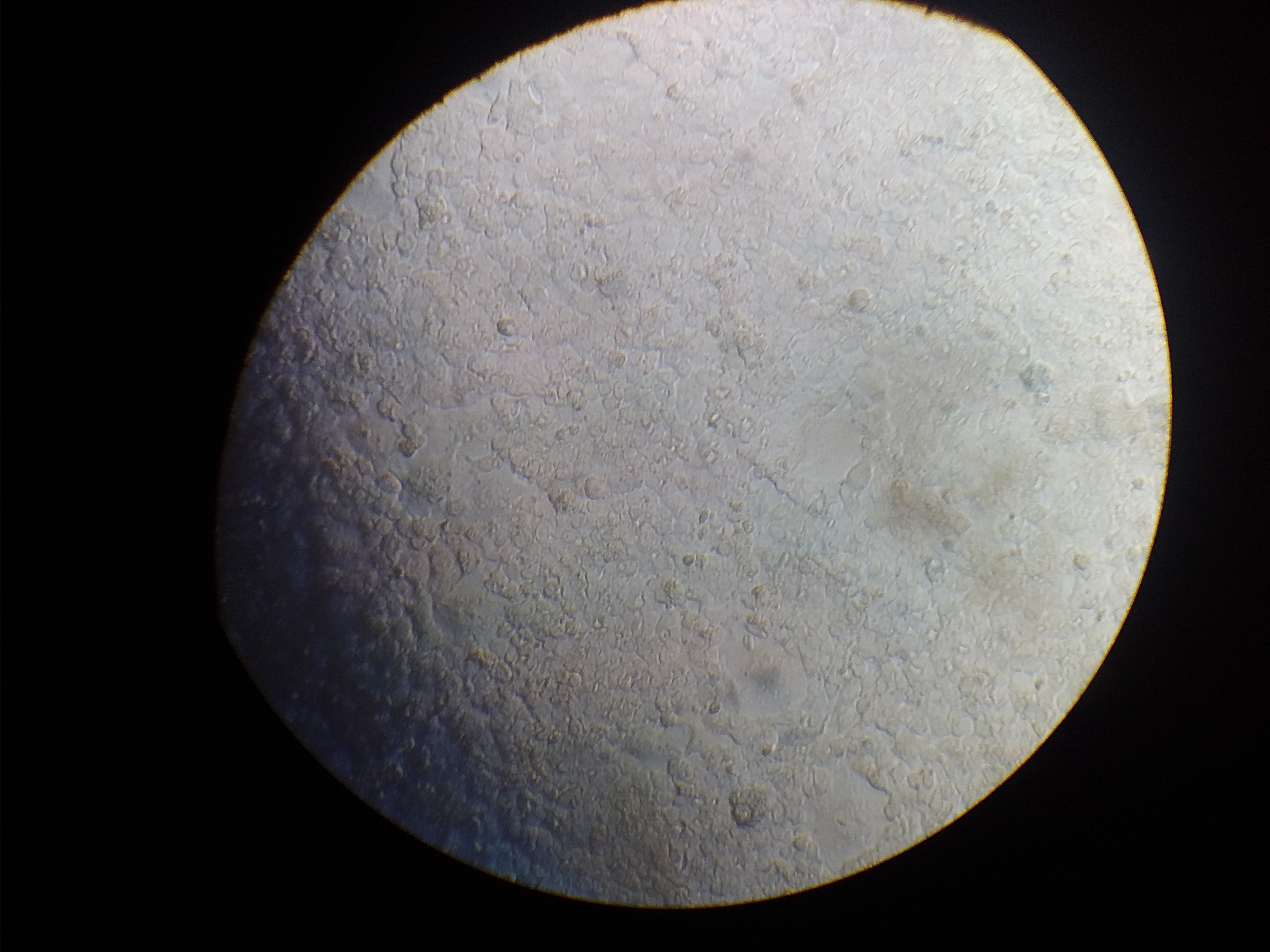

Supplement: Supplementary file 2 — Supplementary Information 2. [file 41598_2023_36721_MOESM2_ESM.zip › Raw data/Culture photos/20210609_175735.jpg]

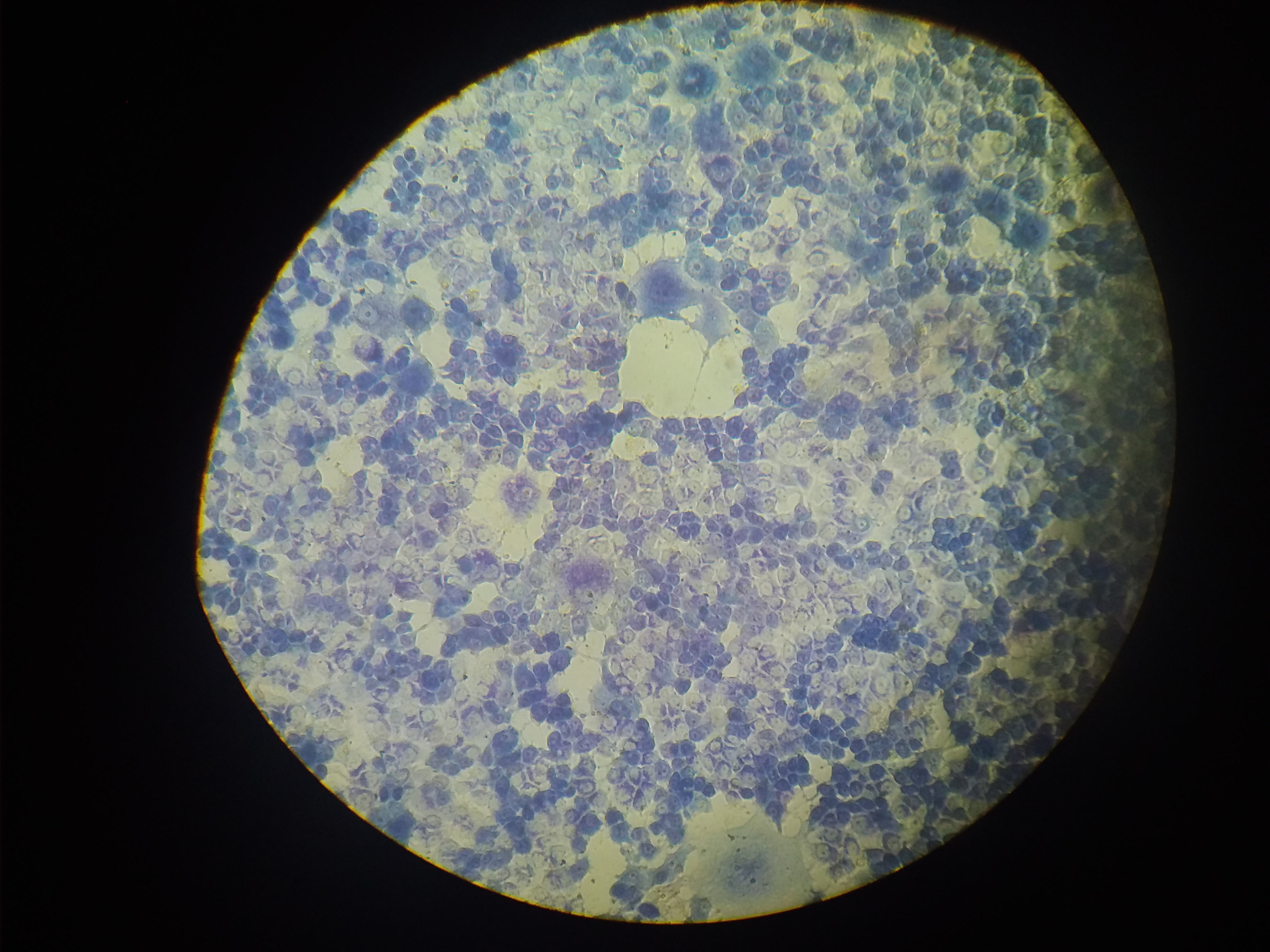

Supplement: Supplementary file 2 — Supplementary Information 2. [file 41598_2023_36721_MOESM2_ESM.zip › Raw data/Culture photos/20210609_175836.jpg]

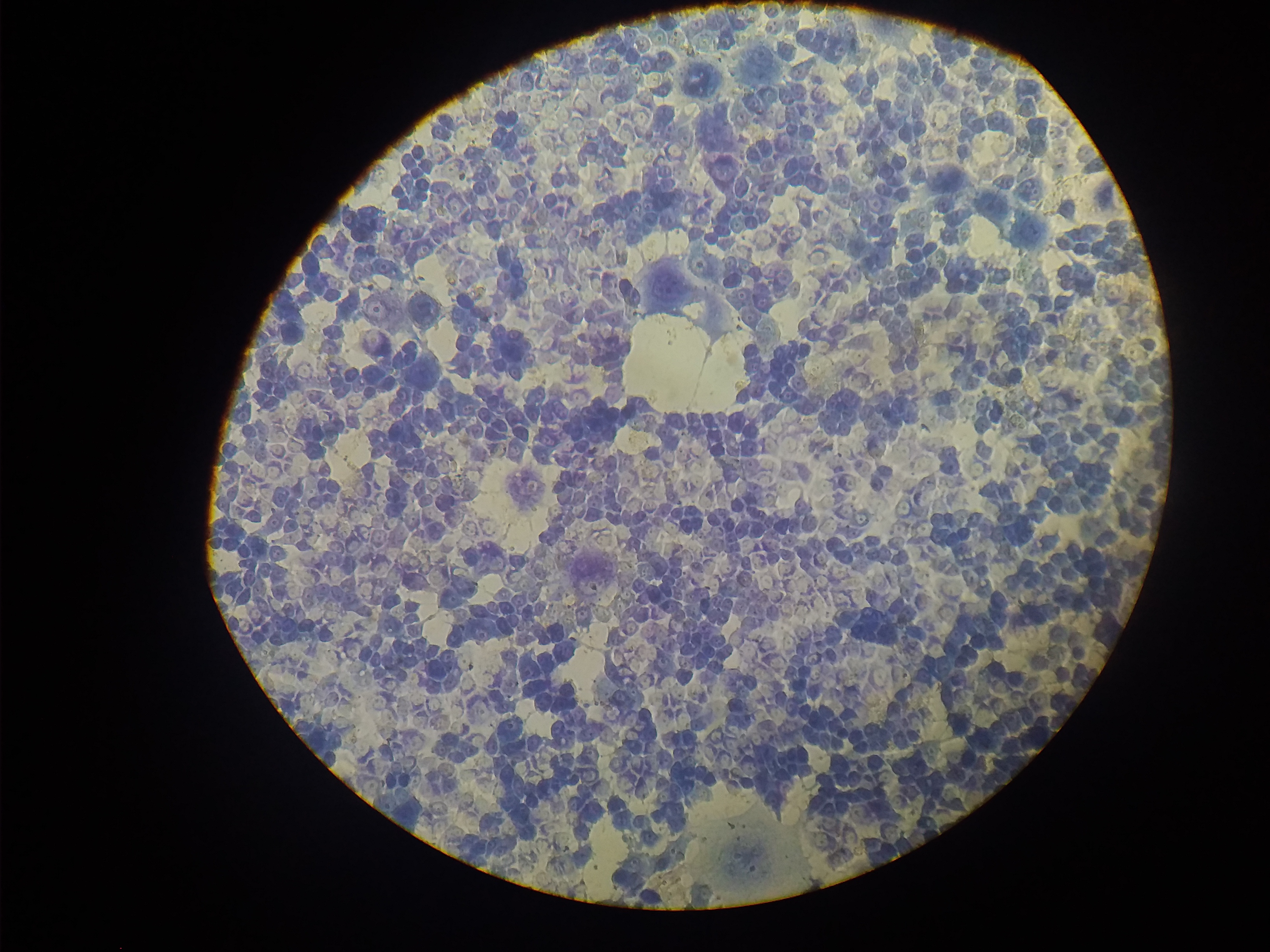

Supplement: Supplementary file 2 — Supplementary Information 2. [file 41598_2023_36721_MOESM2_ESM.zip › Raw data/Culture photos/20210609_175847(0).jpg]

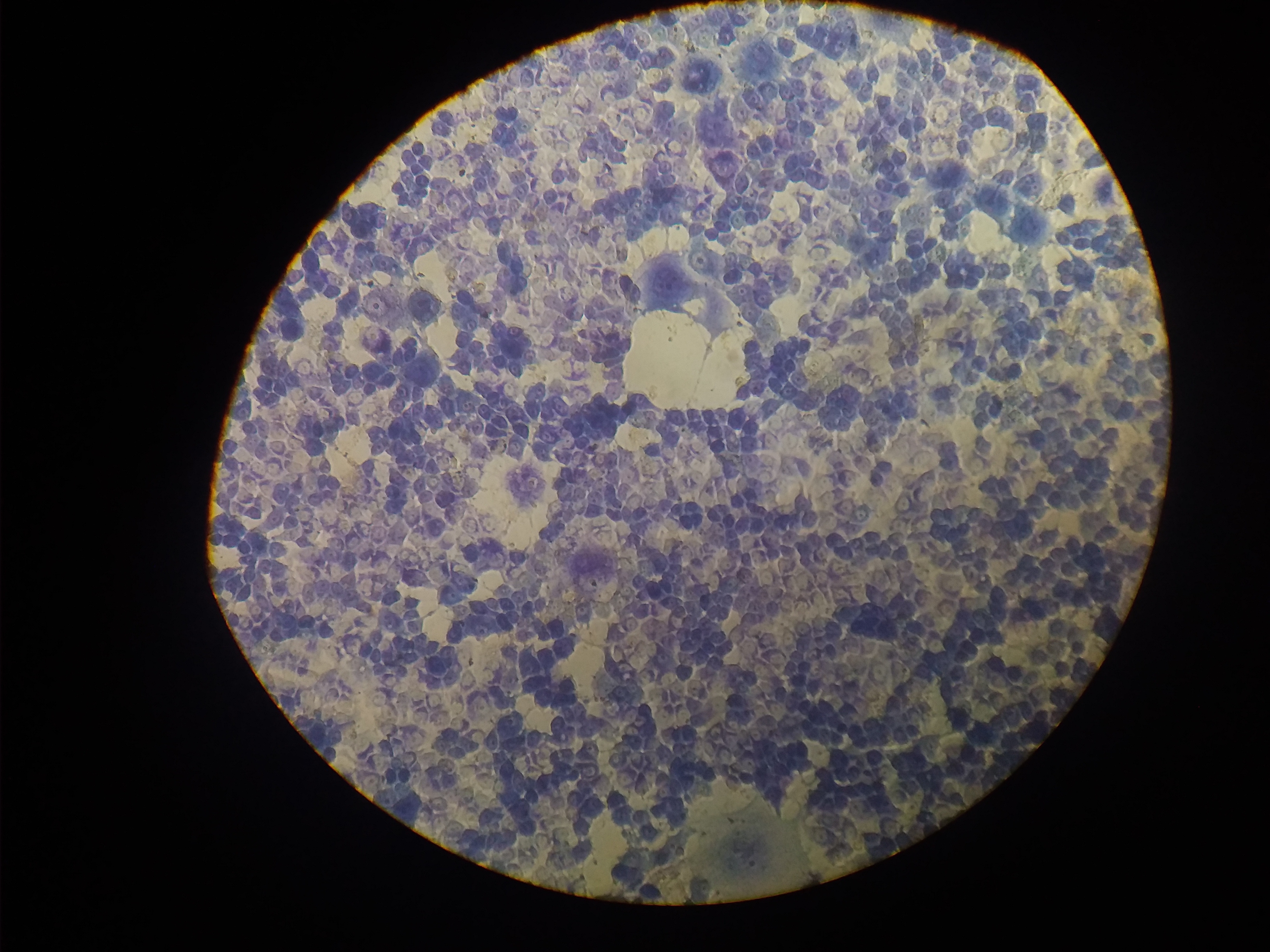

Supplement: Supplementary file 2 — Supplementary Information 2. [file 41598_2023_36721_MOESM2_ESM.zip › Raw data/Culture photos/20210609_175847.jpg]

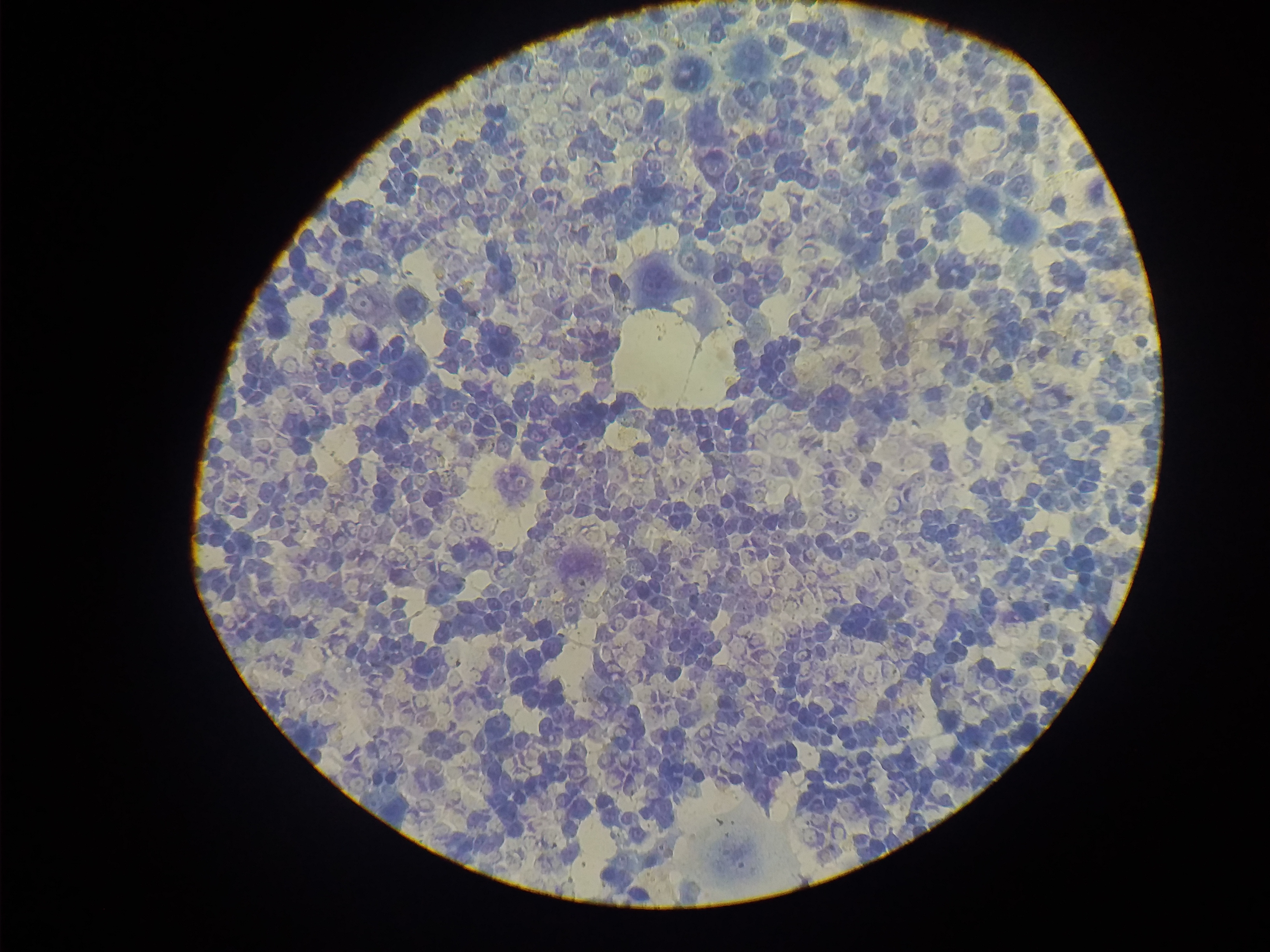

Supplement: Supplementary file 2 — Supplementary Information 2. [file 41598_2023_36721_MOESM2_ESM.zip › Raw data/Culture photos/20210609_175849.jpg]

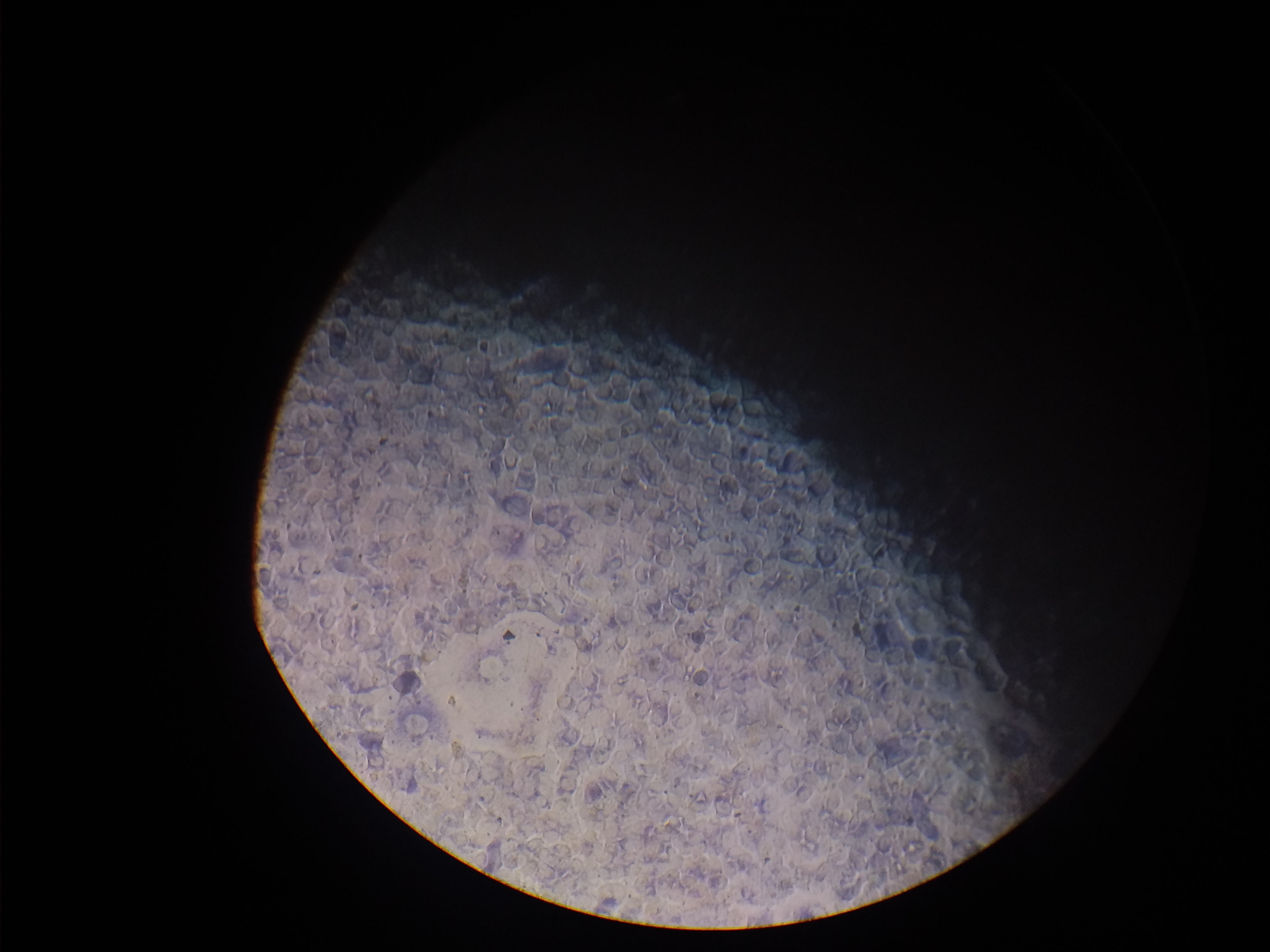

Supplement: Supplementary file 2 — Supplementary Information 2. [file 41598_2023_36721_MOESM2_ESM.zip › Raw data/Culture photos/20210609_175916.jpg]

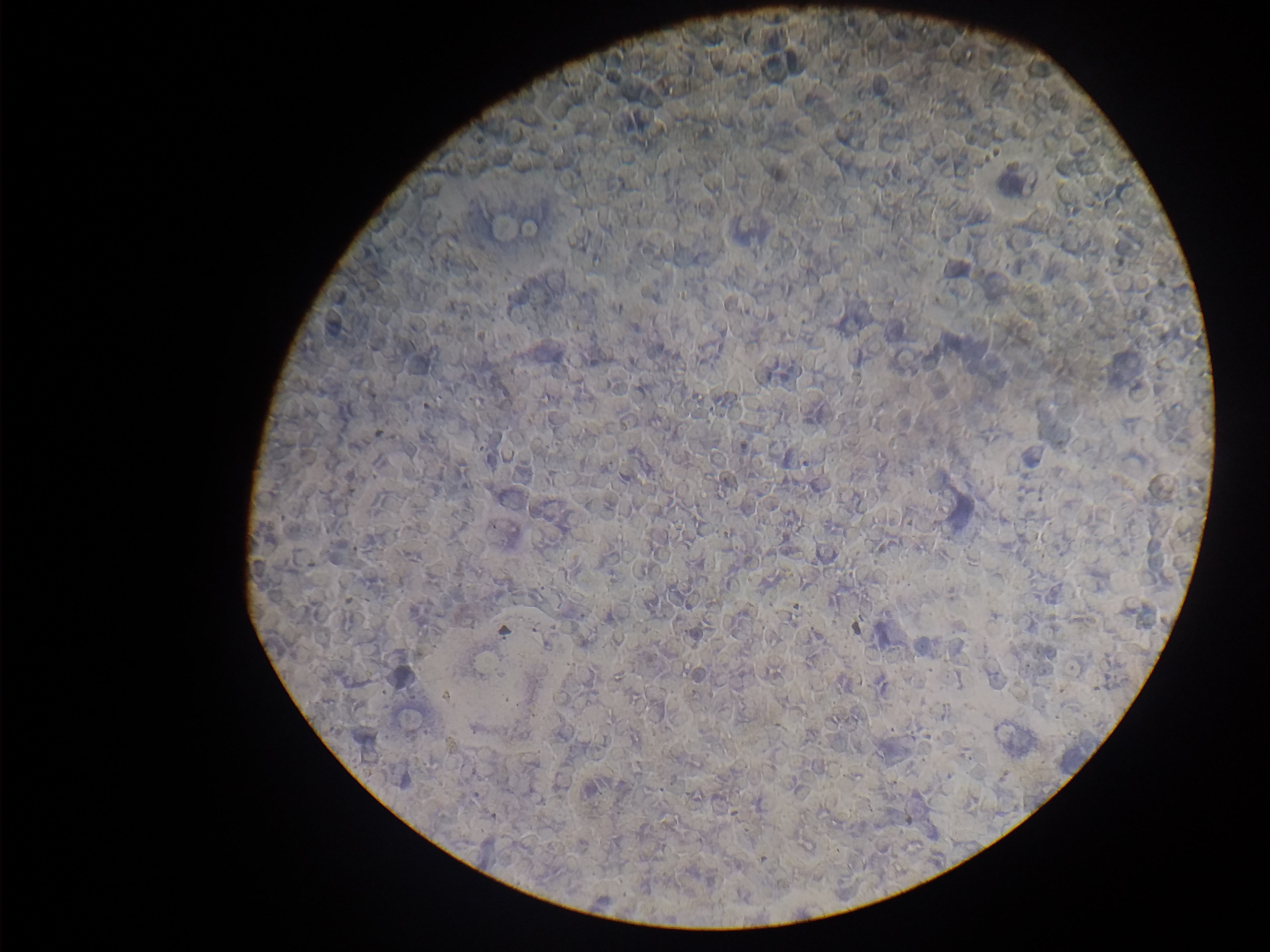

Supplement: Supplementary file 2 — Supplementary Information 2. [file 41598_2023_36721_MOESM2_ESM.zip › Raw data/Culture photos/20210609_175918.jpg]

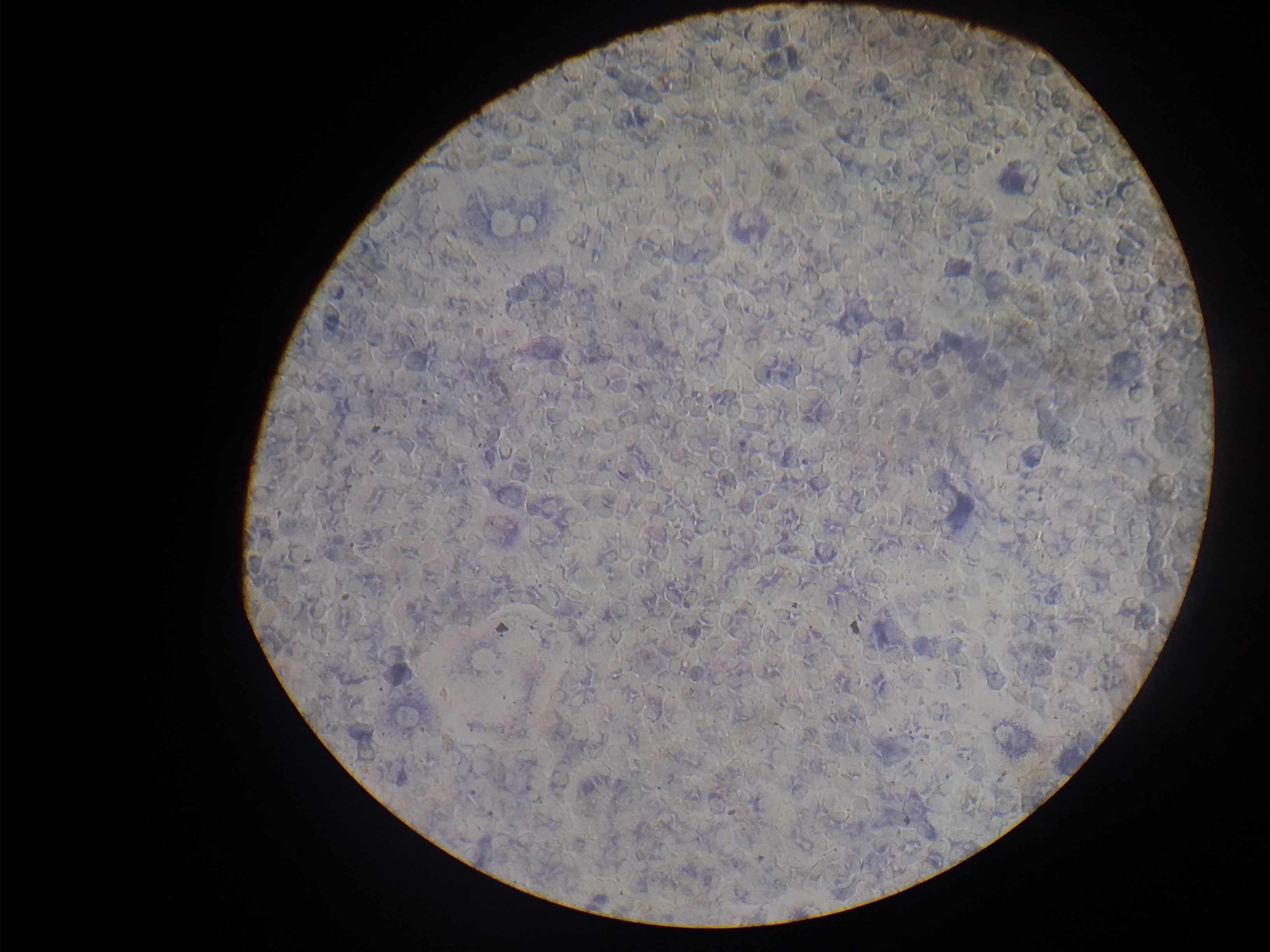

Supplement: Supplementary file 2 — Supplementary Information 2. [file 41598_2023_36721_MOESM2_ESM.zip › Raw data/Culture photos/20210609_175920(0).jpg]

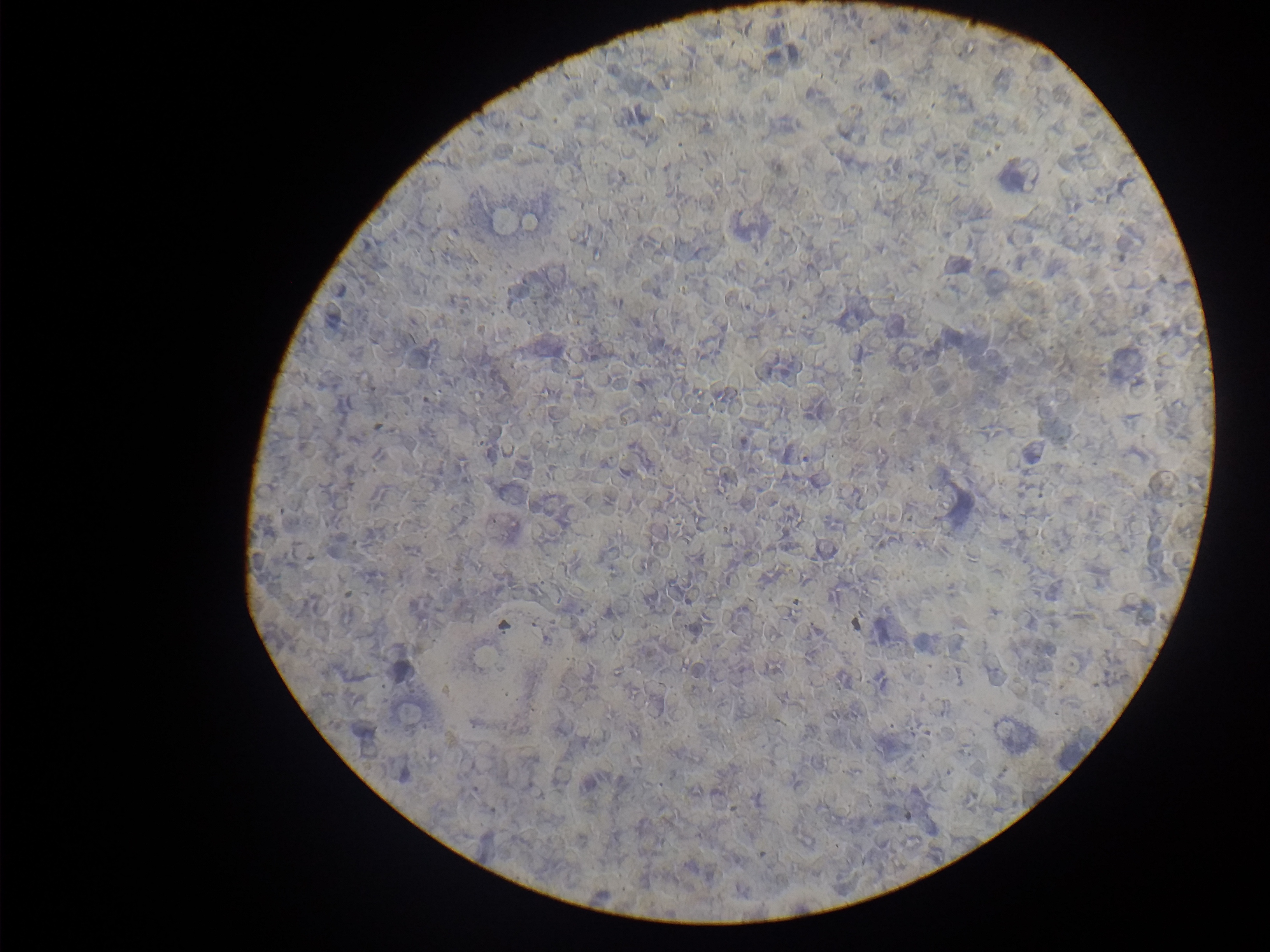

Supplement: Supplementary file 2 — Supplementary Information 2. [file 41598_2023_36721_MOESM2_ESM.zip › Raw data/Culture photos/20210609_175920.jpg]

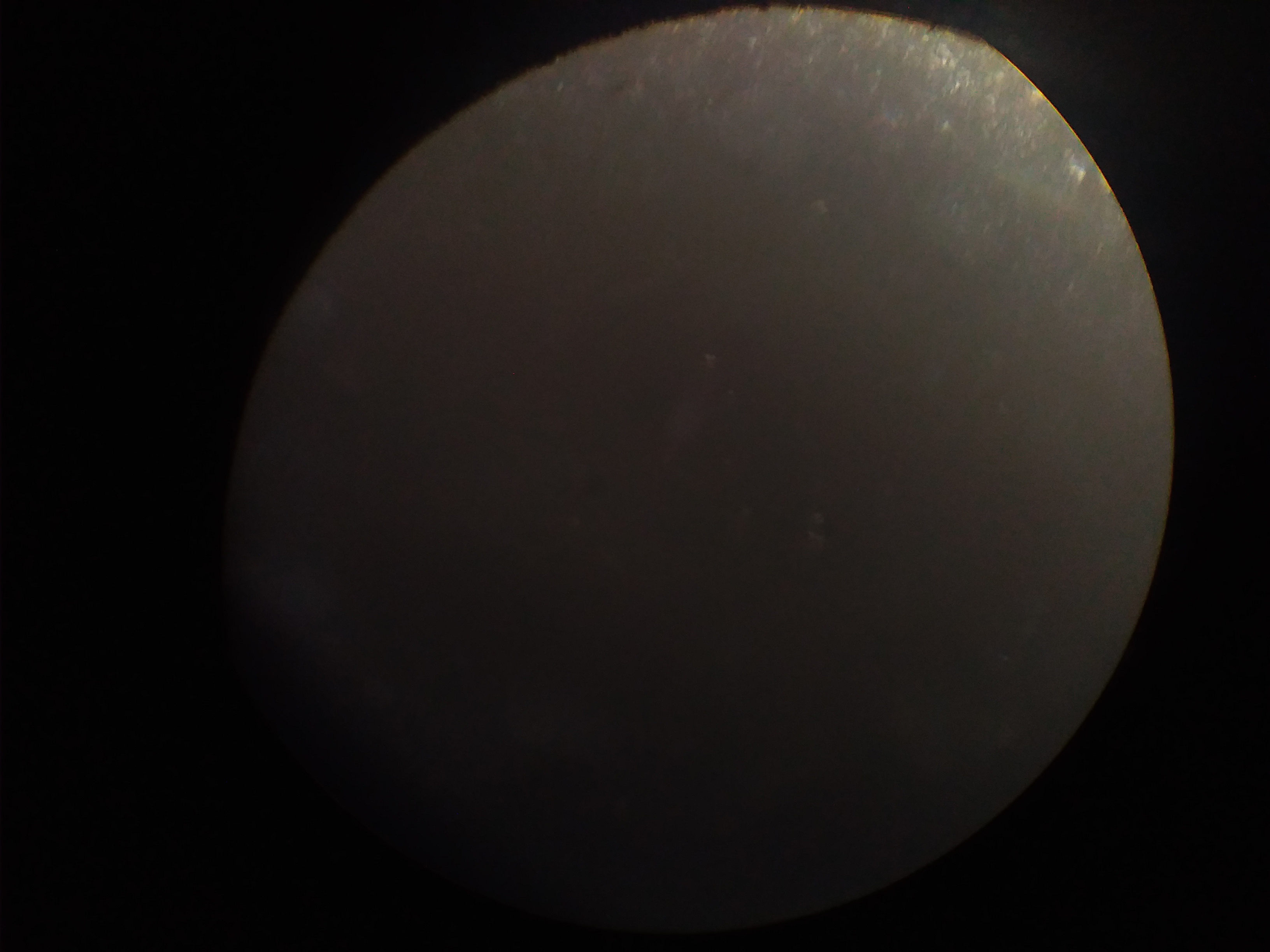

Supplement: Supplementary file 2 — Supplementary Information 2. [file 41598_2023_36721_MOESM2_ESM.zip › Raw data/Culture photos/20210609_175945.jpg]

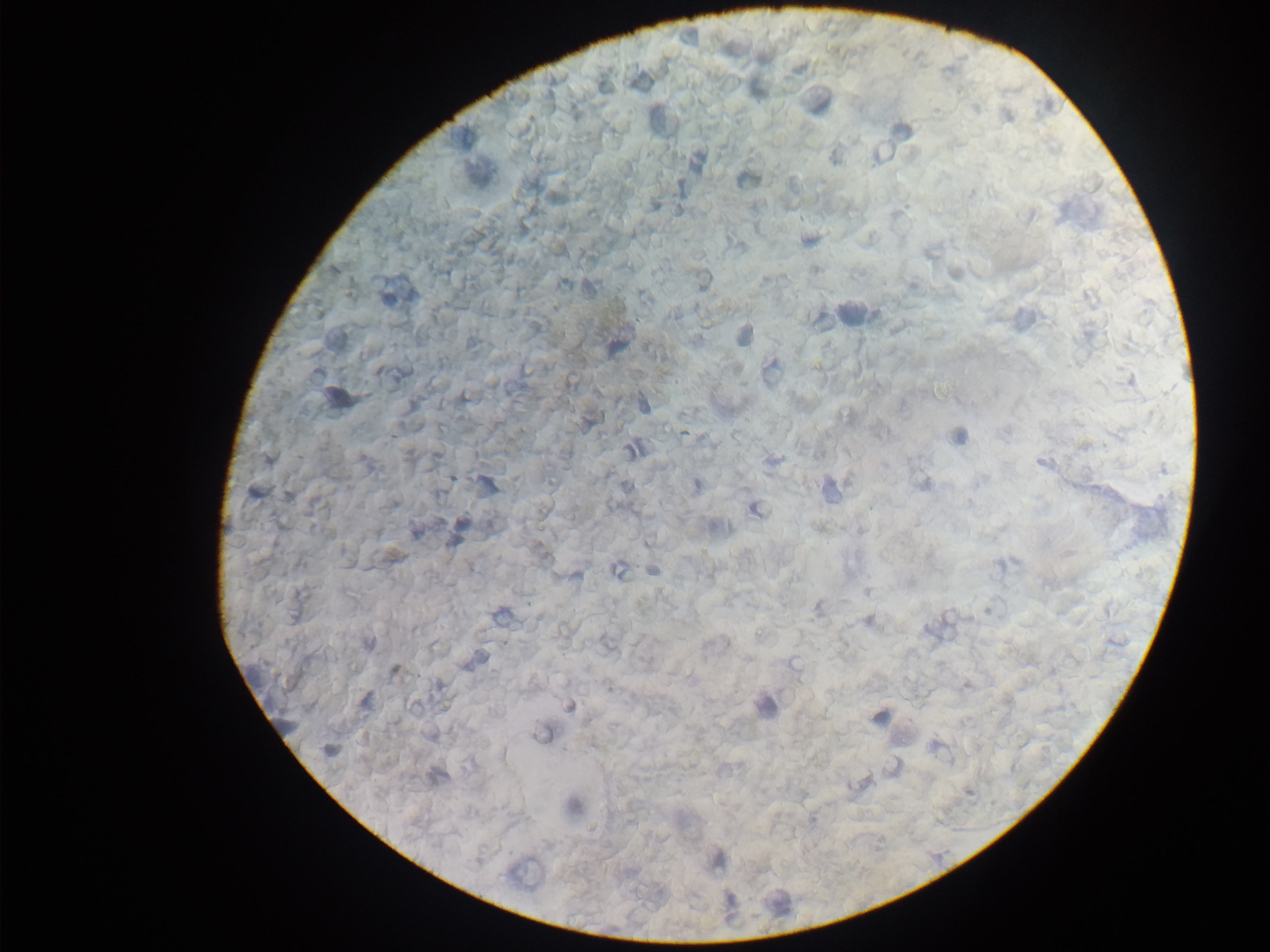

Supplement: Supplementary file 2 — Supplementary Information 2. [file 41598_2023_36721_MOESM2_ESM.zip › Raw data/Culture photos/20210609_175953.jpg]

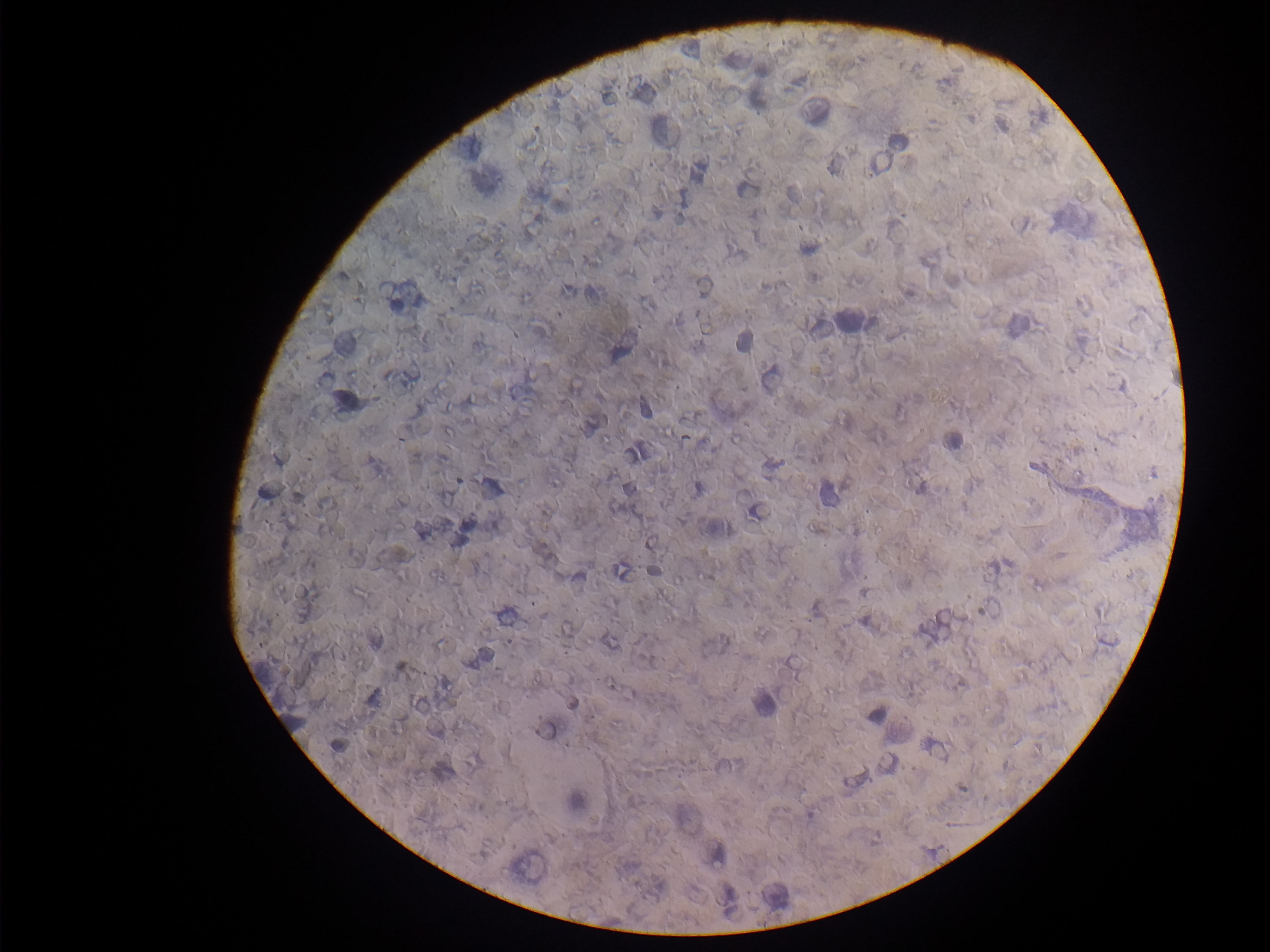

Supplement: Supplementary file 2 — Supplementary Information 2. [file 41598_2023_36721_MOESM2_ESM.zip › Raw data/Culture photos/20210609_175956.jpg]

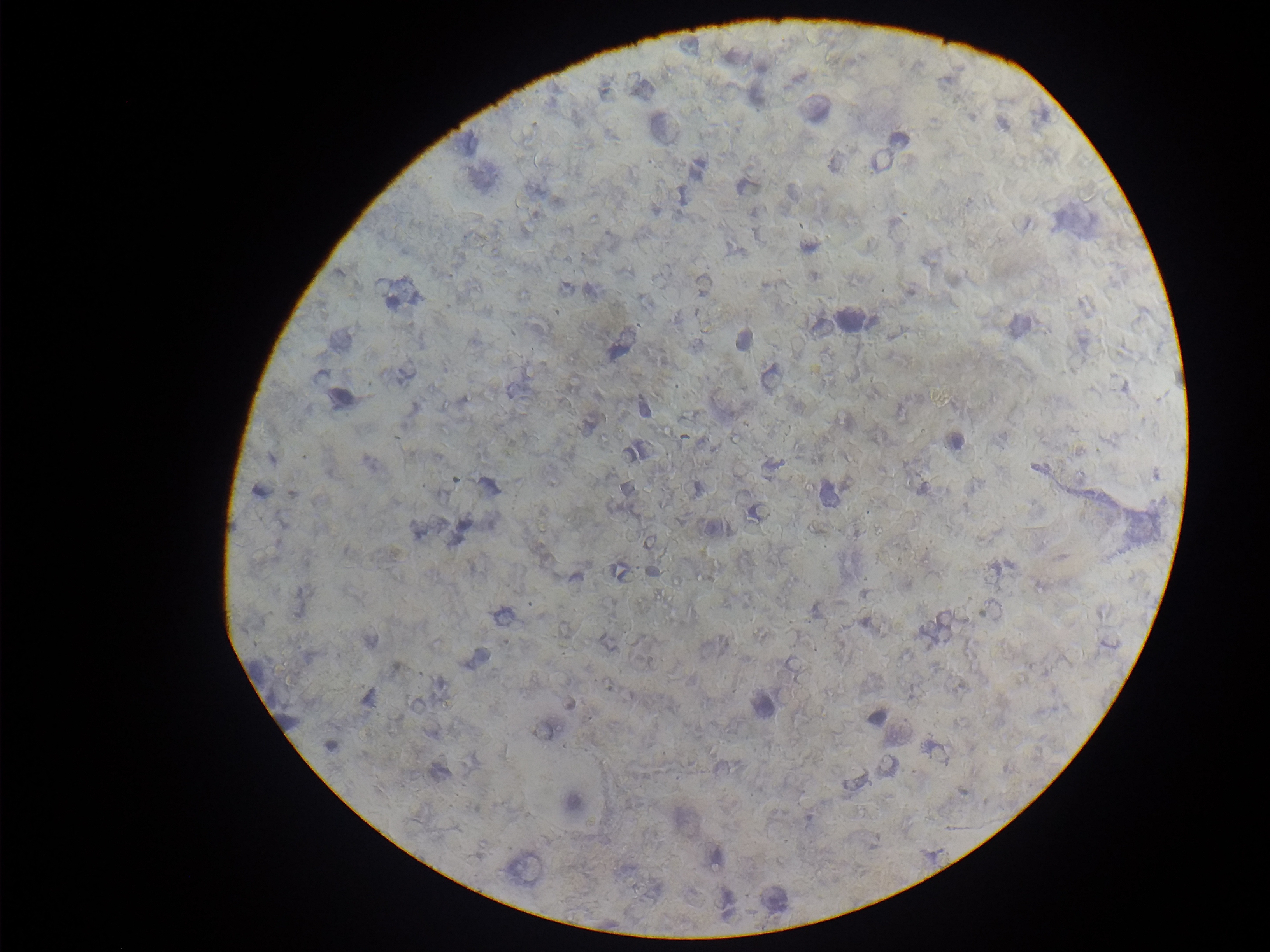

Supplement: Supplementary file 2 — Supplementary Information 2. [file 41598_2023_36721_MOESM2_ESM.zip › Raw data/Culture photos/20210609_175958.jpg]

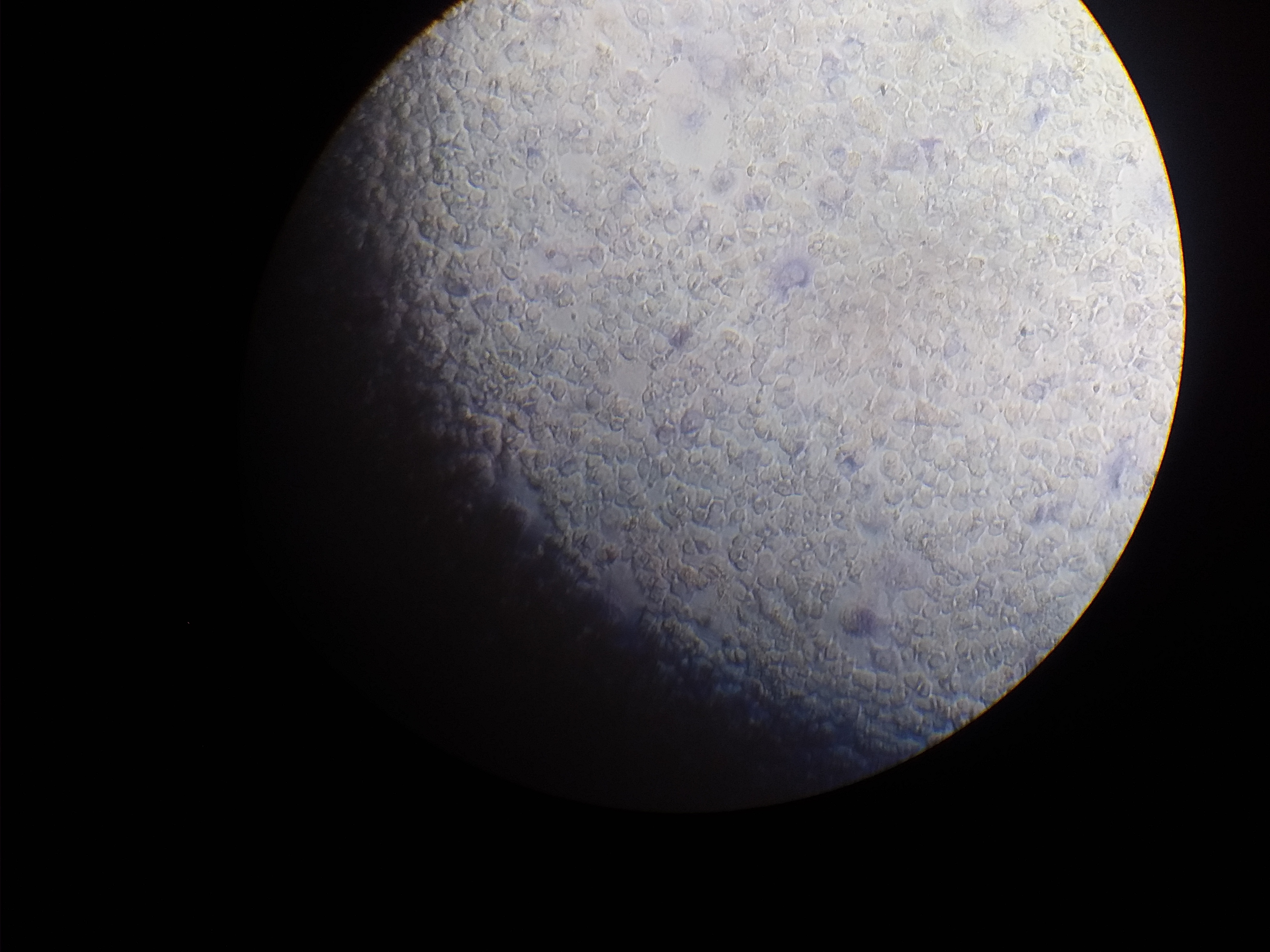

Supplement: Supplementary file 2 — Supplementary Information 2. [file 41598_2023_36721_MOESM2_ESM.zip › Raw data/Culture photos/20210609_180017.jpg]

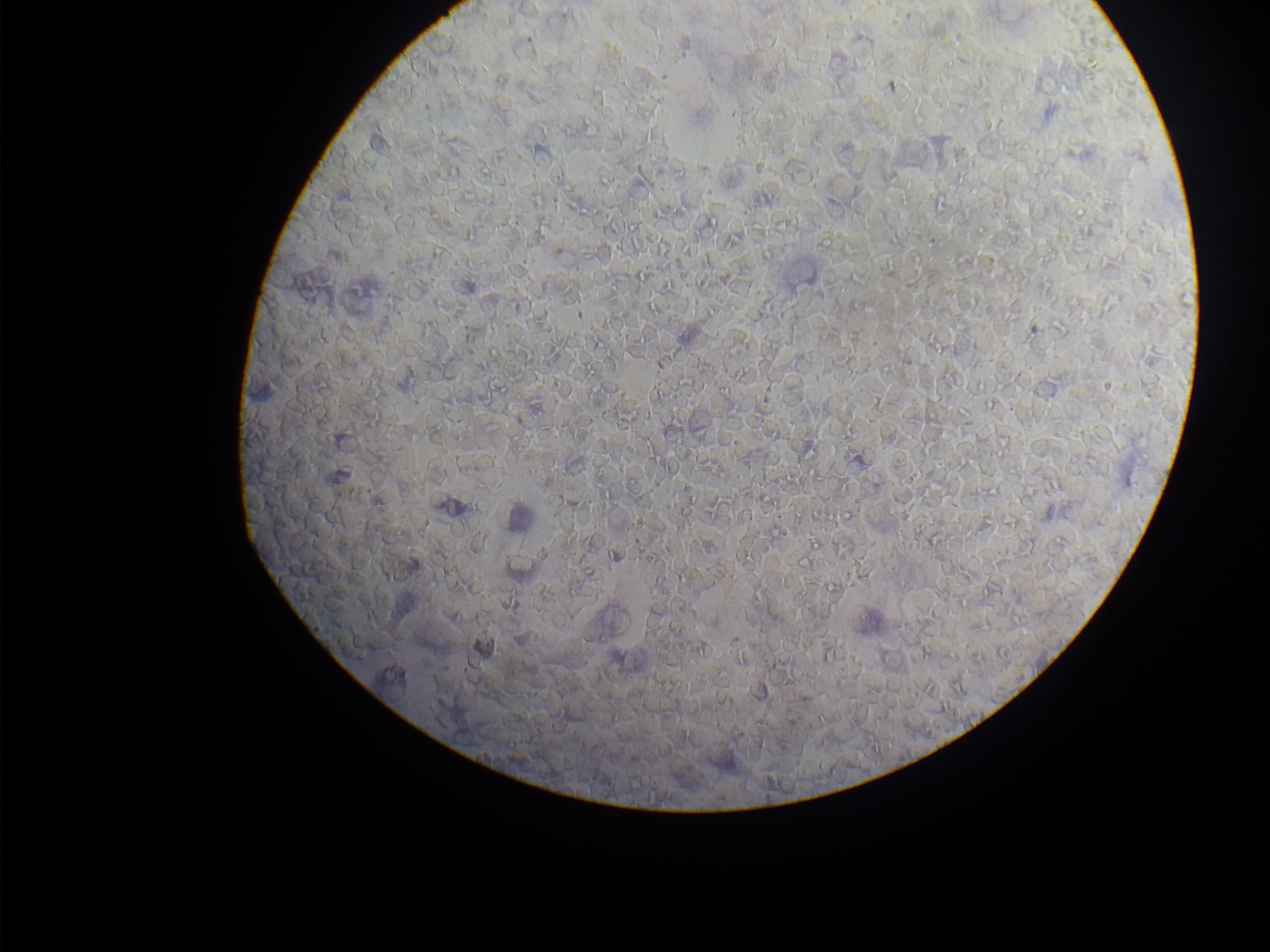

Supplement: Supplementary file 2 — Supplementary Information 2. [file 41598_2023_36721_MOESM2_ESM.zip › Raw data/Culture photos/20210609_180019.jpg]

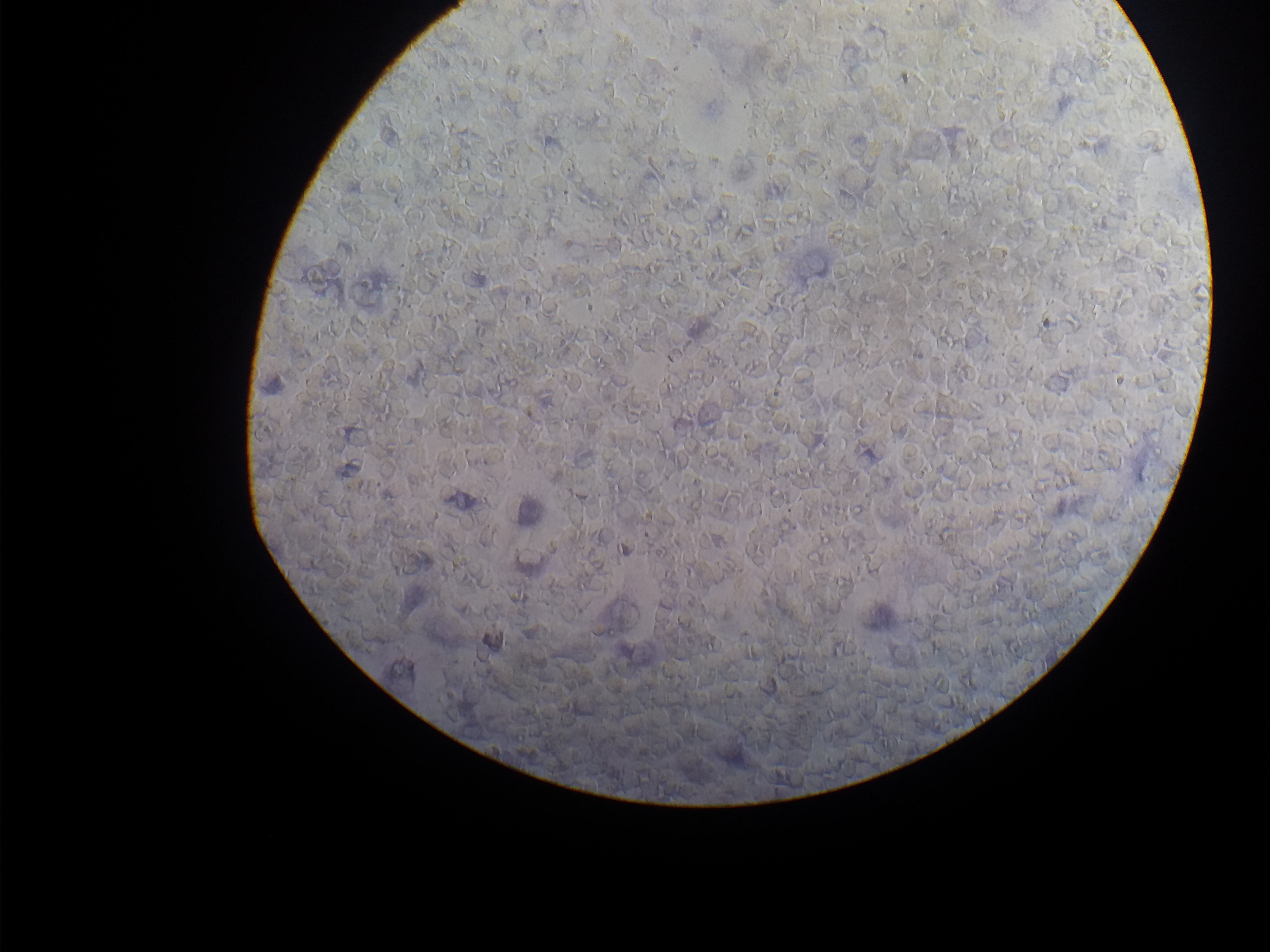

Supplement: Supplementary file 2 — Supplementary Information 2. [file 41598_2023_36721_MOESM2_ESM.zip › Raw data/Culture photos/20210609_180021.jpg]

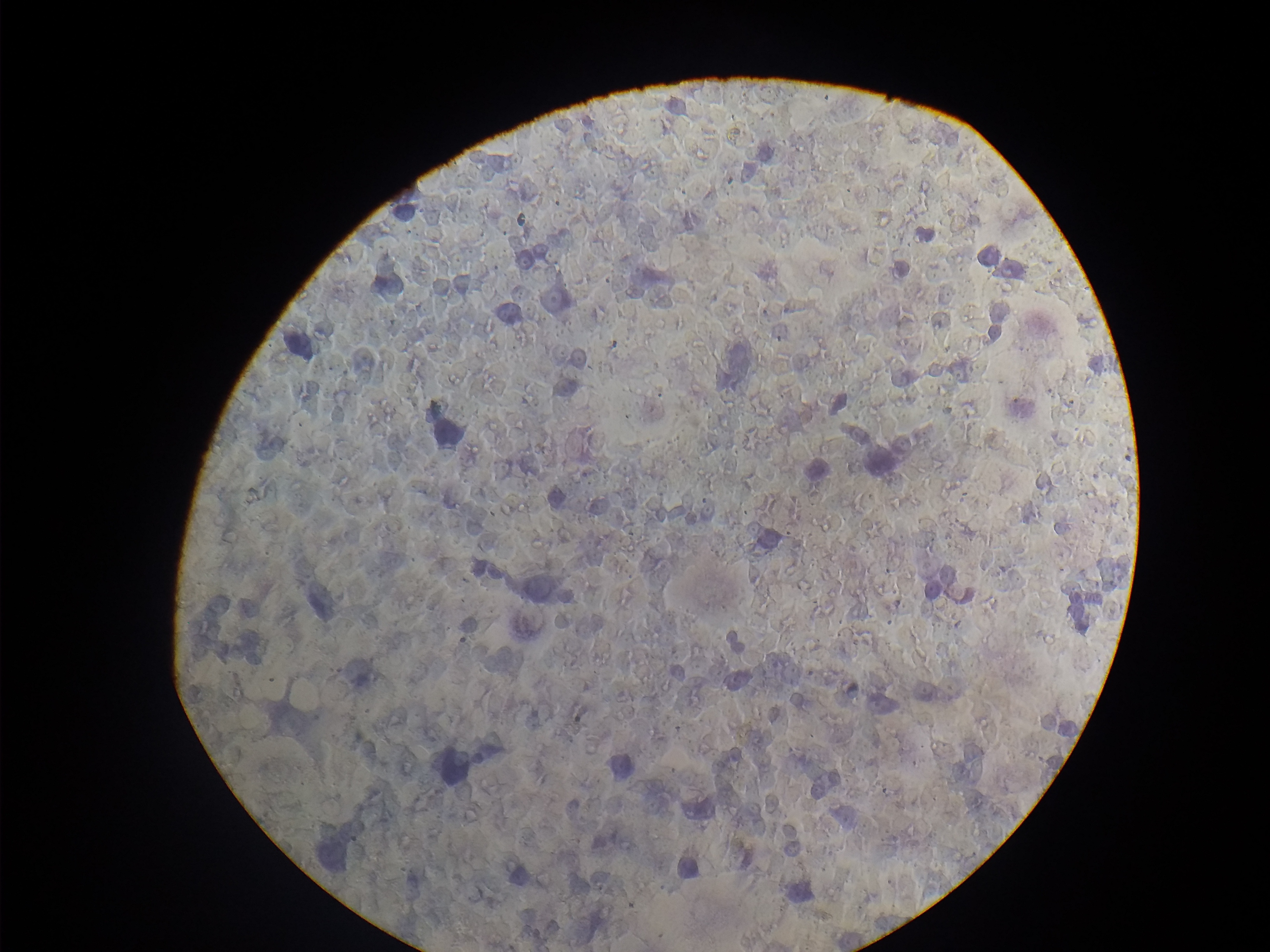

Supplement: Supplementary file 2 — Supplementary Information 2. [file 41598_2023_36721_MOESM2_ESM.zip › Raw data/Culture photos/20210609_180043.jpg]

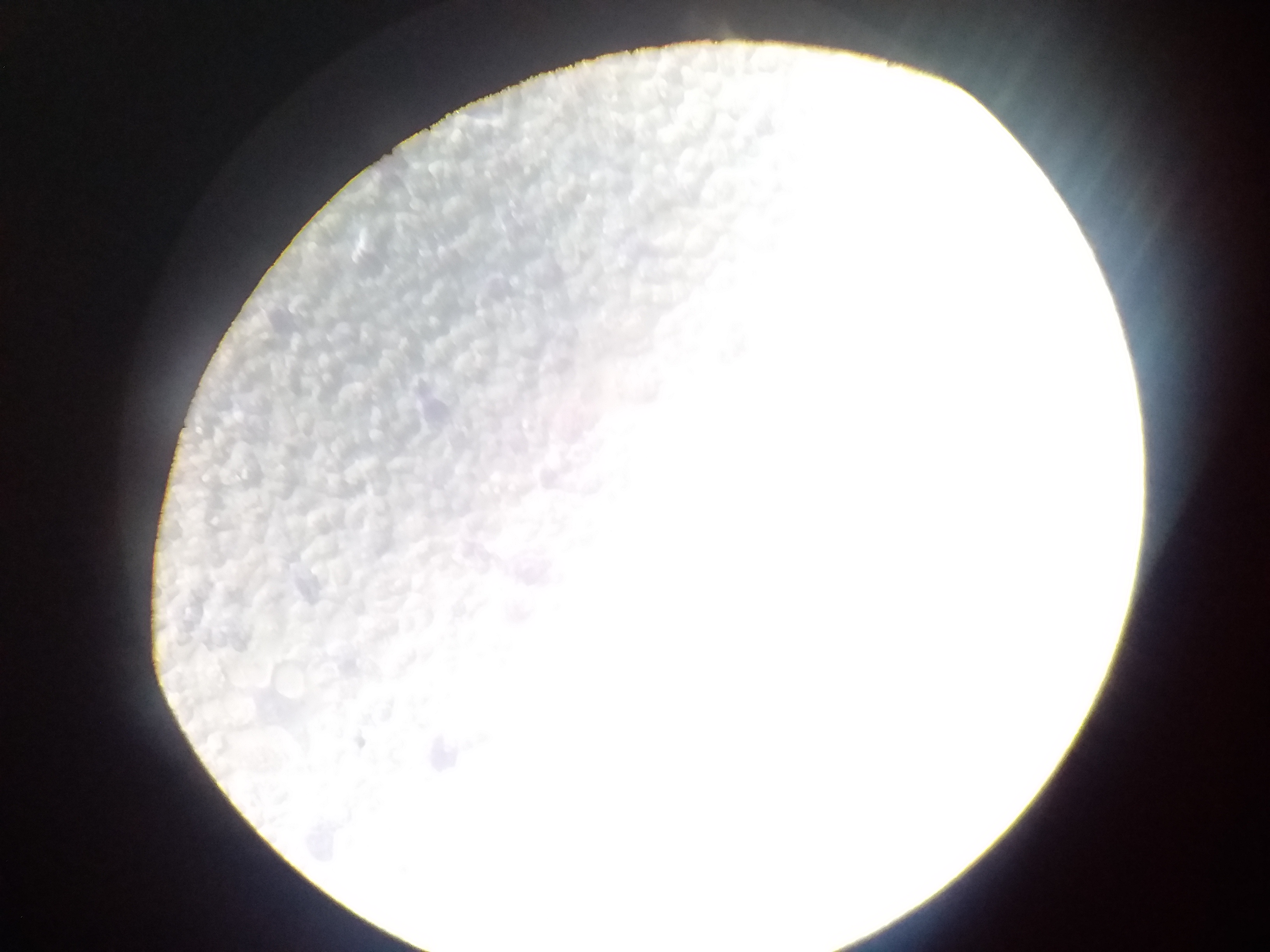

Supplement: Supplementary file 2 — Supplementary Information 2. [file 41598_2023_36721_MOESM2_ESM.zip › Raw data/Culture photos/20210609_180045.jpg]

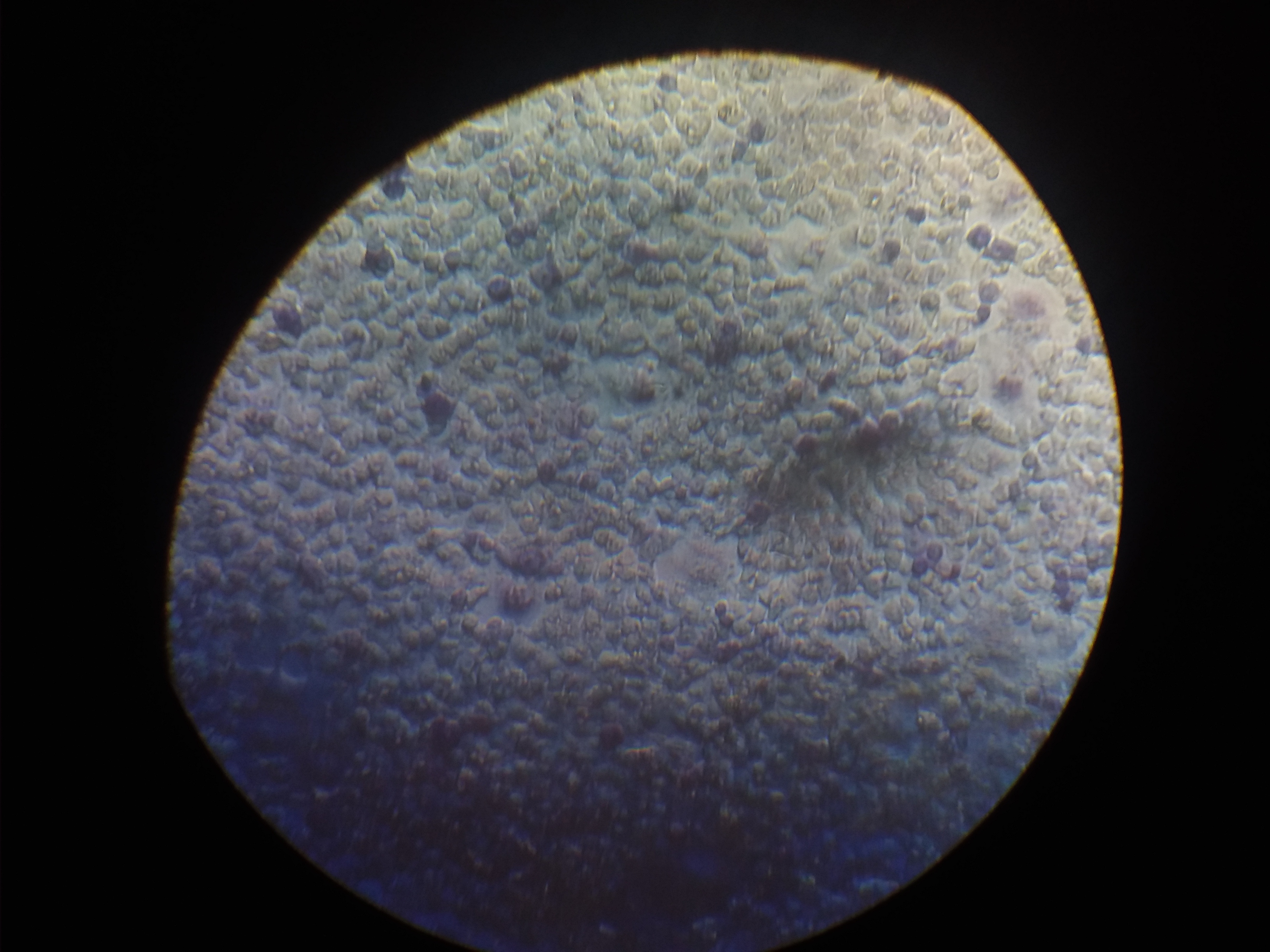

Supplement: Supplementary file 2 — Supplementary Information 2. [file 41598_2023_36721_MOESM2_ESM.zip › Raw data/Culture photos/20210609_180052.jpg]

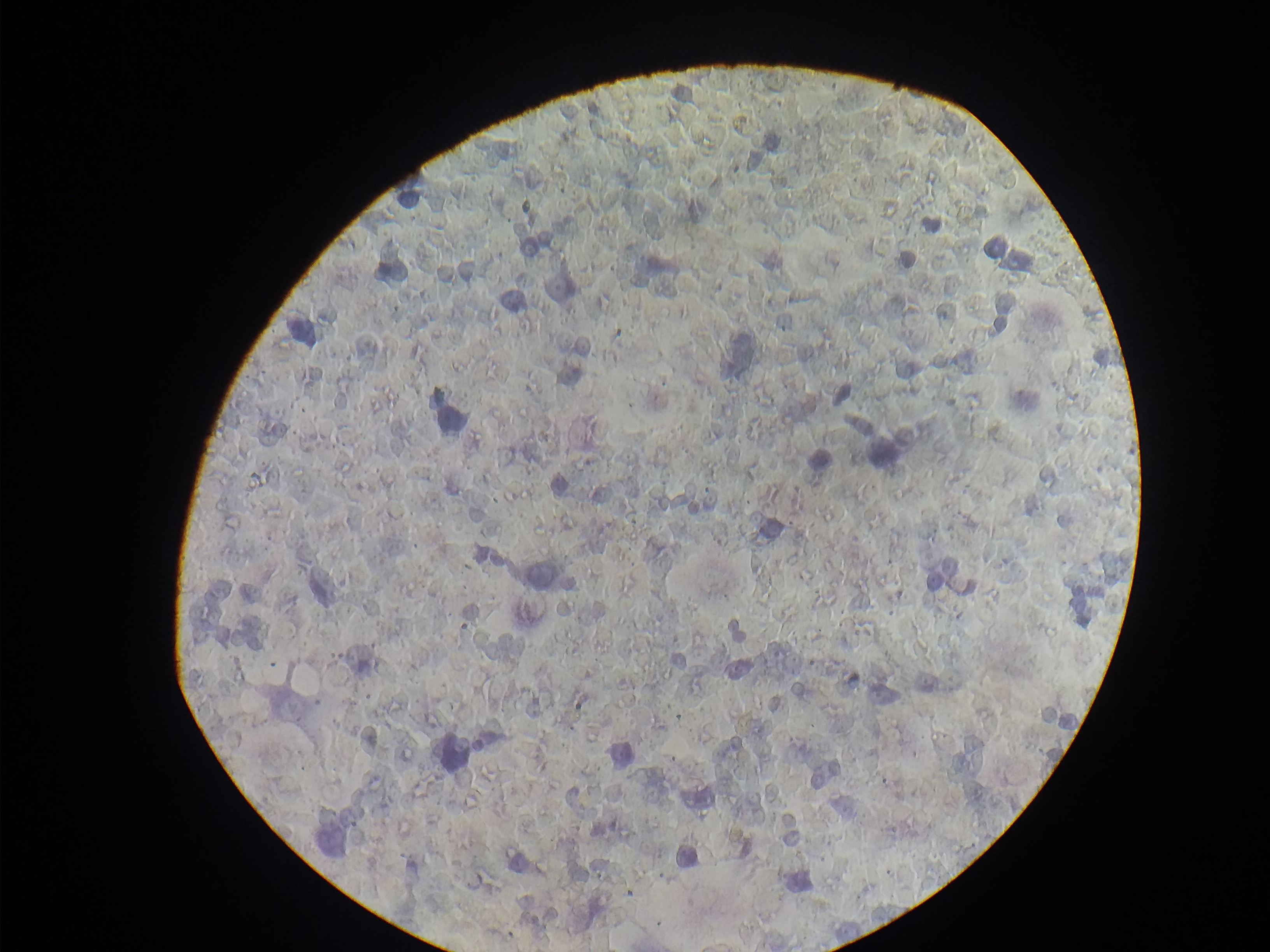

Supplement: Supplementary file 2 — Supplementary Information 2. [file 41598_2023_36721_MOESM2_ESM.zip › Raw data/Culture photos/20210609_180057.jpg]

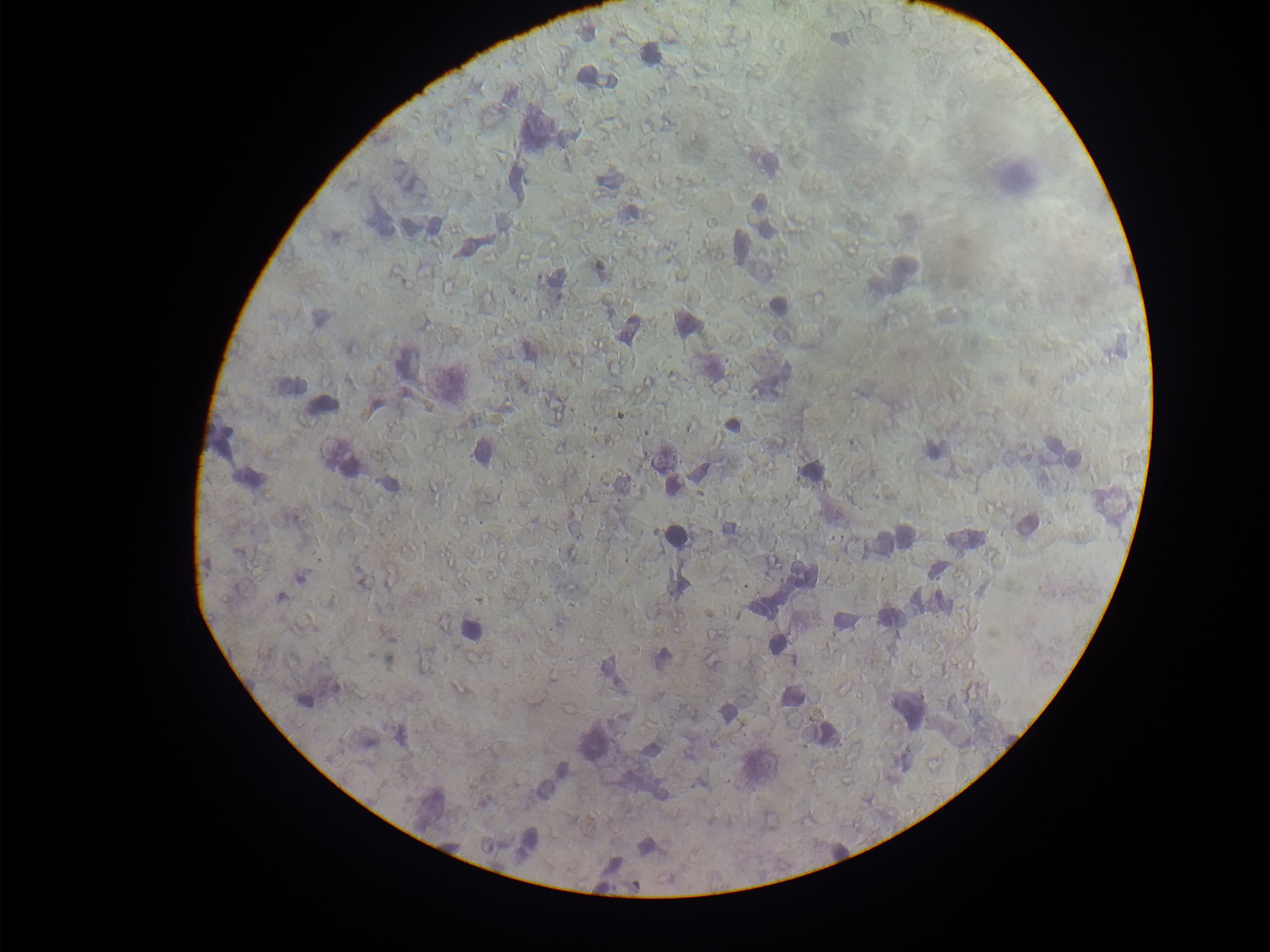

Supplement: Supplementary file 2 — Supplementary Information 2. [file 41598_2023_36721_MOESM2_ESM.zip › Raw data/Culture photos/20210609_180121.jpg]

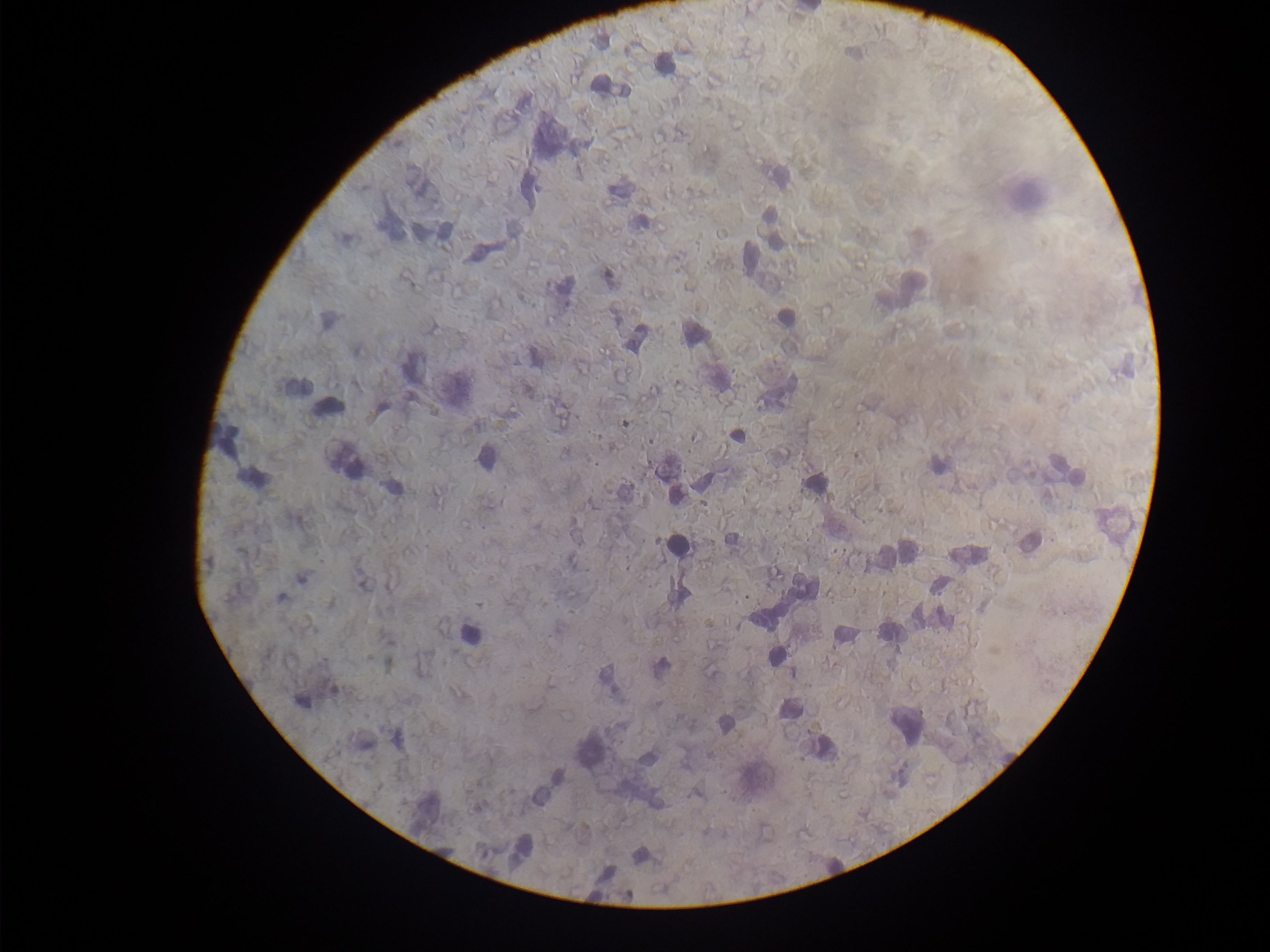

Supplement: Supplementary file 2 — Supplementary Information 2. [file 41598_2023_36721_MOESM2_ESM.zip › Raw data/Culture photos/20210609_180134.jpg]

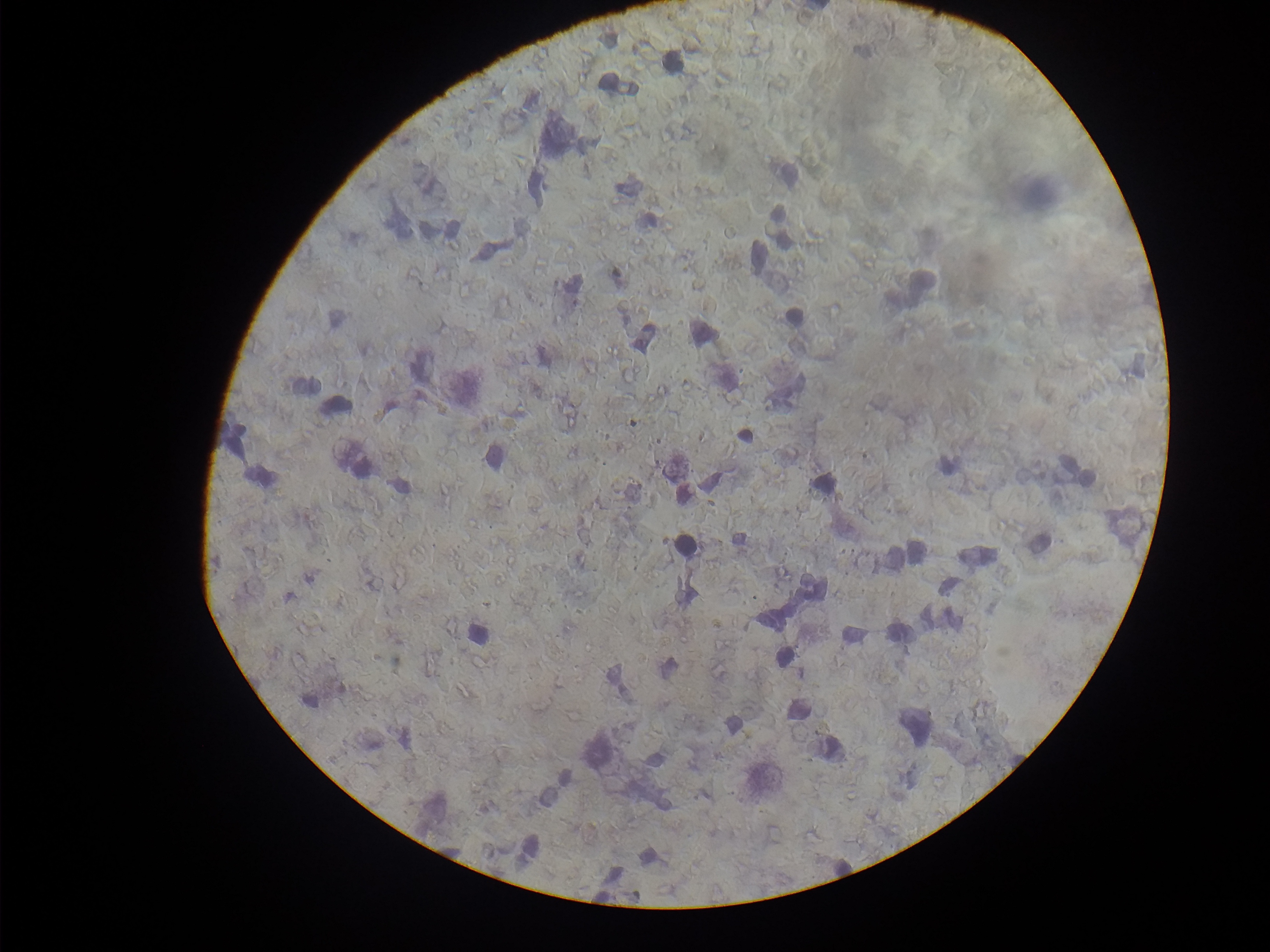

Supplement: Supplementary file 2 — Supplementary Information 2. [file 41598_2023_36721_MOESM2_ESM.zip › Raw data/Culture photos/20210609_180135.jpg]

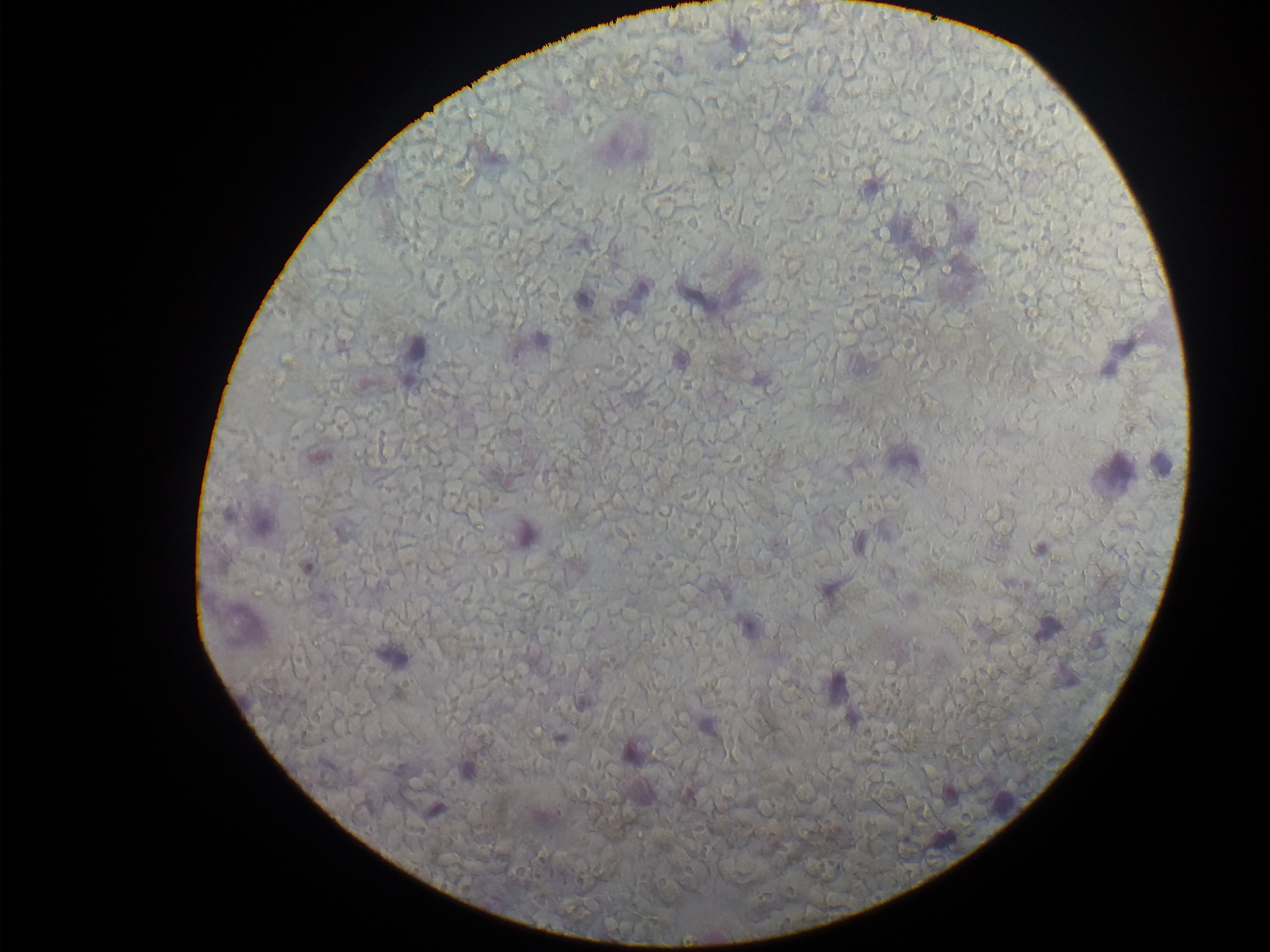

Supplement: Supplementary file 2 — Supplementary Information 2. [file 41598_2023_36721_MOESM2_ESM.zip › Raw data/Culture photos/20210609_180154.jpg]

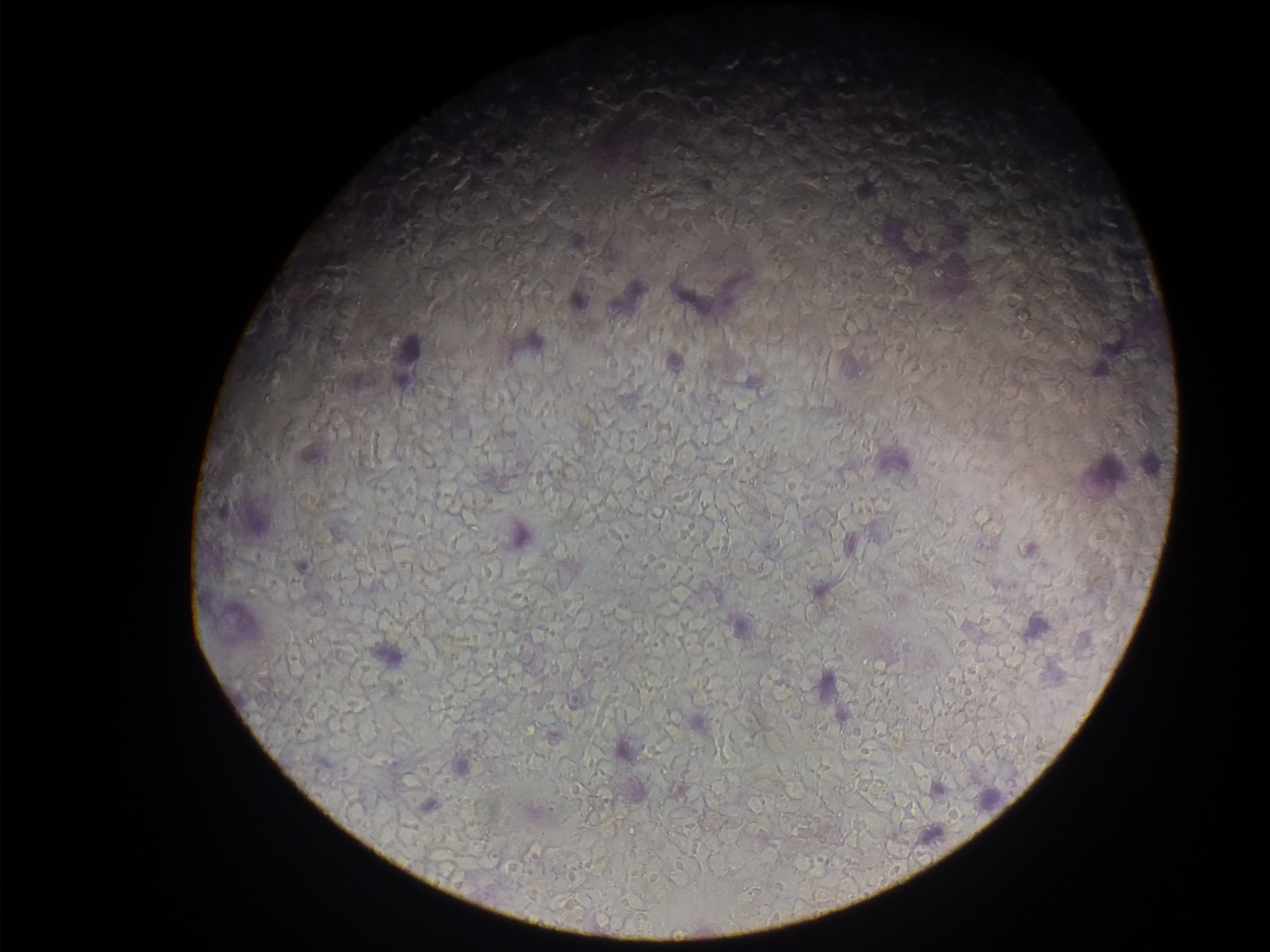

Supplement: Supplementary file 2 — Supplementary Information 2. [file 41598_2023_36721_MOESM2_ESM.zip › Raw data/Culture photos/20210609_180156.jpg]

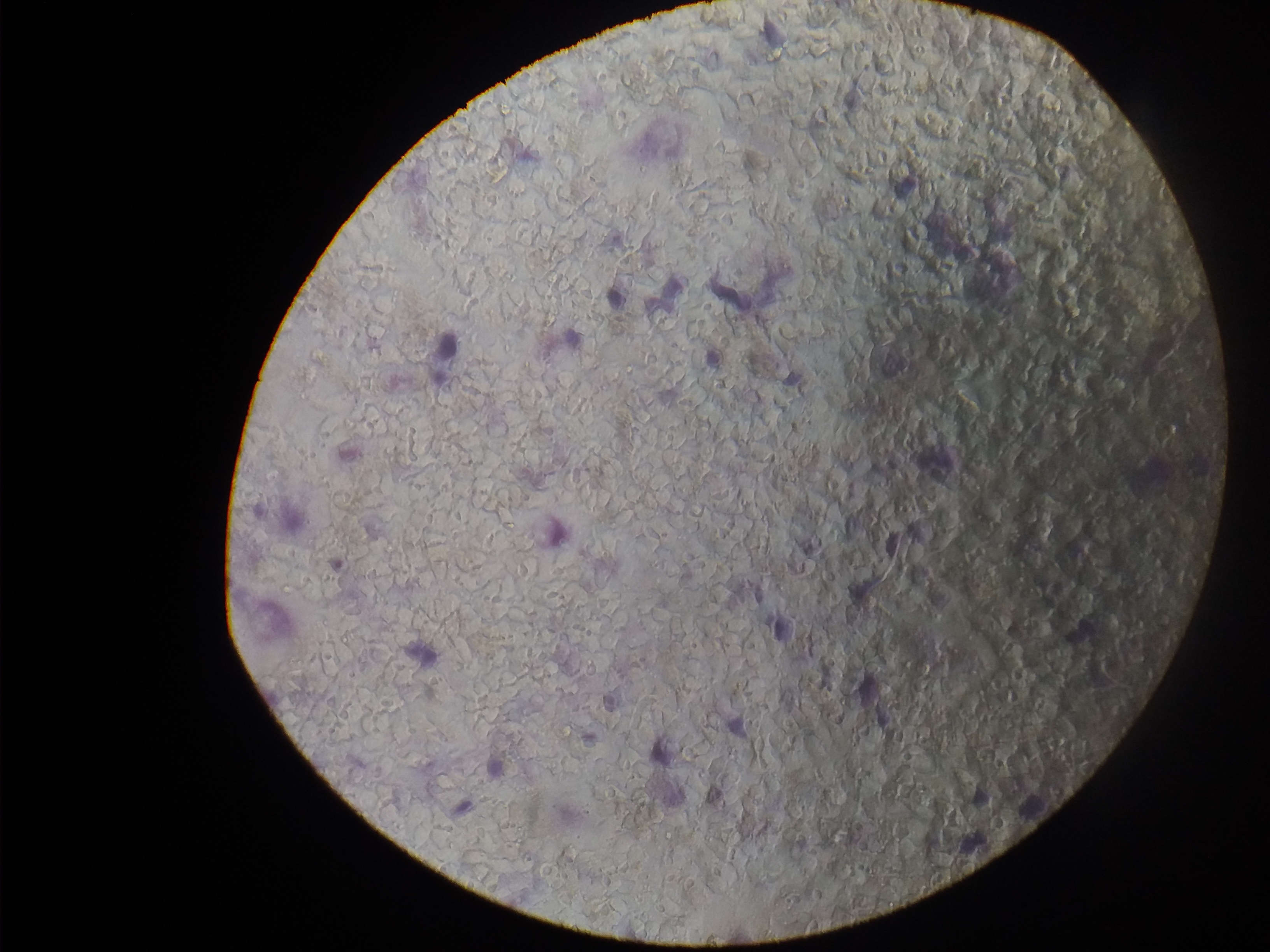

Supplement: Supplementary file 2 — Supplementary Information 2. [file 41598_2023_36721_MOESM2_ESM.zip › Raw data/Culture photos/20210609_180201.jpg]

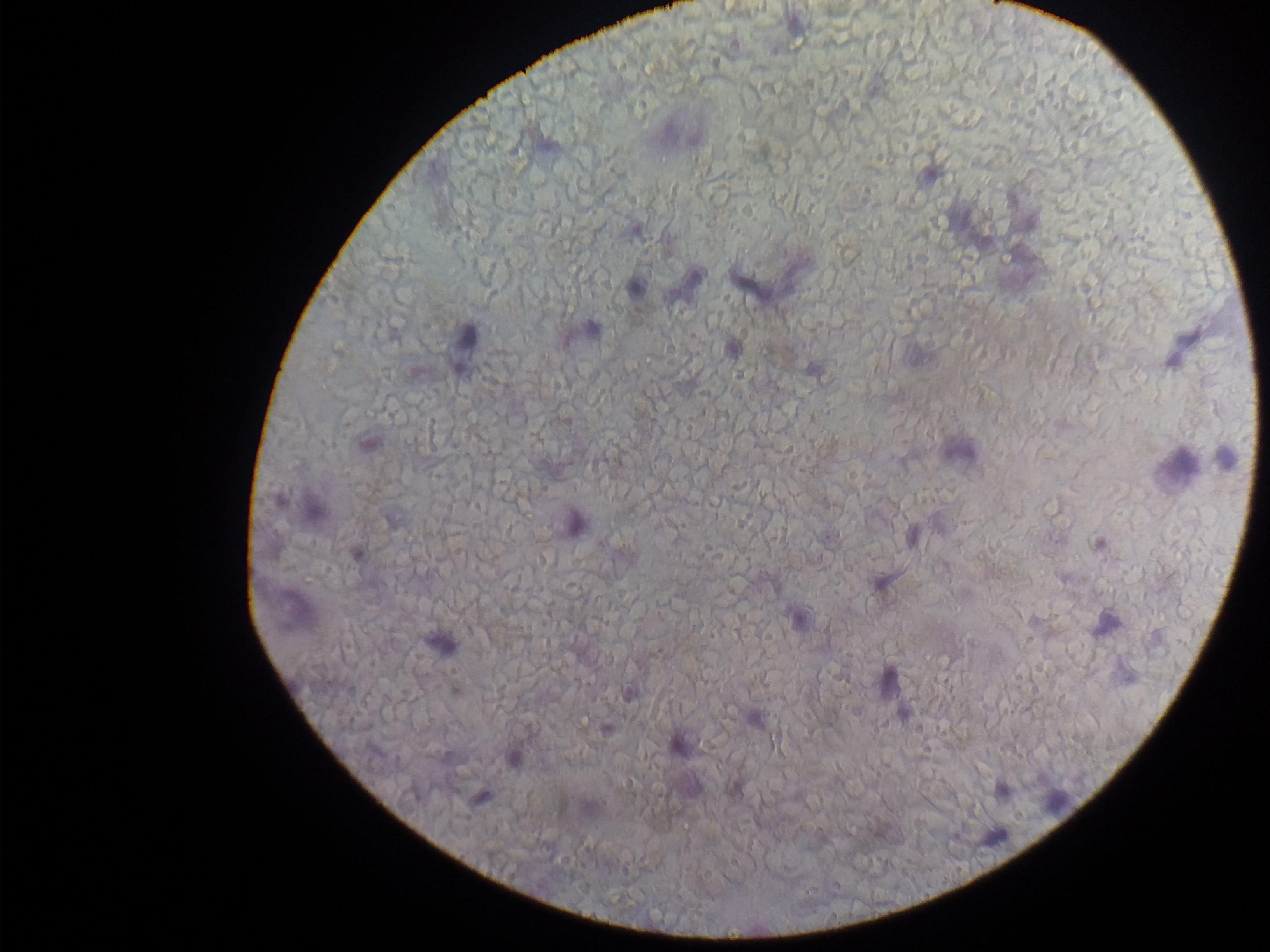

Supplement: Supplementary file 2 — Supplementary Information 2. [file 41598_2023_36721_MOESM2_ESM.zip › Raw data/Culture photos/20210609_180207.jpg]

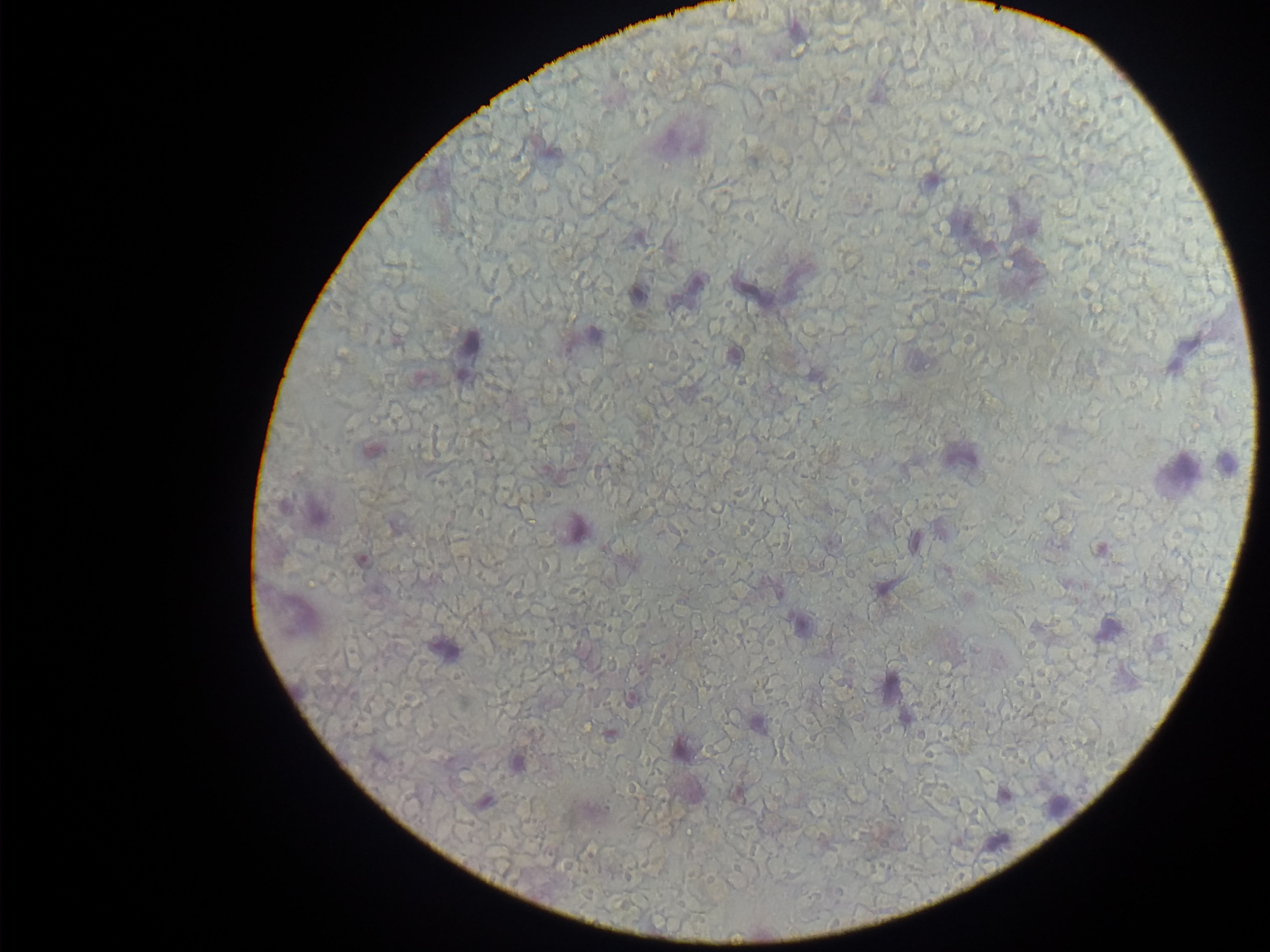

Supplement: Supplementary file 2 — Supplementary Information 2. [file 41598_2023_36721_MOESM2_ESM.zip › Raw data/Culture photos/20210609_180208.jpg]

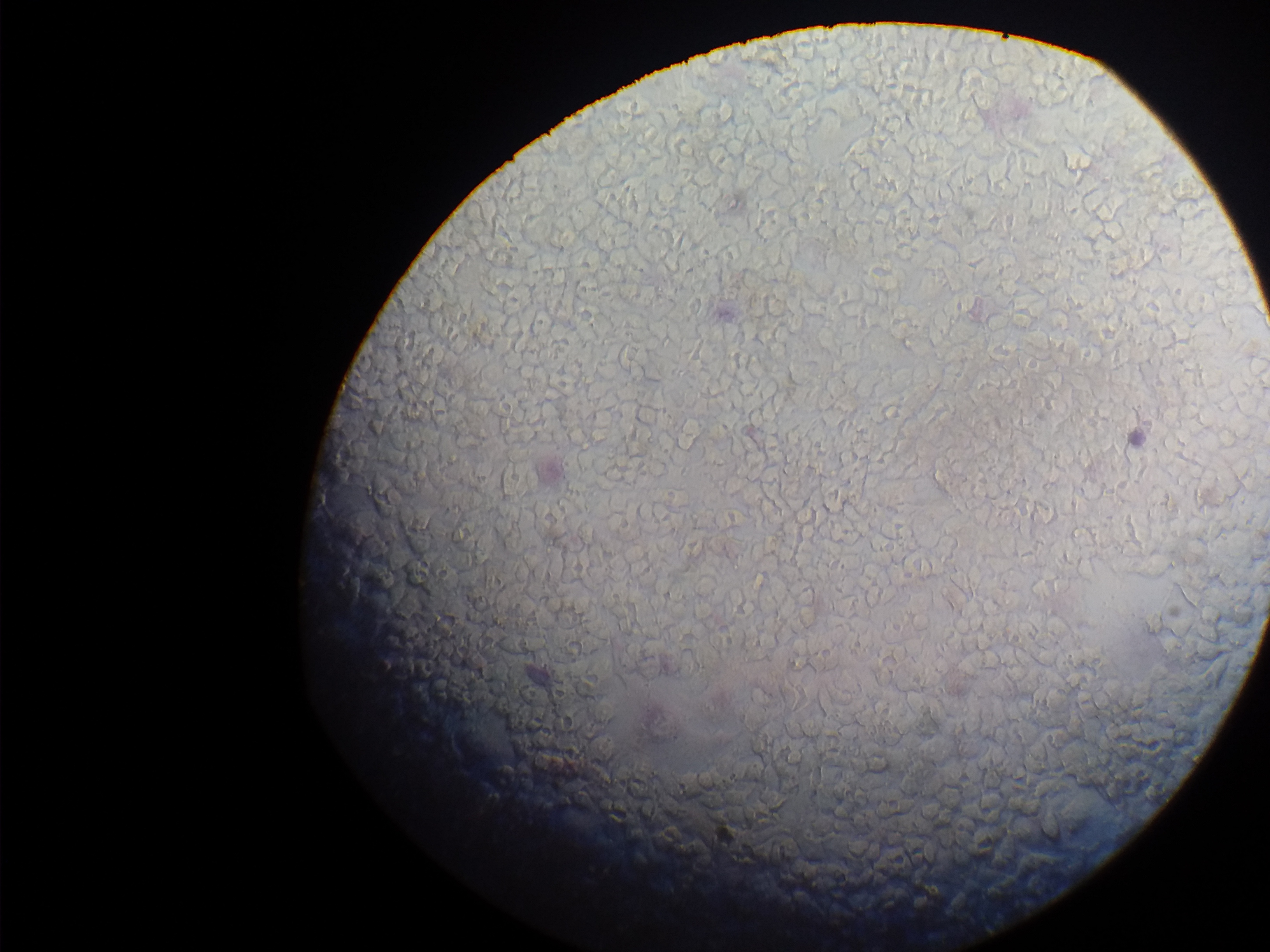

Supplement: Supplementary file 2 — Supplementary Information 2. [file 41598_2023_36721_MOESM2_ESM.zip › Raw data/Culture photos/20210609_180249.jpg]

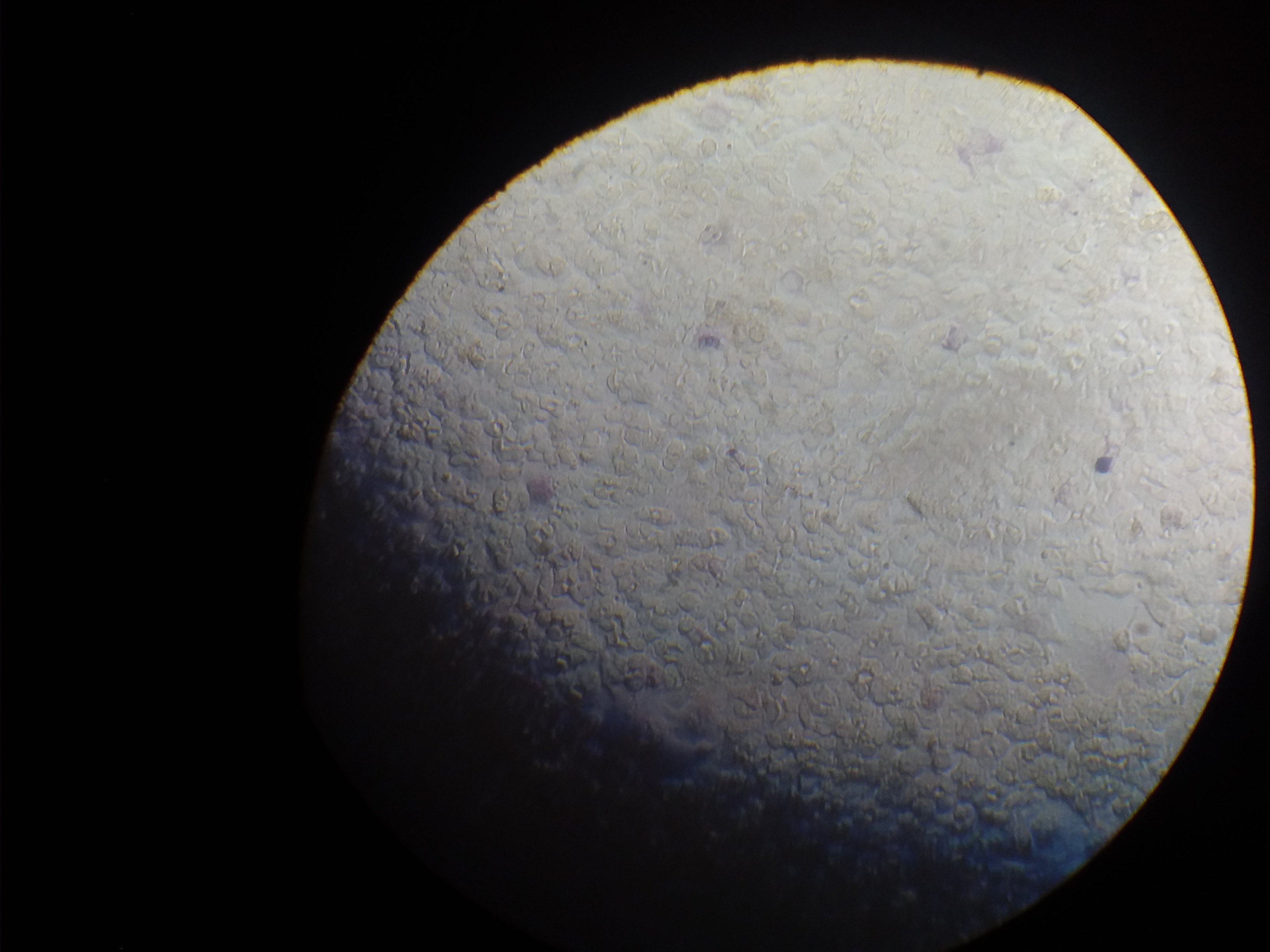

Supplement: Supplementary file 2 — Supplementary Information 2. [file 41598_2023_36721_MOESM2_ESM.zip › Raw data/Culture photos/20210609_180254.jpg]

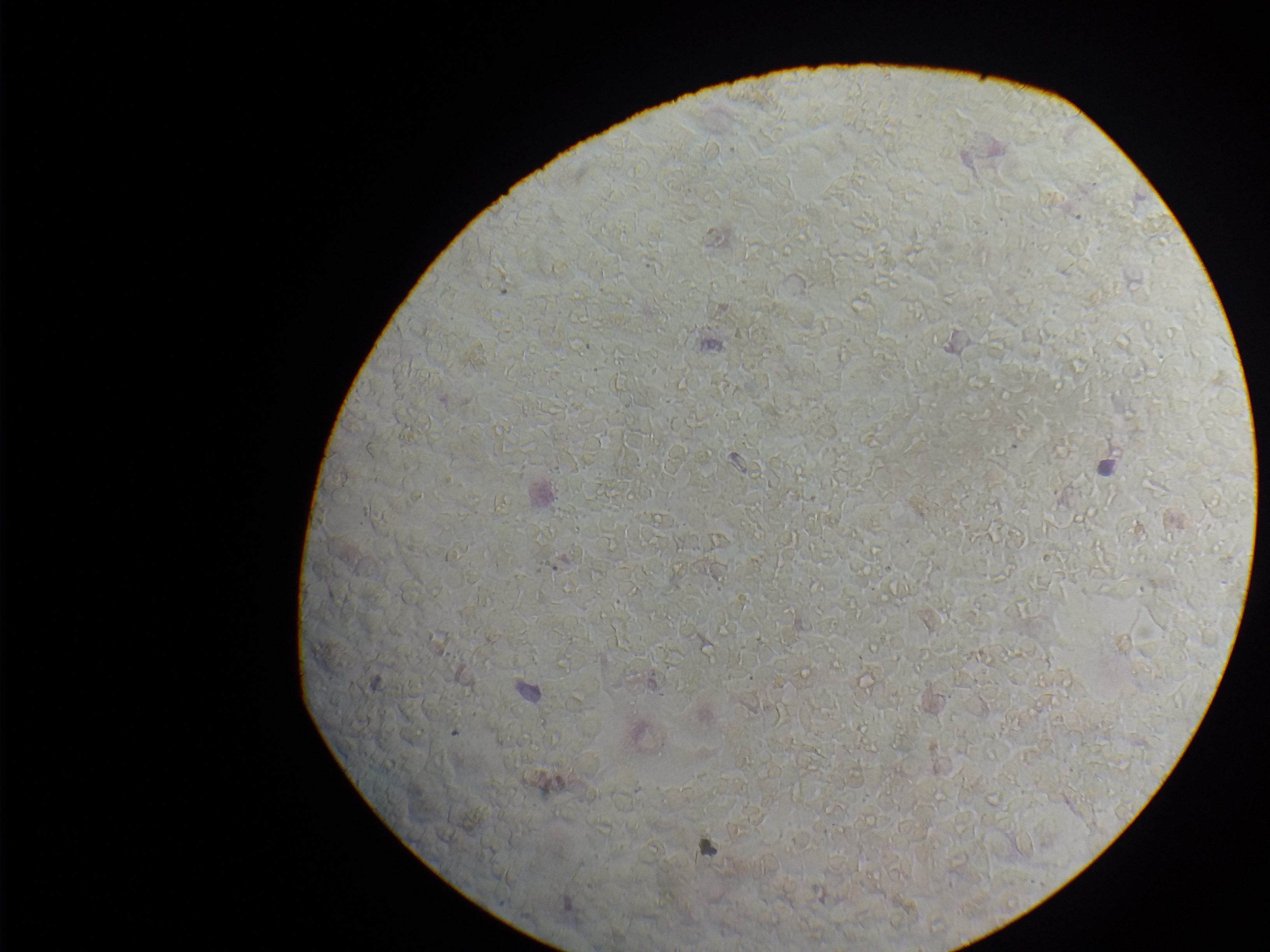

Supplement: Supplementary file 2 — Supplementary Information 2. [file 41598_2023_36721_MOESM2_ESM.zip › Raw data/Culture photos/20210609_180257.jpg]

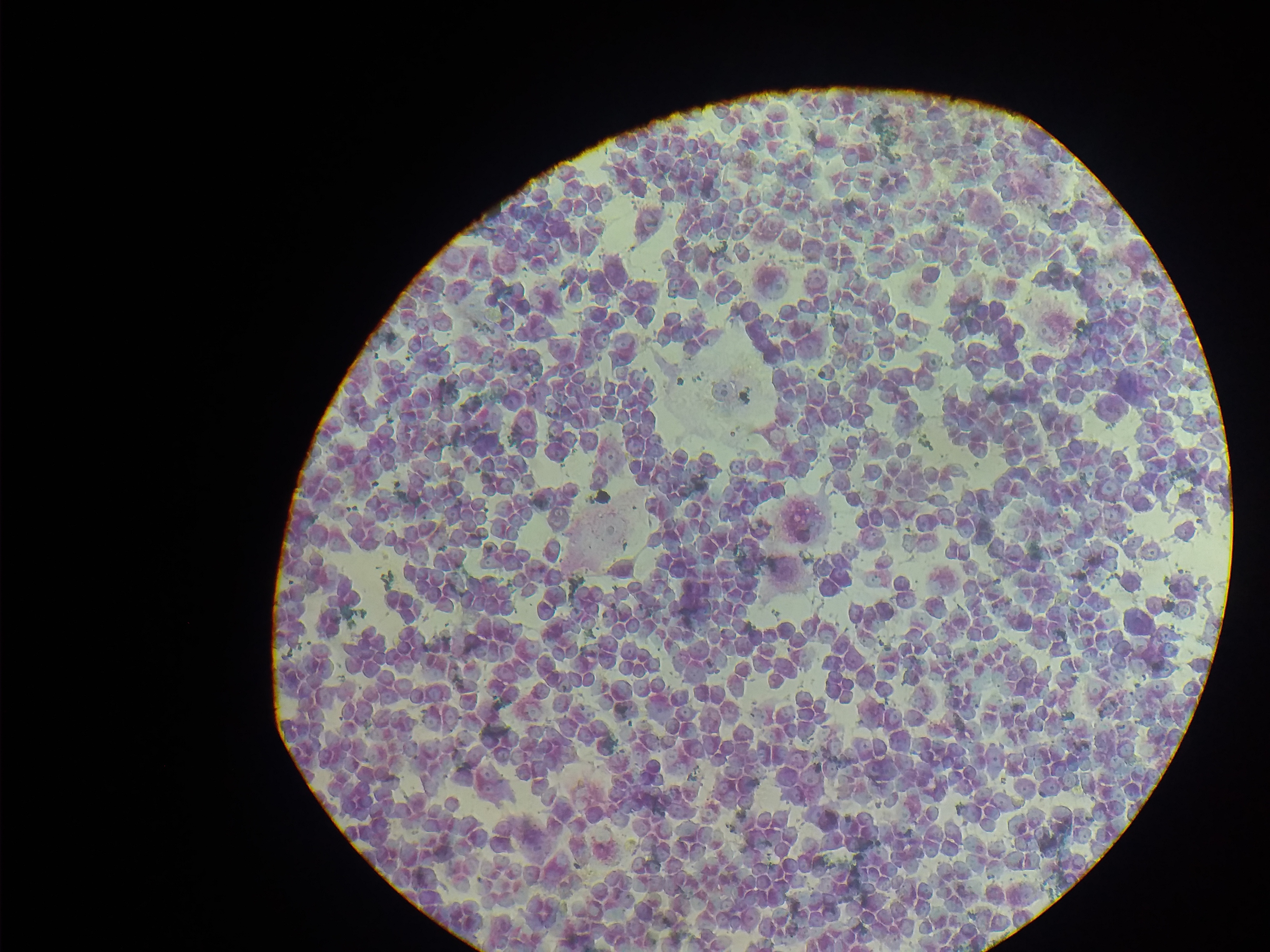

Supplement: Supplementary file 2 — Supplementary Information 2. [file 41598_2023_36721_MOESM2_ESM.zip › Raw data/Culture photos/20210609_180334.jpg]

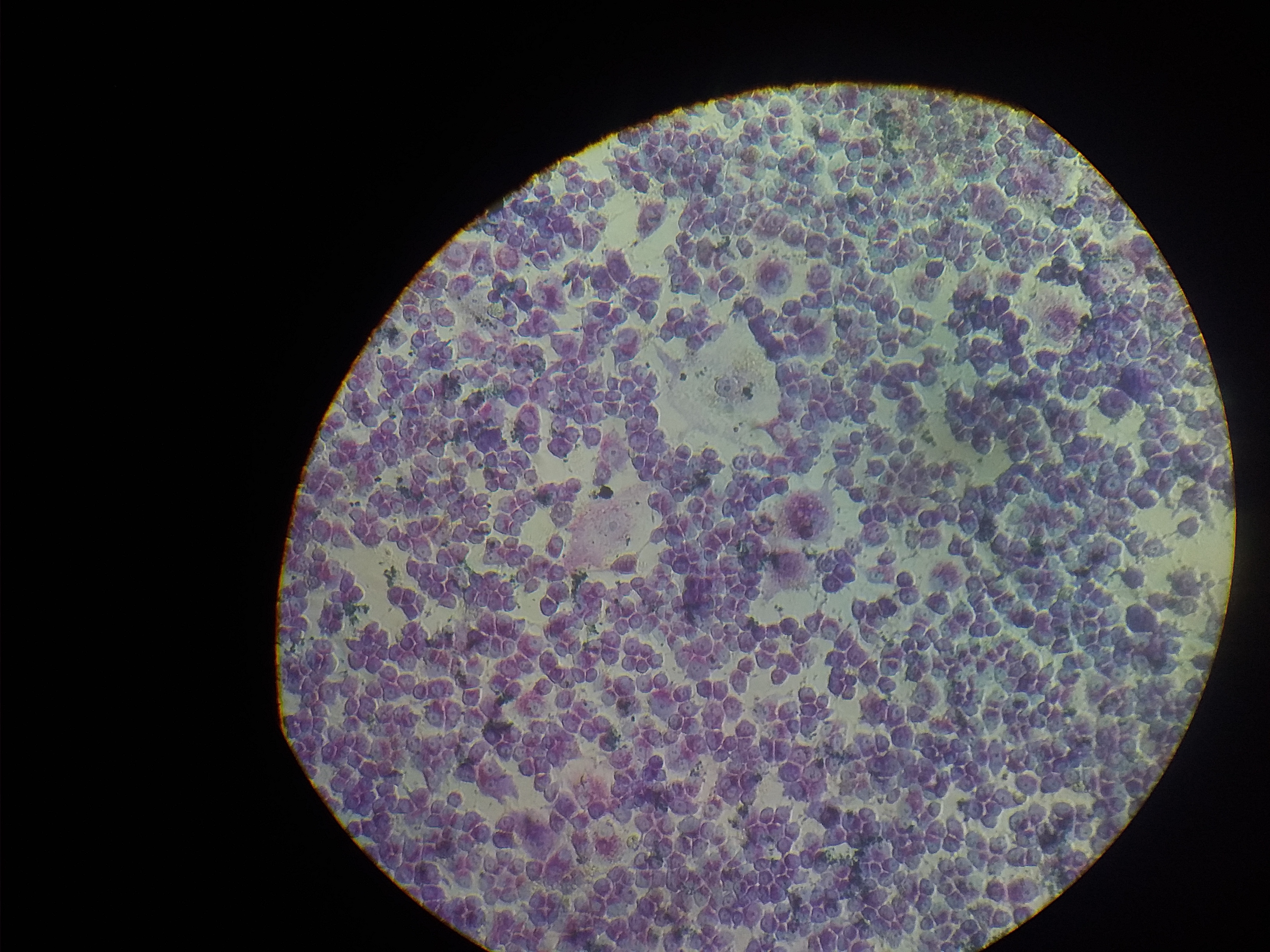

Supplement: Supplementary file 2 — Supplementary Information 2. [file 41598_2023_36721_MOESM2_ESM.zip › Raw data/Culture photos/20210609_180335.jpg]

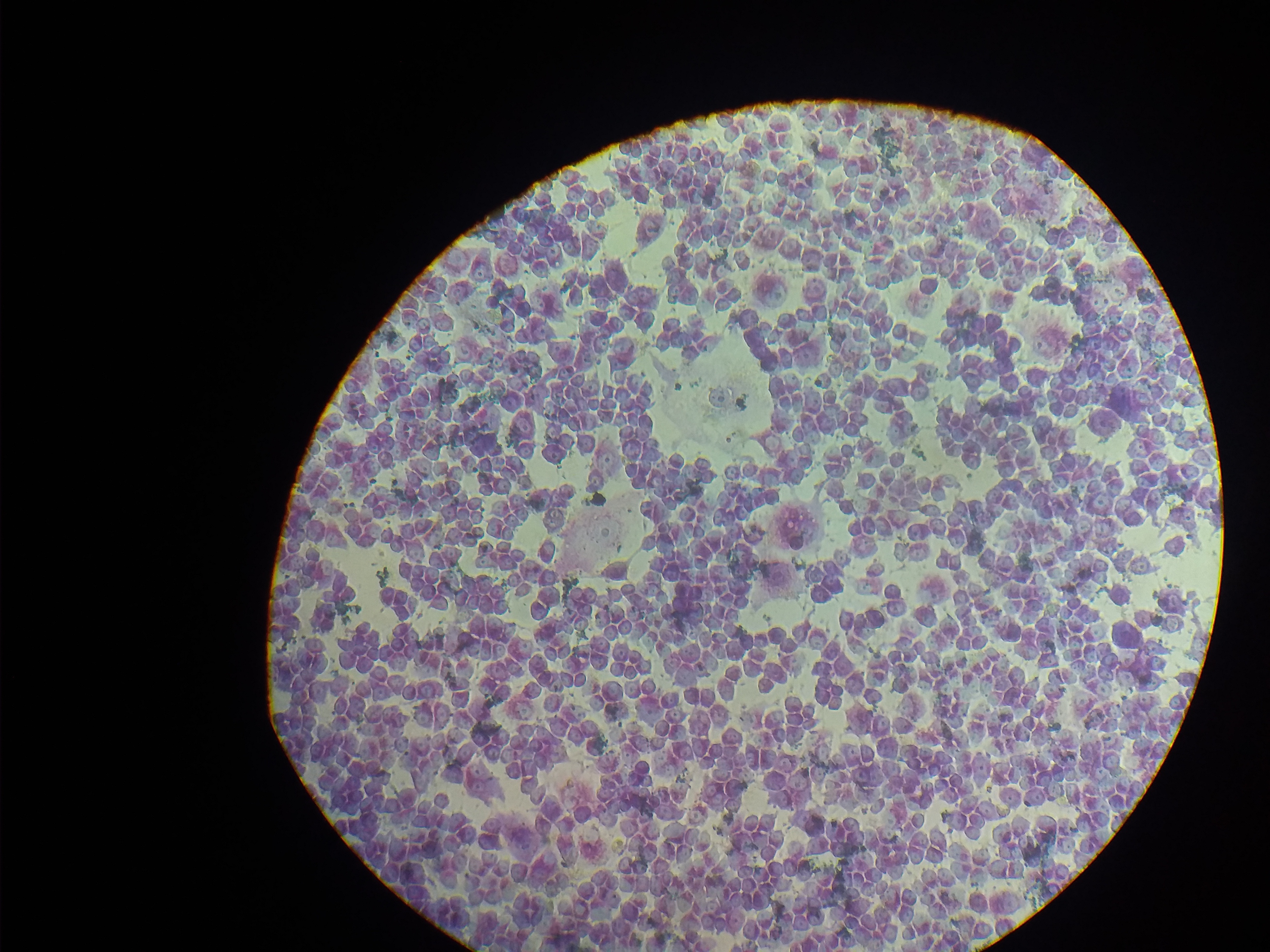

Supplement: Supplementary file 2 — Supplementary Information 2. [file 41598_2023_36721_MOESM2_ESM.zip › Raw data/Culture photos/20210609_180345.jpg]

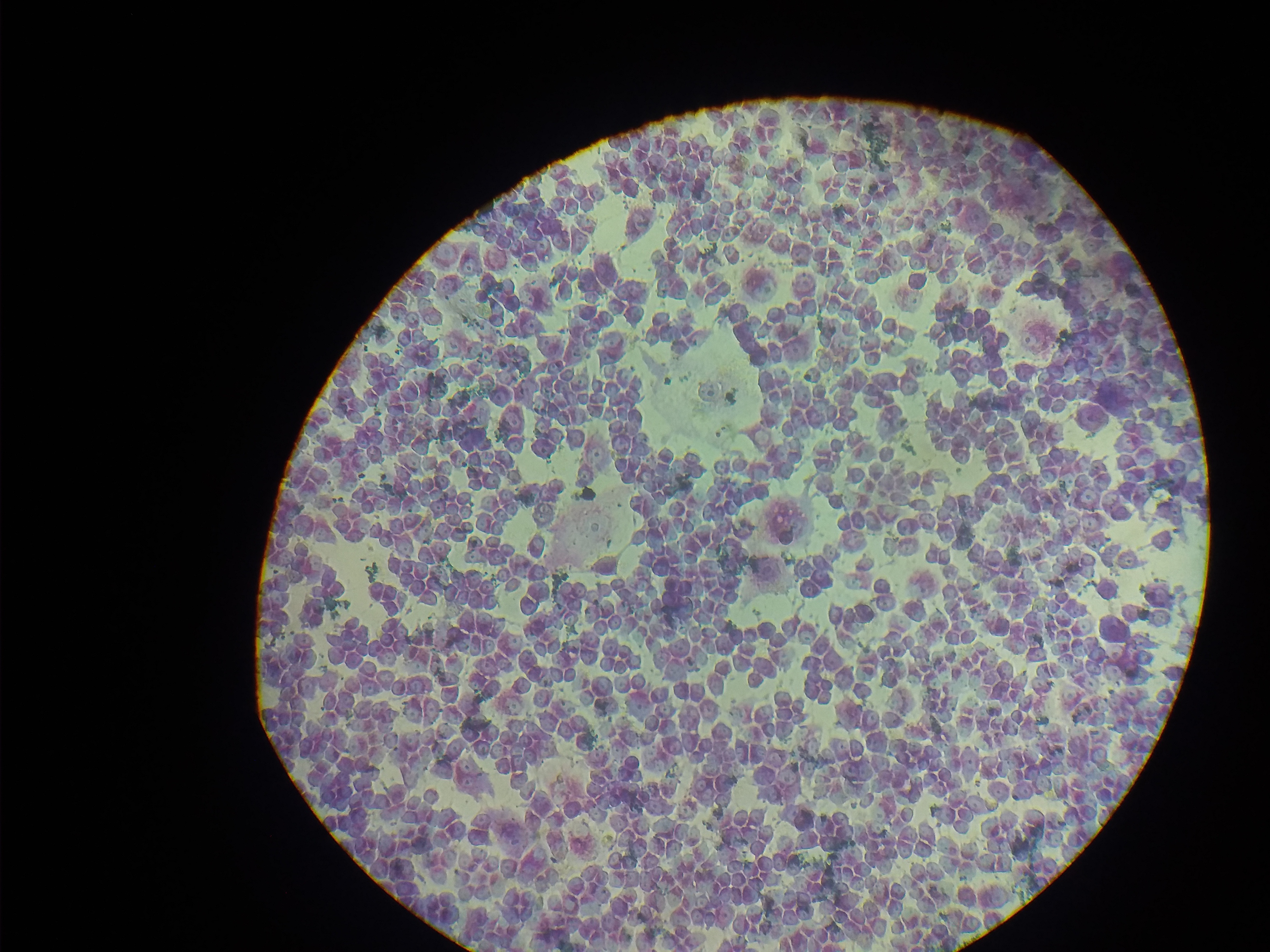

Supplement: Supplementary file 2 — Supplementary Information 2. [file 41598_2023_36721_MOESM2_ESM.zip › Raw data/Culture photos/20210609_180347.jpg]

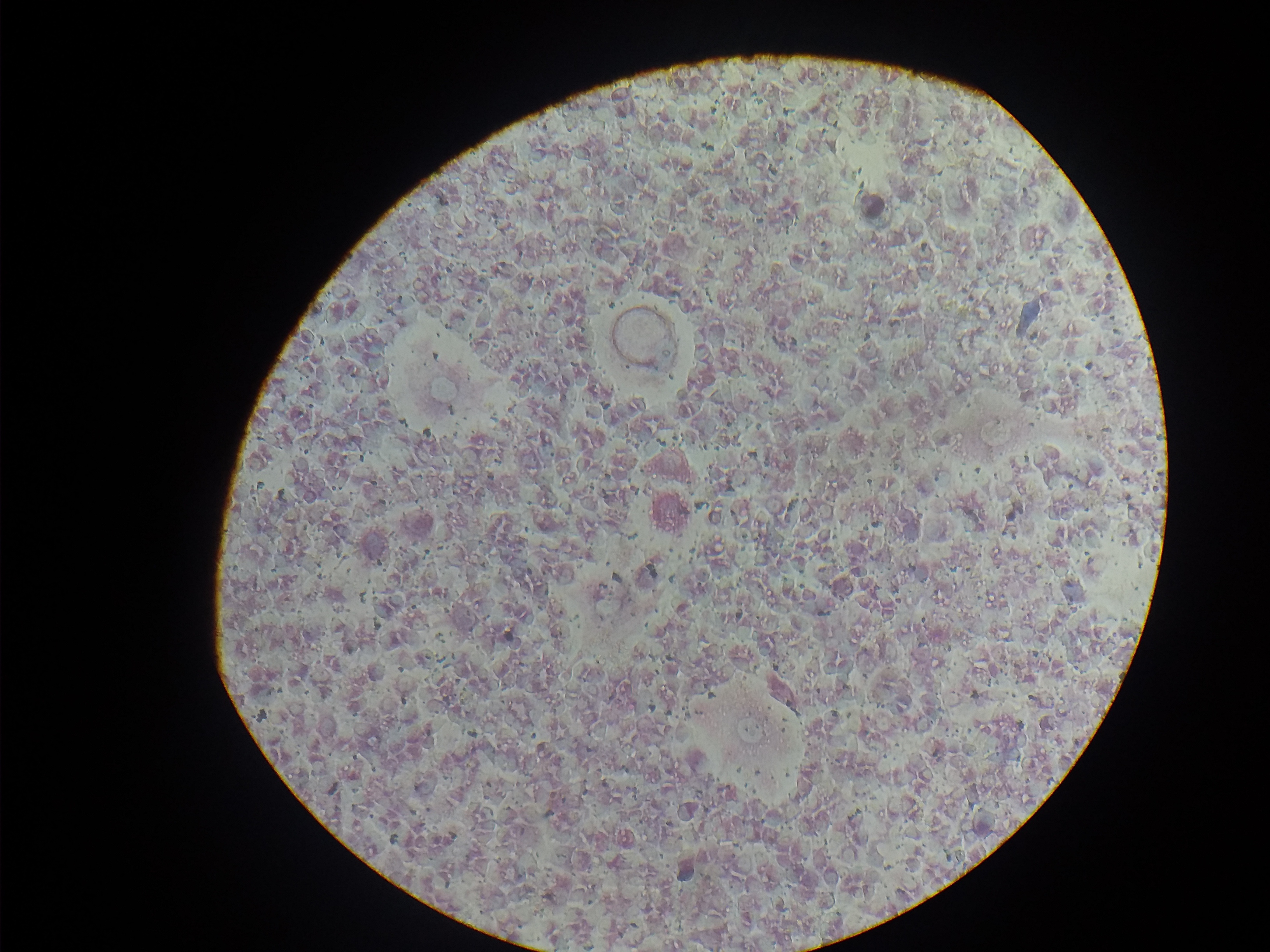

Supplement: Supplementary file 2 — Supplementary Information 2. [file 41598_2023_36721_MOESM2_ESM.zip › Raw data/Culture photos/20210609_180428.jpg]

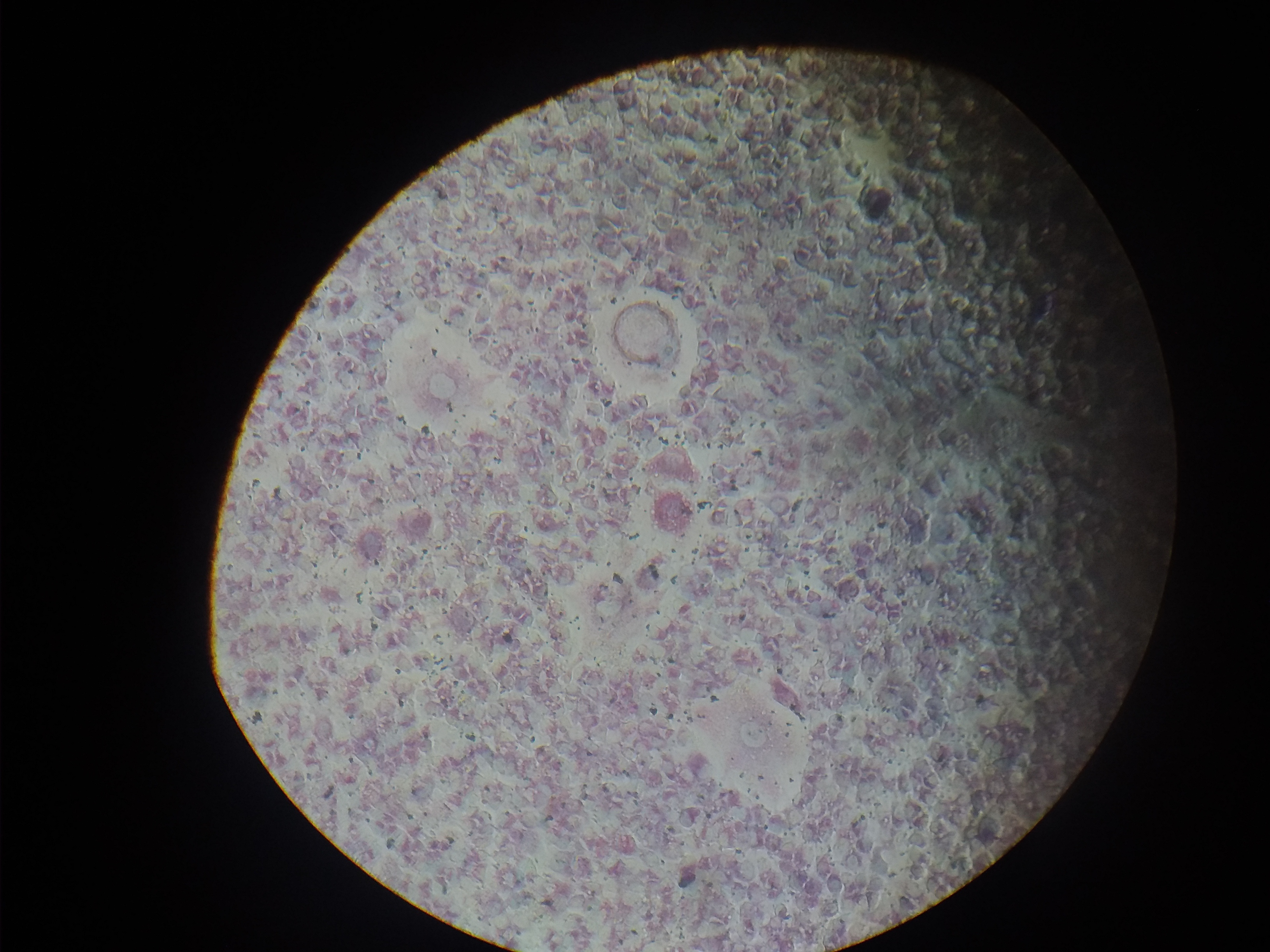

Supplement: Supplementary file 2 — Supplementary Information 2. [file 41598_2023_36721_MOESM2_ESM.zip › Raw data/Culture photos/20210609_180430.jpg]

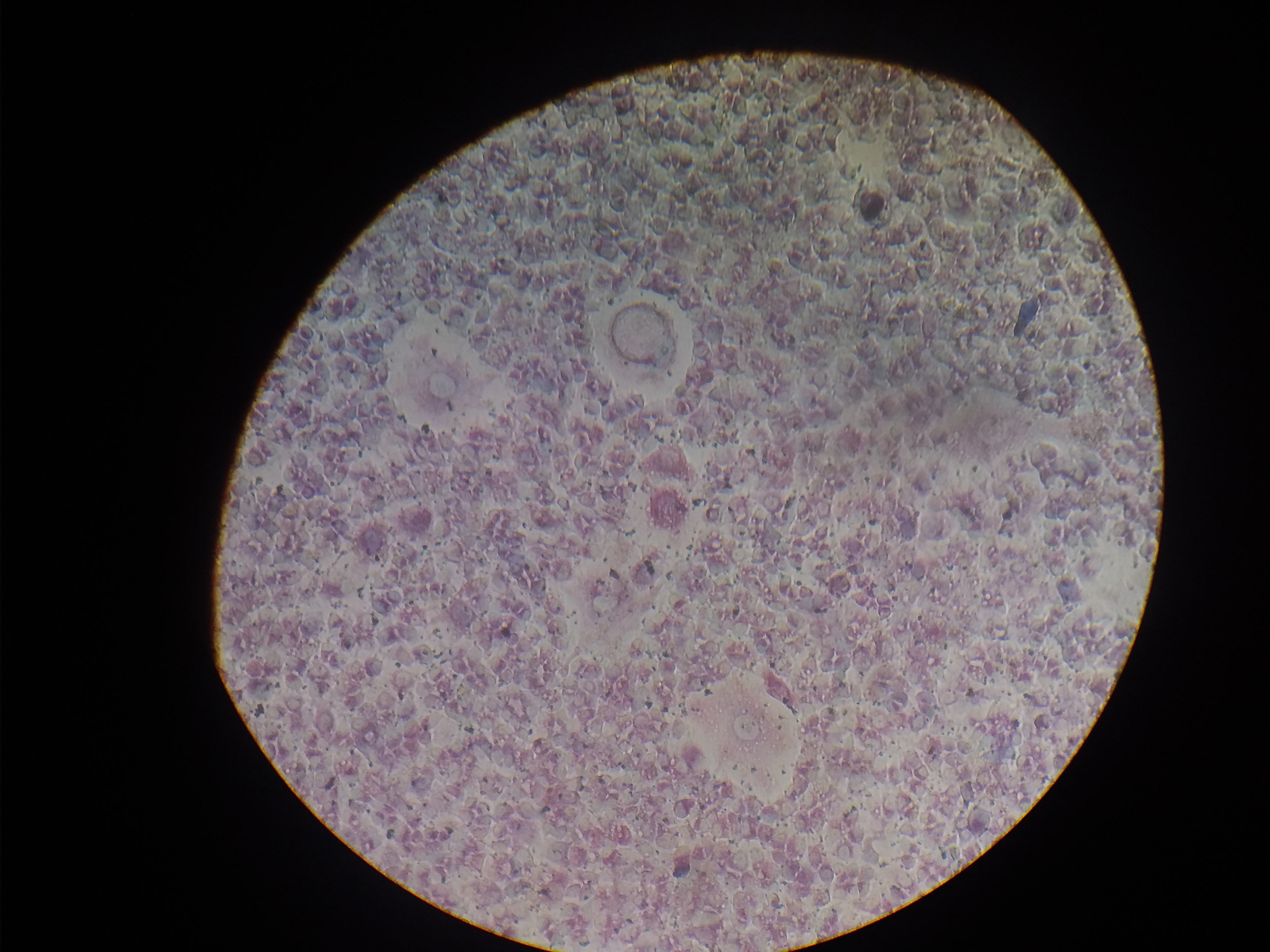

Supplement: Supplementary file 2 — Supplementary Information 2. [file 41598_2023_36721_MOESM2_ESM.zip › Raw data/Culture photos/20210609_180432.jpg]

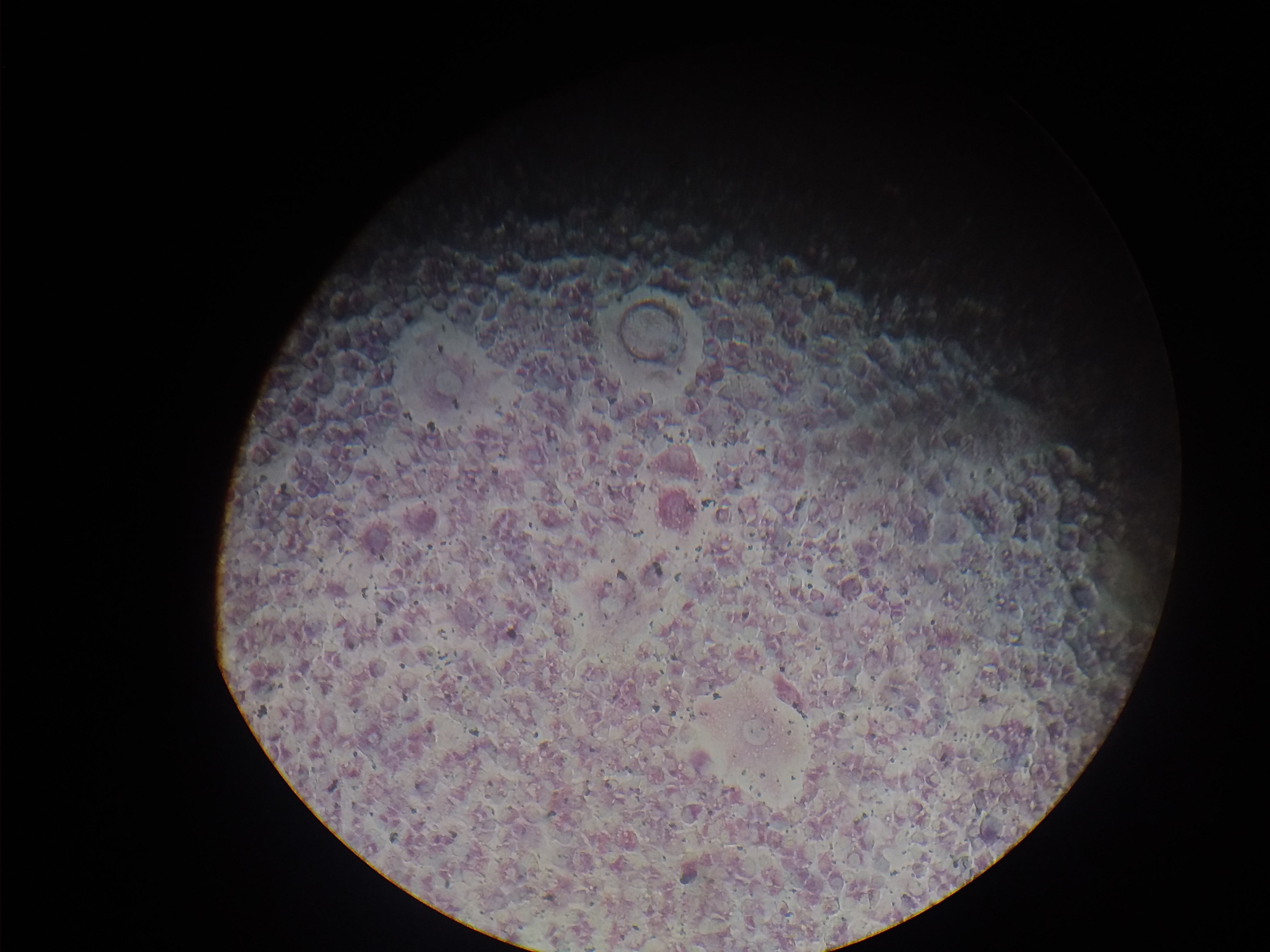

Supplement: Supplementary file 2 — Supplementary Information 2. [file 41598_2023_36721_MOESM2_ESM.zip › Raw data/Culture photos/20210609_180434.jpg]

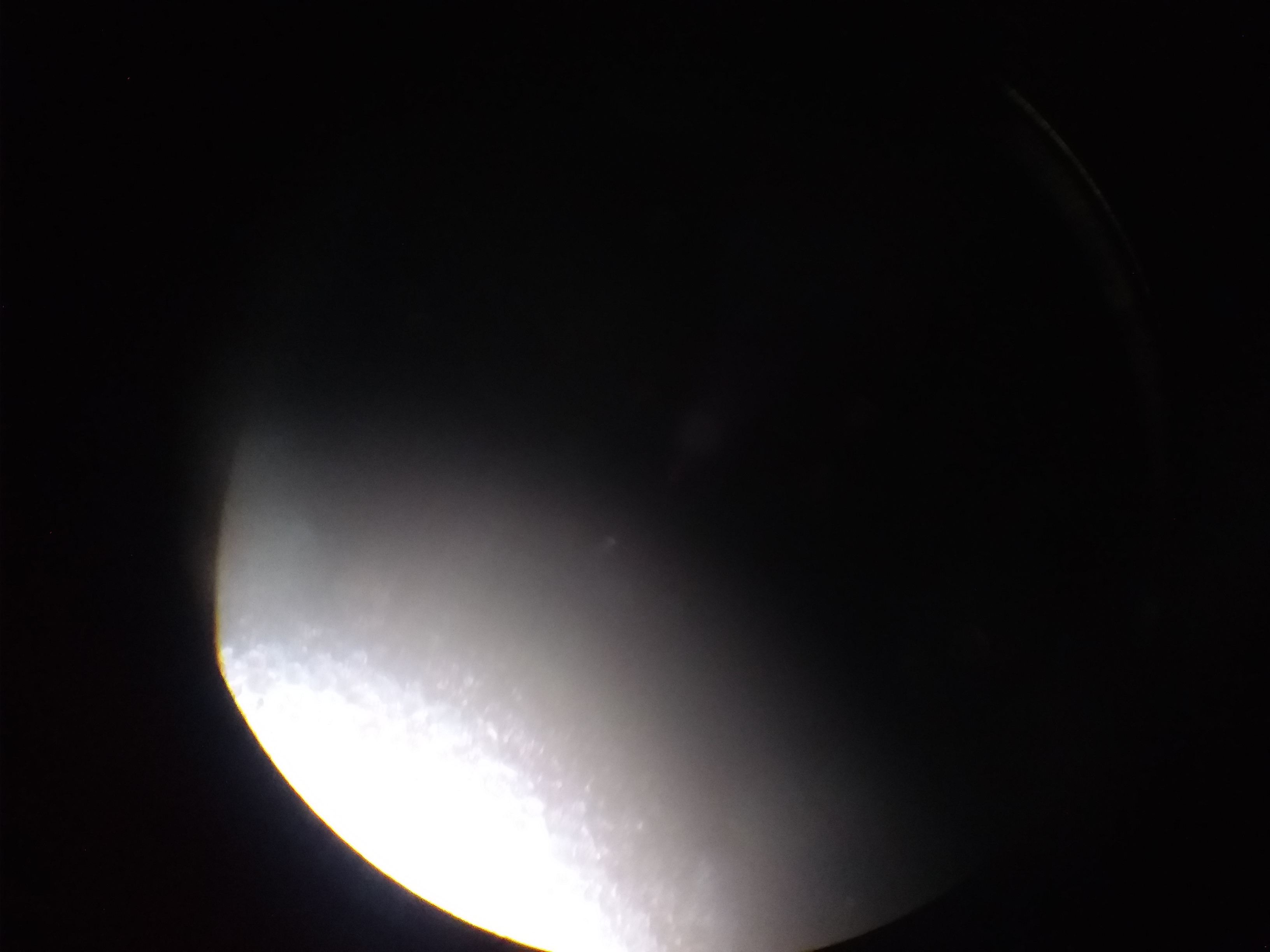

Supplement: Supplementary file 2 — Supplementary Information 2. [file 41598_2023_36721_MOESM2_ESM.zip › Raw data/Culture photos/20210609_180436.jpg]

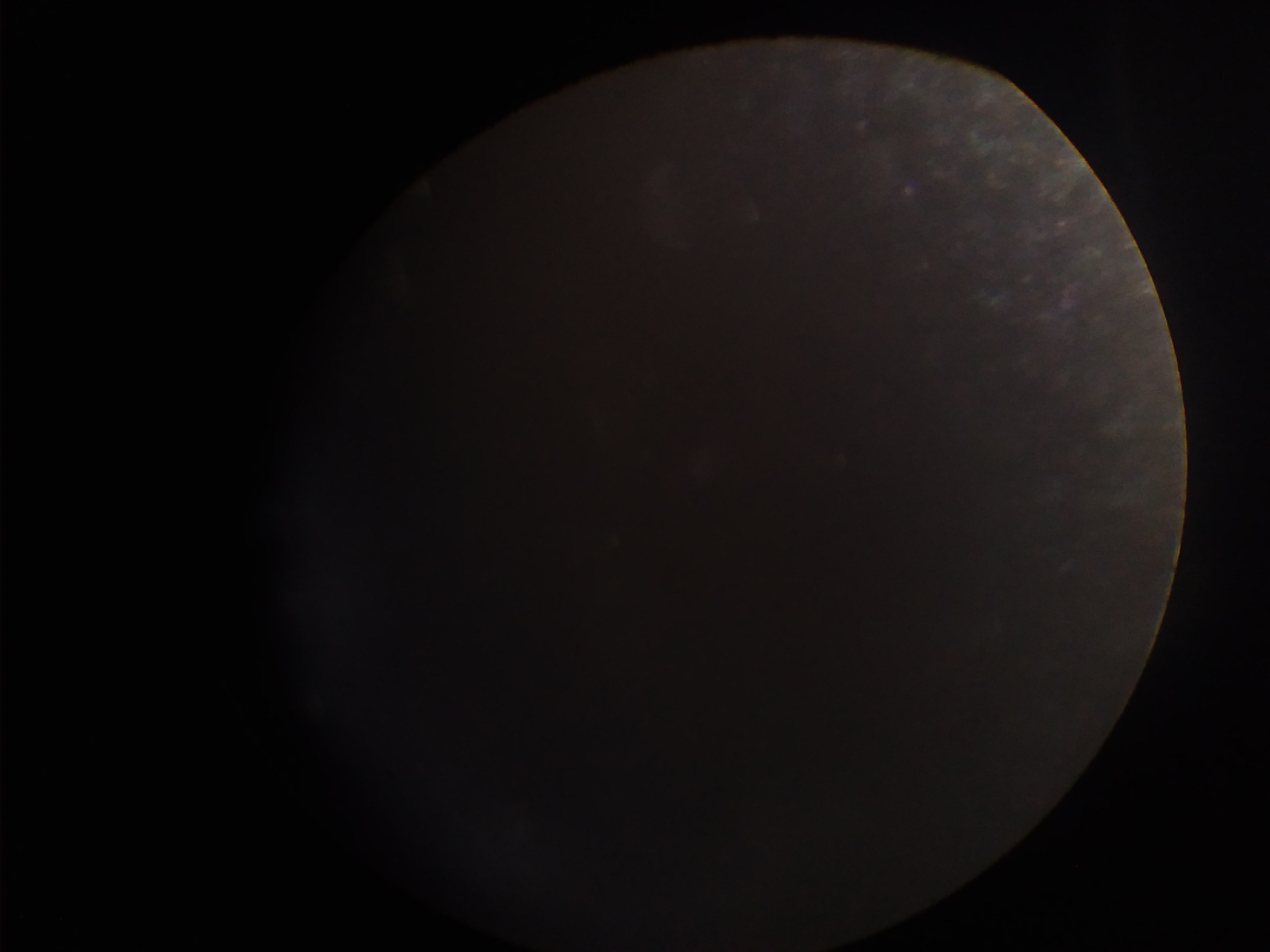

Supplement: Supplementary file 2 — Supplementary Information 2. [file 41598_2023_36721_MOESM2_ESM.zip › Raw data/Culture photos/20210609_180447.jpg]

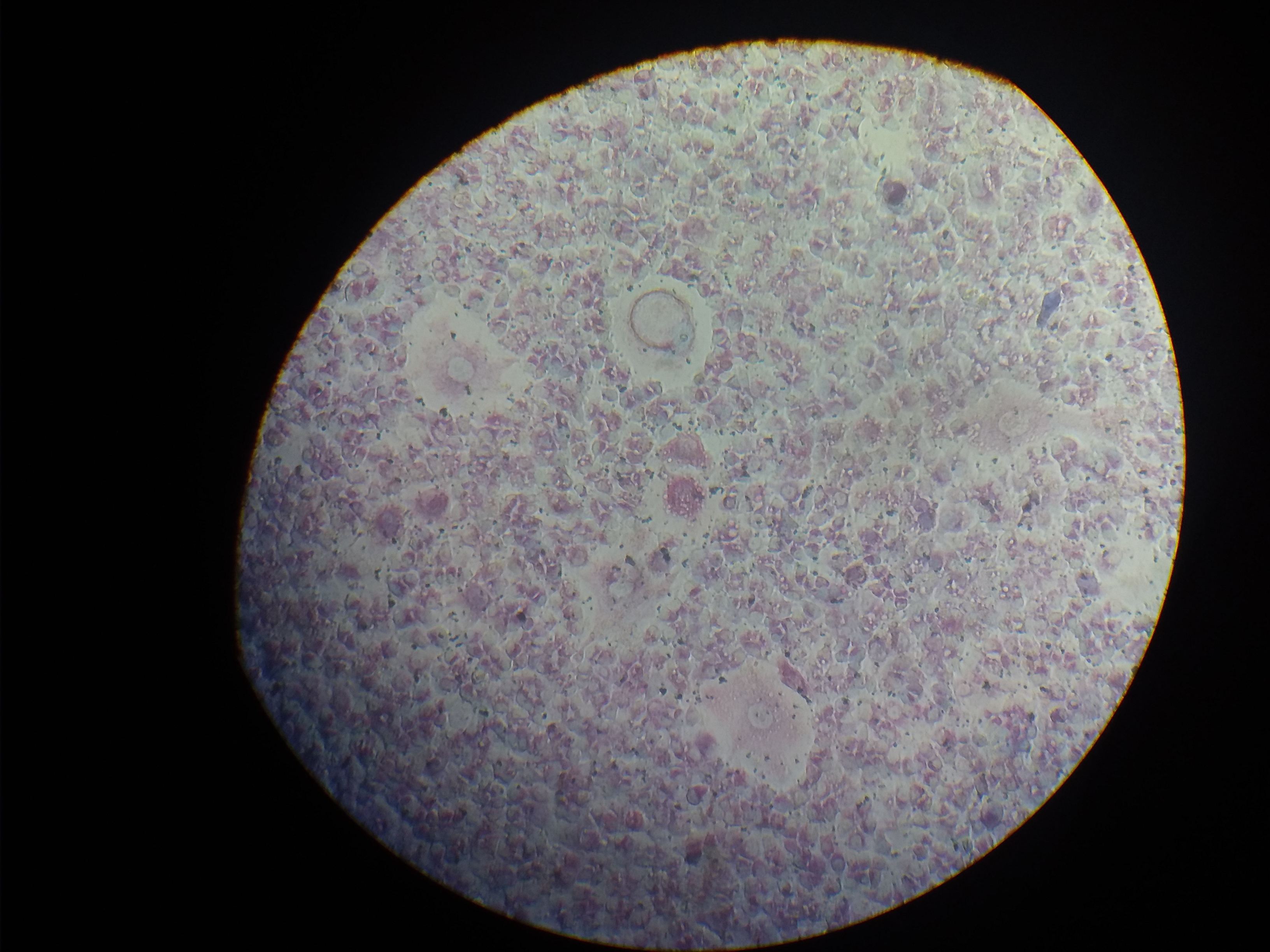

Supplement: Supplementary file 2 — Supplementary Information 2. [file 41598_2023_36721_MOESM2_ESM.zip › Raw data/Culture photos/20210609_180450.jpg]

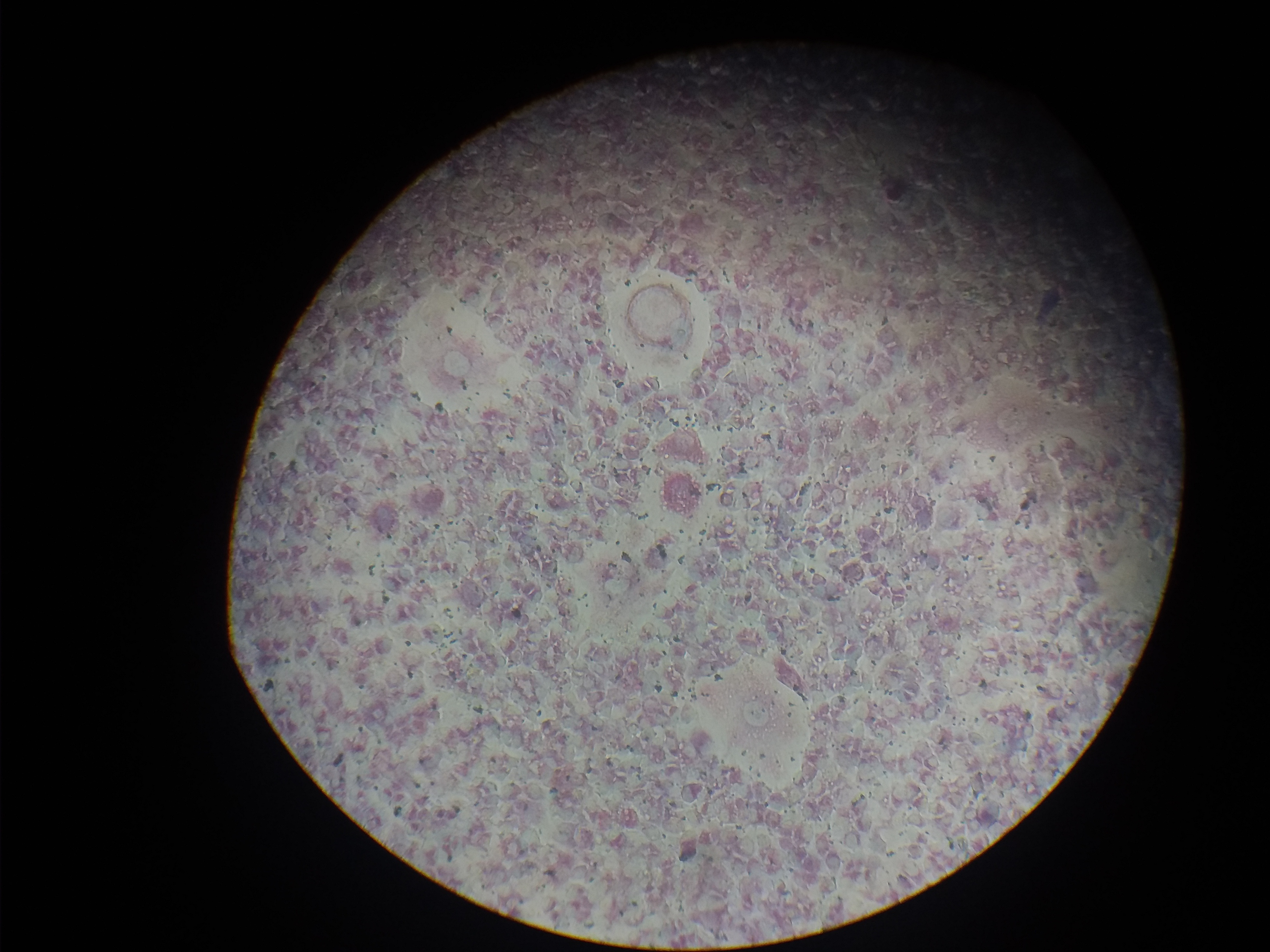

Supplement: Supplementary file 2 — Supplementary Information 2. [file 41598_2023_36721_MOESM2_ESM.zip › Raw data/Culture photos/20210609_180453.jpg]

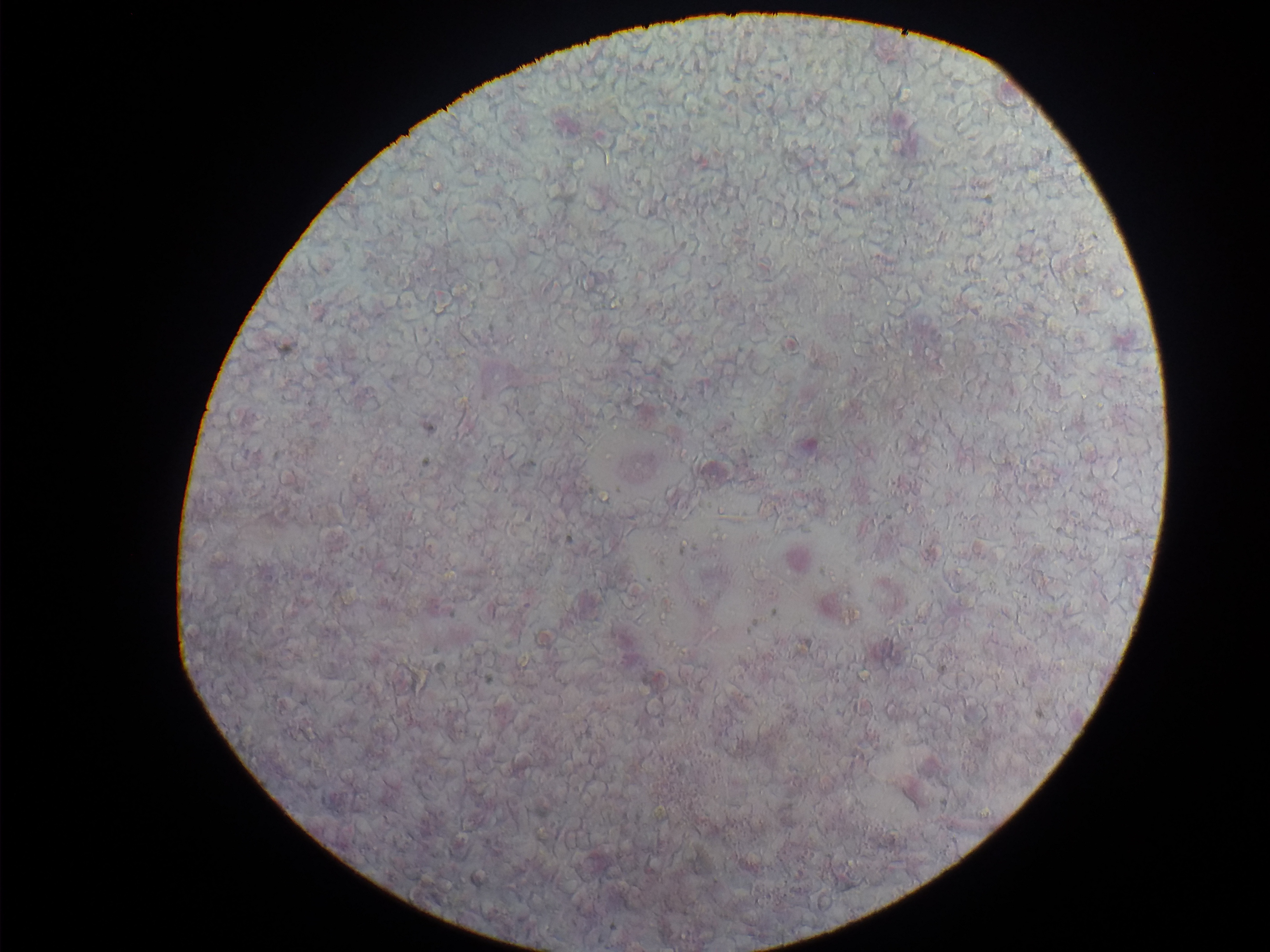

Supplement: Supplementary file 2 — Supplementary Information 2. [file 41598_2023_36721_MOESM2_ESM.zip › Raw data/Culture photos/20210609_180515.jpg]

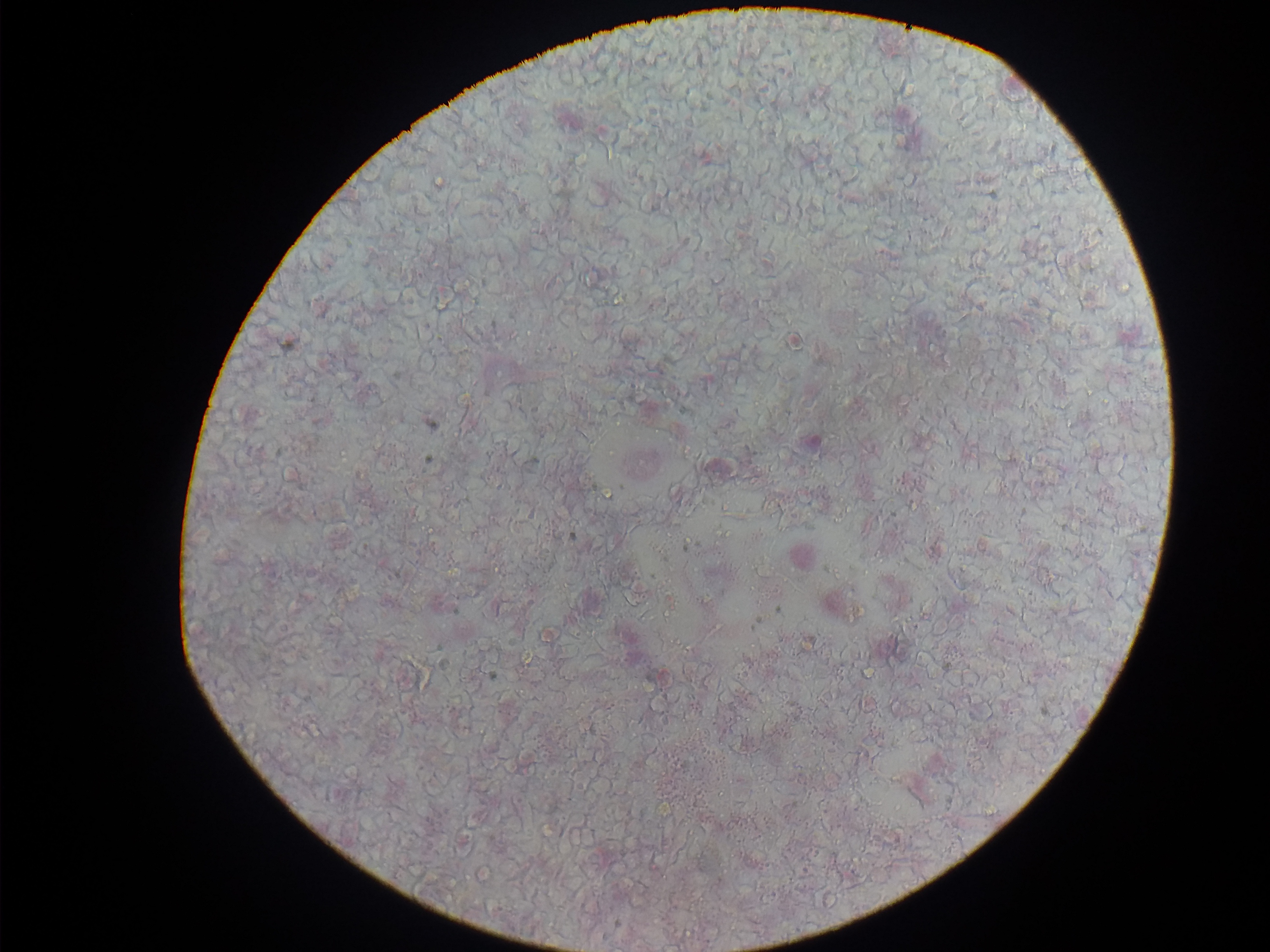

Supplement: Supplementary file 2 — Supplementary Information 2. [file 41598_2023_36721_MOESM2_ESM.zip › Raw data/Culture photos/20210609_180519.jpg]

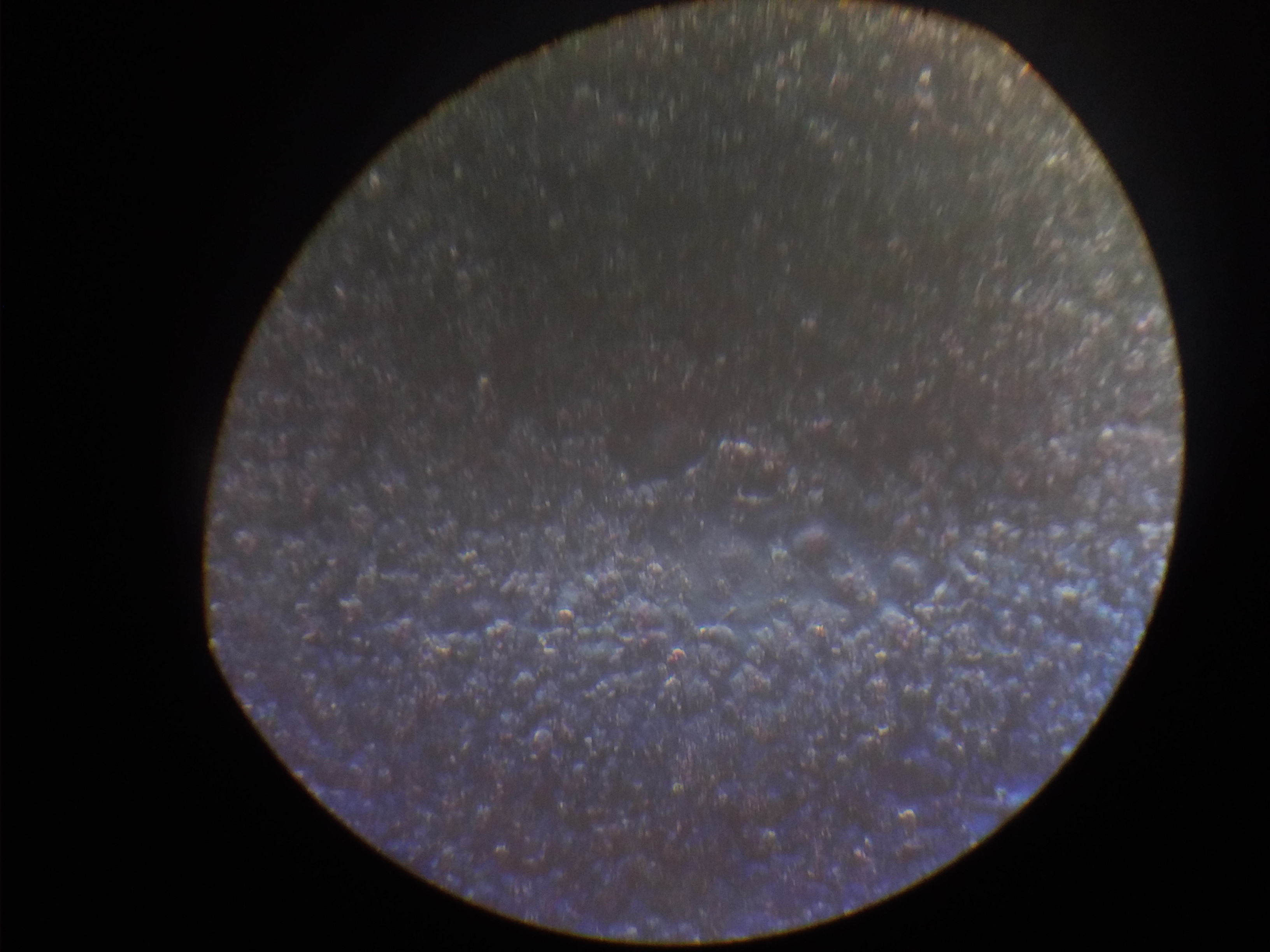

Supplement: Supplementary file 2 — Supplementary Information 2. [file 41598_2023_36721_MOESM2_ESM.zip › Raw data/Culture photos/20210609_180527.jpg]

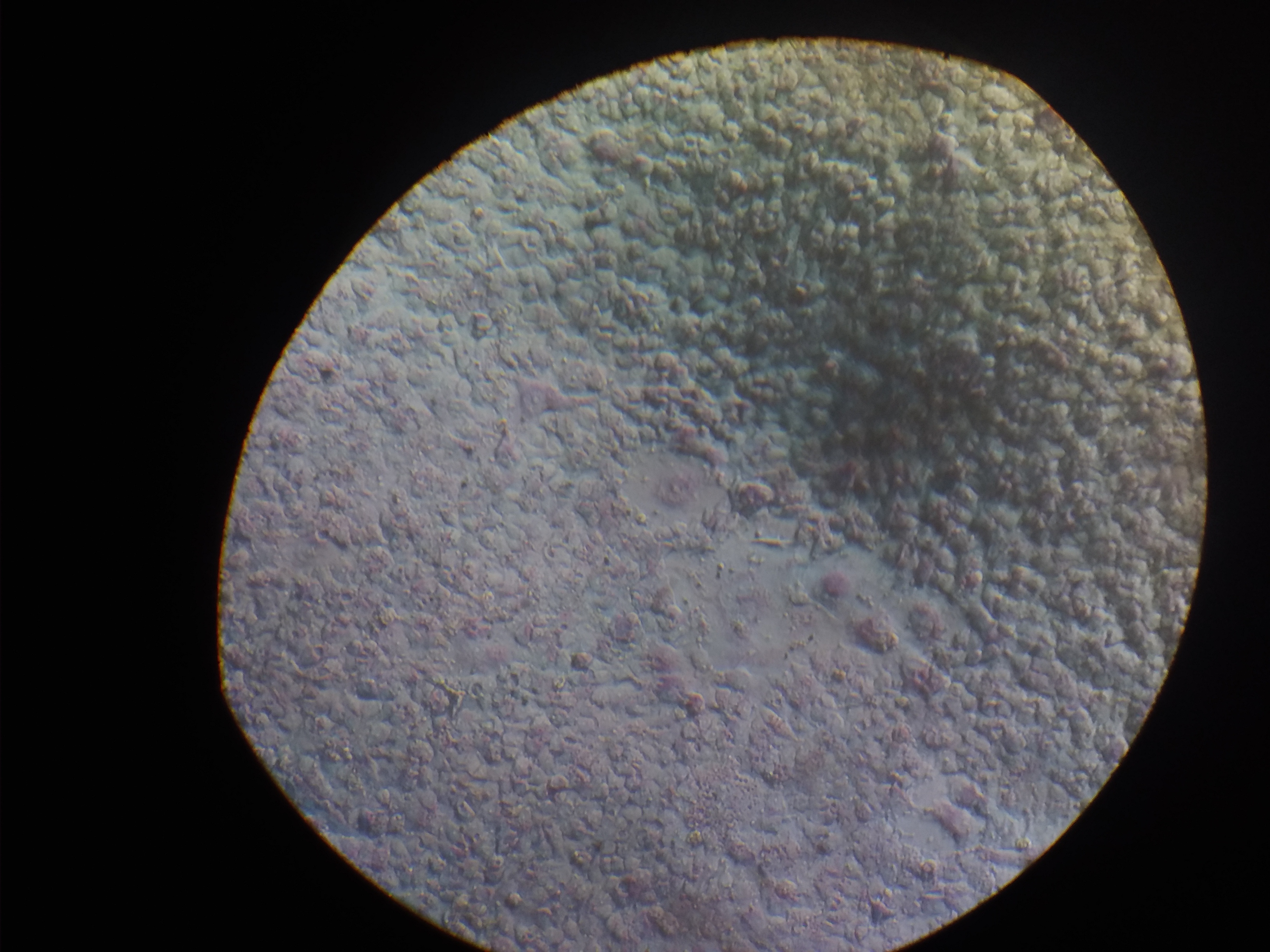

Supplement: Supplementary file 2 — Supplementary Information 2. [file 41598_2023_36721_MOESM2_ESM.zip › Raw data/Culture photos/20210609_180531.jpg]

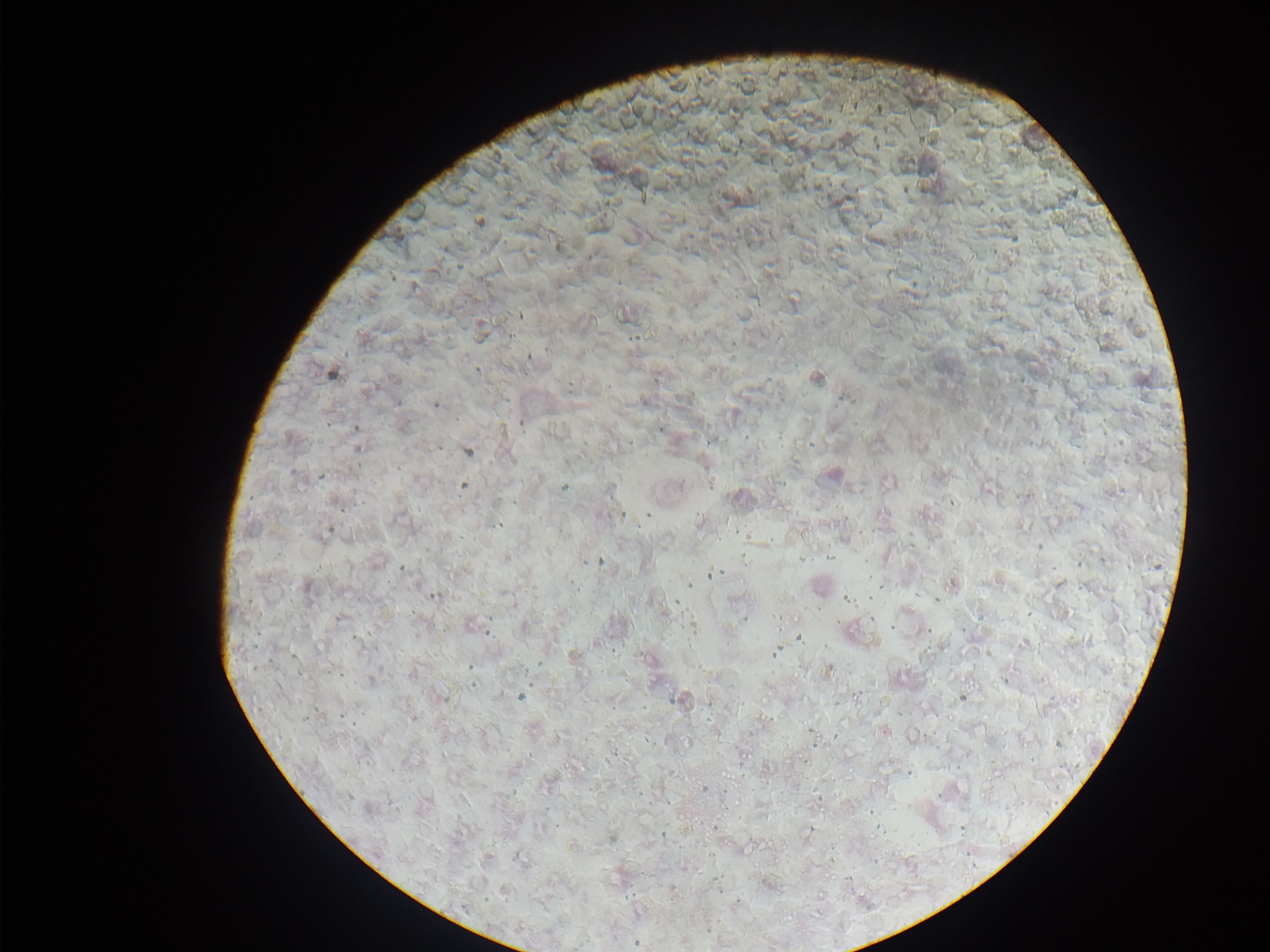

Supplement: Supplementary file 2 — Supplementary Information 2. [file 41598_2023_36721_MOESM2_ESM.zip › Raw data/Culture photos/20210609_180542.jpg]

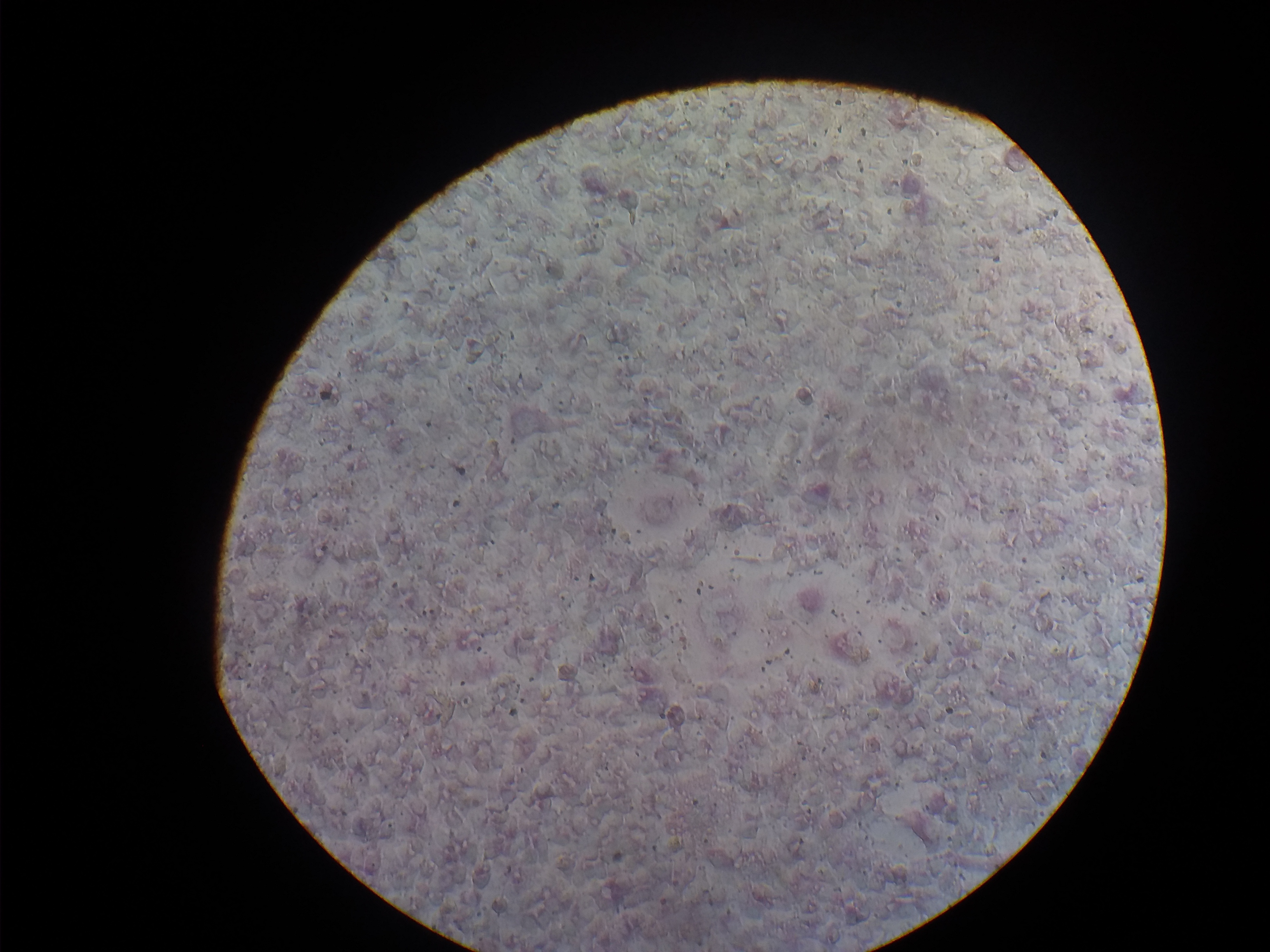

Supplement: Supplementary file 2 — Supplementary Information 2. [file 41598_2023_36721_MOESM2_ESM.zip › Raw data/Culture photos/20210609_180556.jpg]

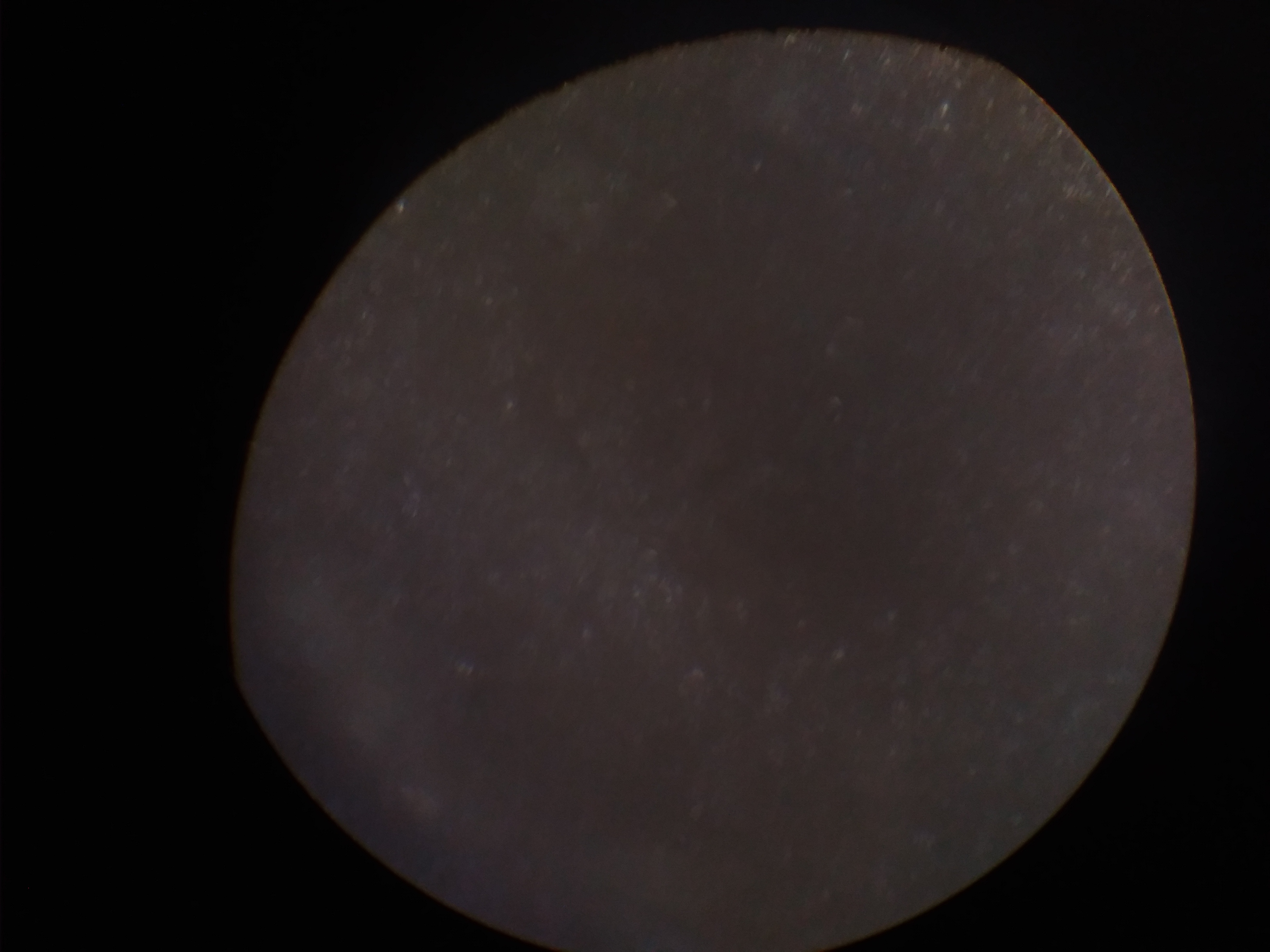

Supplement: Supplementary file 2 — Supplementary Information 2. [file 41598_2023_36721_MOESM2_ESM.zip › Raw data/Culture photos/20210609_180615.jpg]

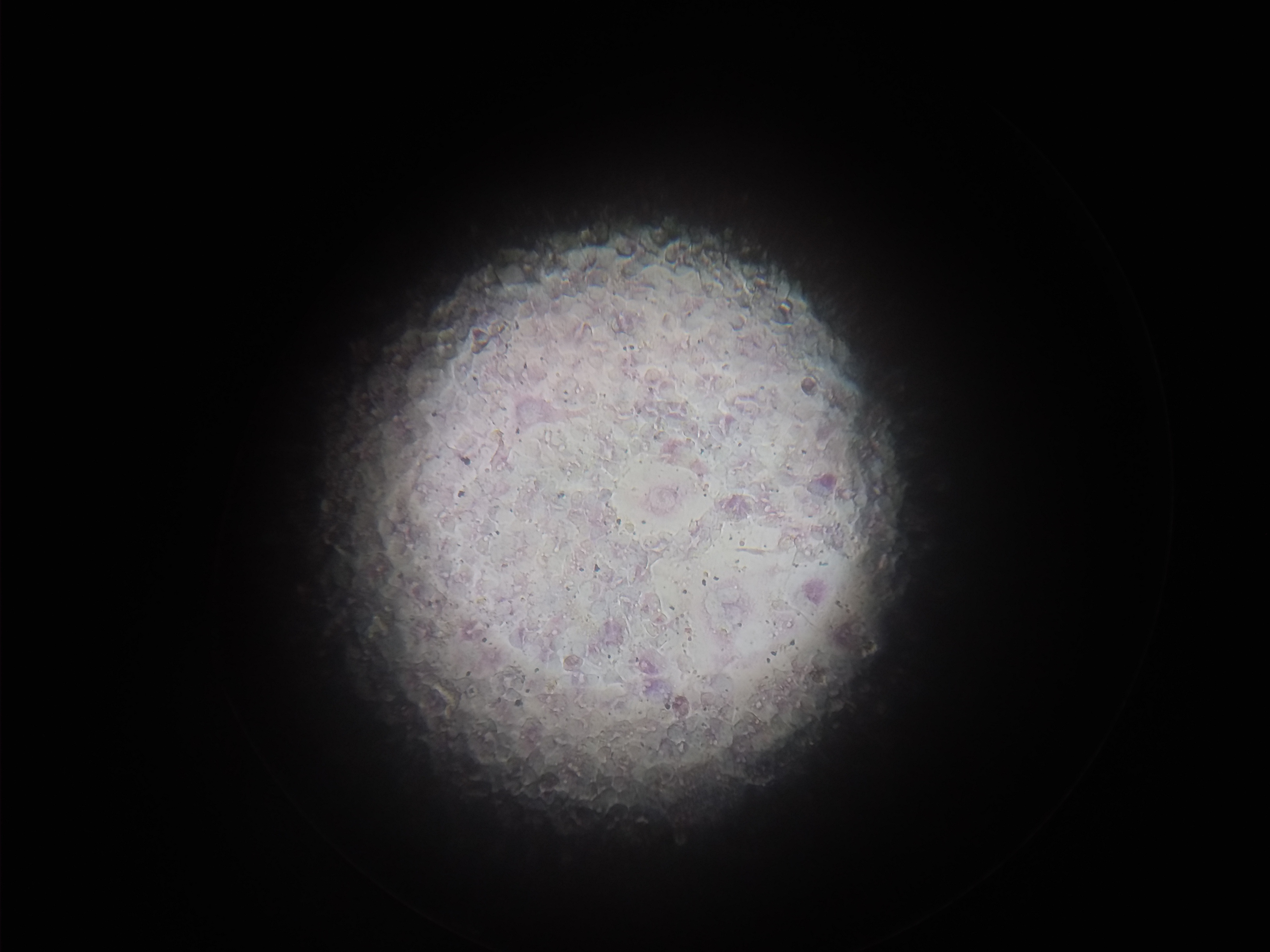

Supplement: Supplementary file 2 — Supplementary Information 2. [file 41598_2023_36721_MOESM2_ESM.zip › Raw data/Culture photos/20210609_180626.jpg]

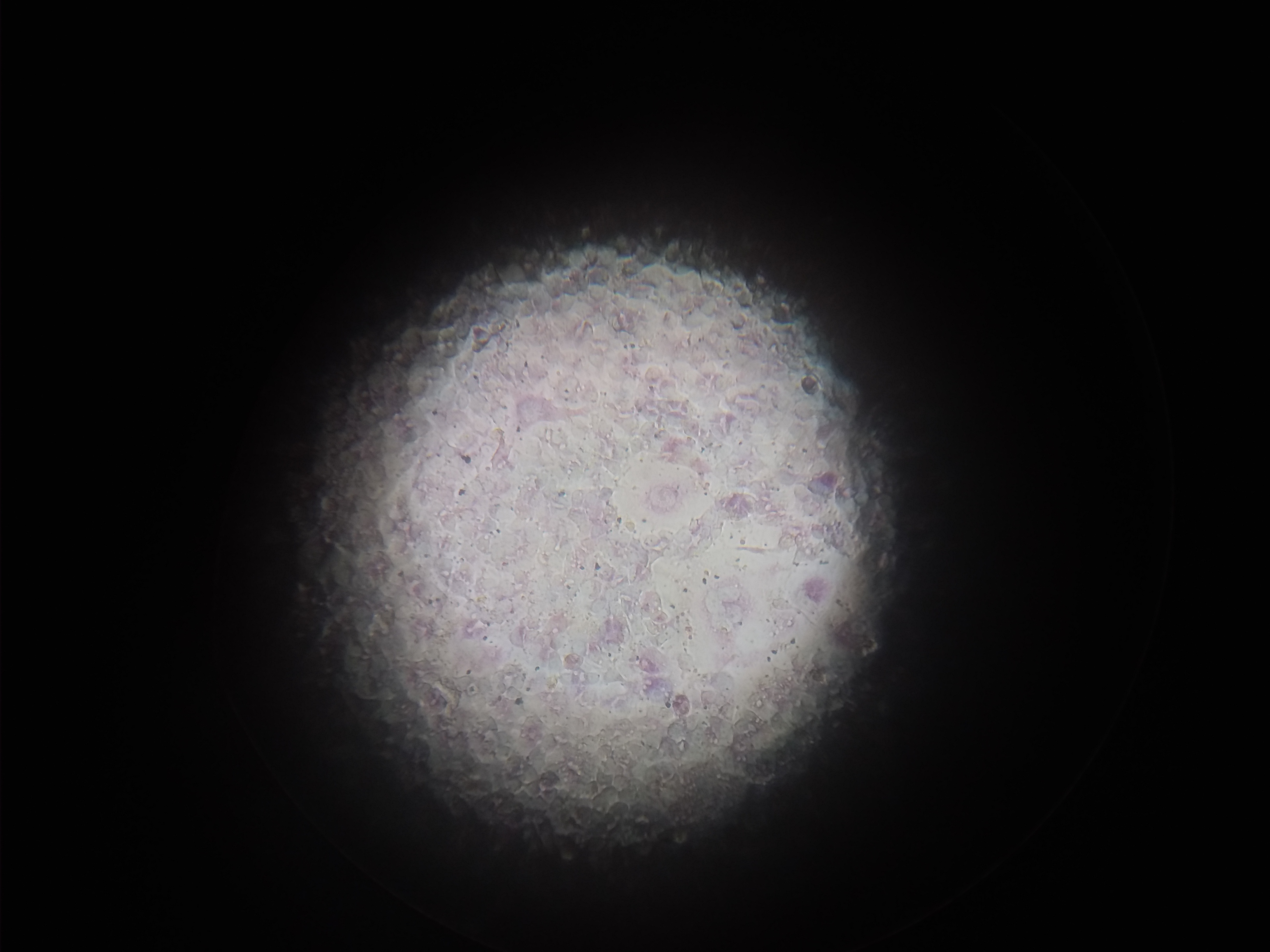

Supplement: Supplementary file 2 — Supplementary Information 2. [file 41598_2023_36721_MOESM2_ESM.zip › Raw data/Culture photos/20210609_180628.jpg]

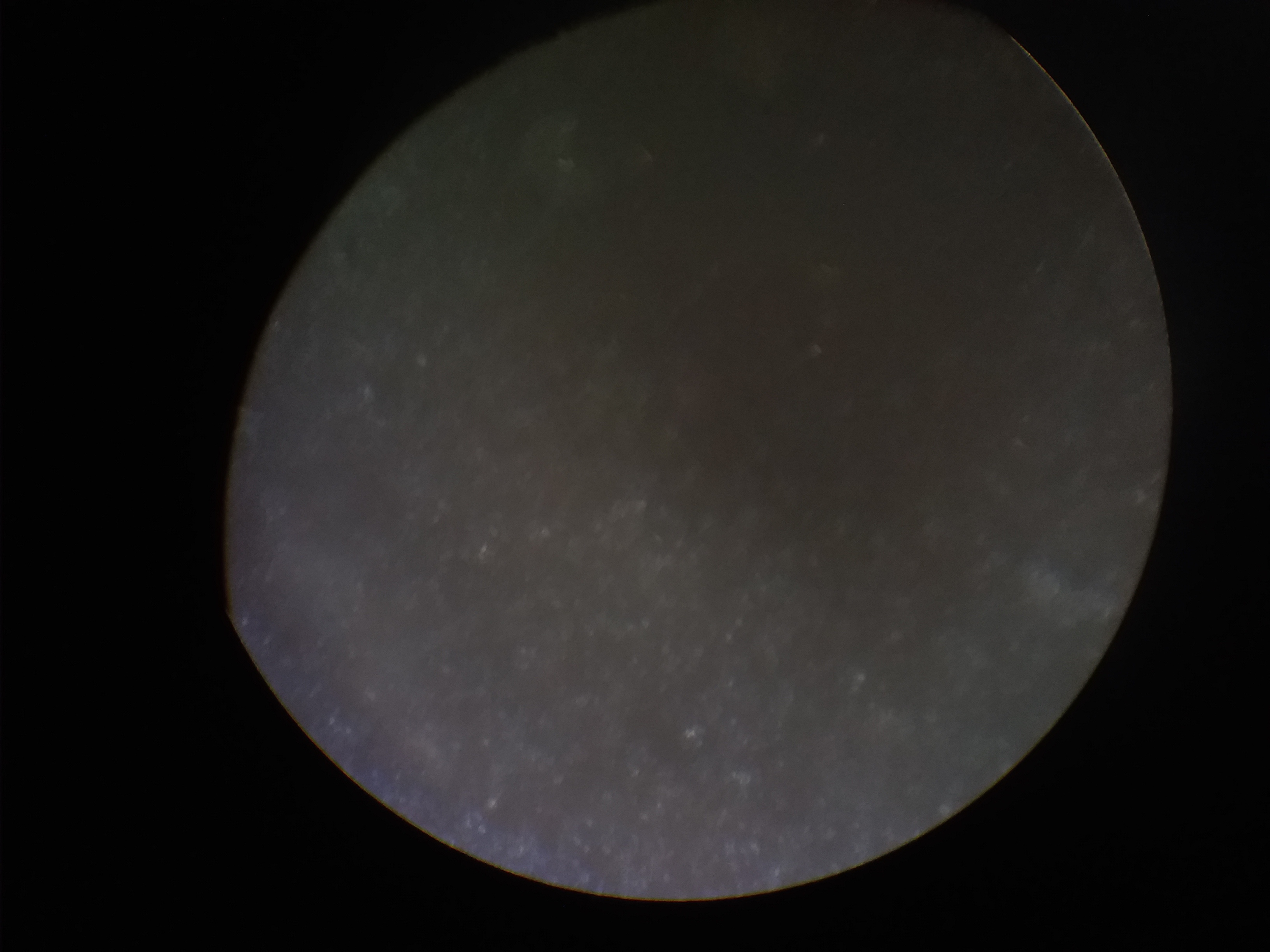

Supplement: Supplementary file 2 — Supplementary Information 2. [file 41598_2023_36721_MOESM2_ESM.zip › Raw data/Culture photos/20210609_180652.jpg]

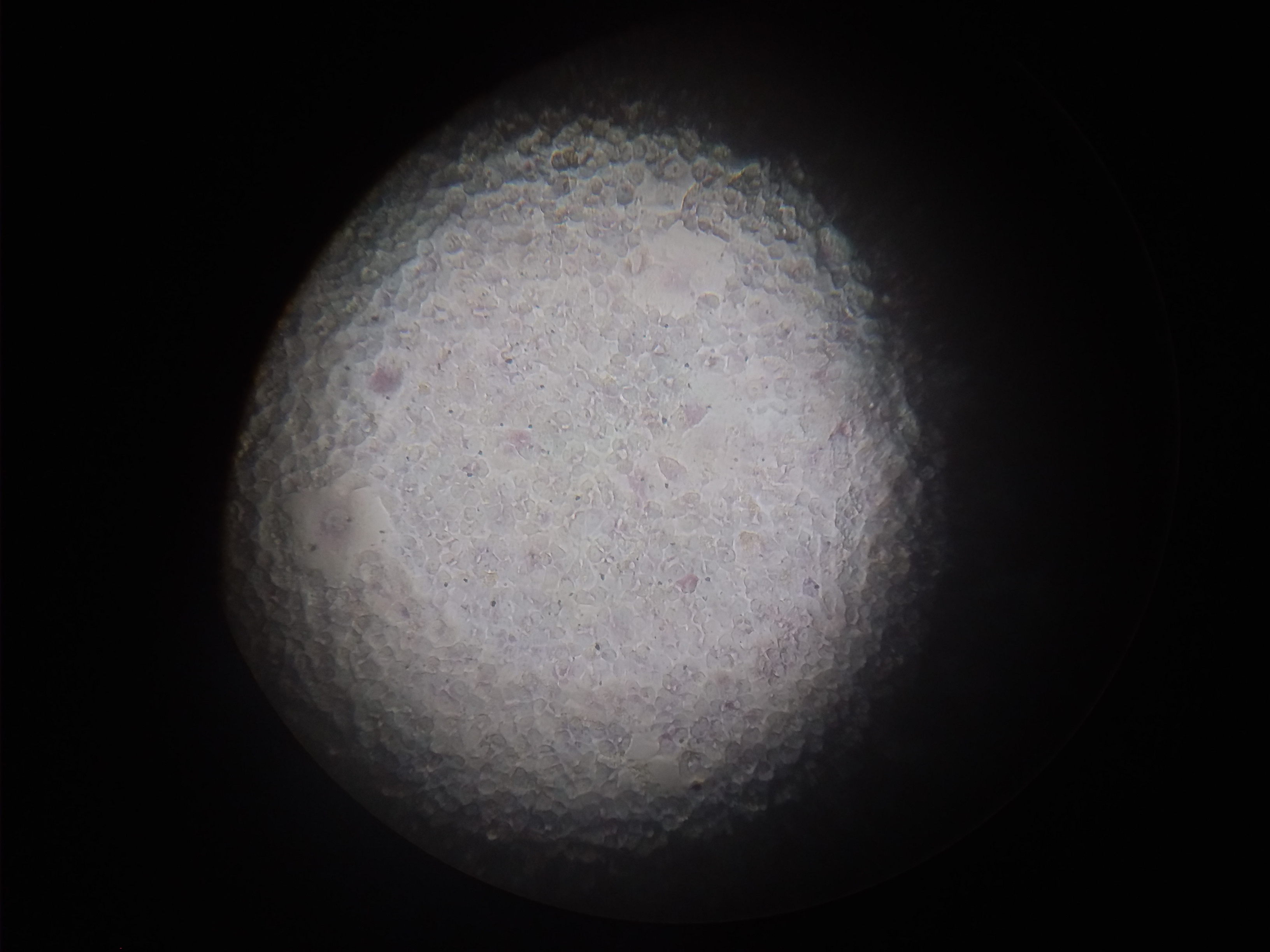

Supplement: Supplementary file 2 — Supplementary Information 2. [file 41598_2023_36721_MOESM2_ESM.zip › Raw data/Culture photos/20210609_180657.jpg]

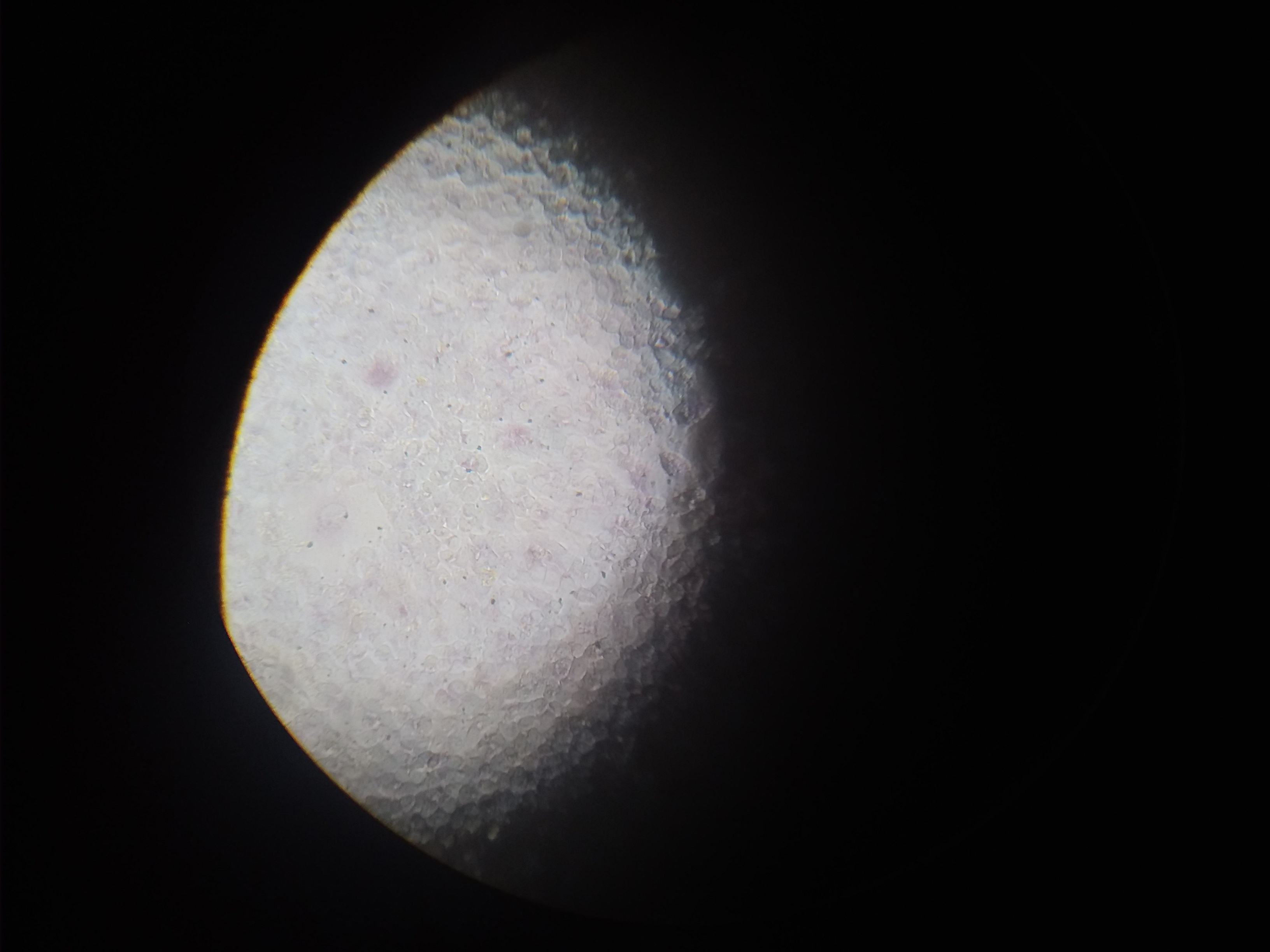

Supplement: Supplementary file 2 — Supplementary Information 2. [file 41598_2023_36721_MOESM2_ESM.zip › Raw data/Culture photos/20210609_180659.jpg]

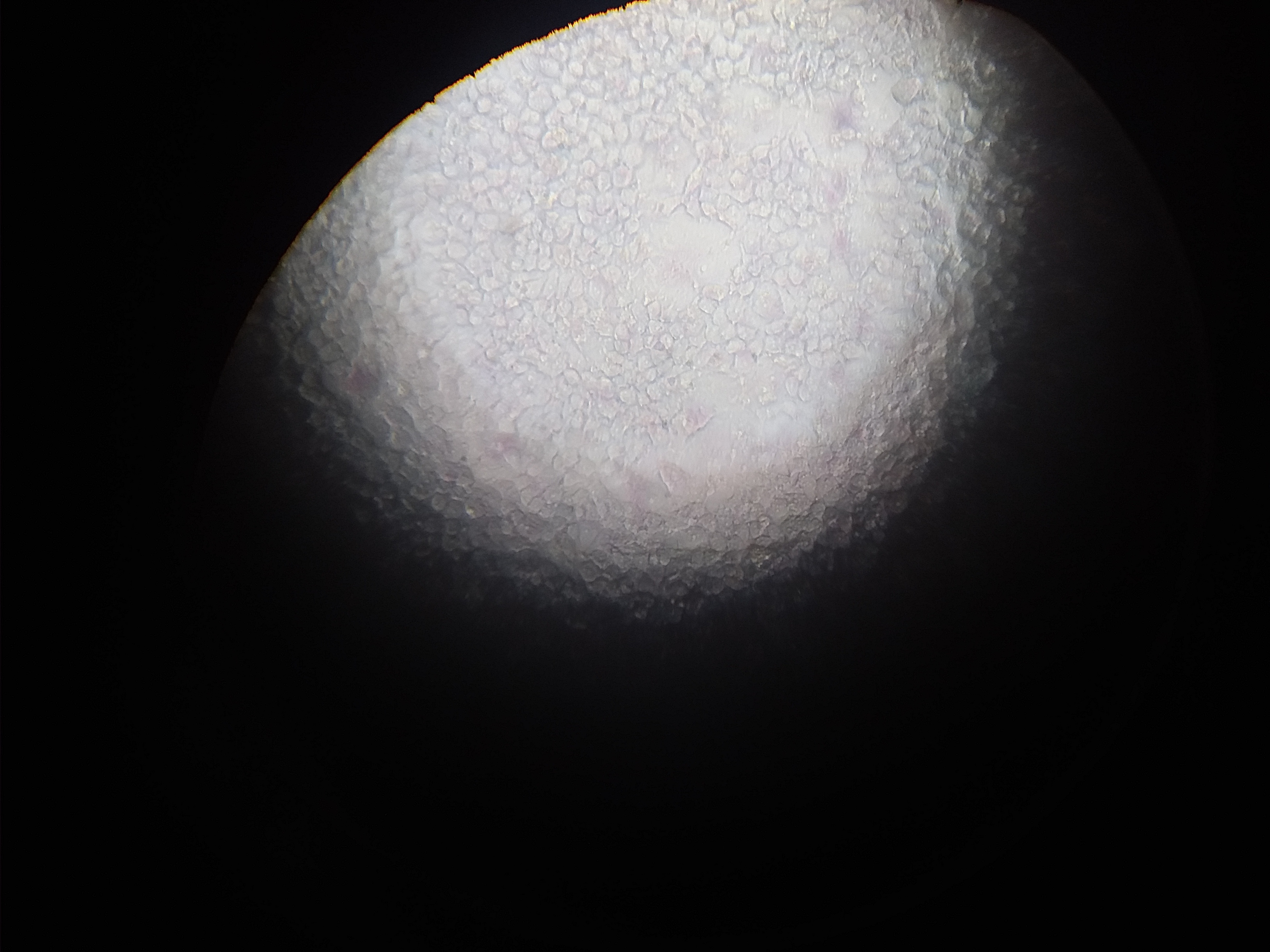

Supplement: Supplementary file 2 — Supplementary Information 2. [file 41598_2023_36721_MOESM2_ESM.zip › Raw data/Culture photos/20210609_180702.jpg]

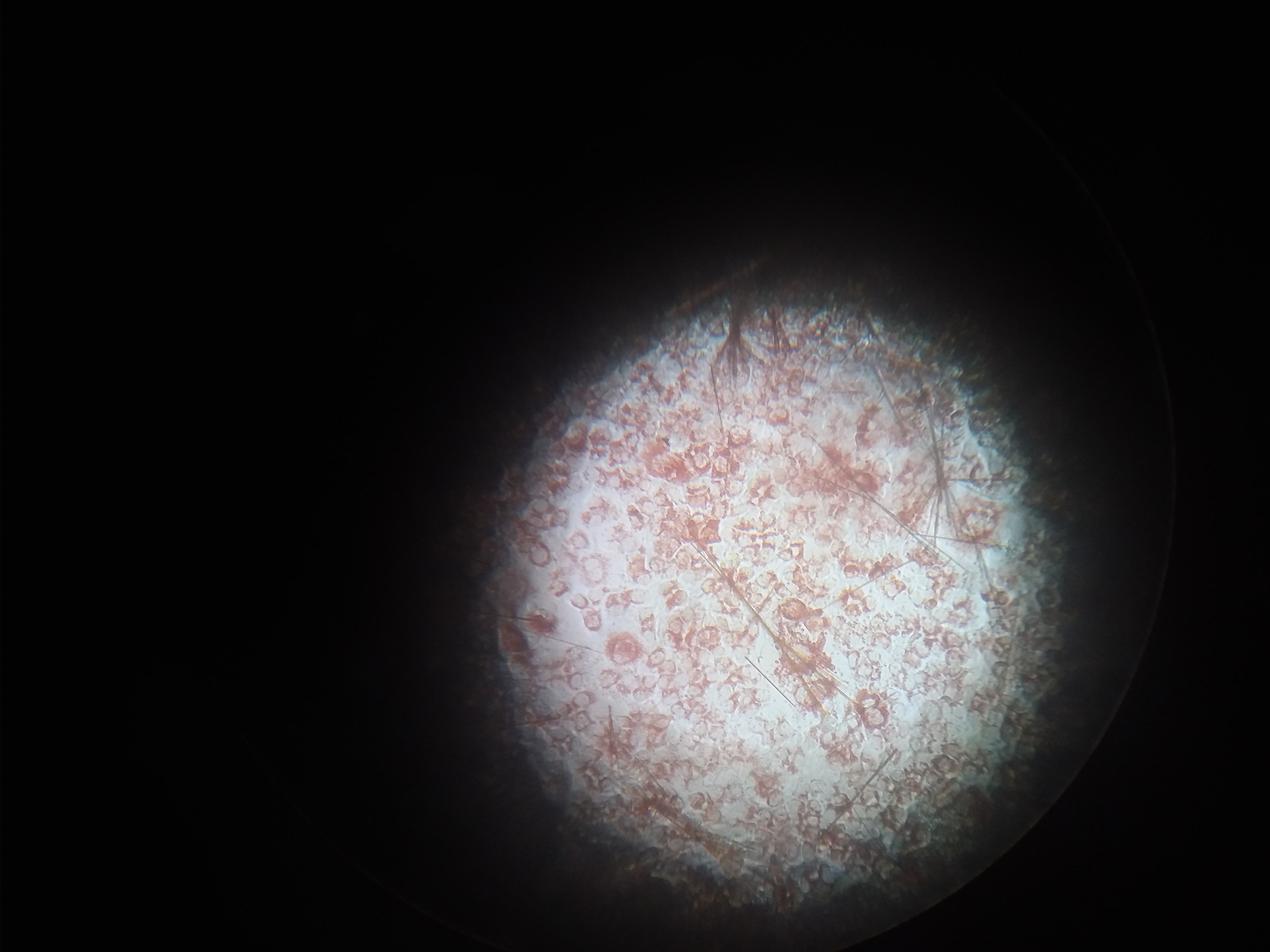

Supplement: Supplementary file 2 — Supplementary Information 2. [file 41598_2023_36721_MOESM2_ESM.zip › Raw data/Culture photos/20210609_180721.jpg]

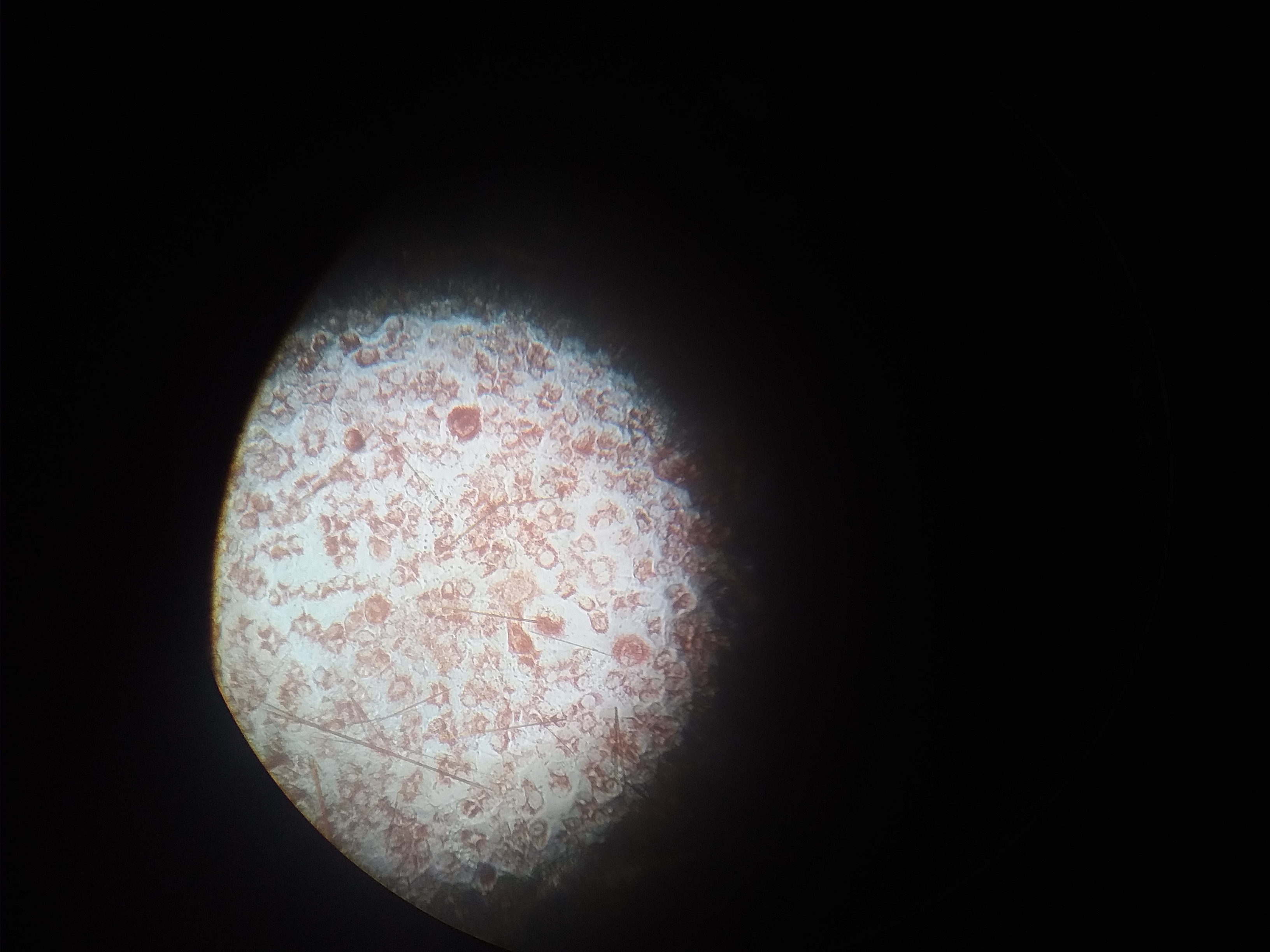

Supplement: Supplementary file 2 — Supplementary Information 2. [file 41598_2023_36721_MOESM2_ESM.zip › Raw data/Culture photos/20210609_180723.jpg]

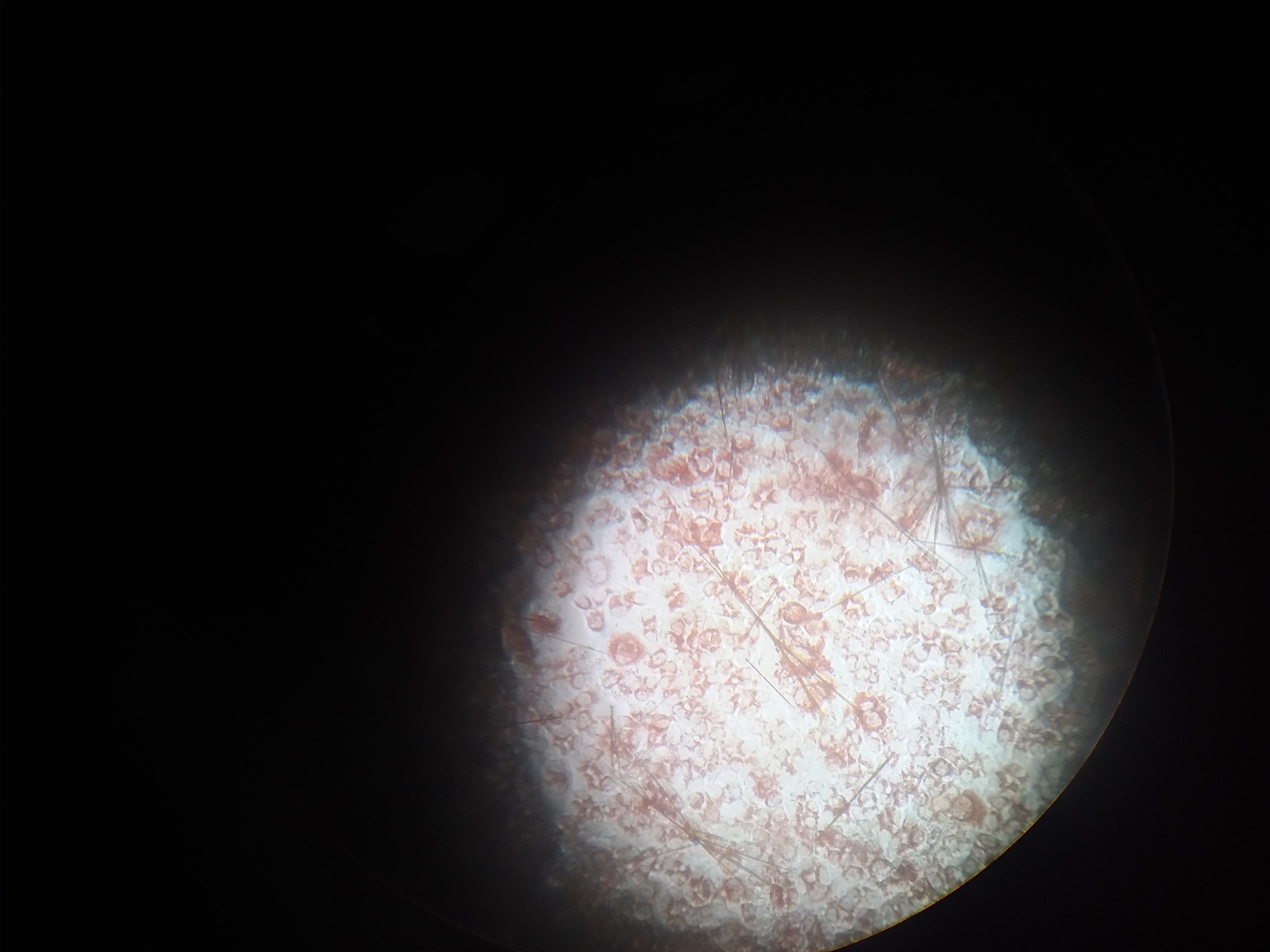

Supplement: Supplementary file 2 — Supplementary Information 2. [file 41598_2023_36721_MOESM2_ESM.zip › Raw data/Culture photos/20210609_180729.jpg]

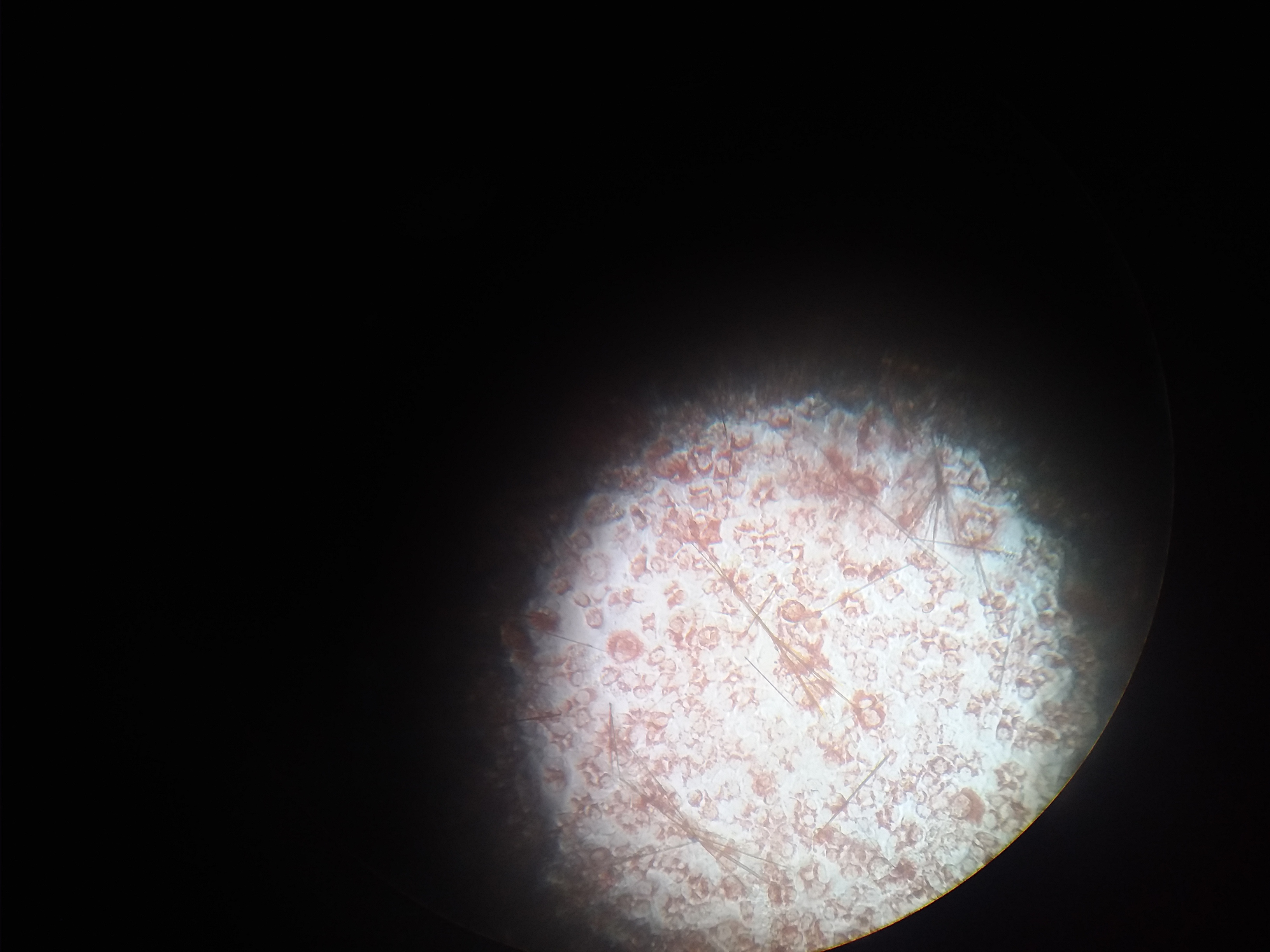

Supplement: Supplementary file 2 — Supplementary Information 2. [file 41598_2023_36721_MOESM2_ESM.zip › Raw data/Culture photos/20210609_180730.jpg]

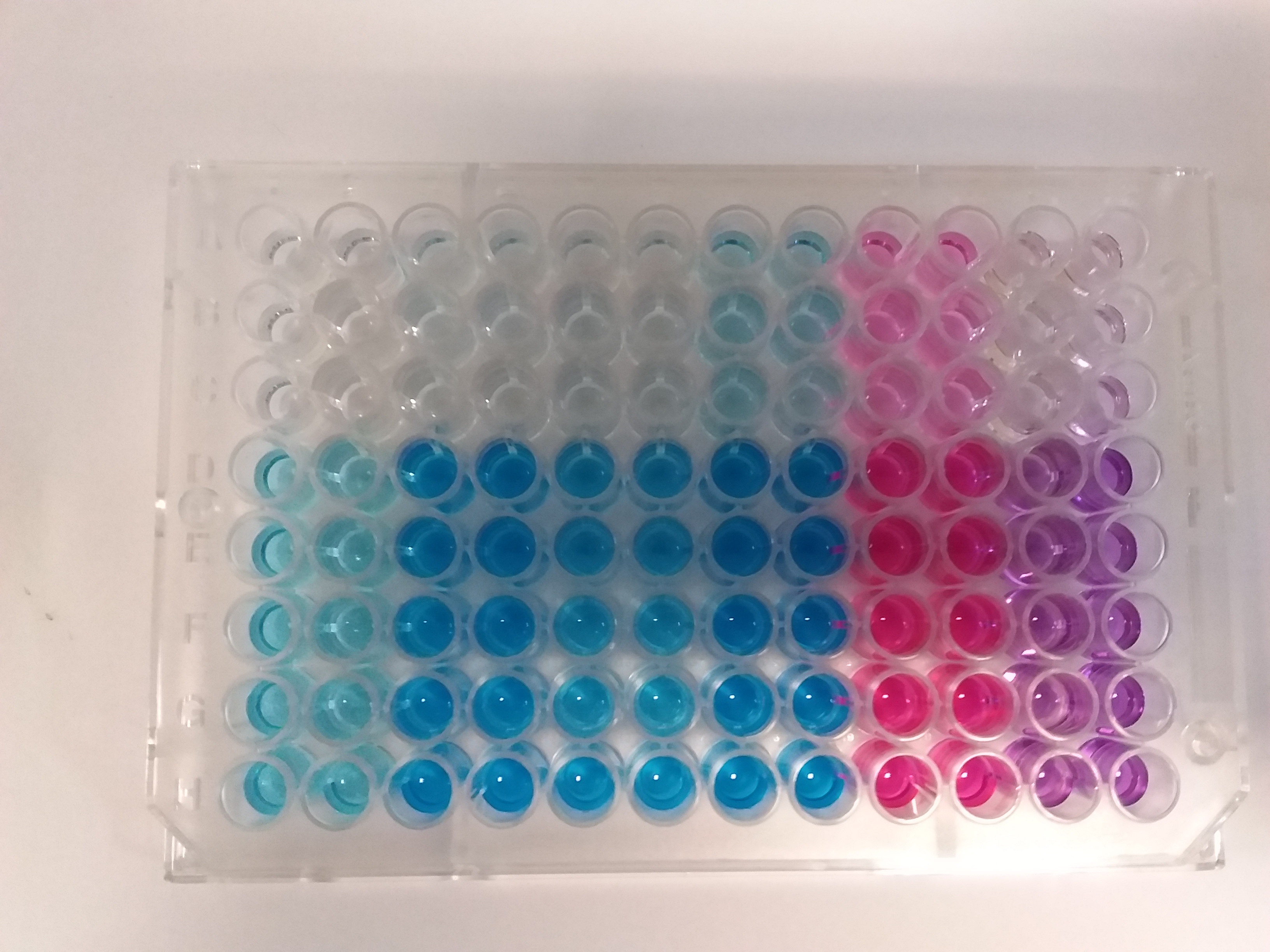

Supplement: Supplementary file 2 — Supplementary Information 2. [file 41598_2023_36721_MOESM2_ESM.zip › Raw data/Plate photos/20201009_175539.jpg]

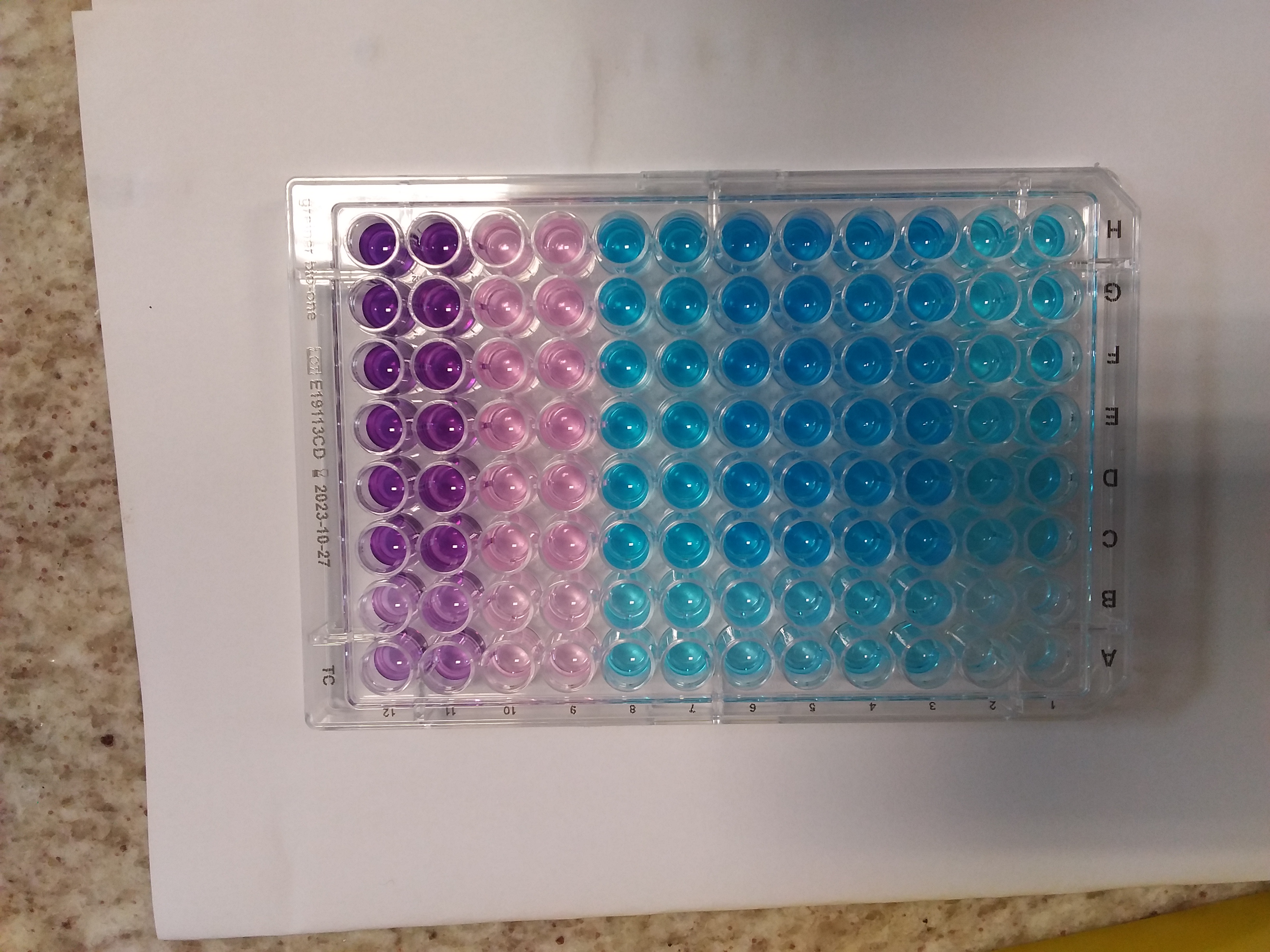

Supplement: Supplementary file 2 — Supplementary Information 2. [file 41598_2023_36721_MOESM2_ESM.zip › Raw data/Plate photos/20201020_164813.jpg]

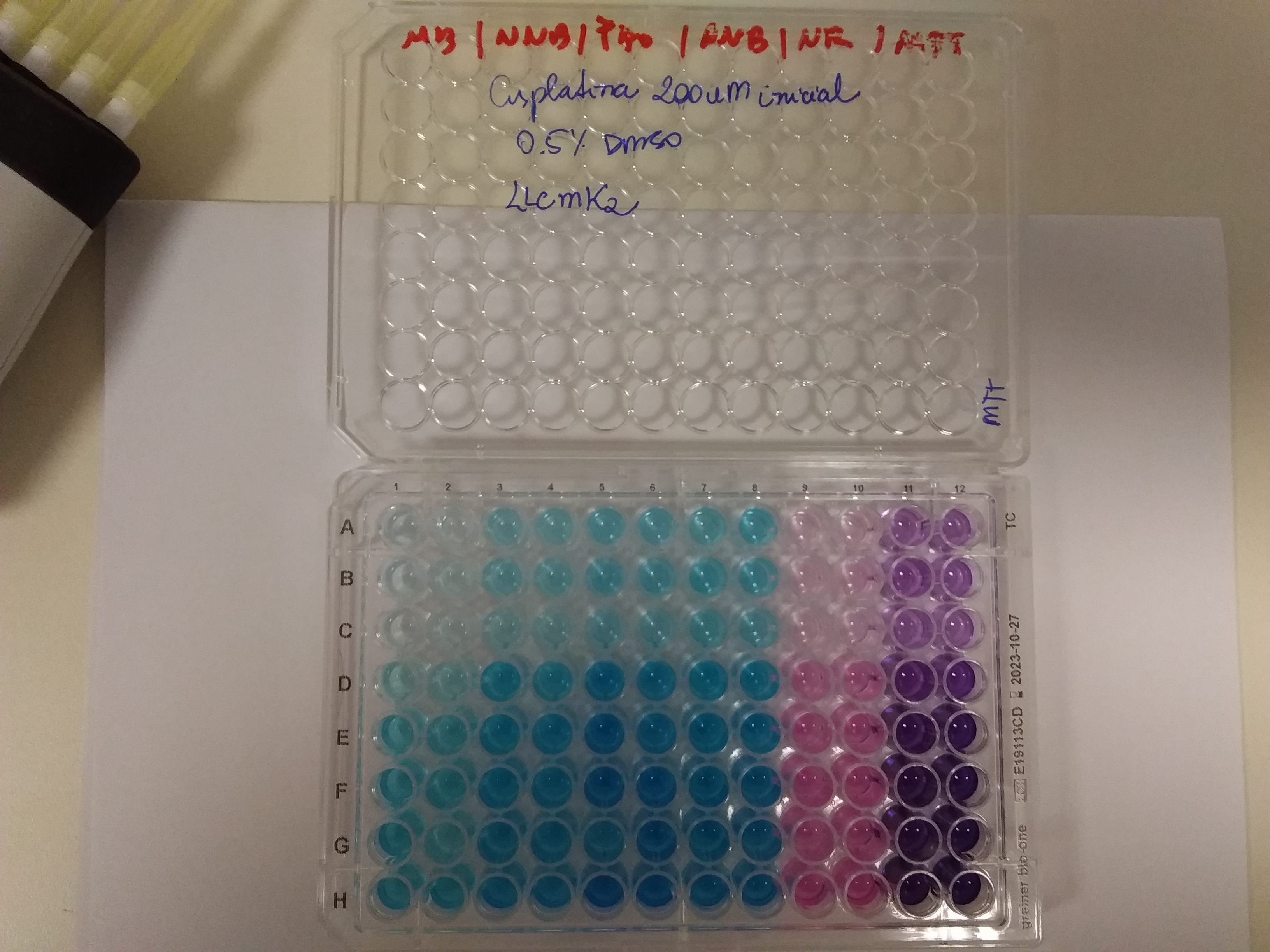

Supplement: Supplementary file 2 — Supplementary Information 2. [file 41598_2023_36721_MOESM2_ESM.zip › Raw data/Plate photos/20201109_181217.jpg]

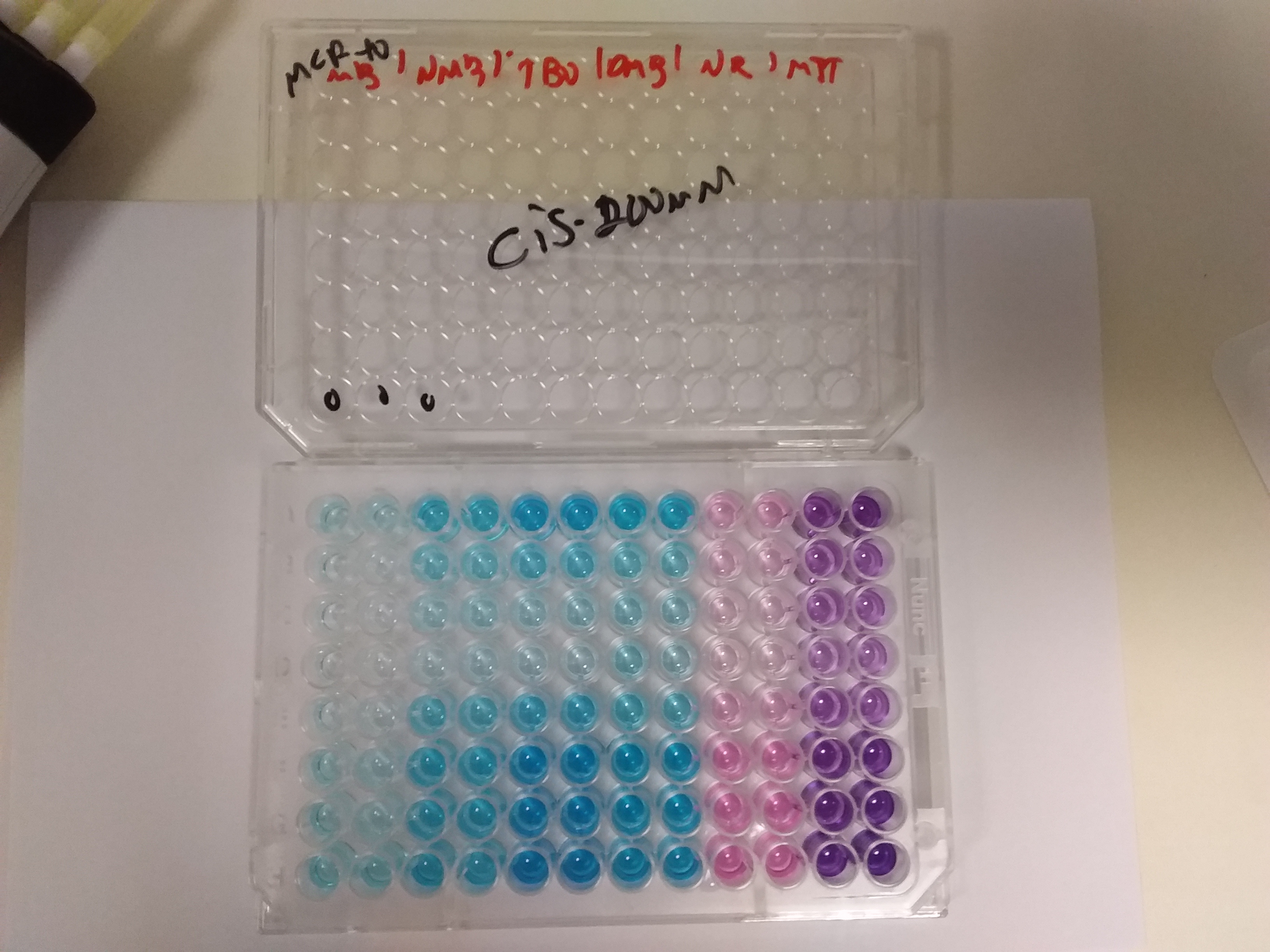

Supplement: Supplementary file 2 — Supplementary Information 2. [file 41598_2023_36721_MOESM2_ESM.zip › Raw data/Plate photos/20201109_181251.jpg]

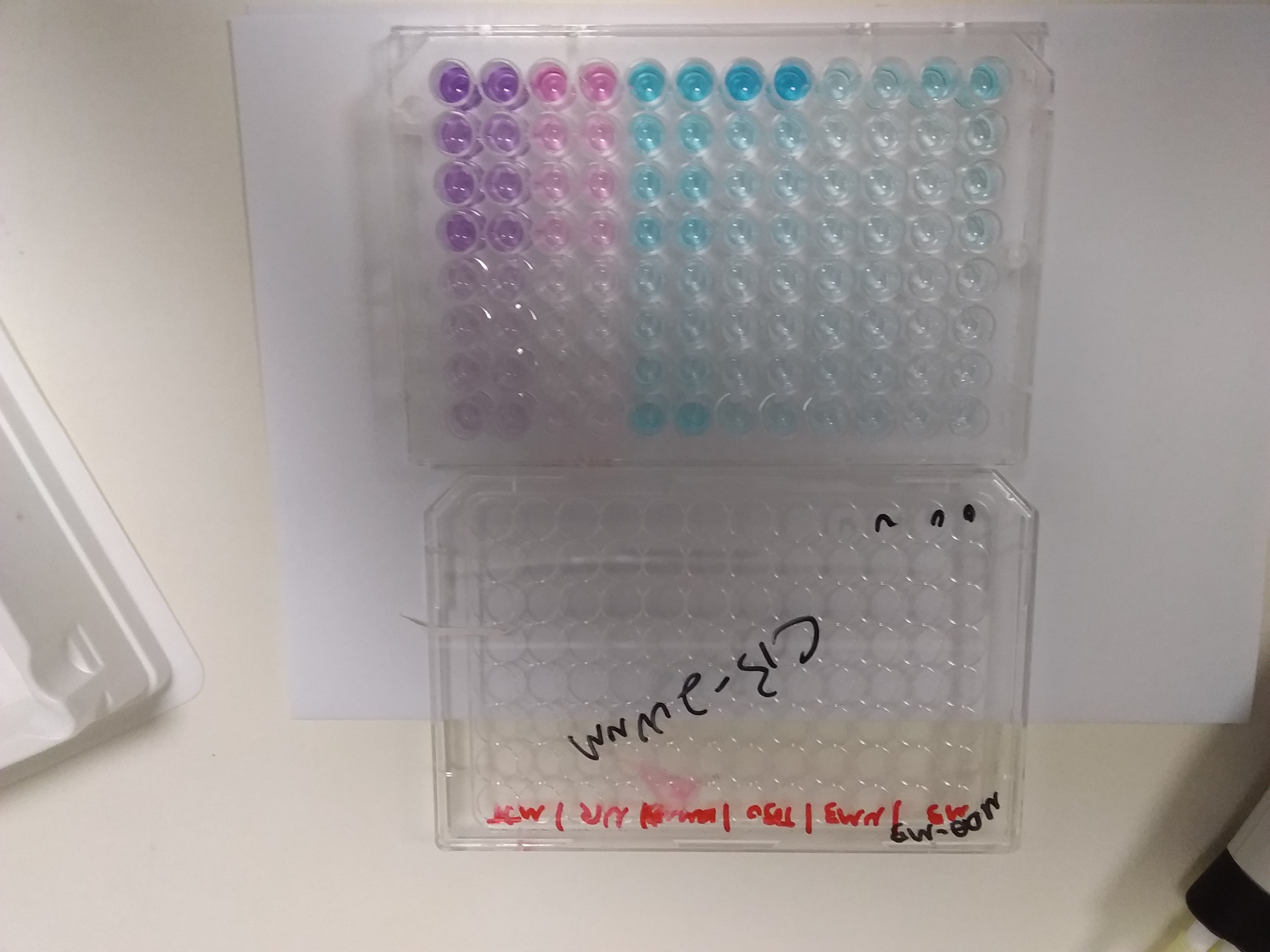

Supplement: Supplementary file 2 — Supplementary Information 2. [file 41598_2023_36721_MOESM2_ESM.zip › Raw data/Plate photos/20201109_181522.jpg]

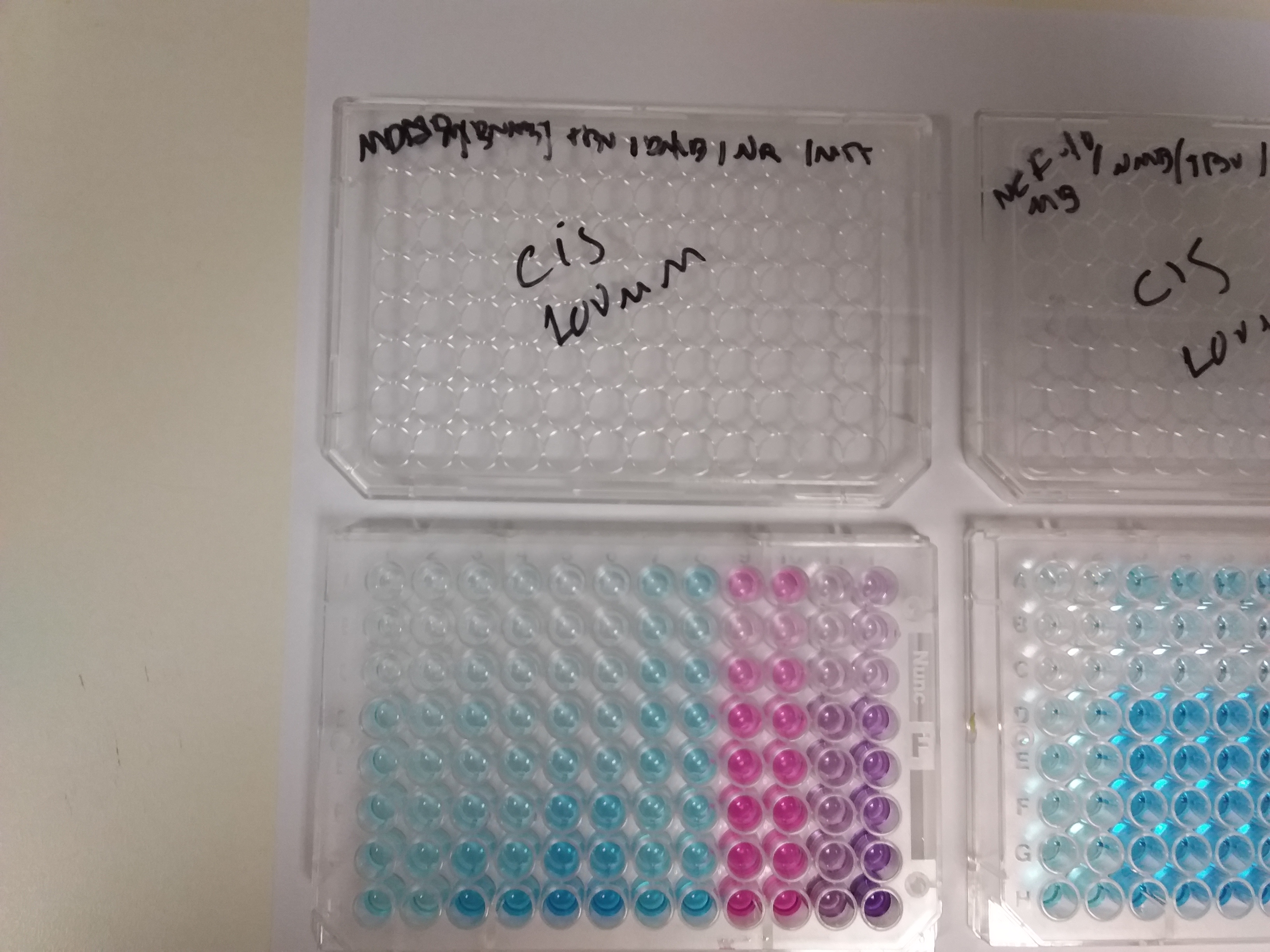

Supplement: Supplementary file 2 — Supplementary Information 2. [file 41598_2023_36721_MOESM2_ESM.zip › Raw data/Plate photos/20201112_180829.jpg]

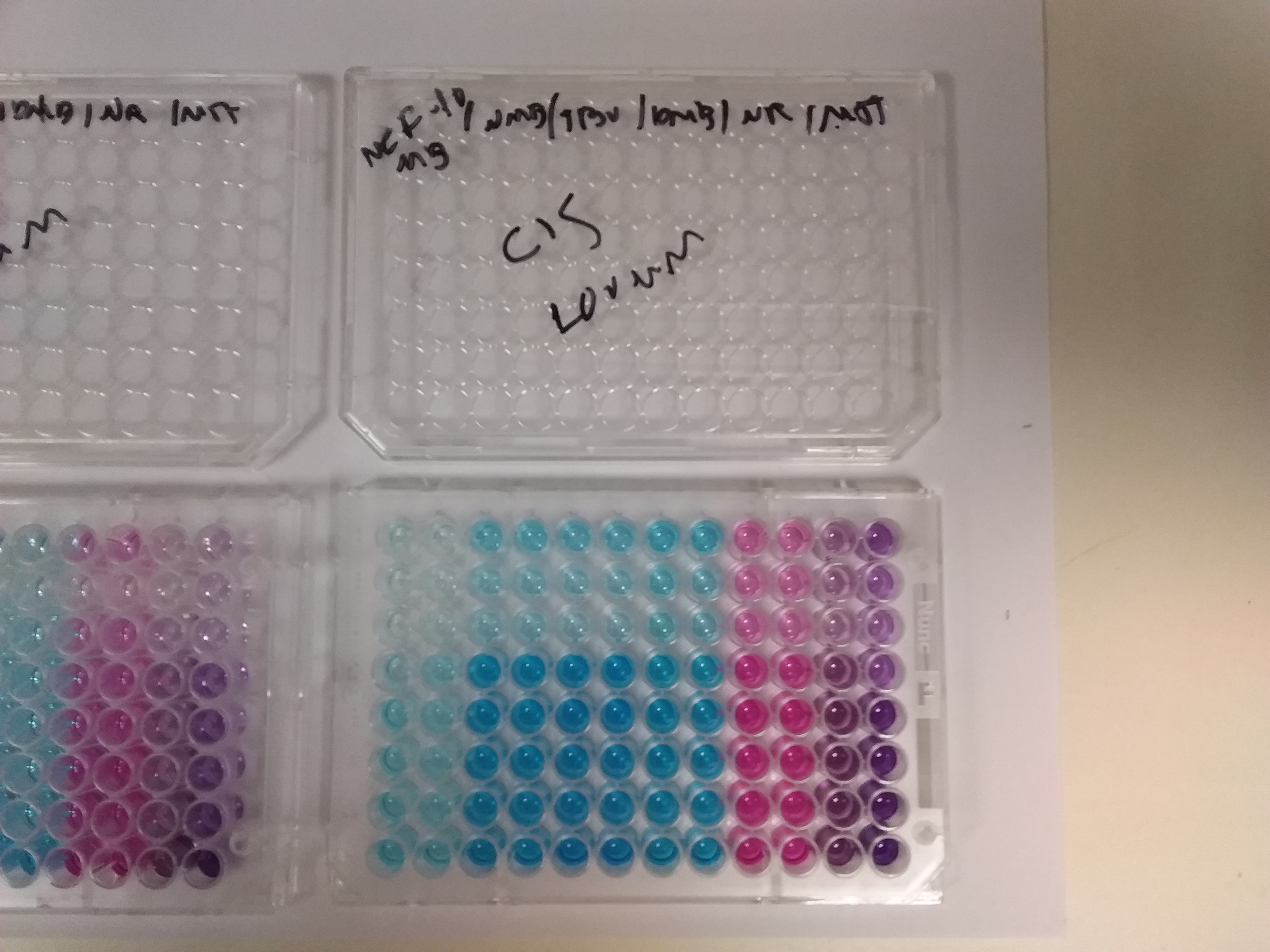

Supplement: Supplementary file 2 — Supplementary Information 2. [file 41598_2023_36721_MOESM2_ESM.zip › Raw data/Plate photos/20201112_180836.jpg]

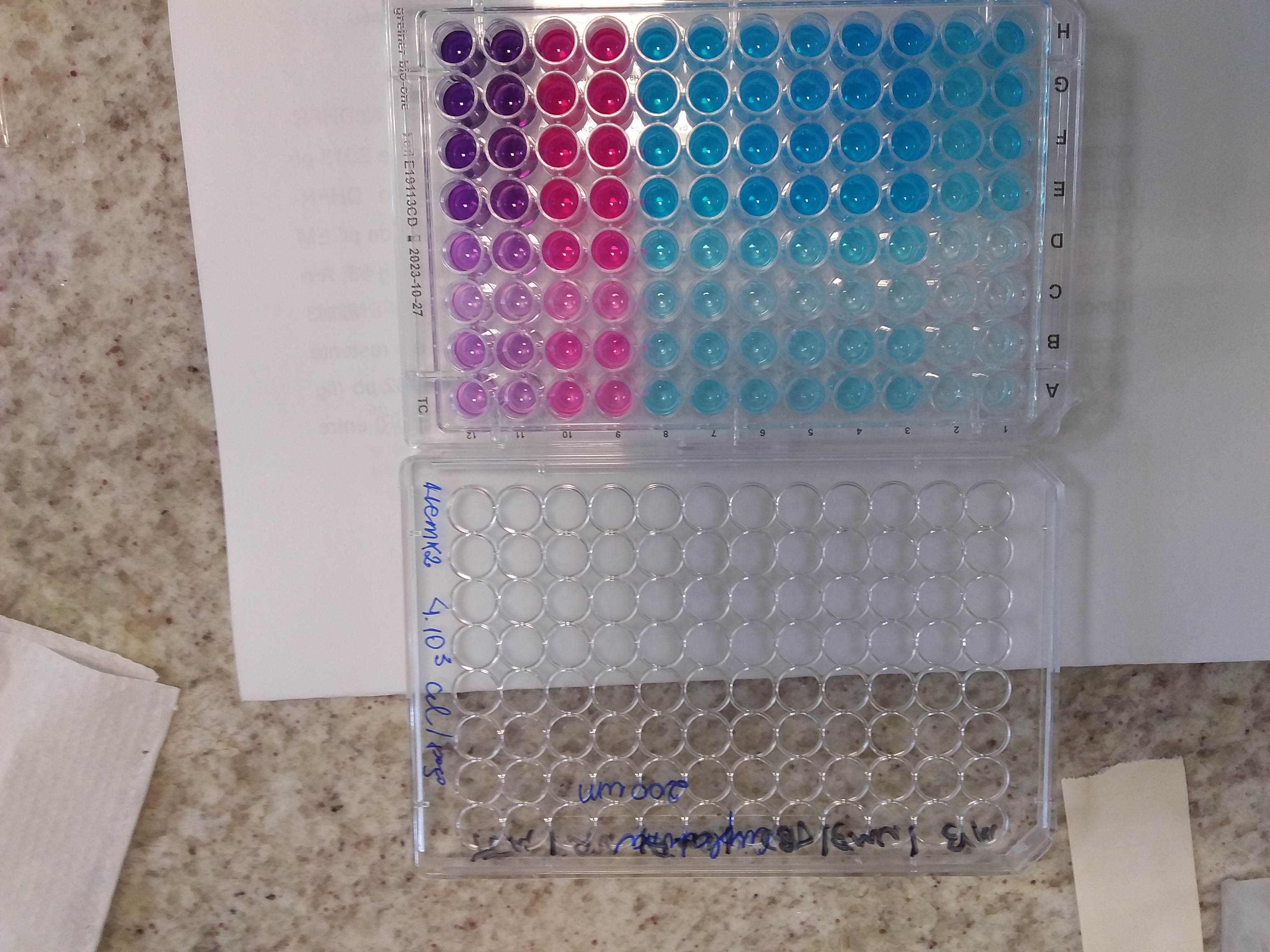

Supplement: Supplementary file 2 — Supplementary Information 2. [file 41598_2023_36721_MOESM2_ESM.zip › Raw data/Plate photos/20201124_155951.jpg]

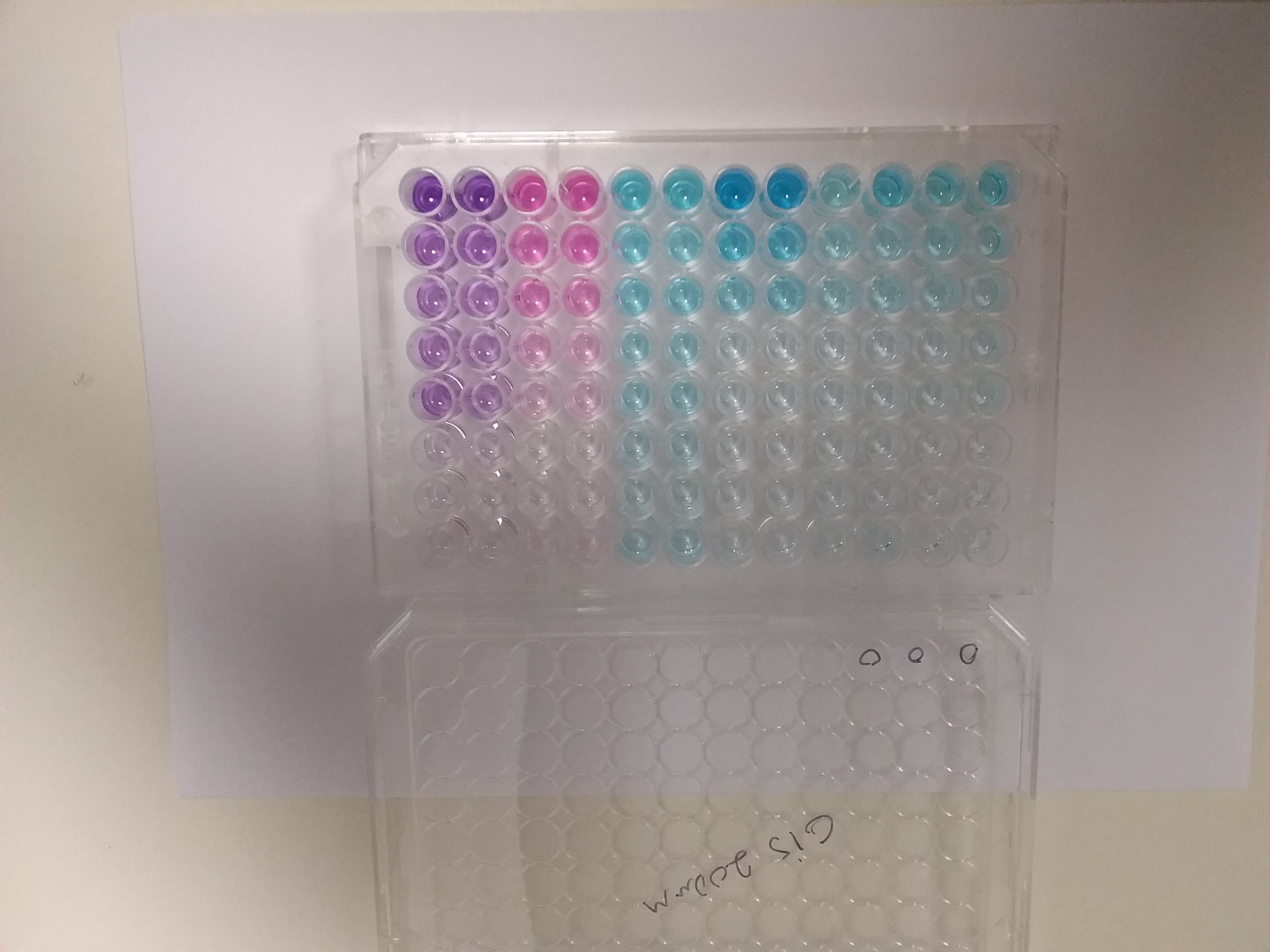

Supplement: Supplementary file 2 — Supplementary Information 2. [file 41598_2023_36721_MOESM2_ESM.zip › Raw data/Plate photos/20201208_174111.jpg]
