# Supplementary material for: The potential of phenothiazinium dyes as cytotoxicity markers in cisplatin-treated cells
Source: Sci Rep. 2023 Jun 23;13:10203. doi: 10.1038/s41598-023-36721-0 (PMC10290130; doi:10.1038/s41598-023-36721-0)
Supplement: Supplementary file 4 — Supplementary Figure 2. [file 41598_2023_36721_MOESM4_ESM.docx]

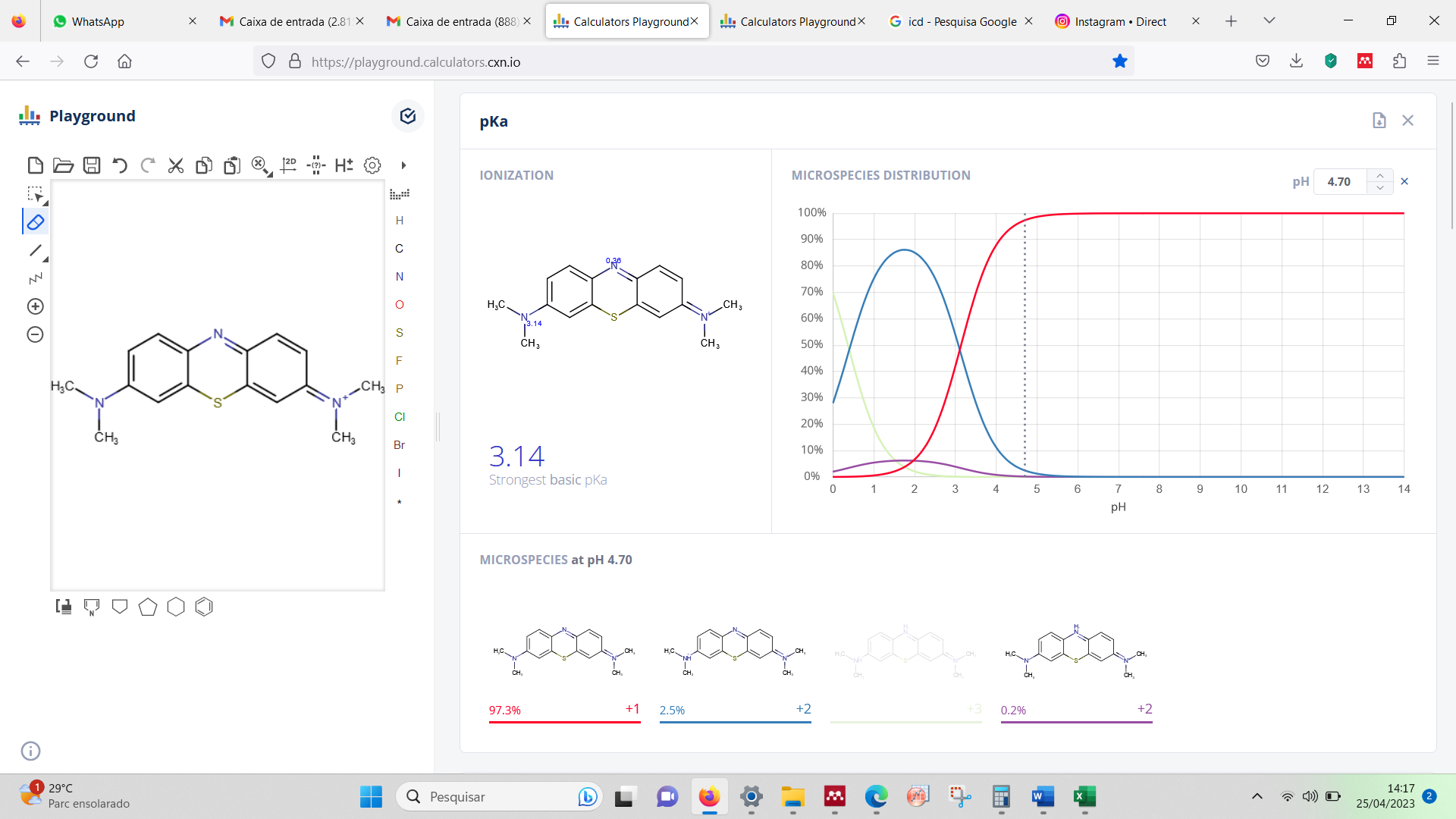


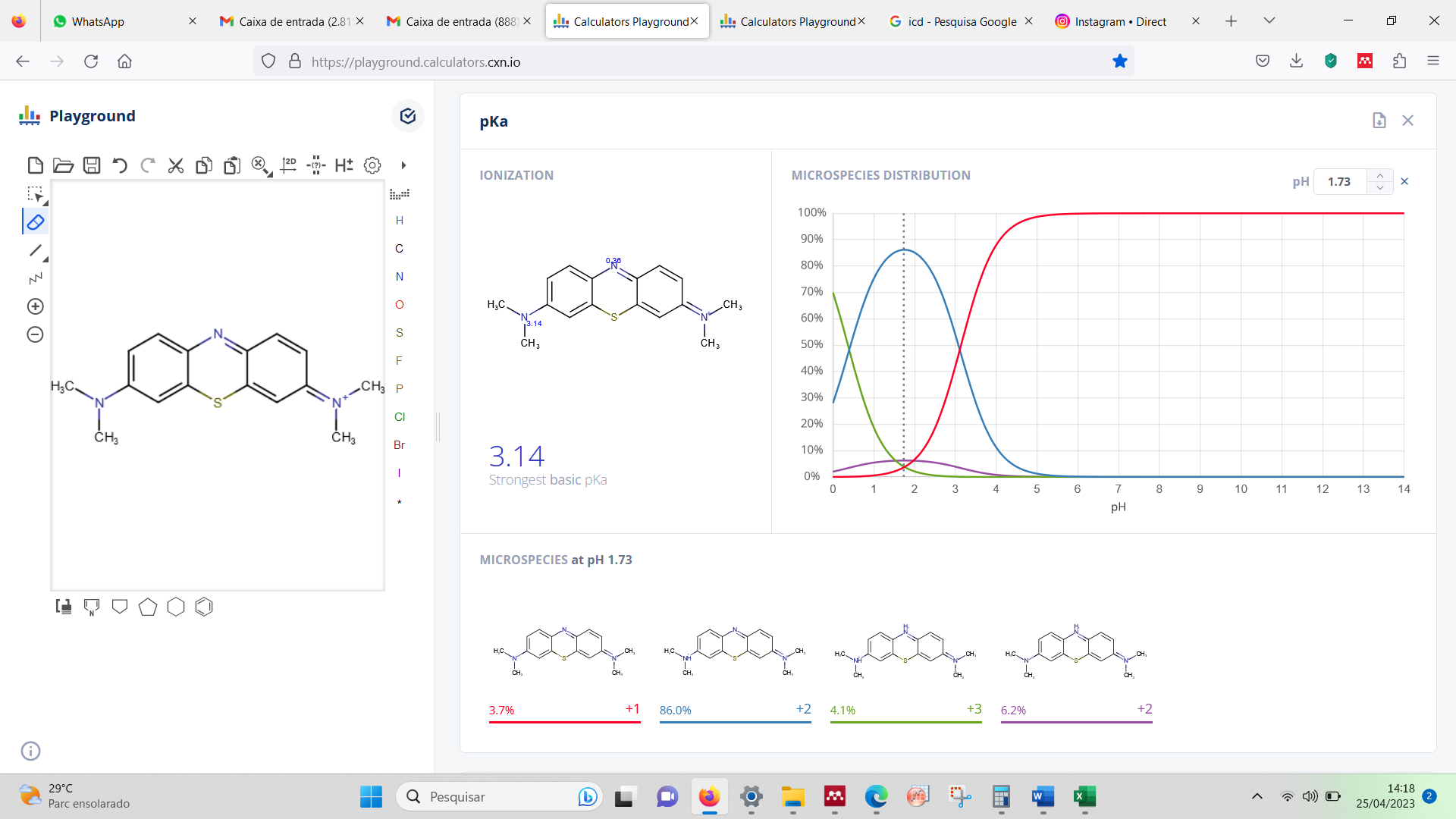


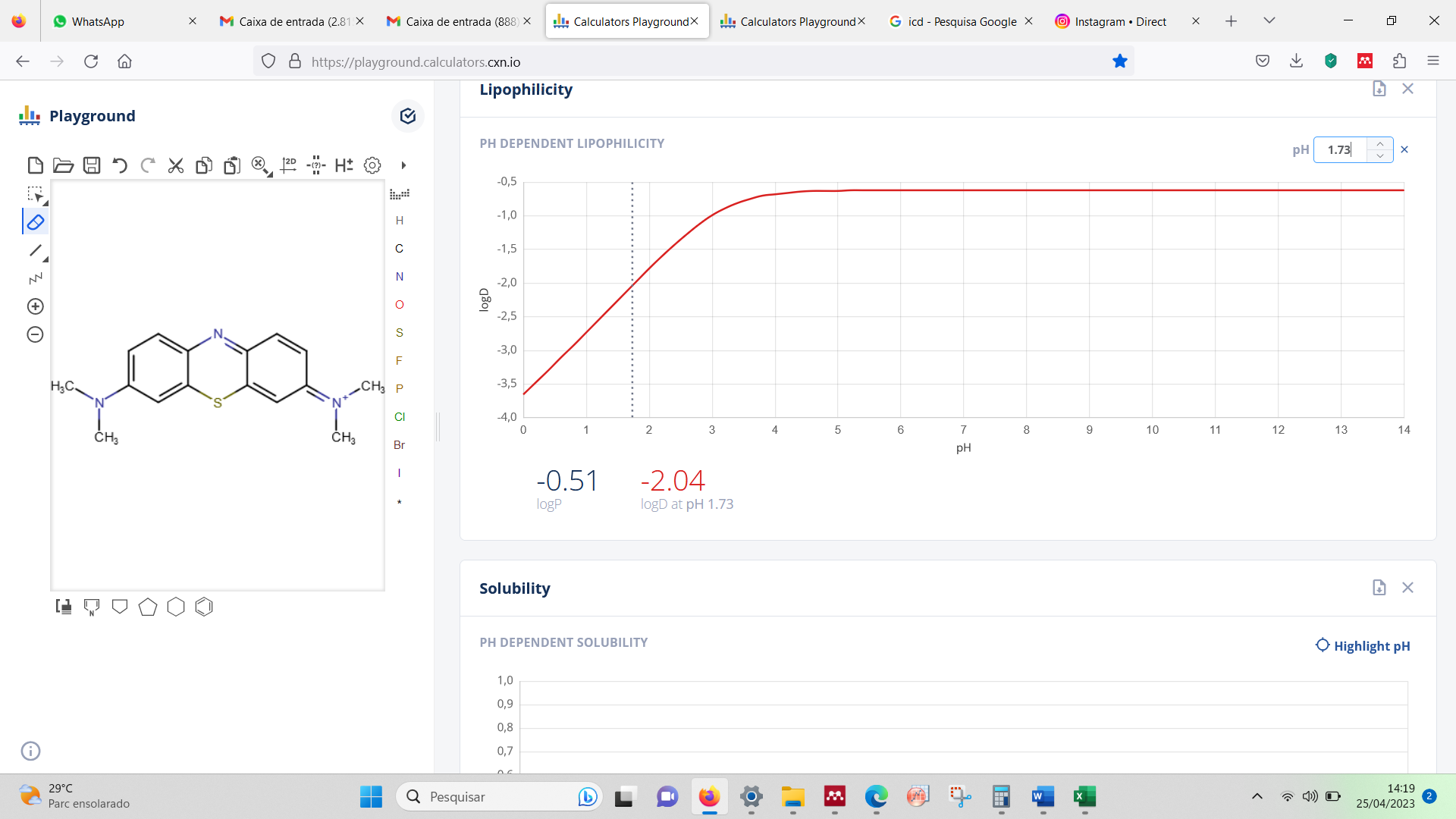


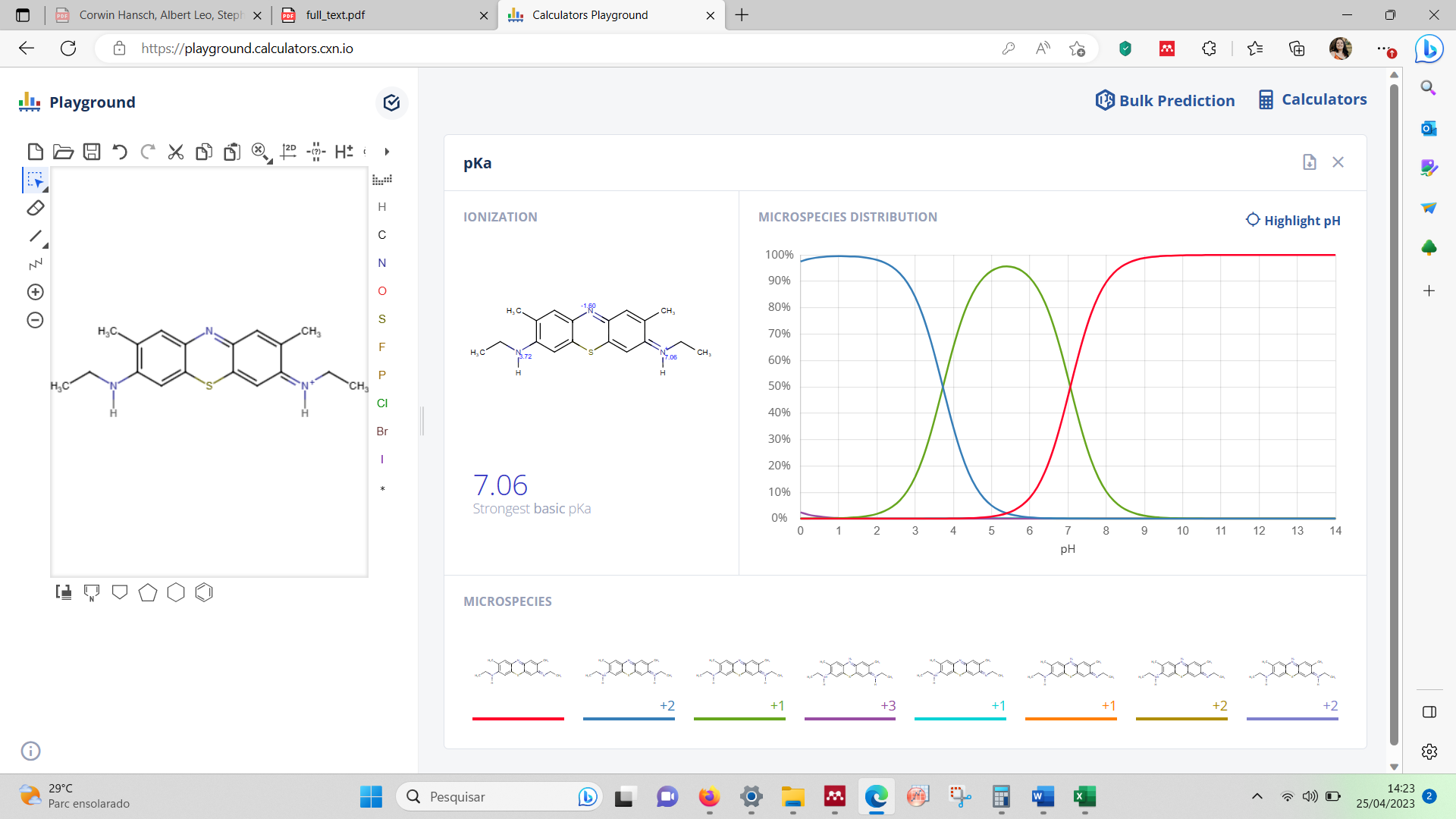


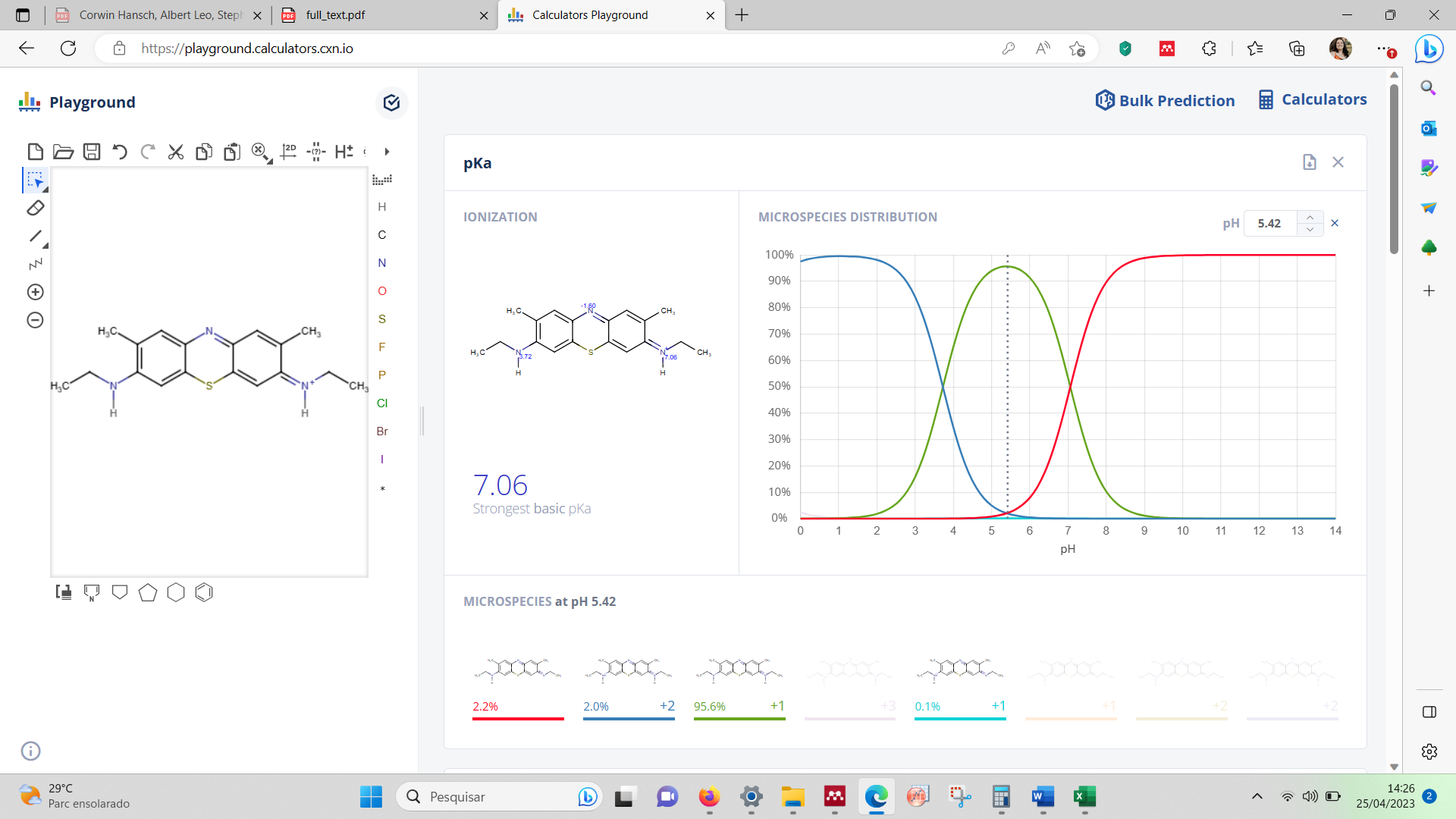


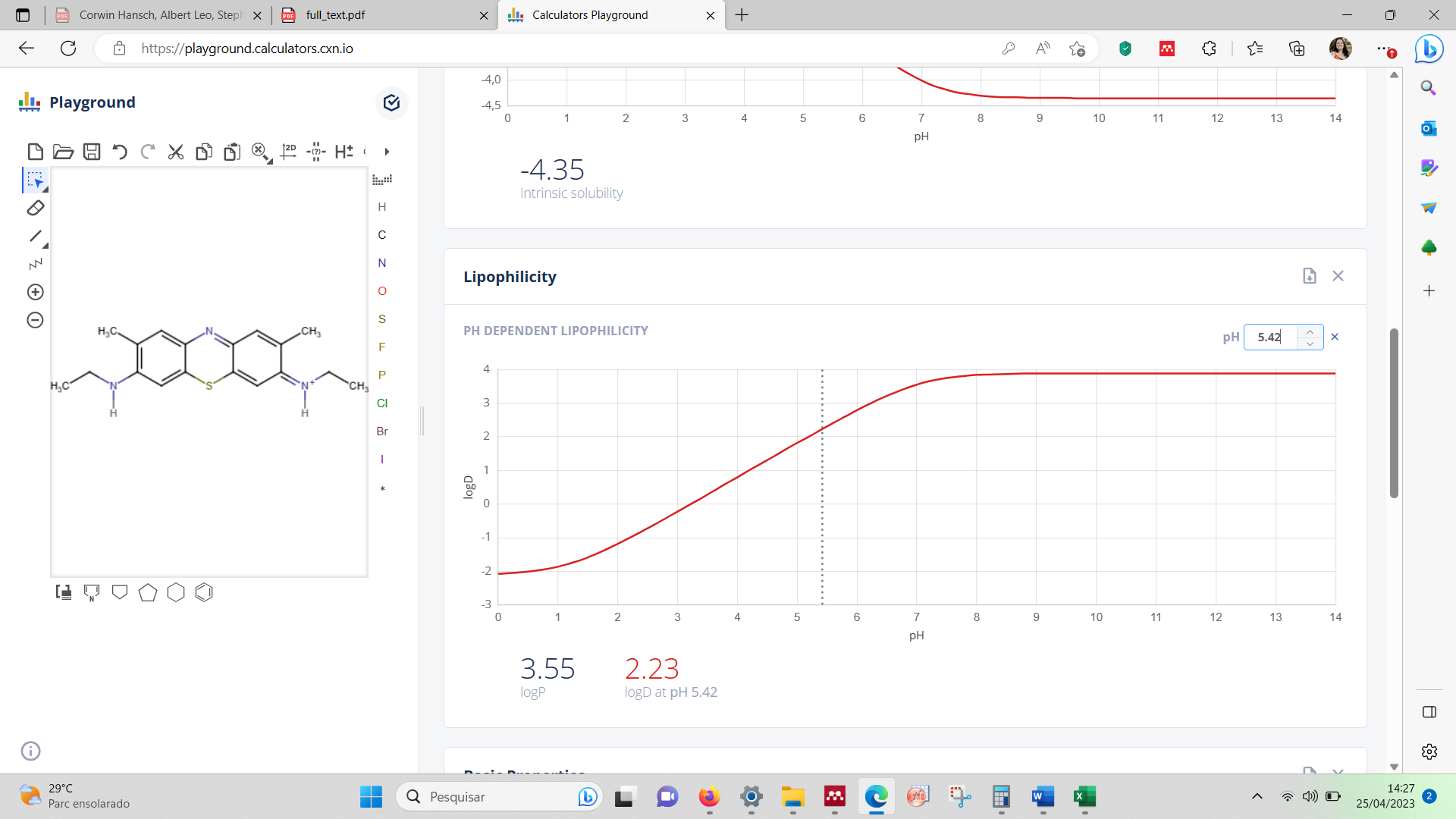


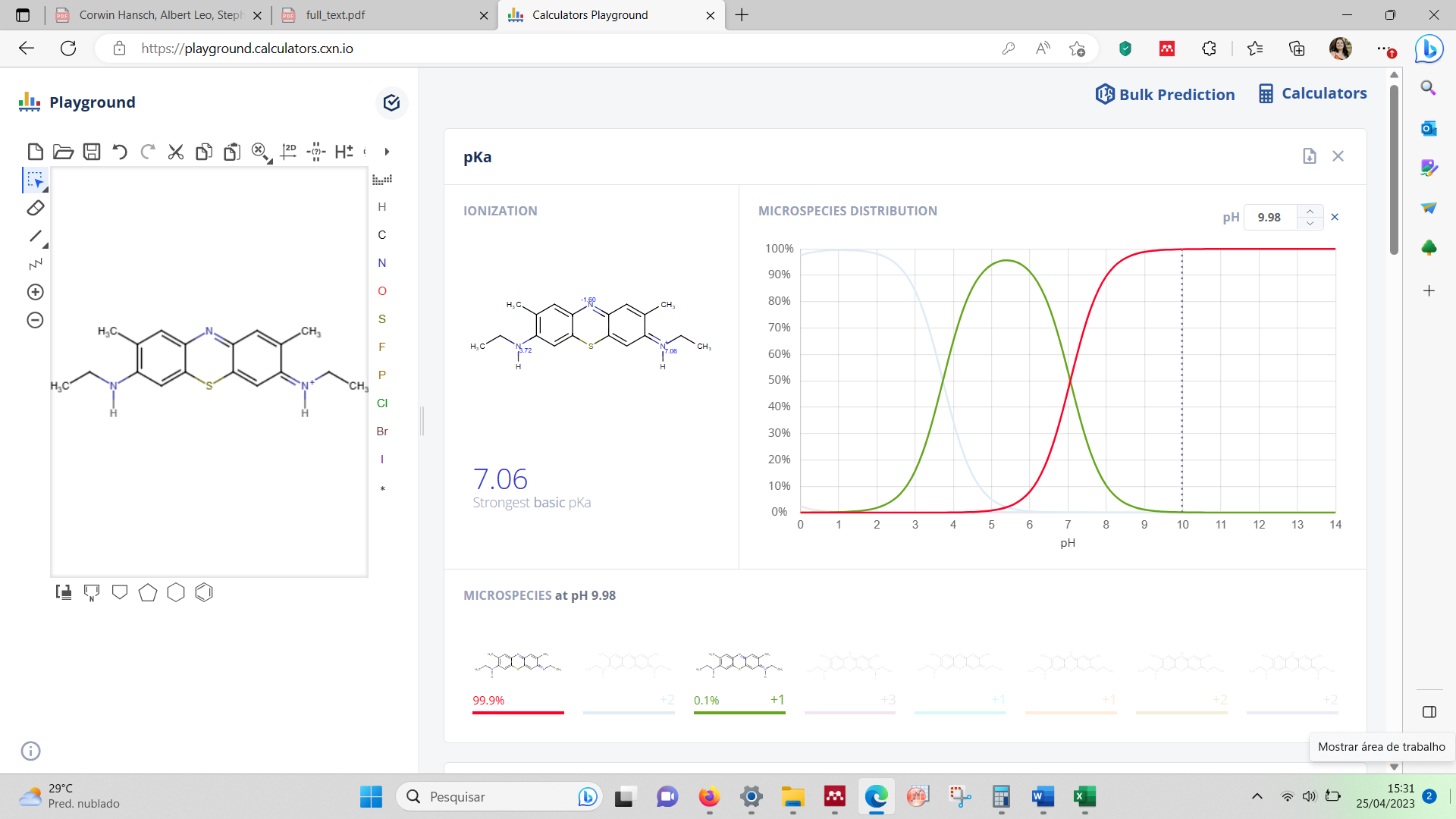


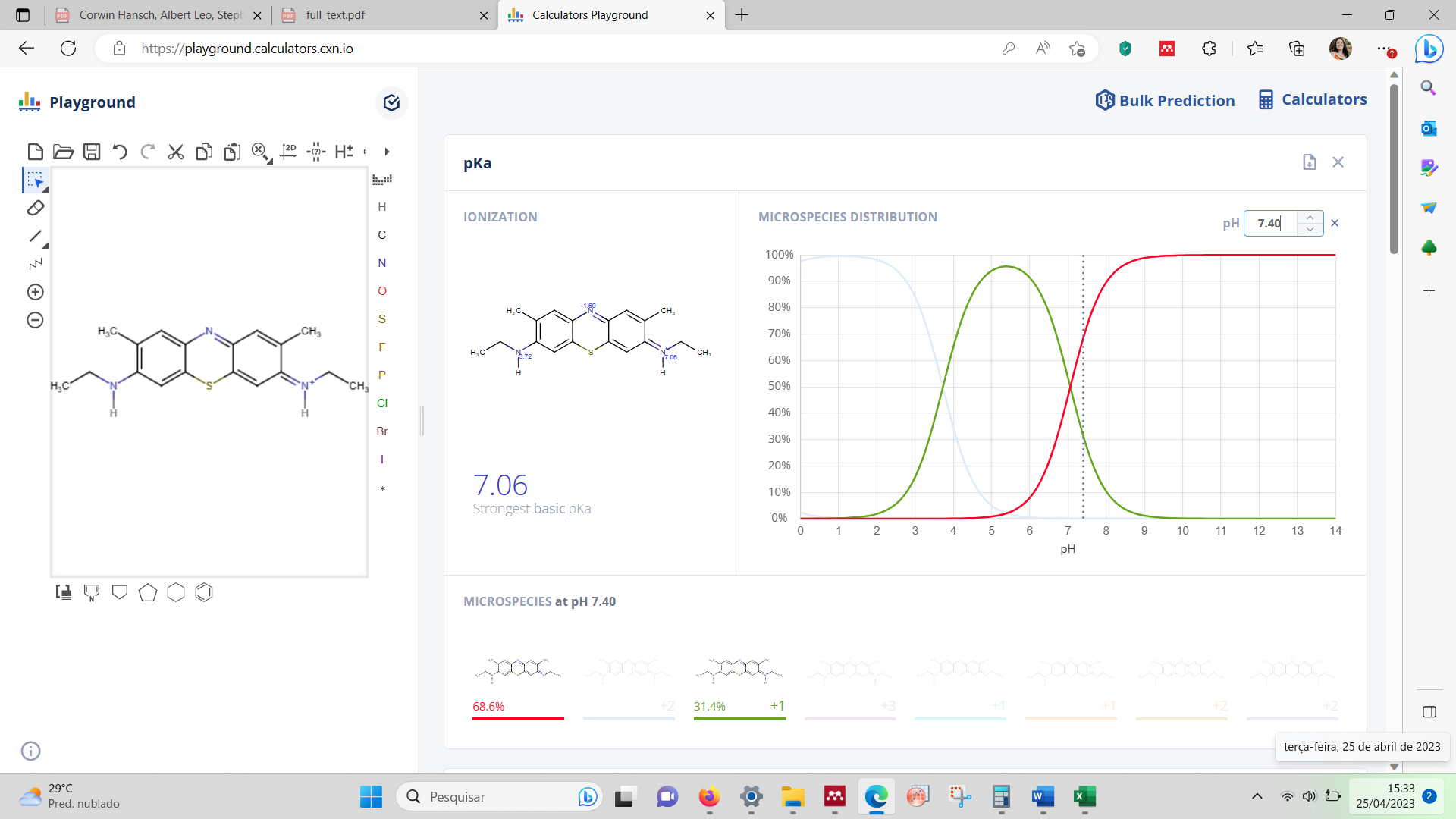


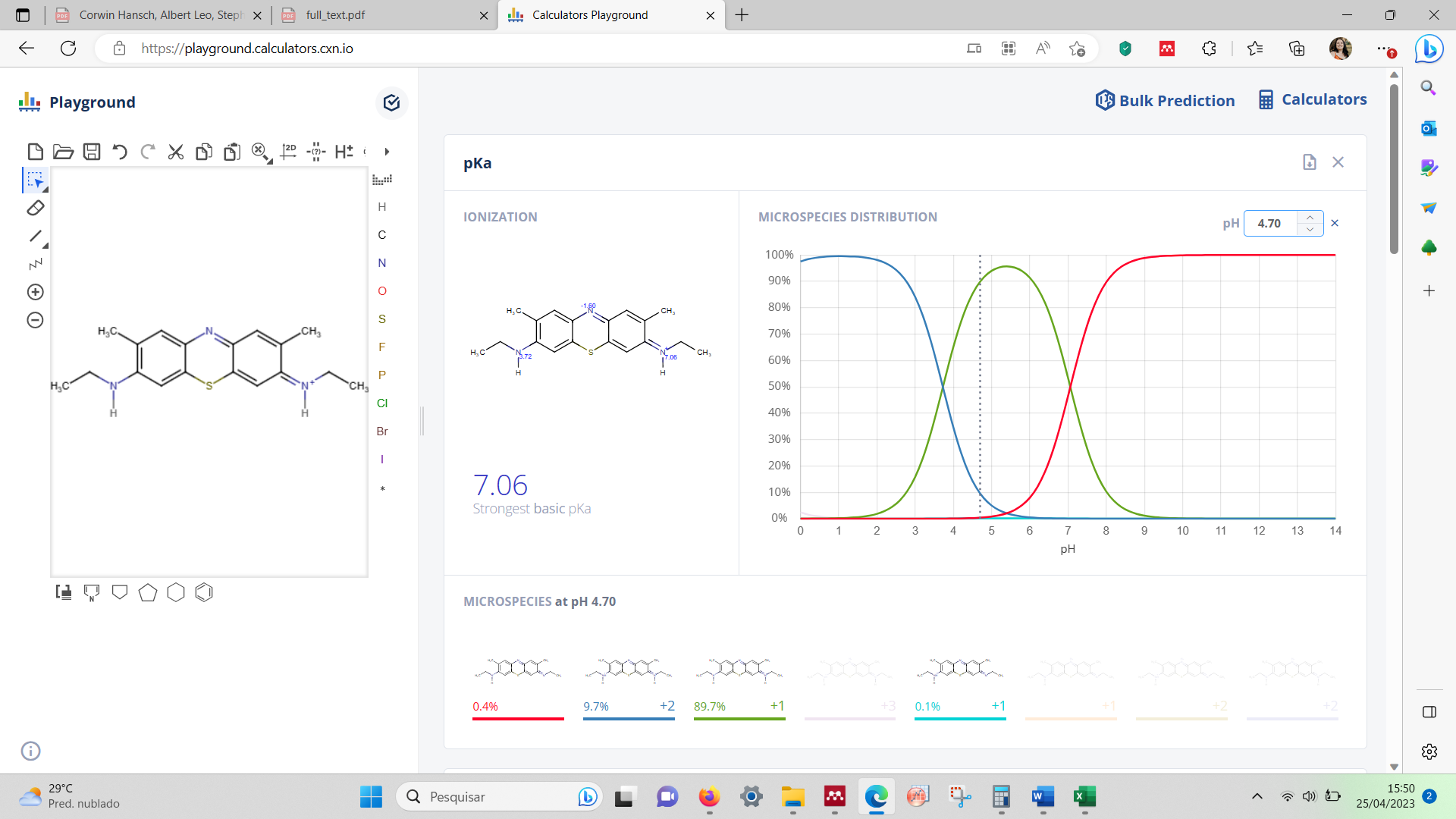


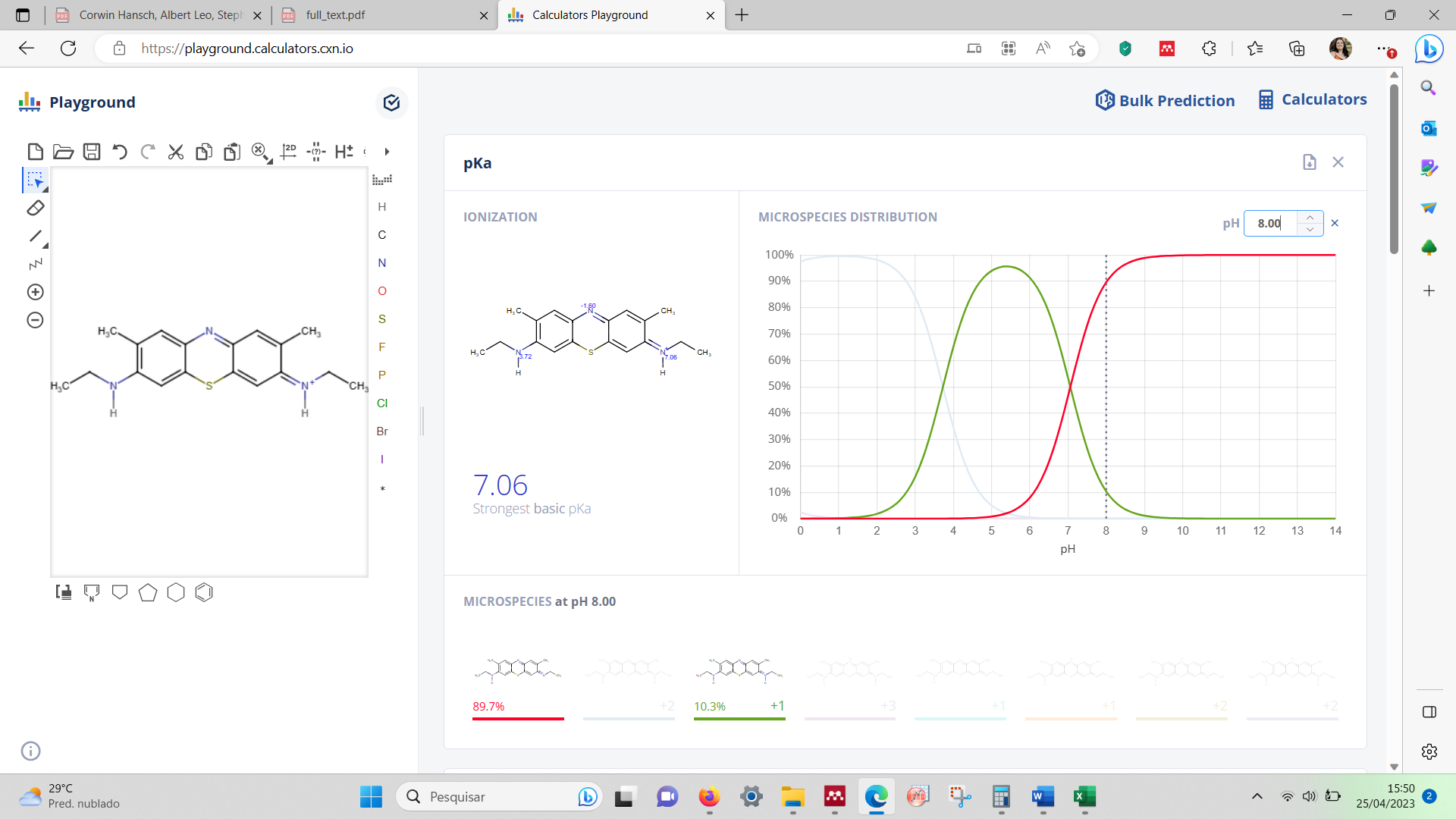


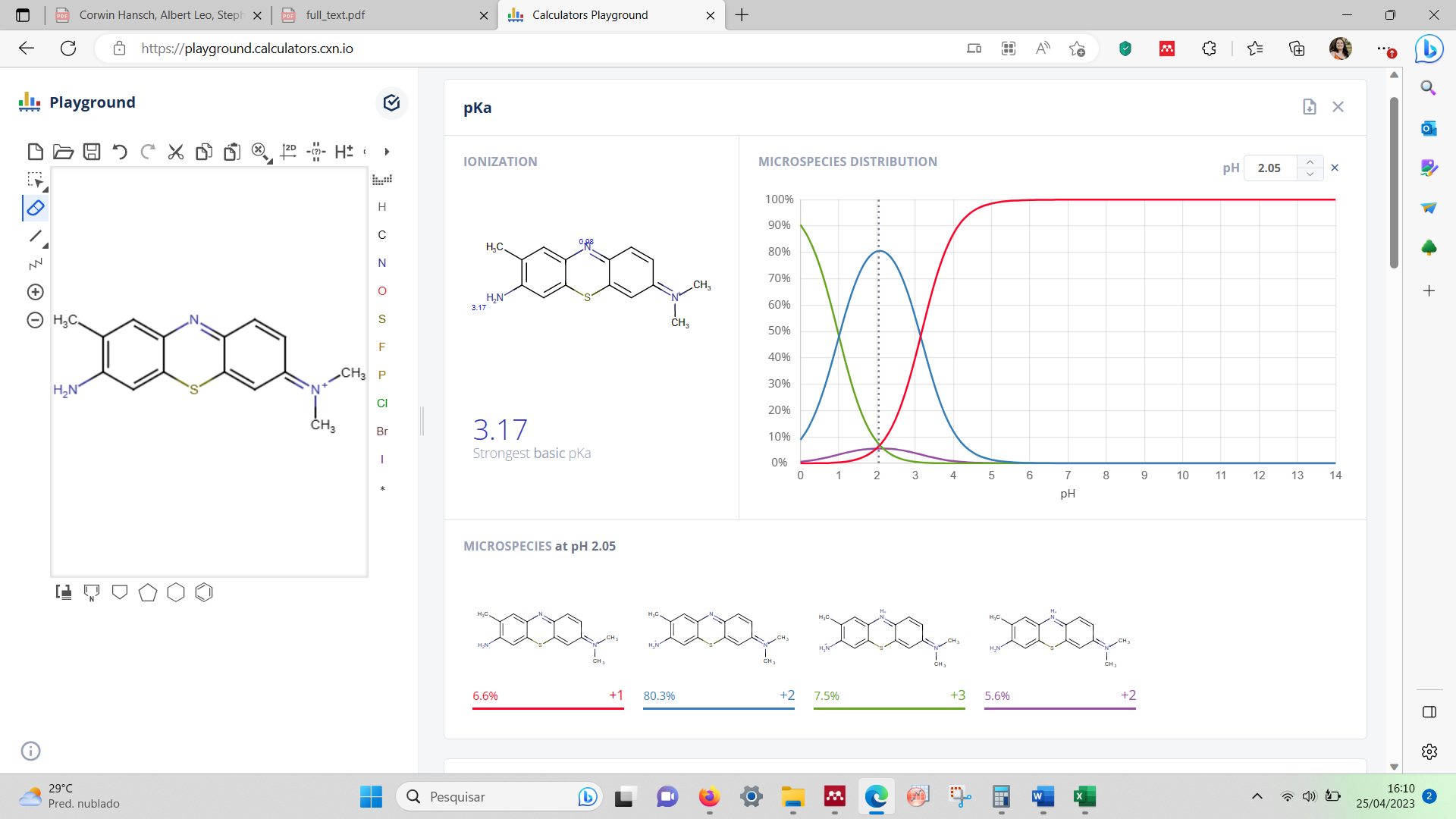


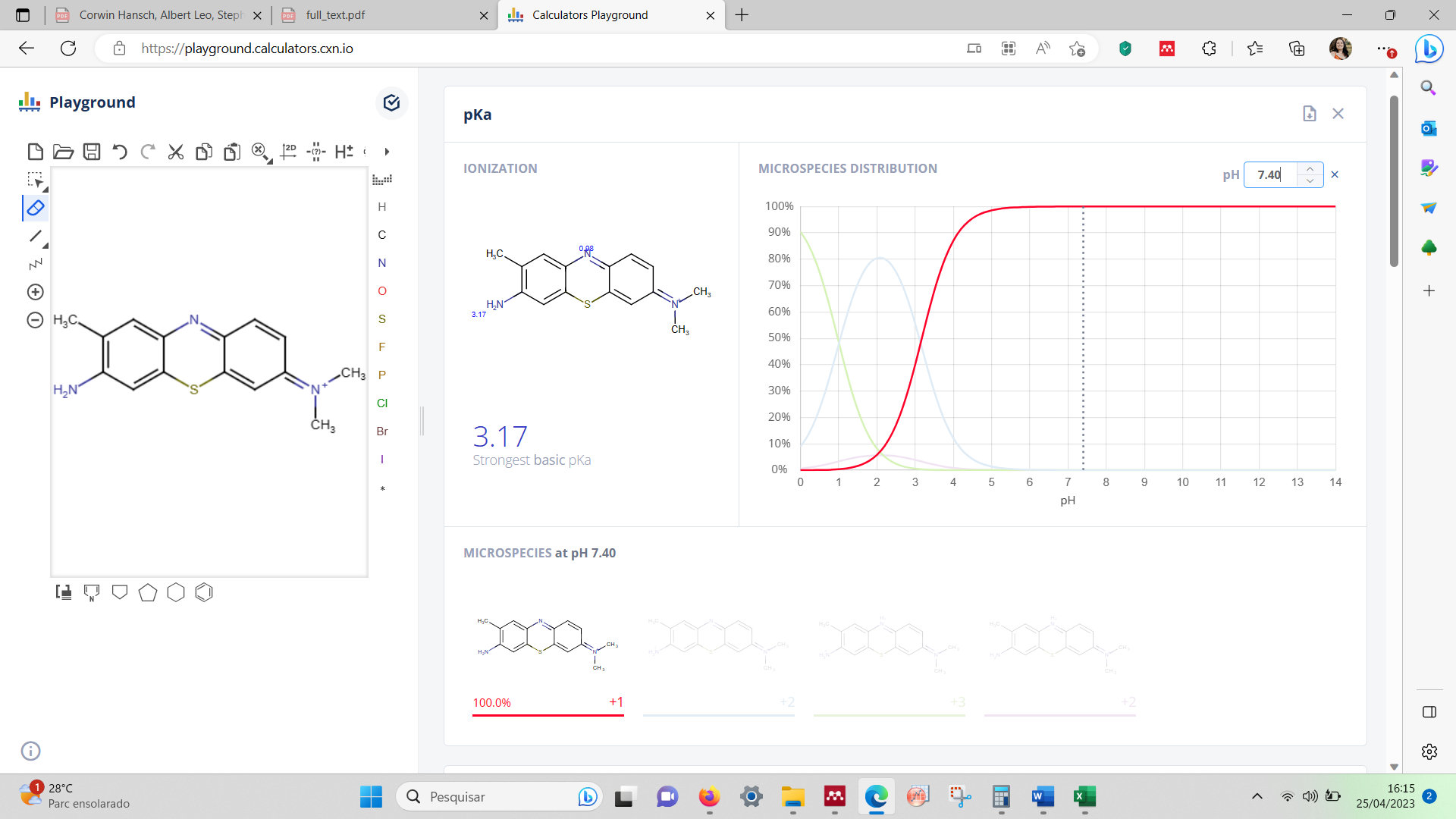


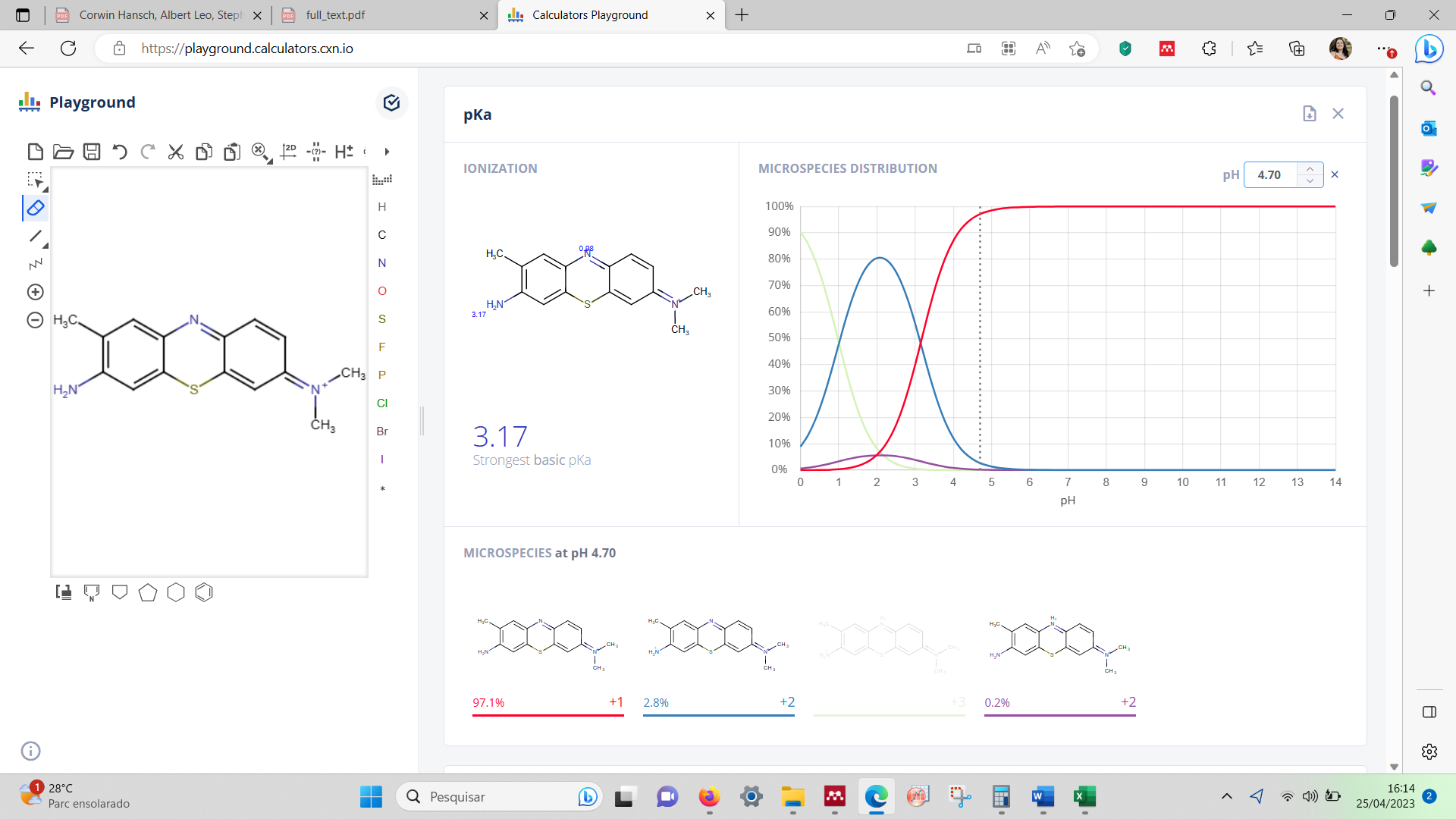


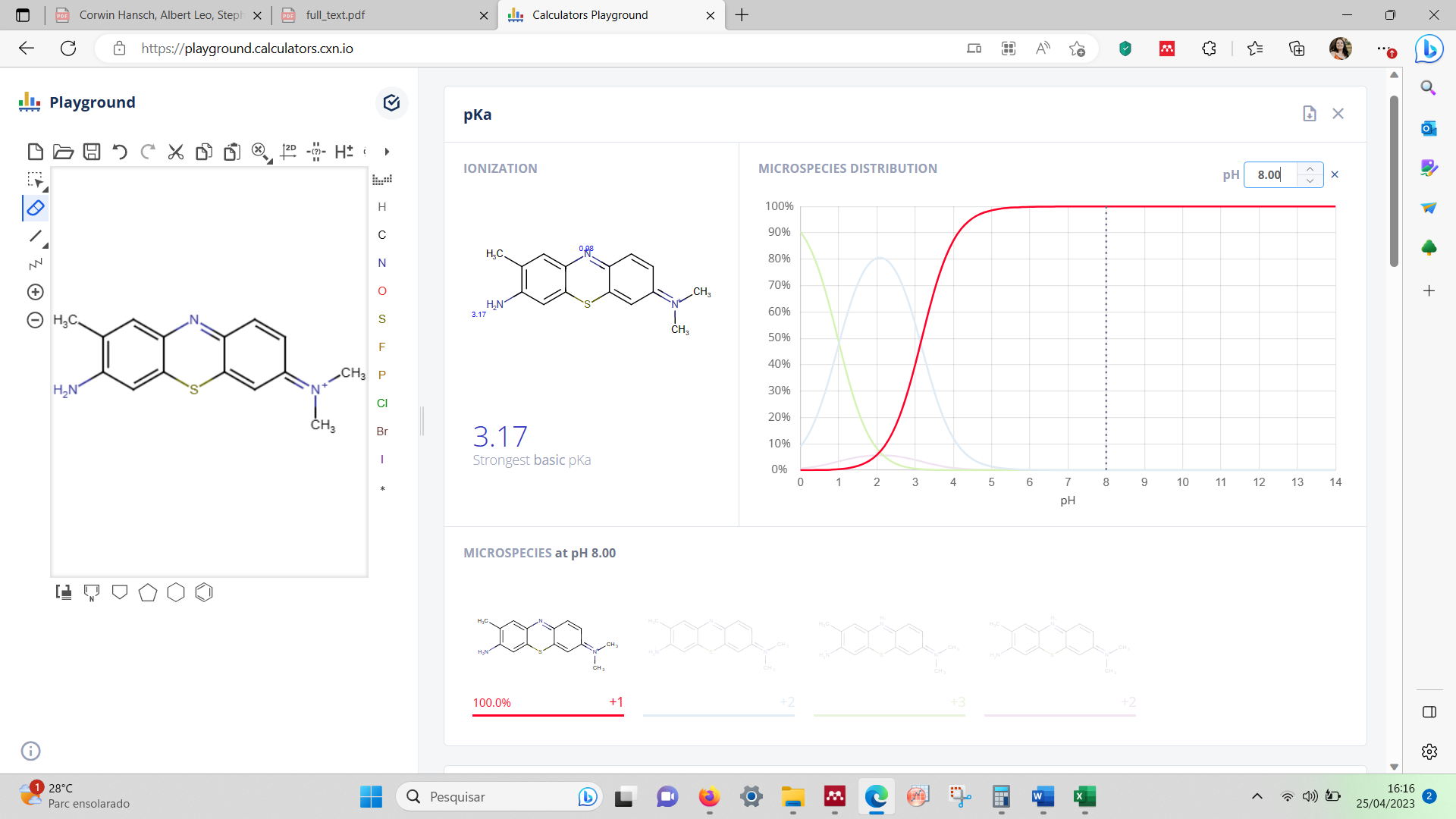


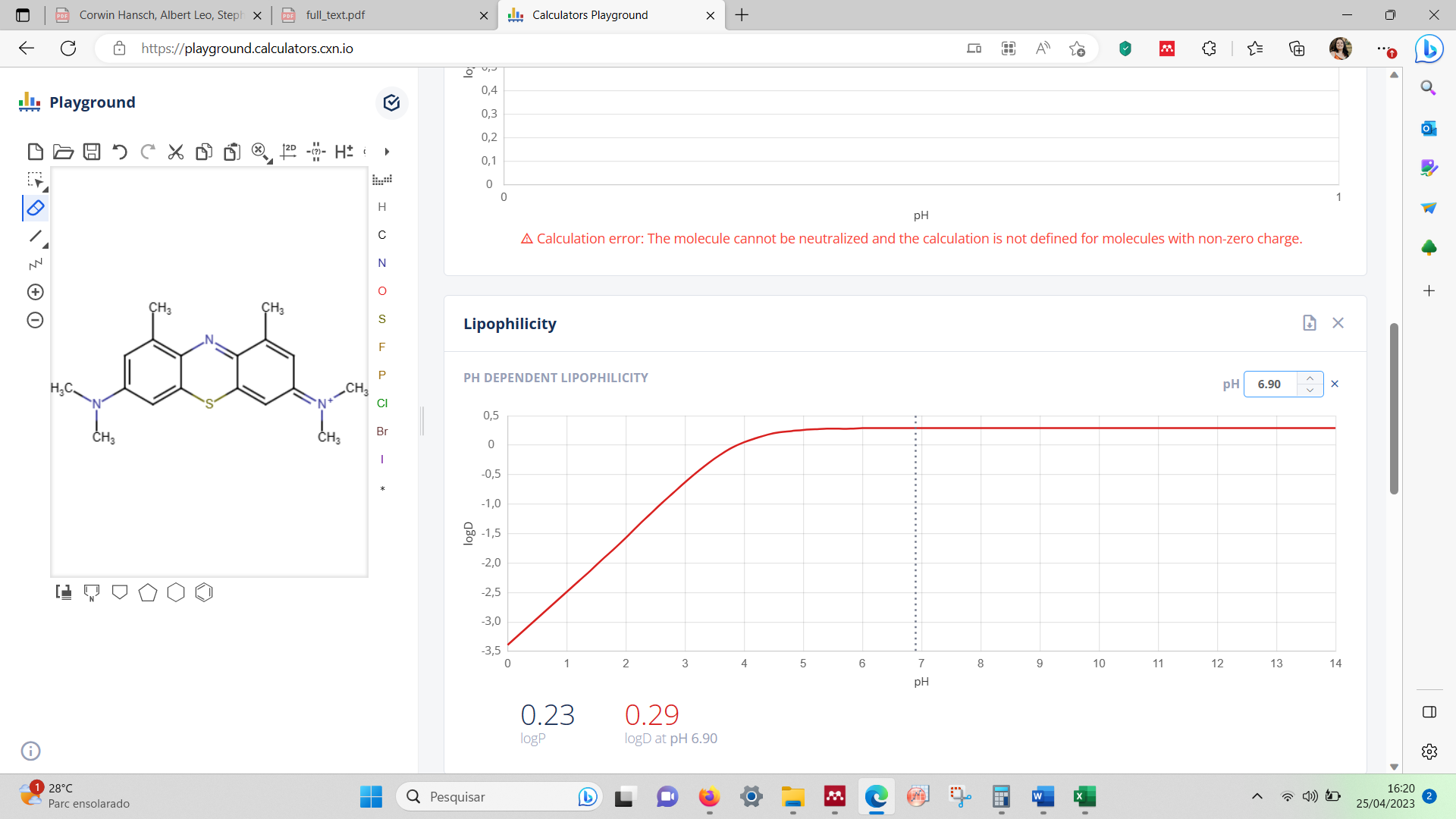


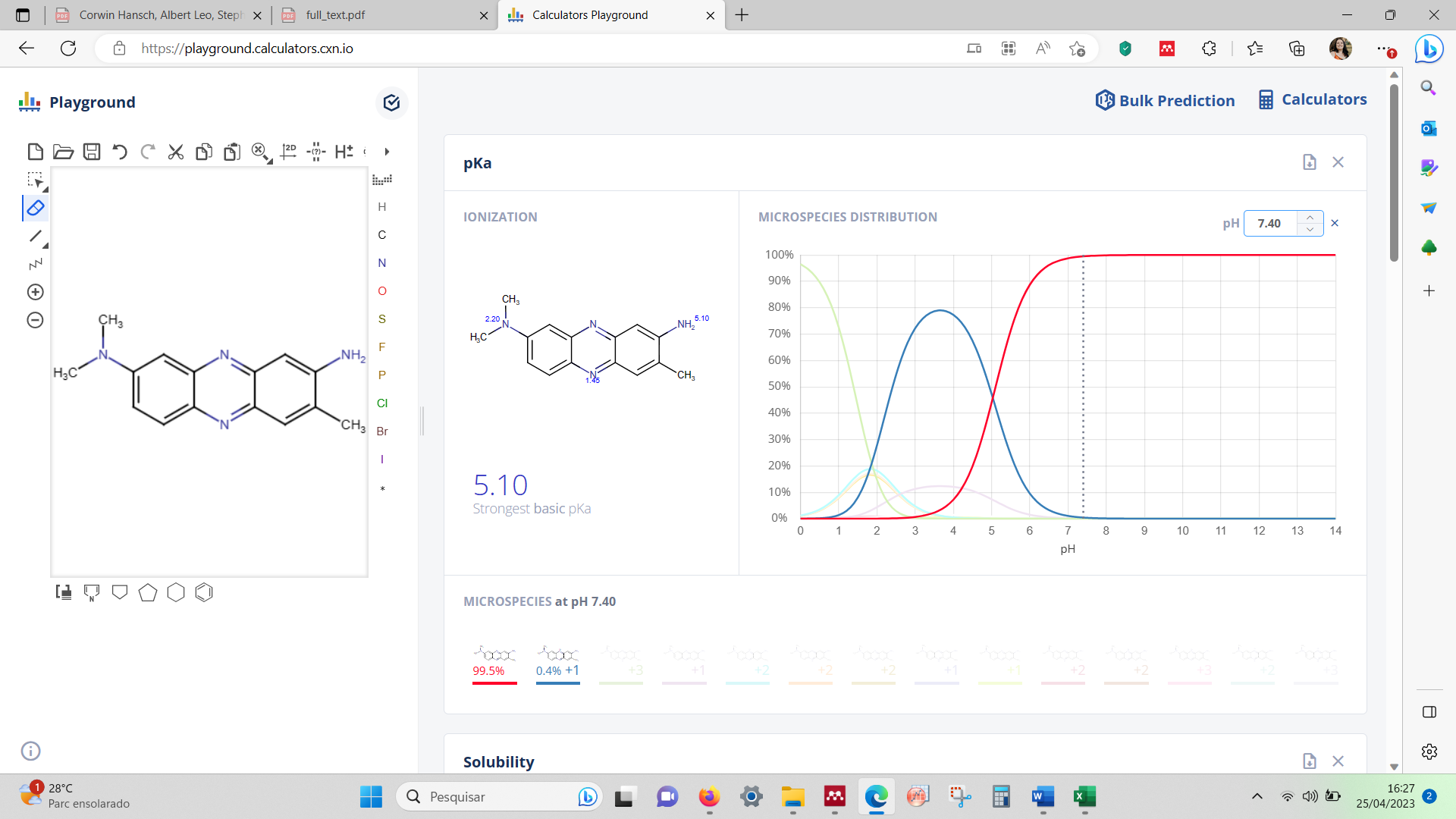


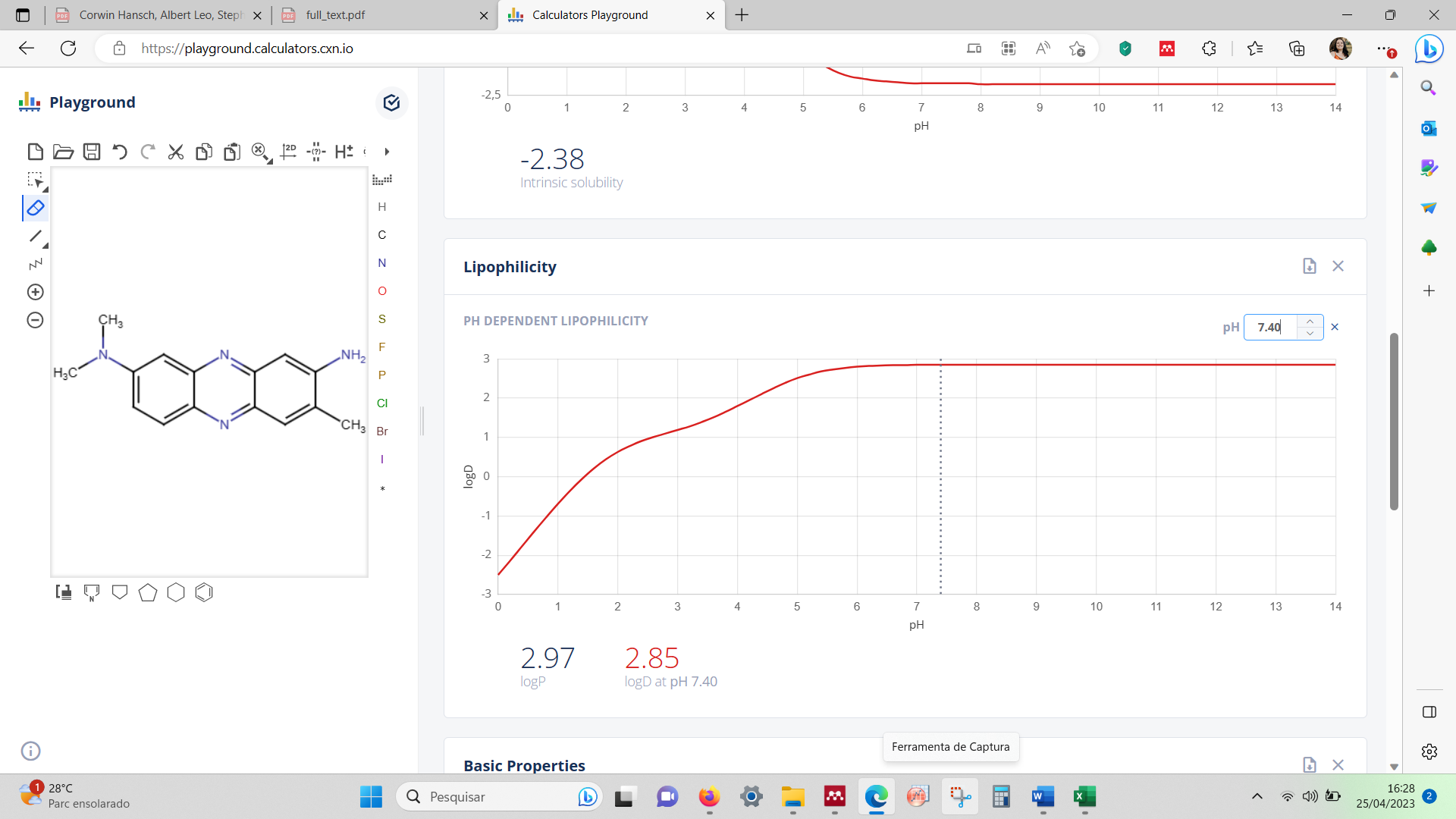


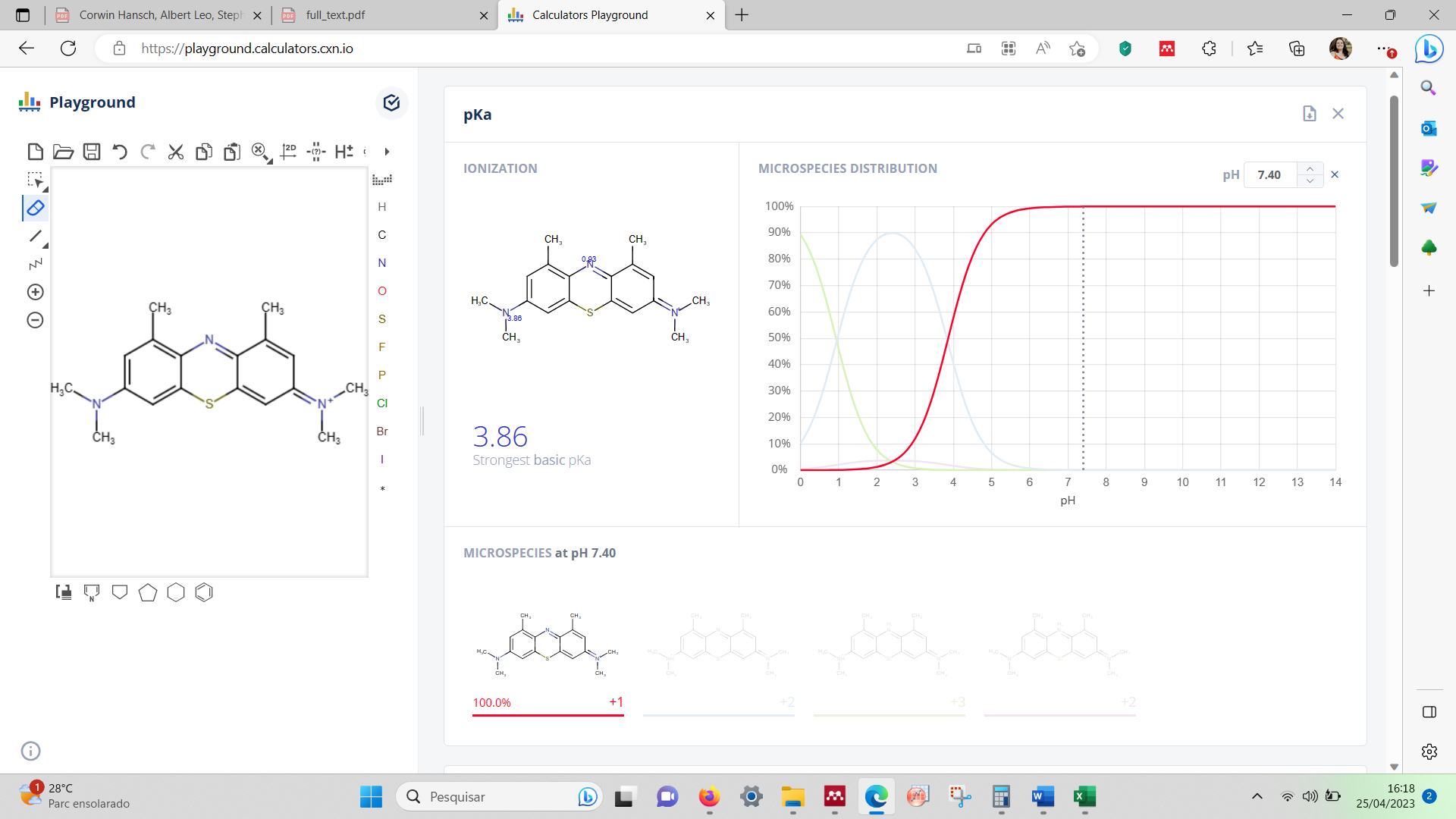


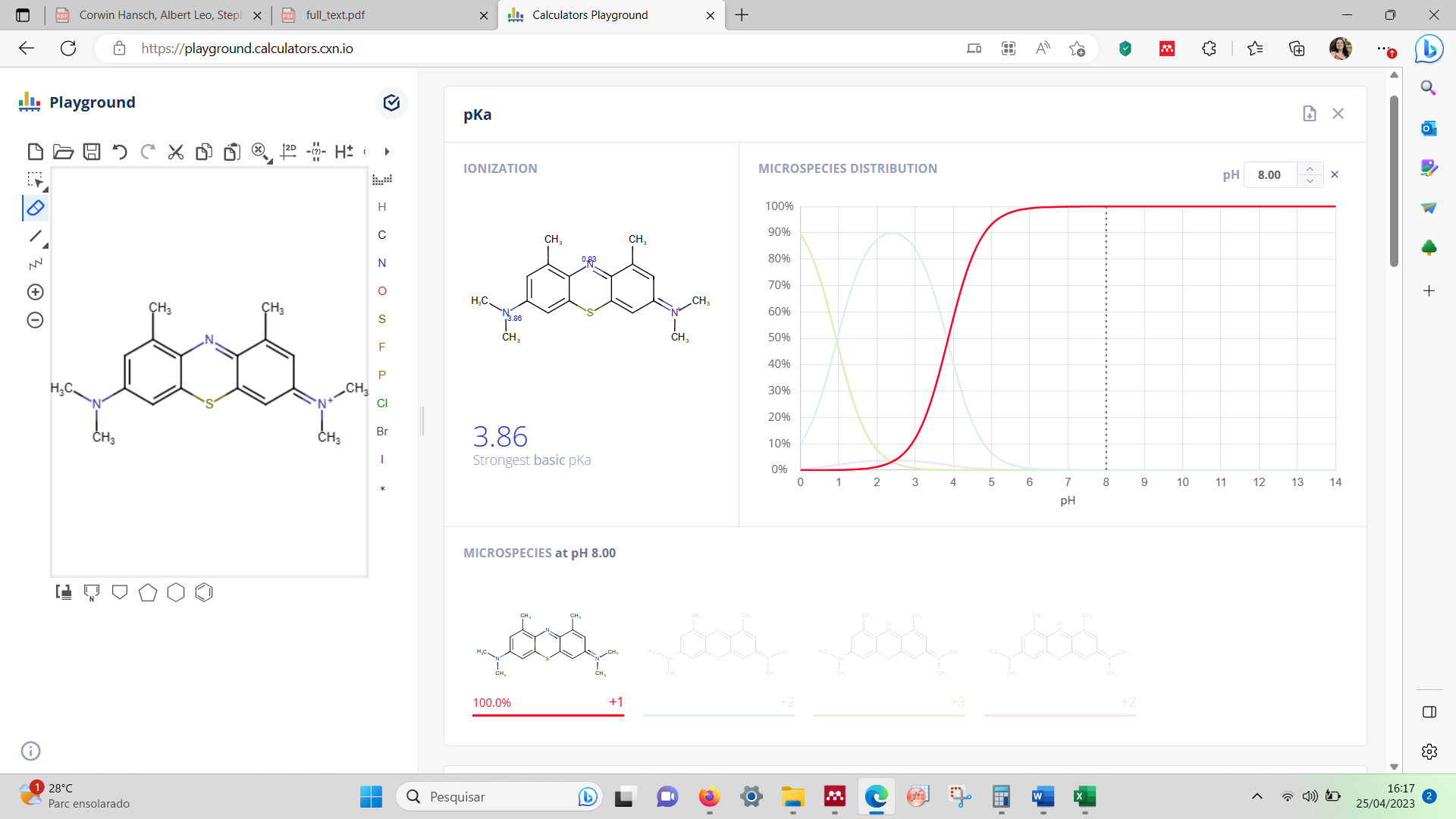


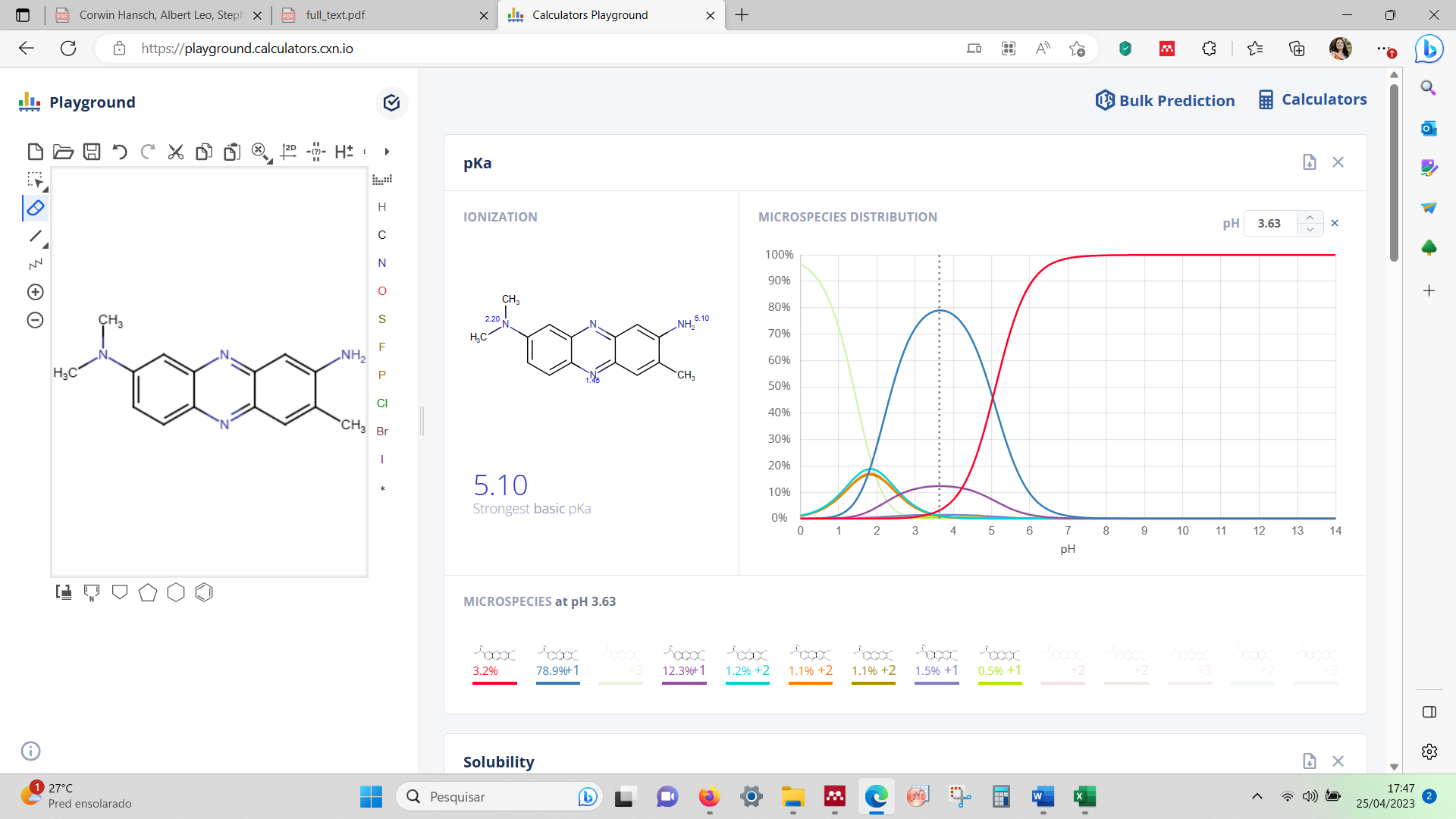


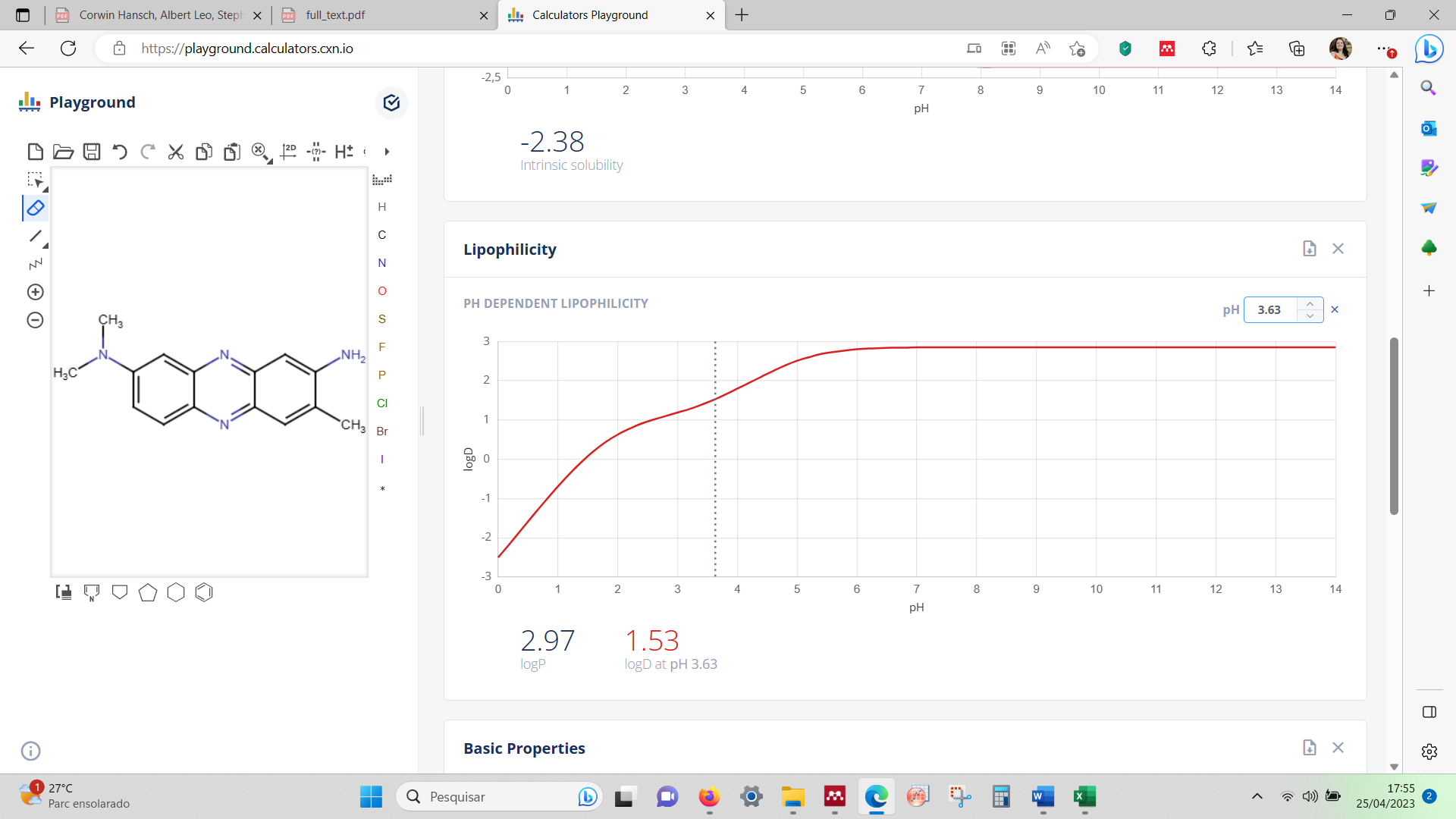


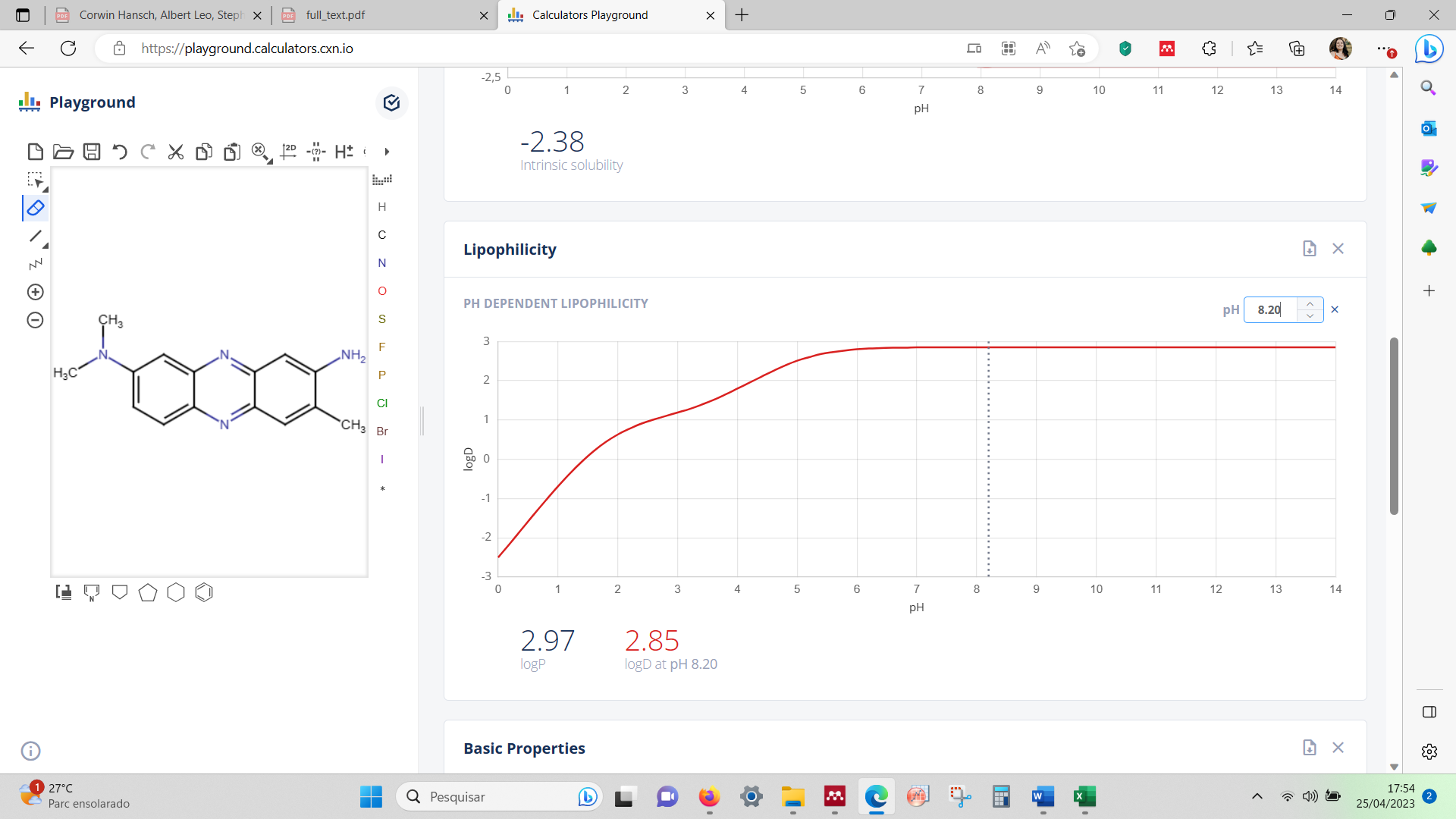


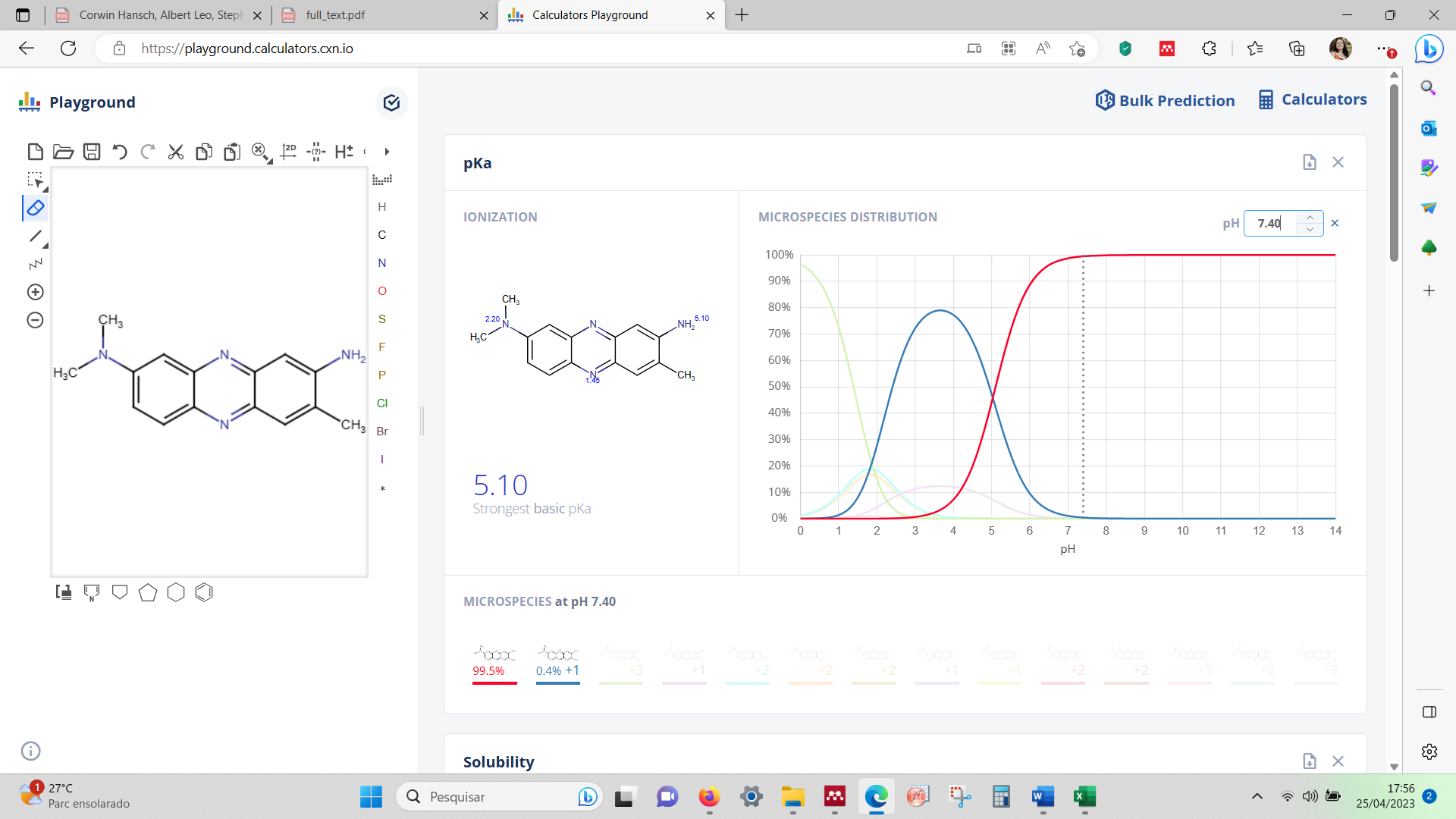


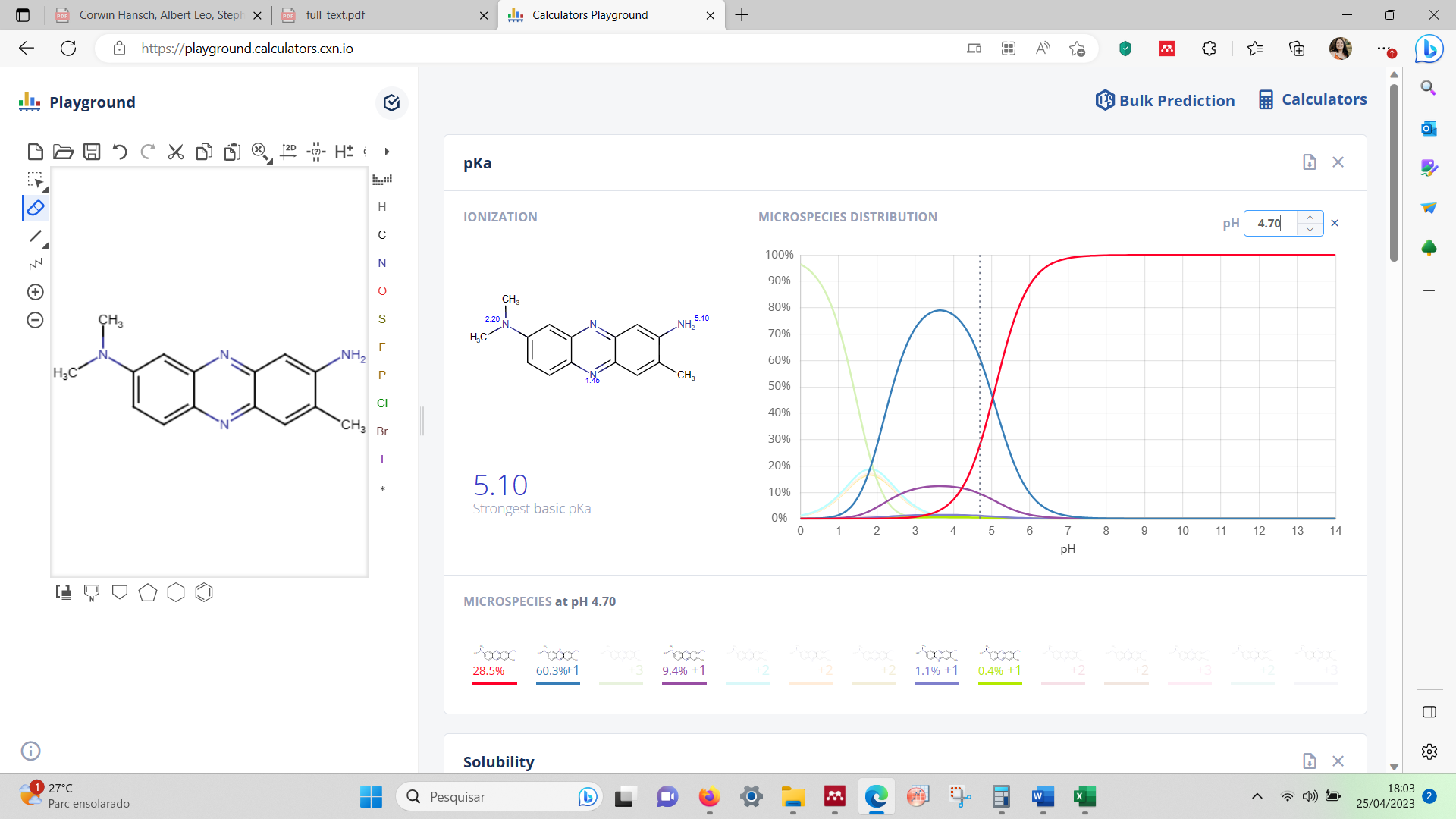


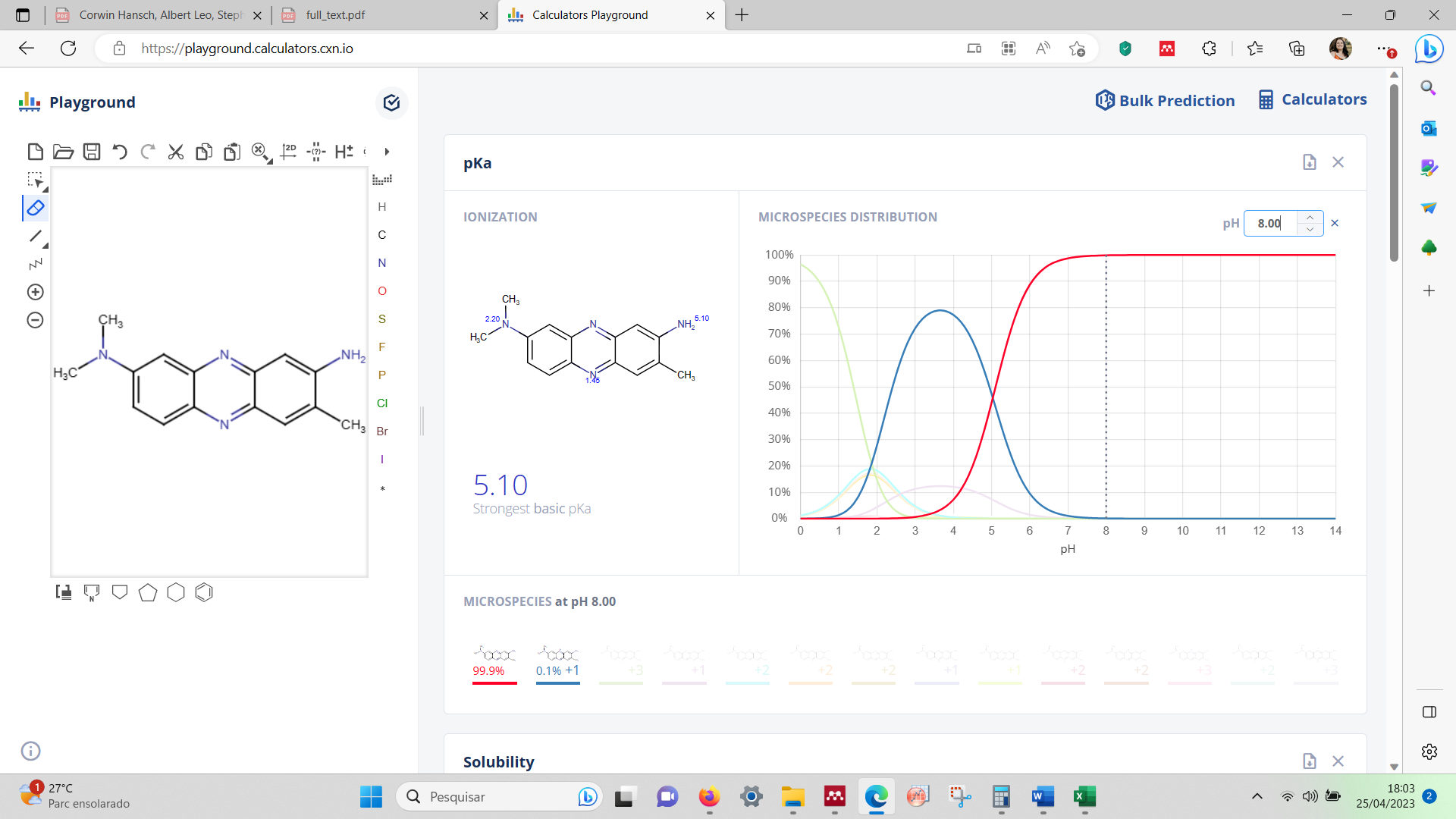


**Supplementary Figure 2** - Distribution of dyes forms under different pHs generated by ChemAxon’s MarvinSketch software (version 23.5).
